# Organocatalytic Synthesis of Spiro-Bridged Heterocyclic Compounds via a Chemoselective Vinylogous Michael/Cyclization/Rearrangement Sequence

**DOI:** 10.1021/acs.joc.5c00443

**Published:** 2025-05-16

**Authors:** I-Ting Chen, Hsuan Lin, Jeng-Liang Han

**Affiliations:** Department of Chemistry, 34916 National Chung Hsing University, Taichung 40227, Taiwan

## Abstract

An organocatalytic cascade reaction of 2-ethylidene 1,3-indandiones and isatylidene-malononitriles has been achieved using quinine as the catalyst. The unexpected vinylogous Michael addition at the β position of isatylidene-malononitriles, followed by aldol cyclization, 1,2-addition of alkoxide to nitrile, and [1,3]-O-to-N rearrangement, leads to the generation of unique spiro-bridged heterocyclic compounds containing amide, indanone, and oxindole moieties in good to excellent yields with high diastereoselectivity.

## Graphical abstract

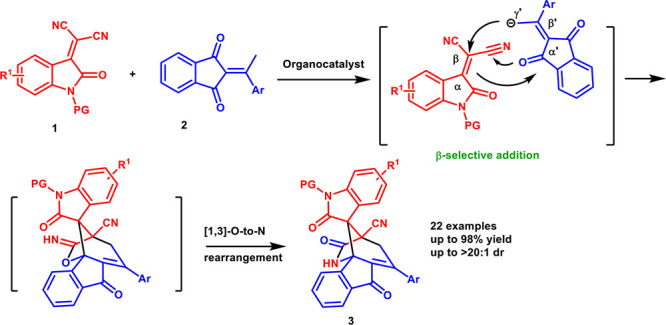

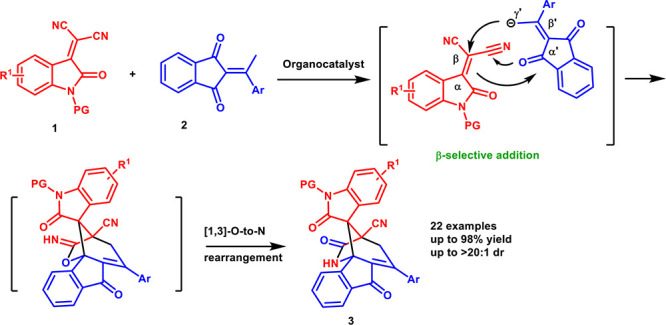

Oxindole alkaloids are an important group of monoterpene alkaloids with spirocyclic oxindole nuclei and are often associated with significant pharmacological activity.^1,2^ Among these, spiro-bridged oxindole frameworks are core structures widely encountered in many natural products (Figure 1). For example, chitsenine **I** and alkaloid **II** exhibit short-lived inhibitory activity *in vivo* of ganglionic transmission in both rats and rabbits.^3^ Spiro-bridged oxindole **III**, prepared by Sakai and co-workers as an analogue of **II**, has been employed in a formulation known to inhibit ulcers.^4^ On the other hand, gelsemine **IV** is a spiro-bridged oxindole alkaloid isolated from flowering plants of the genus *Gelsemium* and exhibited potent and specific antinociception in chronic pain by acting at the three spinal glycine receptors.^5^

**Figure 1:**
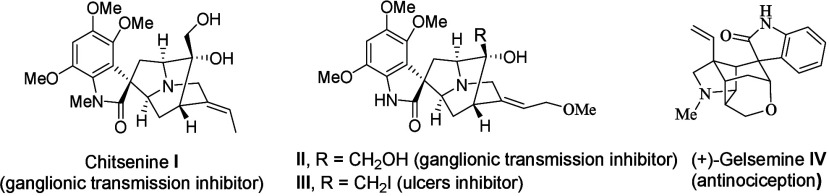
Selected natural products containing a spiro-bridged oxindole moiety.

The combinatorial pharmacophore strategy has played a central role in the search of leading compounds for new drug development.^6^ Oxindole,^7^ indanone,^8^ and amide^9^ fragments are three well-known pharmacophores and are found in various natural and bioactive molecules (Figure 2). However, most established protocols furnish the products containing only oxindole and indanone pharmacophores in spiro-fused heterocyclic systems.^10^ To the best of our knowledge, there have been no reports on the construction of compounds combining all of them in one spiro-bridged heterocyclic molecule, which may potentially have biological activities. Hence, it is highly desirable to develop efficient synthetic procedures for the synthesis of spiro-bridged heterocyclic compounds containing these three potential pharmacophores.

**Figure 2:**
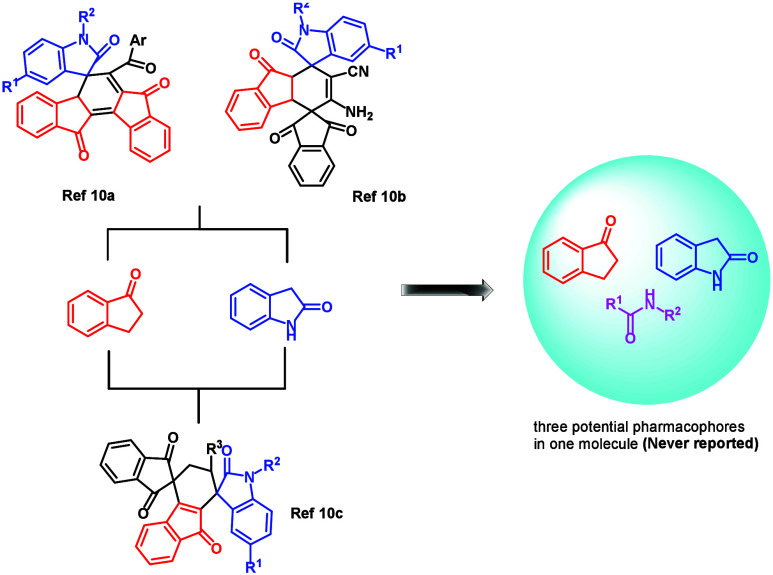
Selected compounds containing oxindole and indanone moieties.

Isatylidene-malononitriles, which are a class of *α,α*-dicyanoolefins, have been widely employed as versatile acceptors or dienophiles in various reactions.^11^ In the past few years, significant progress has been made in the development of organocatalyzed annulation reactions using isatylidene-malononitriles as electrophiles by a number of publications in the literature.^12^ In this regard, the annulation reactions are known to take place exclusively at the α position first by nucleophilic addition, and then electrophilic sites are attacked from the β position (Scheme 1a, path A). In contrast, the annulation reactions take place exclusively at the β position first by nucleophilic addition, and then electrophilic sites are attacked from the α position, which remains rare (Scheme 1a, path B). Recently, Namboothiri and co-workers reported a vinylogous Michael addition of nitroalkylideneoxindoles to isatylidene-malononitriles in the regioselective synthesis of dispirocyclopentylbisoxindoles.^13^ To the best of our knowledge, this is the only example of nucleophilic addition to isatylidene-malononitriles at the β position first. Hence, inspired by the work mentioned above, we envisioned that 2-ethylidene 1,3-indandiones **2** will also proceed via vinylogous Michael addition at the β position of isatylidene-malononitriles **1** to the construction of spirooxindoles with an indandione moiety. However, we found that the obtained products contained amide, indanone, and oxindole moieties. Moreover, we discovered that the amide group formed via a [1,3]-O-to-N rearrangement of allylic imidates **A**.^14^ It is interesting because this type of rearrangement usually has been accomplished in the presence of a Lewis acid catalyst.^15^ The base-catalyzed [1,3]-O-to-N rearrangement is underexplored. Continuing our efforts to develop efficient vinylogous reactions with 2-ethylidene indane-1,3-diones,^16^ we herein report the successful execution of an unusual cascade reaction to establish unique spiro-bridged heterocyclic compounds containing amide, indanone, and oxindole moieties.

**Scheme 1:**
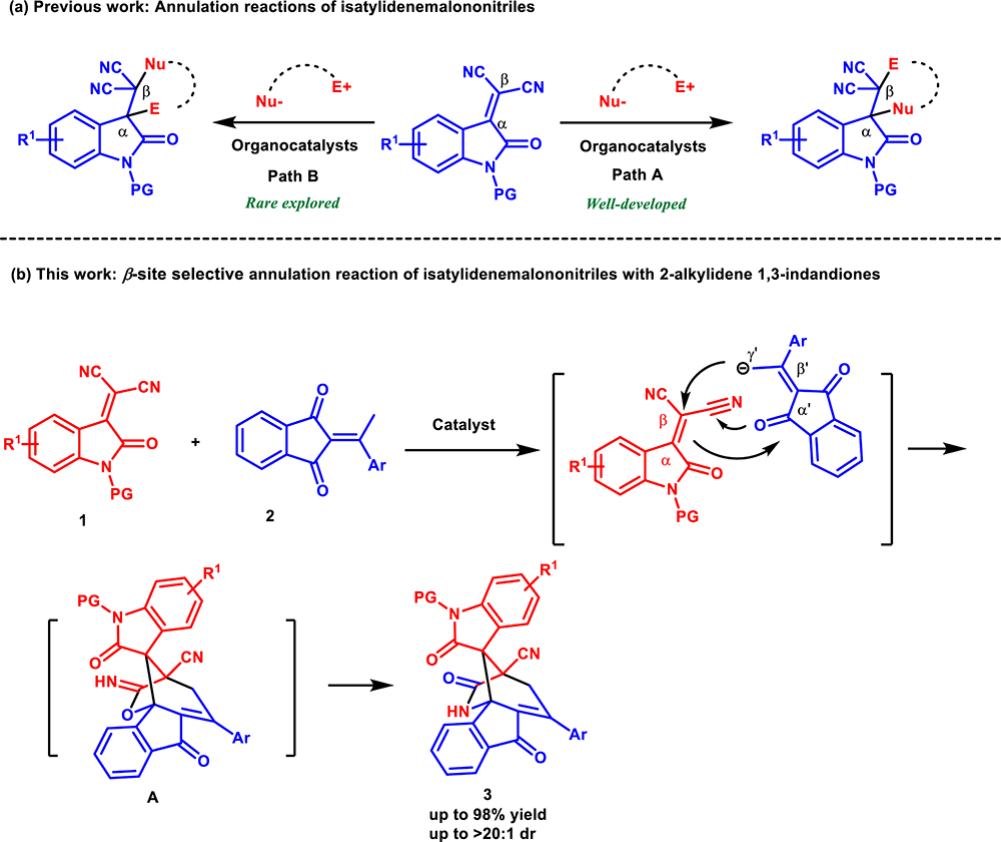
Site Selectivity of Isatylidene-malononitriles

At the outset of the investigation, the reaction between 2-ethylidene 1,3-indandione **1a** and isatylidene-malononitrile **2a** was employed as the model reaction for optimization (Table 1). First, several inorganic bases were evaluated in CH_2_Cl_2_, and all of them yielded moderate results (entries 1–3). Subsequently, various organic bases were used to catalyze the reaction. However, both the yield and diastereoselectivity were lower than those of inorganic bases (entries 4–6). To our delight, quinine, which forms one hydrogen bond between reactants, delivered the product with the highest yield and good diastereoselectivity (entry 7). The investigations of various solvents showed that THF gave a similar diastereoselectivity and a better product yield (entries 8–11).

**Table 1:**
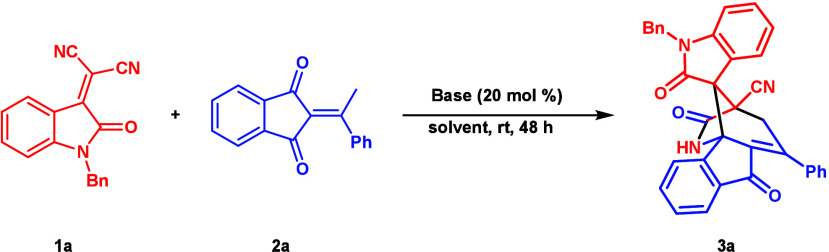
Optimization of the Reaction Conditions^a^

entry	base	solvent	yield (%)^b^	dr^c^
1	K_2_CO_3_	CH_2_Cl_2_	62	>20:1
2	K_3_PO_4_	CH_2_Cl_2_	56	>20:1
3	NaOH	CH_2_Cl_2_	58	>20:1
4	DABCO	CH_2_Cl_2_	52	7:1
5	Et_3_N	CH_2_Cl_2_	50	11:1
6	DMAP	CH_2_Cl_2_	52	12:1
7	quinine	CH_2_Cl_2_	87	17:1
8	quinine	toluene	88	15:1
9	quinine	THF	92	16:1
10	quinine	EA	83	>20:1
11	quinine	CH_3_CN	51	11:1
12^d^	quinine	THF	91	>20:1
13^e^	quinine	THF	93	>20:1
14,^f^	quinine	THF	80	>20:1
15^e^,^g^	quinine	THF	85	>20:1

a Unless otherwise noted, the reaction was carried out by using 0.15 mmol of **2a**, 0.1 mmol of **1a**, and 20 mol % base in 0.25 mL of solvent at RT (25 °C) for 48 h.

b Isolated yield.

c Detemined by NMR analysis of the crude mixture.

d With 0.5 mL of THF.

e With 1.0 mL of THF.

f With 2.0 mL of THF.

g With 10 mol % base.

Upon treatment of the reaction mixture with different concentrations, 0.1 M was identified as the best concentration to promote diastereoselectivity with a slightly increased yield (entries 12–14). Reducing the amount of base resulted in a decreased yield (entry 15). Finally, the optimal conditions were selected by conducting the reaction at room temperature in THF with 20 mol % quinine at a substrate concentration of 0.1 M (entry 13).

With the optimal conditions established, we next examined the scope of various isatylidene-malononitriles **1** and 2-ethylidene 1,3-indandiones **2**. As depicted in Scheme 2, all reactions proceeded well under the described conditions, affording the desired products in moderate to high isolated yields with excellent levels of diastereoselectivity. First, the positions and electronic properties of isatin substituents were tested.

**Scheme 2:**
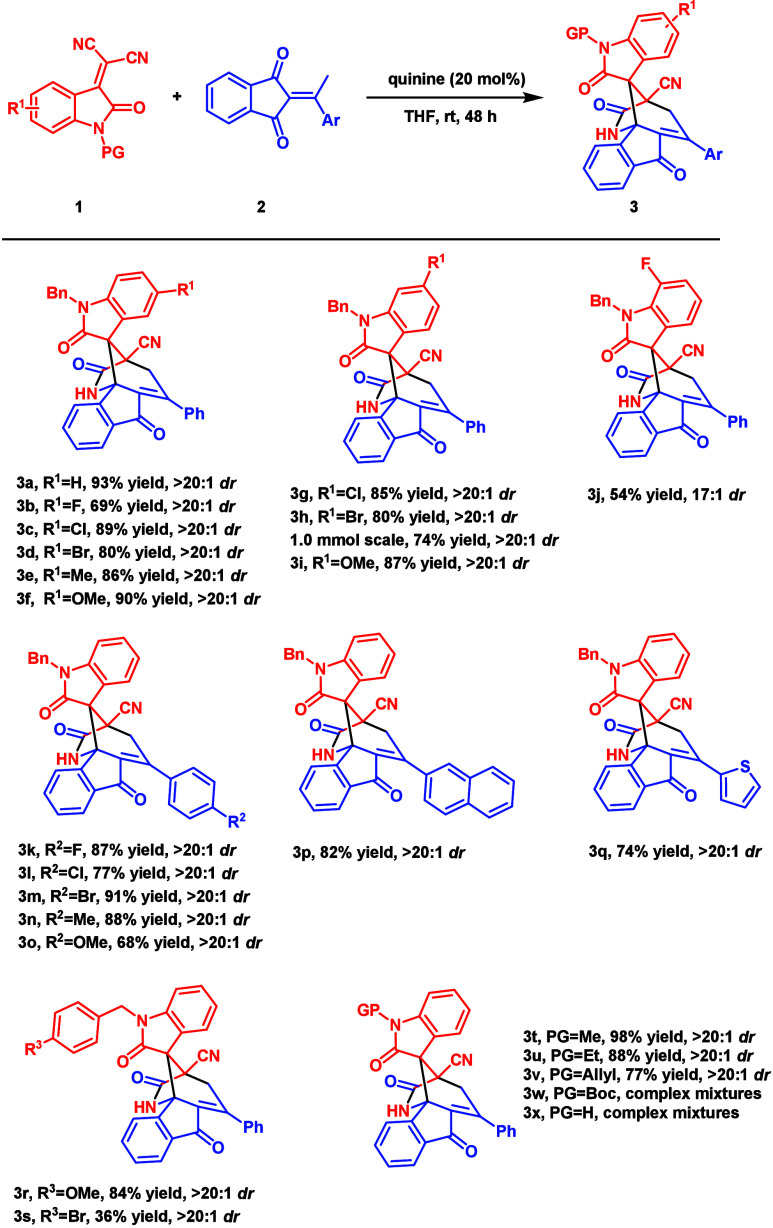
Substrate Scope^a^

a Reaction conditions: 0.15 mmol of **2a**, 0.1 mmol of **1a**, and 20 mol % quinine in 1.0 mL of THF at RT (25 °C) for 48 h. Isolated yield.

Notably, the 5-F- and 7-F-substituted isatylidene-malononitriles gave lower yields of products **3b** (69%) and **3j** (54%), respectively. The lower reactivity of fluoro-substituted isatylidene-malononitriles was observed. It is possible that the fluorine atom generates hydrogen bonding with the catalyst and retards the turnover efficiency of the catalyst.

Indandione **2a** could react with electron-rich and electron-deficient isatylidene-malononitriles and provided products **3c**–**i** in high yields (80–90%) with high diastereoselectivity (>20:1). The scale-up reaction for **1h** and **2a** also proceeded smoothly to give product **3h** in 74% isolated yield and >20:1 dr. This result is also similar to the small-scale reaction. Subsequent investigations then focused on the different aryl substitutions on 2-ethylidene 1,3-indandiones **2**. In all cases, the reactions proceeded well, affording **3k**–**q** in good to high yields (68–91%) and excellent stereoselectivities (>20:1 dr). Furthermore, the effect of protecting groups of isatylidene-malononitriles **1** was also investigated, leading to the formation of products **3r**–**3v** with excellent diastereoselectivity (>20:1). However, complex mixtures were observed without protection or when the protecting group was replaced with an electron-withdrawing group (Boc) (**3w** and **3x**). The structure of **3** was confirmed using single-crystal X-ray diffraction analysis of **3f** (CCDC 2370395).^17^

We then carried out the functionalization of product **3h**. Treatment of **3h** with Boc_2_O under basic conditions resulted in the protection of amide and ketone groups, leading to the formation of di-Boc-protected product **4** in 75% reaction yield (Scheme 3a). The protection of ketone was unexpected, and it might be from the deprotonation of the γ position of the ketone by a base. To prove this, we then performed a deuterium labeling study under basic conditions and obtained product **3h-*d*_2_** in 69% yield with 76% deuterium labeling (Scheme 3b).

**Scheme 3:**
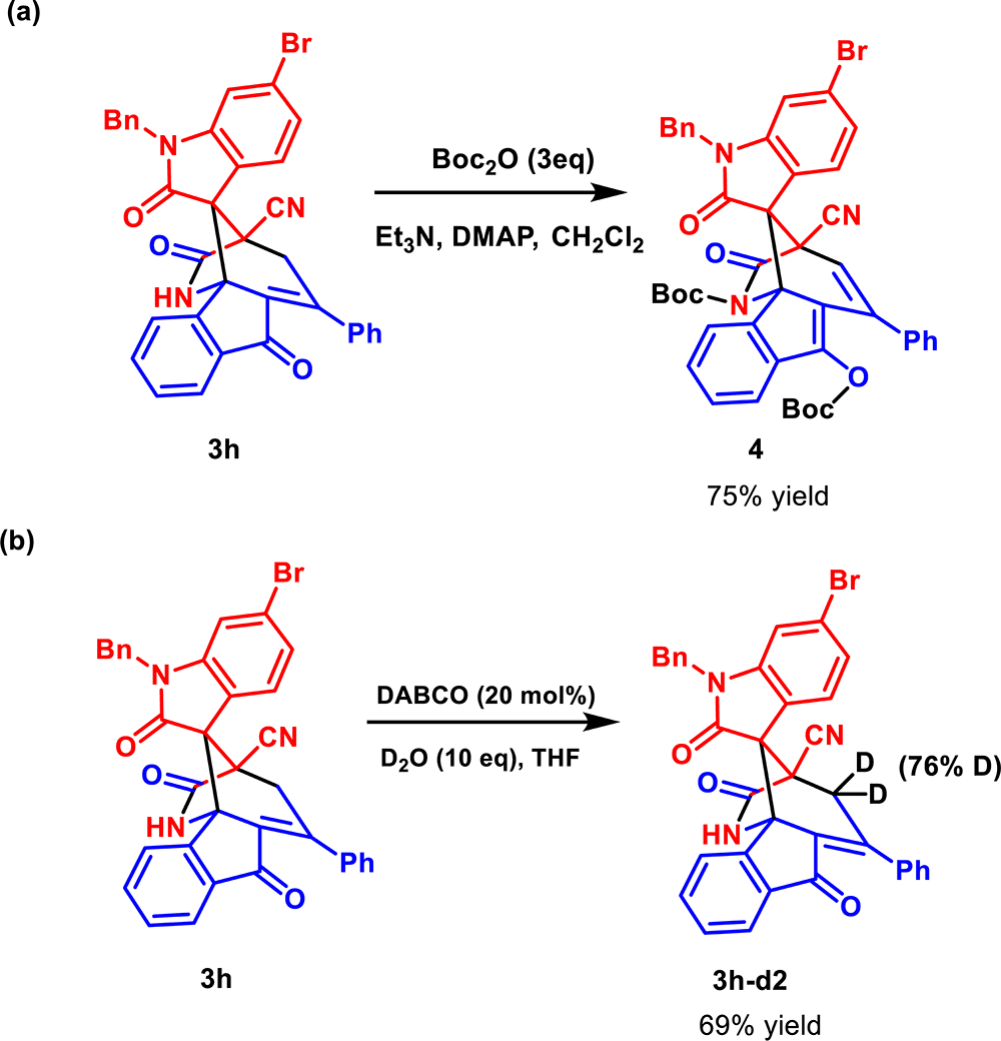
Chemical Transformation and Deuterium Labeling of **3h**

On the basis of previous work^12,13^ and a deuterium labeling study (Scheme 3b), a plausible catalytic cycle for the tertiary amine-catalyzed reaction is proposed in Scheme 4. First, the acidic hydrogen of 2-ethylidene 1,3-indandione **2a** is deprotonated by the catalyst to generate **Int-1**. Furthermore, **Int-1** undergoes Michael addition to the β position of electrophile **1a**, forming intermediate **Int-2**. Next, conjugated electrons attack the carbonyl group of indandione to form **Int-3**. The 1,2-addition of alkoxide to nitrile afforded imidate **Int-4** after protonation. Finally, the deprotonation of the γ position of ketone by a base triggered a [1,3]-O-to-N rearrangement of allylic imidate **Int-4** and delivered product **3a**.

**Scheme 4:**
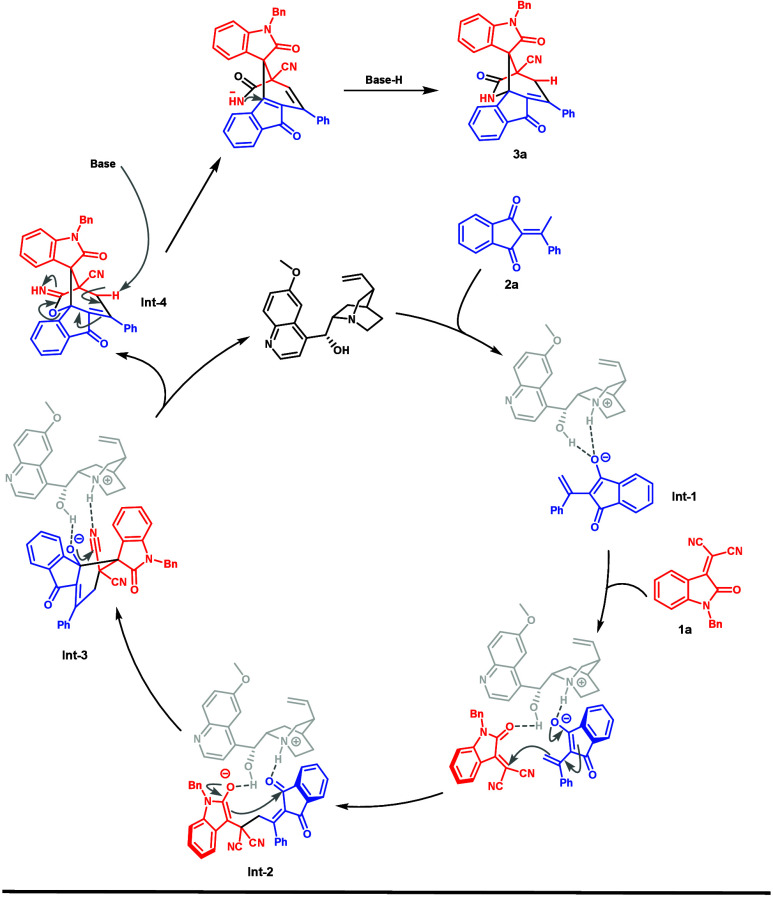
Plausible Reaction Mechanism

To gain further mechanistic insights into the process, we conducted density functional theory (DFT) calculations performed at the M06-2X-D3/def2-TZVPP/SMD­(THF)//B3LYP/6-311G­(d,p)/IEFPCM­(THF) theoretical level.

In the catalytic cycles listed in Scheme 4, a comprehensive depiction of the energy profiles for this cascade reaction is provided in Figure 3. The structure of the catalyst was simplified to DABCO for the following DFT calculation. The energy of **TS2**, where the nucleophile adds to the β position of **1a**, is lower than the energy of the nucleophile addition at the α position of **1a** (**TS1**). Hence, the vinylogous Michael addition of 1,3-indandione **1a** at the β position of **1a** is preferred. Enolate **Int-6**, which originates from the addition of a nucleophile to the β position of **1a**, then attacks the carbonyl group of indandione with a barrier of 9.5 kcal mol^–1^. The 1,2-addition of alkoxide to nitrile afforded imidate **Int-8** after protonation. Although the deprotonation of the γ position of ketone by a base has a higher energy barrier (14.8 kcal mol^–1^), the barrier for the rearrangement of allylic imidate **Int-9** to amide anion **Int-10** is lower than that of the deprotonation step (10.9 kcal mol^–1^). The intramolecular aza-Michael addition to enone to form product **3a** suffers a high barrier of 24.8 kcal mol^–1^ due to steric hindrance. However, product **3a** is exothermically formed by 39.2 kcal mol^–1^, indicating the formation of **3a** is thermodynamically favorable.

**Figure 3:**
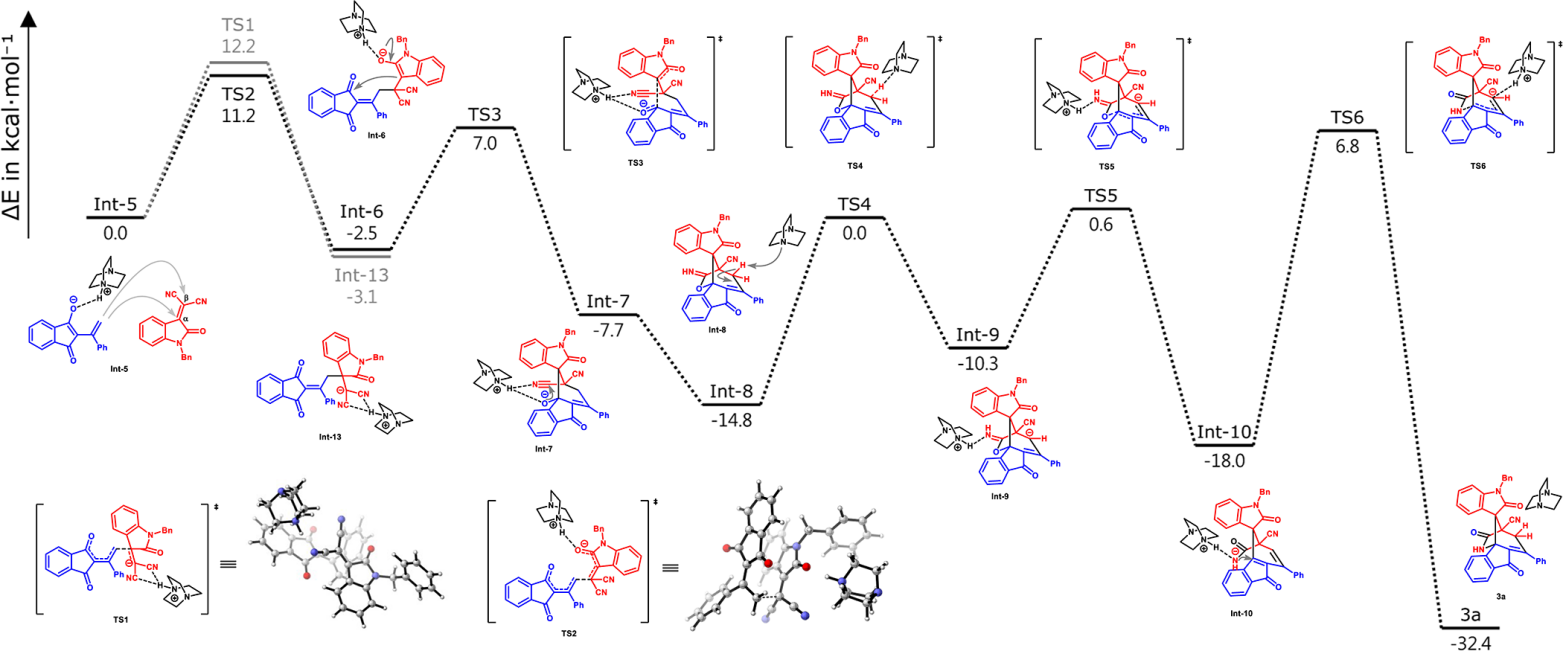
Energy profiles for the cascade reaction of **1a** and **2a** catalyzed by DABCO. The relative free energies are given in kilocalories per mole.

The asymmetric version of this cascade reaction was also investigated. Preliminary screening of chiral organocatalysts was conducted in CH_2_Cl_2_ at room temperature. The enantioselectivity and diastereoselectivity were moderate (see the Supporting Information for more details). We have not obtained satisfactory results so far.

In summary, we have developed an organocatalytic cascade reaction of 2-ethylidene 1,3-indandiones **2** and isatylidene-malononitriles **1** using quinine as the catalyst. The unexpected vinylogous Michael addition at the β position of isatylidene-malononitriles, followed by aldol cyclization, 1,2-addition of alkoxide to nitrile, and [1,3]-O-to-N rearrangement, led to the generation of unique spiro-bridged heterocyclic compounds containing amide, indanone, and oxindole moieties in good to excellent yields with high diastereoselectivities. The DFT calculations were conducted to demonstrate why the vinylogous Michael addition occurred at the β position of the isatylidene-malononitriles.

## Experimental Section

All commercially available reagents were used without further purification unless otherwise stated. All of the reaction solvents were purified before use. Proton nuclear magnetic resonance (^1^H NMR) spectra were recorded on a commercial instrument at 400 MHz. Carbon-13 nuclear magnetic resonance (^13^C­{^1^H} NMR) spectra were recorded at 100 MHz. The proton signal for a residual nondeuterated solvent (δ 7.26 for CHCl_3_) was used as an internal reference for ^1^H NMR spectra. For ^13^C­{^1^H} NMR spectra, chemical shifts are reported relative to the δ 77.0 resonance of CHCl_3_. Coupling constants are reported in hertz. Melting points were determined on a BUCHI B-545 melting point apparatus and were uncorrected. High-resolution mass spectra were recorded on a Thermo Fisher Scientific LTQ Orbitrap XL mass spectrometer. The single crystal was measured by a Bruker D8 VENTURE X-ray single-crystal diffractometer. Analytical thin-layer chromatography (TLC) was performed on silica gel 60 F254 precoated plates with visualization under UV light. Column chromatography was generally performed using 40–63 μm (230–400 mesh) silica gel, typically using a 50–100:1 weight ratio of silica gel to the crude product. Isatylidene-malononitriles **1** and spiroindane-1,3-diones **2** were prepared according to known procedures.^18,19^

### General Procedure for the Synthesis of Compounds **3**

To a 7 mL vial equipped with a stirring bar were added **1** (0.10 mmol, 1 equiv), **2** (0.15 mmol, 1.5 equiv), and quinine (20 mol %). Then, 1.0 mL of THF was added to the mixture, and the resulting reaction mixture was allowed to stir at room temperature for 48 h. After completion of the reaction as indicated by TLC, the solvent was removed under reduced pressure and the resulting residue was purified by column chromatography on silica gel using a hexane/ethyl acetate eluent at 7:1 to 5:1 to 3:1 to afford **3**.

### Synthesis of **3h** on a 1.0 mmol Scale

To a 25 mL vial equipped with a stirring bar were added **1h** (364 mg, 1.0 mmol), **2** (372 mg, 0.15 mmol), and quinine (65 mg, 0.2 mmol, 20 mol %). Then, 10 mL of THF was added to the mixture, and the resulting reaction mixture was allowed to stir at room temperature for 48 h. After completion of the reaction as indicated by TLC, the solvent was removed under reduced pressure and the resulting residue was purified by column chromatography on silica gel using a hexane/ethyl acetate eluent at 7:1 to 5:1 to 3:1 to afford **3h** (453 mg, 74% yield).

### 3′-((λ^2^-Azaneylidene)-λ^3^-methyl)-1-benzyl-5′-phenyl-3′,4′-dihydro-1′*H*-spiro­[indoline-3,11′-[3,10*b*]­methanoindeno­[1,2-*b*]­azepine]-2,2′,6′-trione (**3a**)

Following the general procedure with **1a** (28.5 mg, 0.10 mmol), **2a** (37.2 mg, 0.15 mmol), and quinine (6.8 mg, 20 mol %), the title compound was obtained as an orange powder after purification by column chromatography on silica gel using a hexane/ethyl acetate eluent (7:1 to 5:1 to 3:1): yield 93% (49.6 mg); dr >20:1; mp 201.5–202.5 °C; ^1^H NMR (400 MHz, CDCl_3_) δ 7.67–7.61 (m, 4H), 7.58–7.50 (m, 4H), 7.45–7.39 (m, 2H), 7.34–7.28 (m, 3H), 7.28–7.25 (m, 2H), 7.18–7.12 (m, 2H), 6.889 (t, *J* = 7.7 Hz, 1H), 6.72 (d, *J* = 7.9 Hz, 1H), 5.05–4.96 (m, 2H), 3.60 (d, *J* = 20.2 Hz, 1H), 3.38 (d, *J* = 20.3 Hz, 1H); ^13^C­{^1^H}­NMR (101 MHz, CDCl_3_) δ 186.2, 173.3, 168.5, 148.0, 143.8, 140.6, 140.4, 136.7, 135.8, 134.7, 134.2, 131.6, 131.3, 131.0, 129.1, 129.0, 128.8, 128.64, 128.60, 128.1, 127.8, 127.63, 127.59, 125.8, 125.5, 124.7, 123.6, 119.6, 115.6, 110.3, 66.1, 61.2, 50.3, 44.4, 36.2; HRMS (ESI) calcd for C_35_H_23_N_3_O_3_Na [M + Na]^+^ 556.1632, found 556.1629.

### 3′-((λ^2^-Azaneylidene)-λ^3^-methyl)-1-benzyl-5-fluoro-5′-phenyl-3′,4′-dihydro-1′*H*-spiro­[indoline-3,11′-[3,10*b*]­methanoindeno­[1,2-*b*]­azepine]-2,2′,6′-trione (**3b**)

Following the general procedure with **1b** (30.3 mg, 0.10 mmol), **2a** (37.2 mg, 0.15 mmol), and quinine (6.8 mg, 20 mol %), the title compound was obtained as an orange powder after purification by column chromatography on silica gel using a hexane/ethyl acetate eluent (7:1 to 5:1 to 3:1): yield 69% (38.0 mg); dr >20:1; mp 233.8–234.8 °C; ^1^H NMR (400 MHz, CDCl_3_) δ 7.73–7.71 (m, 1H), 7.67–7.60 (m, 3H), 7.58–7.51 (m, 3H), 7.51–7.44 (m, 2H), 7.36–7.31 (m, 4H), 7.28–7.25 (m, 2H), 6.93–6.88 (m, 2H), 6.66 (dd, *J* = 9.2, 4.4 Hz, 1H), 5.02 (s, 2H), 3.57 (d, *J* = 20.3 Hz, 1H), 3.43 (d, *J* = 20.3 Hz, 1H); ^13^C­{^1^H} NMR (101 MHz, CDCl_3_) δ 185.9, 173.1, 168.2, 160.2, 157.8 (d, *J* = 244.4 Hz), 147.8, 140.5, 140.3, 139.9, 136.4, 135.9, 134.4, 134.0, 131.8, 131.4, 129.1, 128.8, 128.6, 128.2, 127.6, 125.4, 124.9, 121.0 (d, *J* = 13.1 Hz), 117.6 (d, *J* = 23.2 Hz), 115.4, 114.4, 114.1, 111.0 (d, *J* = 8.1 Hz), 66.1, 61.3, 50.2, 44.6, 36.2; ^19^F­{^1^H} NMR (376 MHz, CDCl_3_) δ −117.2; HRMS (ESI) calcd for C_35_H_22_N_3_O_3_FNa [M + Na]^+^ 574.1537, found 574.1542.

### 3′-((λ^2^-Azaneylidene)-λ^3^-methyl)-1-benzyl-5-chloro-5′-phenyl-3′,4′-dihydro-1′*H*-spiro­[indoline-3,11′-[3,10*b*]­methanoindeno­[1,2-*b*]­azepine]-2,2′,6′-trione (**3c**)

Following the general procedure with **1c** (32.0 mg, 0.10 mmol), **2a** (37.2 mg, 0.15 mmol), and quinine (6.8 mg, 20 mol %), the title compound was obtained as a brick red powder after purification by column chromatography on silica gel using a hexane/ethyl acetate eluent (7:1 to 5:1 to 3:1): yield 89% (50.6 mg); dr >20:1; mp 200.0–201.0 °C; ^1^H NMR (400 MHz, acetone-*d*_6_) δ 8.63 (s, 1H), 7.89–7.87 (m, 2H), 7.65–7.60 (m, 3H), 7.59–7.55 (m, 4H), 7.44–7.42 (m, 2H), 7.37–7.33 (m, 3H), 7.28–7.24 (m, 2H), 6.96 (d, *J* = 8.4 Hz, 1H), 5.27 (d, *J* = 15.7 Hz, 1H), 5.03 (d, *J* = 15.7 Hz, 1H), 4.07 (d, *J* = 20.6 Hz, 1H), 3.45 (d, *J* = 20.6 Hz, 1H); ^13^C­{^1^H} NMR (101 MHz, acetone-*d*_6_) δ 186.6, 173.8, 168.5, 148.8, 143.7, 141.9, 141.2, 137.7, 136.6, 136.0, 135.7, 132.4, 131.5, 131.4, 129.8, 129.6, 129.2, 128.7, 128.6, 127.4, 126.3, 124.8, 122.7, 116.9, 112.2, 66.9, 61.4, 50.9, 44.6, 36.8; HRMS (ESI) calcd for C_35_H_22_N_3_O_3_ClNa [M + Na]^+^ 590.1242, found 590.1240.

### 3′-((λ^2^-Azaneylidene)-λ^3^-methyl)-1-benzyl-5-bromo-5′-phenyl-3′,4′-dihydro-1′*H*-spiro­[indoline-3,11′-[3,10*b*]­methanoindeno­[1,2-*b*]­azepine]-2,2′,6′-trione (**3d**)

Following the general procedure with **1d** (36.4 mg, 0.10 mmol), **2a** (37.2 mg, 0.15 mmol), and quinine (6.8 mg, 20 mol %), the title compound was obtained as a brick red powder after purification by column chromatography on silica gel using a hexane/ethyl acetate eluent (7:1 to 5:1 to 3:1): yield 80% (48.7 mg); dr >20:1; mp 216.9–217.9 °C; ^1^H NMR (400 MHz, acetone-*d*_6_) δ 8.62 (s, 1H), 7.88–7.85 (m, 2H), 7.64 (dt, *J* = 7.1, 1.3 Hz, 1H), 7.63–7.60 (m, 2H), 7.60–7.54 (m, 4H), 7.45–7.44 (m, 1H), 7.43–7.40 (m, 3H), 7.38–7.32 (m, 3H), 6.92 (dd, *J* = 8.1, 0.7 Hz, 1H), 5.26 (d, *J* = 15.7 Hz, 1H), 5.02 (d, *J* = 15.7 Hz, 1H), 4.05 (d, *J* = 20.6 Hz, 1H), 3.45 (d, *J* = 20.6 Hz, 1H); ^13^C­{^1^H} NMR (101 MHz, acetone-*d*_6_) δ 186.6, 173.7, 168.5, 148.8, 144.1, 141.9, 141.2, 137.7, 136.6, 136.0, 135.7, 134.3, 132.4, 131.5, 130.2, 130.0, 129.8, 129.6, 129.2, 128.74, 128.65, 126.3, 124.8, 123.0, 116.9, 116.0, 112.7, 66.9, 61.6, 50.9, 44.6, 36.9; HRMS (ESI) calcd for C_35_H_22_N_3_O_3_BrNa [M + Na]^+^ 634.0737, found 634.0736.

### 3′-((λ^2^-Azaneylidene)-λ^3^-methyl)-1-benzyl-5-methyl-5′-phenyl-3′,4′-dihydro-1′*H*-spiro­[indoline-3,11′-[3,10*b*]­methanoindeno­[1,2-*b*]­azepine]-2,2′,6′-trione (**3e**)

Following the general procedure with **1e** (29.9 mg, 0.10 mmol), **2a** (37.2 mg, 0.15 mmol), and quinine (6.8 mg, 20 mol %), the title compound was obtained as a red powder after purification by column chromatography on silica gel using a hexane/ethyl acetate eluent (7:1 to 5:1 to 3:1): yield 86% (46.9 mg); dr >20:1; mp 181.7–182.7 °C; ^1^H NMR (400 MHz, CDCl_3_) δ 7.65–7.63 (m, 1H), 7.63–7.60 (m, 2H), 7.59–7.57 (m, 1H), 7.54–7.51 (m, 2H), 7.50–7.49 (m, 1H), 7.43–7.36 (m, 3H), 7.29–7.21 (m, 5H), 6.95–6.93 (m, 2H), 6.60 (d, *J* = 8.4 Hz, 1H), 5.01–4.91 (m, 2H), 3.56 (d, *J* = 20.2 Hz, 1H), 3.40 (d, *J* = 20.3 Hz, 1H), 2.14 (s, 3H); ^13^C­{^1^H} NMR (101 MHz, CDCl_3_) δ 186.3, 173.2, 168.6, 147.8, 141.4, 140.7, 140.4, 136.7, 135.8, 134.9, 134.4, 133.0, 131.5, 131.3, 131.1, 128.9, 128.7, 128.5, 128.0, 127.6, 126.7, 125.6, 124.7, 119.6, 115.6, 110.1, 66.1, 61.1, 50.2, 44.4, 36.4, 21.4; HRMS (ESI) calcd for C_36_H_25_N_3_O_3_Na [M + Na]^+^ 570.1788, found 570.1778.

### 3′-((λ^2^-Azaneylidene)-λ^3^-methyl)-1-benzyl-5-methoxy-5′-phenyl-3′,4′-dihydro-1′*H*-spiro­[indoline-3,11′-[3,10*b*]­methanoindeno­[1,2-*b*]­azepine]-2,2′,6′-trione (**3f**)

Following the general procedure with **1f** (31.5 mg, 0.10 mmol), **2a** (37.2 mg, 0.15 mmol), and quinine (6.8 mg, 20 mol %), the title compound was obtained as a yellow orange powder after purification by column chromatography on silica gel using a hexane/ethyl acetate eluent (7:1 to 5:1 to 3:1): yield 90% (50.6 mg); dr >20:1; mp 200.0–201.0 °C; ^1^H NMR (400 MHz, acetone-*d*_6_) δ 8.58 (s, 1H), 7.91–7.89 (m, 2H), 7.66–7.63 (m, 1H), 7.63–7.58 (m, 2H), 7.58–7.52 (m, 4H), 7.44–7.41 (m, 2H), 7.36–7.30 (m, 3H), 6.85–6.74 (m, 3H), 5.21 (d, *J* = 15.7 Hz, 1H), 4.97 (d, *J* = 15.7 Hz, 1H), 4.06 (d, *J* = 20.4 Hz, 1H), 3.54 (s, 3H), 3.39 (d, *J* = 20.4 Hz, 1H); ^13^C­{^1^H} NMR (101 MHz, acetone-*d*_6_) δ 186.8, 173.8, 168.9, 156.6, 148.8, 142.2, 141.2, 137.94, 137.91, 136.5, 136.4, 135.7, 132.2, 131.6, 130.0, 129.5, 129.1, 128.6, 128.5, 126.4, 124.7, 121.9, 117.2, 115.8, 114.2, 111.4, 67.0, 61.8, 55.8, 51.0, 44.5, 36.8; HRMS (ESI) calcd for C_36_H_25_N_3_O_4_Na [M + Na]^+^ 586.1737, found 586.1743.

### 3′-((λ^2^-Azaneylidene)-λ^3^-methyl)-1-benzyl-6-chloro-5′-phenyl-3′,4′-dihydro-1′*H*-spiro­[indoline-3,11′-[3,10*b*]­methanoindeno­[1,2-*b*]­azepine]-2,2′,6′-trione (**3g**)

Following the general procedure with **1g** (32.0 mg, 0.10 mmol), **2a** (37.2 mg, 0.15 mmol), and quinine (6.8 mg, 20 mol %), the title compound was obtained as a red powder after purification by column chromatography on silica gel using a hexane/ethyl acetate eluent (7:1 to 5:1 to 3:1): yield 85% (48.1 mg); dr >20:1; mp 184.8–185.8 °C; ^1^H NMR (400 MHz, CDCl_3_) δ 7.70 (dt, *J* = 7.7, 1.0 Hz, 1H), 7.63–7.61 (m, 2H), 7.57–7.50 (m, 4H), 7.47 (dd, *J* = 7.4, 1.3 Hz, 1H), 7.43 (dd, *J* = 7.5, 1.4 Hz, 1H), 7.35–7.32 (m, 3H), 7.29–7.26 (m, 2H), 7.04–7.02 (m, 2H), 6.87 (dd, *J* = 8.3, 1.9 Hz, 1H), 6.73 (d, *J* = 1.9 Hz, 1H), 5.03–4.94 (m, 2H), 3.57 (d, *J* = 20.3 Hz, 1H), 3.40 (d, *J* = 20.2 Hz, 1H); ^13^C­{^1^H} NMR (101 MHz, CDCl_3_) δ 185.9, 173.3, 168.1, 148.1, 145.2, 140.5, 140.3, 137.3, 136.0, 134.2, 133.9, 131.9, 131.5, 129.2, 128.8, 128.7, 128.4, 127.7, 126.7, 125.3, 125.0, 123.6, 117.9, 115.3, 111.0, 66.0, 61.0, 50.1, 44.6, 36.2; HRMS (ESI) calcd for C_35_H_22_N_3_O_3_ClNa [M + Na]^+^ 590.1242, found 590.1238.

### 3′-((λ^2^-Azaneylidene)-λ^3^-methyl)-1-benzyl-6-bromo-5′-phenyl-3′,4′-dihydro-1′*H*-spiro­[indoline-3,11′-[3,10*b*]­methanoindeno­[1,2-*b*]­azepine]-2,2′,6′-trione (**3h**)

Following the general procedure with **1h** (36.4 mg, 0.10 mmol), **2a** (37.2 mg, 0.15 mmol), and quinine (6.8 mg, 20 mol %), the title compound was obtained as a brick red powder after purification by column chromatography on silica gel using a hexane/ethyl acetate eluent (7:1 to 5:1 to 3:1): yield 80% (49.0 mg); dr >20:1; mp 222.8–223.8 °C; ^1^H NMR (400 MHz, CDCl_3_) δ 7.66 (d, *J* = 7.5 Hz, 1H), 7.61 (d, *J* = 7.3 Hz, 2H), 7.56–7.37 (m, 7H), 7.32–7.30 (m, 3H), 7.25–7.22 (m, 2H), 7.01 (d, *J* = 8.3 Hz, 1H), 6.95 (d, *J* = 8.2 Hz, 1H), 6.85 (s, 1H), 4.98–4.90 (m, 2H), 3.54 (d, *J* = 20.3 Hz, 1H), 3.36 (d, *J* = 20.1 Hz, 1H); ^13^C­{^1^H} NMR (101 MHz, CDCl_3_) δ 186.0, 173.2, 168.3, 147.9, 145.1, 140.4, 140.3, 136.5, 135.9, 134.2, 133.9, 131.8, 131.4, 129.1, 128.8, 128.7, 128.3, 127.6, 126.9, 126.5, 125.4, 125.2, 124.9, 118.5, 115.4, 113.7, 66.0, 61.1, 50.1, 44.5, 36.1; HRMS (ESI) *m*/*z* calcd for C_35_H_21_O_3_N_3_Br [M – H]^−^ 610.0761, found 610.0777.

### 3′-((λ^2^-Azaneylidene)-λ^3^-methyl)-1-benzyl-6-methoxy-5′-phenyl-3′,4′-dihydro-1′*H*-spiro­[indoline-3,11′-[3,10*b*]­methanoindeno­[1,2-*b*]­azepine]-2,2′,6′-trione (**3i**)

Following the general procedure with **1i** (31.5 mg, 0.10 mmol), **2a** (37.2 mg, 0.15 mmol), and quinine (6.8 mg, 20 mol %), the title compound was obtained as a brick red powder after purification by column chromatography on silica gel using a hexane/ethyl acetate eluent (7:1 to 5:1 to 3:1): yield 87% (48.7 mg); dr >20:1; mp 244.3–245.3 °C; ^1^H NMR (400 MHz, CDCl_3_) δ 7.65–7.62 (m, 3H), 7.59–7.56 (m, 1H), 7.53–7.47 (m, 2H), 7.44–7.36 (m, 3H), 7.29–7.23 (m, 5H), 7.00 (d, *J* = 8.6 Hz, 1H), 6.33 (dd, *J* = 8.7, 2.4 Hz, 1H), 6.25 (d, *J* = 2.4 Hz, 1H), 4.98–4.89 (m, 2H), 3.60 (s, 3H), 3.57 (d, *J* = 20.3 Hz, 1H), 3.34 (d, *J* = 20.1 Hz, 1H); ^13^C­{^1^H} NMR (101 MHz, CDCl_3_) δ 186.3, 173.8, 168.6, 161.8, 147.9, 145.2, 140.7, 140.5, 136.8, 135.8, 134.8, 134.2, 131.5, 131.2, 129.0, 128.8, 128.6, 128.1, 127.6, 126.7, 125.5, 124.7, 115.7, 111.1, 107.1, 98.4, 66.1, 61.1, 55.5, 50.3, 44.4, 36.3; HRMS (ESI) *m*/*z* calcd for C_36_H_24_O_4_N_3_ [M – H]^−^ 562.1761, found 562.1761.

### 3′-((λ^2^-Azaneylidene)-λ^3^-methyl)-1-benzyl-7-fluoro-5′-phenyl-3′,4′-dihydro-1′*H*-spiro­[indoline-3,11′-[3,10*b*]­methanoindeno­[1,2-*b*]­azepine]-2,2′,6′-trione (**3j**)

Following the general procedure with **1j** (30.3 mg, 0.10 mmol), **2a** (37.2 mg, 0.15 mmol), and quinine (6.8 mg, 20 mol %), the title compound was obtained as a brick red powder after purification by column chromatography on silica gel using a hexane/ethyl acetate eluent (7:1 to 5:1 to 3:1): yield 54% (29.8 mg); dr 17:1; mp 199.0–200.0 °C; ^1^H NMR (400 MHz, CDCl_3_) δ 7.64–7.61 (m, 3H), 7.54–7.47 (m, 4H), 7.43–7.38 (m, 3H), 7.35–7.25 (m, 5H), 6.99–6.91 (m, 2H), 6.86–6.82 (m, 1H), 5.19 (d, *J* = 15.1 Hz, 1H), 5.07 (d, *J* = 15.1 Hz, 1H), 3.57 (d, *J* = 20.2 Hz, 1H), 3.37 (d, *J* = 20.2 Hz, 1H); ^13^C­{^1^H} NMR (101 MHz, CDCl_3_) δ 186.0, 173.1, 168.32, 168.28, 147.9, 147.7 (d, *J* = 245.8 Hz), 140.3, 140.1, 136.5, 135.9, 134.0, 131.6, 131.4, 128.80, 128.77, 128.7, 128.10, 128.06, 125.4, 125.3, 124.7, 124.2 (d, *J* = 6.8 Hz), 122.5, 121.7 (d, *J* = 2.8 Hz), 119.4 (d, *J* = 19.6 Hz), 115.4, 66.3, 61.1, 50.3, 46.2, 36.1; ^19^F­{^1^H} NMR (376 MHz, CDCl_3_) δ −130.8; HRMS (ESI) *m*/*z* calcd for C_35_H_21_O_3_N_3_F [M – H]^−^ 550.1561, found 550.1574.

### 3′-((λ^2^-Azaneylidene)-λ^3^-methyl)-1-benzyl-5′-(4-fluorophenyl)-3′,4′-dihydro-1′*H*-spiro­[indoline-3,11′-[3,10*b*]­methanoindeno­[1,2-*b*]­azepine]-2,2′,6′-trione (**3k**)

Following the general procedure with **1a** (28.5 mg, 0.10 mmol), **2b** (39.9 mg, 0.15 mmol), and quinine (6.8 mg, 20 mol %), the title compound was obtained as a brick red powder after purification by column chromatography on silica gel using a hexane/ethyl acetate eluent (7:1 to 5:1 to 3:1): yield 87% (48.1 mg); dr >20:1; mp 175.0–176.0 °C; ^1^H NMR (400 MHz, CDCl_3_) δ 7.66–7.63 (m, 3H), 7.59 (dd, 1H), 7.56–7.49 (m, 4H), 7.44–7.37 (m, 2H), 7.32–7.23 (m, 4H), 7.17–7.10 (m, 2H), 6.87 (t, *J* = 7.7 Hz, 1H), 6.71 (d, *J* = 7.8 Hz, 1H), 5.03–4.95 (m, 2H), 3.60 (d, *J* = 20.1 Hz, 1H), 3.38 (d, *J* = 20.2 Hz, 1H); ^13^C­{^1^H} NMR (101 MHz, CDCl_3_) δ 186.2, 173.3, 168.4, 164.5 (d, *J* = 254.5 Hz), 146.8, 143.9, 140.6, 140.4, 136.8, 135.9, 134.7, 131.7, 131.14, 131.07 (d, *J* = 30.3 Hz), 130.1 (d, *J* = 30.3 Hz), 129.1, 129.0, 128.1, 127.8, 127.7, 125.6, 125.5, 125.4, 124.8, 123.6, 119.5, 115.9 (d, *J* = 22.2 Hz), 115.5, 110.4, 66.2, 61.3, 50.3, 44.5, 36.3; ^19^F­{^1^H} NMR (376 MHz, CDCl_3_) δ −107.7; HRMS (ESI) calcd for C_35_H_22_N_3_O_3_FNa [M + Na]^+^ 574.1537, found 574.1534.

### 3′-((λ^2^-Azaneylidene)-λ^3^-methyl)-1-benzyl-5′-(4-chlorophenyl)-3′,4′-dihydro-1′*H*-spiro­[indoline-3,11′-[3,10*b*]­methanoindeno­[1,2-*b*]­azepine]-2,2′,6′-trione (**3l**)

Following the general procedure with **1a** (28.5 mg, 0.10 mmol), **2c** (42.3 mg, 0.15 mmol), and quinine (6.8 mg, 20 mol %), the title compound was obtained as a brick red powder after purification by column chromatography on silica gel using a hexane/ethyl acetate eluent (7:1 to 5:1 to 3:1): yield 77% (43.9 mg); dr >20:1; mp 223.1–224.1 °C; ^1^H NMR (400 MHz, CDCl_3_) δ 7.66–7.56 (m, 5H), 7.49–7.44 (m, 2H), 7.43–7.37 (m, 2H), 7.33–7.23 (m, 5H), 7.16 (t, *J* = 7.8 Hz, 1H), 7.04 (d, *J* = 7.8 Hz, 1H), 6.86 (t, *J* = 7.7 Hz, 1H), 6.71 (d, *J* = 8.0 Hz, 1H), 5.02–4.93 (m, 2H), 3.56 (d, *J* = 20.1 Hz, 1H), 3.35 (d, *J* = 20.1 Hz, 1H); ^13^C­{^1^H} NMR (101 MHz, CDCl_3_) δ 186.2, 173.2, 168.6, 146.5, 143.8, 140.5, 140.3, 137.4, 137.1, 136.0, 134.6, 132.4, 131.6, 131.1, 130.3, 129.0, 128.9, 128.1, 127.6, 125.6, 125.5, 124.8, 123.6, 119.4, 115.5, 110.4, 66.1, 61.2, 50.2, 44.4, 35.9; HRMS (ESI) calcd for C_35_H_22_N_3_O_3_ClNa [M + Na]^+^ 590.1242, found 590.1245.

### 3′-((λ^2^-Azaneylidene)-λ^3^-methyl)-1-benzyl-5′-(4-bromophenyl)-3′,4′-dihydro-1′*H*-spiro­[indoline-3,11′-[3,10*b*]­methanoindeno­[1,2-*b*]­azepine]-2,2′,6′-trione (**3m**)

Following the general procedure with **1a** (28.5 mg, 0.10 mmol), **2d** (49.1 mg, 0.15 mmol), and quinine (6.8 mg, 20 mol %), the title compound was obtained as a brick red powder after purification by column chromatography on silica gel using a hexane/ethyl acetate eluent (7:1 to 5:1 to 3:1): yield 91% (55.7 mg); dr >20:1; mp 181.7–182.7 °C; ^1^H NMR (400 MHz, CDCl_3_) δ 7.68–7.65 (m, 3H), 7.59–7.53 (m, 3H), 7.47–7.38 (m, 3H), 7.34–7.24 (m, 5H), 7.18 (t, *J* = 7.7 Hz, 1H), 7.04 (d, *J* = 7.7 Hz, 1H), 6.88 (t, *J* = 7.7 Hz, 1H), 6.73 (d, *J* = 7.9 Hz, 1H), 5.04–4.95 (m, 2H), 3.58 (d, *J* = 20.2 Hz, 1H), 3.37 (d, *J* = 20.1 Hz, 1H); ^13^C­{^1^H} NMR (101 MHz, CDCl_3_) δ 186.2, 173.2, 168.5, 146.6, 143.9, 140.5, 140.3, 137.1, 136.0, 134.7, 132.9, 131.9, 131.7, 131.1, 130.4, 129.0, 128.1, 127.6, 125.9, 125.6, 125.4, 124.8, 123.6, 119.4, 115.5, 110.5, 66.1, 61.1, 50.2, 44.4, 35.9; HRMS (ESI) calcd for C_35_H_22_N_3_O_3_BrNa [M + Na]^+^ 634.0737, found 634.0743.

### 3′-((λ^2^-Azaneylidene)-λ^3^-methyl)-1-benzyl-5′-(4-methylphenyl)-3′,4′-dihydro-1′*H*-spiro­[indoline-3,11′-[3,10*b*]­methanoindeno­[1,2-*b*]­azepine]-2,2′,6′-trione (**3n**)

Following the general procedure with **1a** (28.5 mg, 0.10 mmol), **2e** (39.3 mg, 0.15 mmol), and quinine (6.8 mg, 20 mol %), the title compound was obtained as a brick red powder after purification by column chromatography on silica gel using a hexane/ethyl acetate eluent (7:1 to 5:1 to 3:1): yield 88% (47.9 mg); dr >20:1; mp 185.8–186.8 °C; ^1^H NMR (400 MHz, CDCl_3_) δ 7.64–7.61 (m, 1H), 7.57–7.53 (m, 3H), 7.42–7.34 (m, 3H), 7.33–7.22 (m, 7H), 7.12 (t, *J* = 7.7 Hz, 1H), 7.07 (d, *J* = 7.7 Hz, 1H), 6.82 (t, *J* = 7.7 Hz, 1H), 6.68 (d, *J* = 7.8 Hz, 1H), 5.02–4.93 (m, 2H), 3.58 (d, *J* = 20.1 Hz, 1H), 3.34 (d, *J* = 20.1 Hz, 1H), 2.46 (s, 3H); ^13^C­{^1^H} NMR (101 MHz, CDCl_3_) δ 186.2, 173.3, 168.6, 148.2, 143.8, 142.0, 140.5, 136.1, 135.7, 134.8, 131.5, 131.3, 130.9, 129.3, 129.0, 128.8, 128.1, 127.6, 125.8, 125.4, 124.7, 123.6, 119.6, 115.7, 110.3, 66.2, 61.3, 50.2, 44.4, 36.1, 21.7; HRMS (ESI) calcd for C_36_H_25_N_3_O_3_Na [M + Na]^+^ 570.1788, found 570.1790.

### 3′-((λ^2^-Azaneylidene)-λ^3^-methyl)-1-benzyl-5′-(4-methoxyphenyl)-3′,4′-dihydro-1′*H*-spiro­[indoline-3,11′-[3,10*b*]­methanoindeno­[1,2-*b*]­azepine]-2,2′,6′-trione (**3o**)

Following the general procedure with **1a** (28.5 mg, 0.10 mmol), **2f** (41.7 mg, 0.15 mmol), and quinine (6.8 mg, 20 mol %), the title compound was obtained as a yellow powder after purification by column chromatography on silica gel using a hexane/ethyl acetate eluent (7:1 to 5:1 to 3:1): yield 68% (38.2 mg); dr >20:1; mp 233.5–234.5 °C; ^1^H NMR (400 MHz, CDCl_3_) δ 7.68–7.64 (m, 3H), 7.59 (d, *J* = 8.0 Hz, 1H), 7.45–7.35 (m, 3H), 7.30–7.23 (m, 5H), 7.14 (td, *J* = 7.8, 1.2 Hz, 1H), 7.06–7.02 (m, 3H), 6.84 (td, *J* = 7.8, 1.1 Hz, 1H), 6.69 (dd, *J* = 8.0, 1.1 Hz, 1H), 5.04–4.95 (m, 2H), 3.90 (s, 3H), 3.64 (d, *J* = 19.9 Hz, 1H), 3.35 (d, *J* = 19.9 Hz, 1H); ^13^C­{^1^H} NMR (101 MHz, CDCl_3_) δ 186.2, 173.3, 168.6, 162.4, 148.1, 143.8, 140.6, 140.5, 135.6, 135.2, 134.8, 131.5, 130.92, 130.85, 129.0, 128.1, 127.6, 126.3, 125.8, 125.3, 124.7, 123.6, 119.6, 115.7, 114.0, 110.3, 61.4, 55.6, 50.2, 44.4, 36.0; HRMS (ESI) calcd for C_36_H_25_N_3_O_4_Na [M + Na]^+^ 586.1738, found 586.1741.

### 3′-((λ^2^-Azaneylidene)-λ^3^-methyl)-1-benzyl-5′-(naphthalen-2-yl)-3′,4′-dihydro-1′*H*-spiro­[indoline-3,11′-[3,10*b*]­methanoindeno­[1,2-*b*]­azepine]-2,2′,6′-trione (**3p**)

Following the general procedure with **1a** (28.5 mg, 0.10 mmol), **2g** (44.7 mg, 0.15 mmol), and quinine (6.8 mg, 20 mol %), the title compound was obtained as a brick red powder after purification by column chromatography on silica gel using a hexane/ethyl acetate eluent (7:1 to 5:1 to 3:1): yield 82% (47.7 mg); dr >20:1; mp 220.4–221.4 °C; ^1^H NMR (400 MHz, CDCl_3_) δ 8.11 (d, *J* = 1.8 Hz, 1H), 7.93–7.86 (m, 3H), 7.74 (dd, *J* = 8.5, 1.9 Hz, 1H), 7.65–7.62 (m, 1H), 7.59–7.51 (m, 3H), 7.46–7.45 (m, 1H), 7.42–7.35 (m, 2H), 7.30–7.21 (m, 5H), 7.17–7.11 (m, 2H), 6.89 (td, *J* = 7.8, 1.1 Hz, 1H), 6.72 (d, *J* = 7.8 Hz, 1H), 5.01–4.91 (m, 2H), 3.75 (d, *J* = 20.1 Hz, 1H), 3.45 (d, *J* = 20.1 Hz, 1H); ^13^C­{^1^H} NMR (101 MHz, CDCl_3_) δ 186.2, 173.3, 168.7, 148.0, 143.9, 140.6, 140.5, 136.9, 135.8, 134.7, 134.7, 132.9, 131.8, 131.6, 131.0, 129.1, 129.0, 128.5, 128.09, 128.07, 127.94, 127.89, 127.6, 126.9, 126.5, 125.8, 125.5, 124.8, 123.7, 119.6, 115.7, 110.4, 66.3, 61.3, 50.3, 44.4, 36.3; HRMS (ESI) calcd for C_39_H_25_N_3_O_3_Na [M + Na]^+^ 606.1788, found 606.1783.

### 3′-((λ^2^-Azaneylidene)-λ^3^-methyl)-1-benzyl-5′-(thiophen-2-yl)-3′,4′-dihydro-1′*H*-spiro­[indoline-3,11′-[3,10*b*]­methanoindeno­[1,2-*b*]­azepine]-2,2′,6′-trione (**3q**)

Following the general procedure with **1a** (28.5 mg, 0.10 mmol), **2h** (38.1 mg, 0.15 mmol), and quinine (6.8 mg, 20 mol %), the title compound was obtained as a brick red powder after purification by column chromatography on silica gel using a hexane/ethyl acetate eluent (7:1 to 5:1 to 3:1): yield 74% (39.7 mg); dr >20:1; mp 257.9–258.9 °C; ^1^H NMR (400 MHz, acetone-*d*_6_) δ 8.51 (s, 1H), 8.35 (dd, *J* = 3.9, 1.2 Hz, 1H), 7.90 (dd, *J* = 5.0, 1.1 Hz, 1H), 7.65 (dd, *J* = 6.5, 1.7 Hz, 1H), 7.61–7.50 (m, 3H), 7.42–7.39 (m, 2H), 7.34–7.28 (m, 4H), 7.17 (td, *J* = 7.8, 1.3 Hz, 1H), 7.01 (d, *J* = 7.8 Hz, 1H), 6.89 (d, *J* = 7.9 Hz, 1H), 6.84 (td, *J* = 7.7, 1.1 Hz, 1H), 5.21 (d, *J* = 15.6 Hz, 1H), 4.98 (d, *J* = 15.6 Hz, 1H), 3.92 (d, *J* = 19.7 Hz, 1H), 3.74 (d, *J* = 19.7 Hz, 1H); ^13^C­{^1^H} NMR (101 MHz, acetone-*d*_6_) δ 186.2, 173.2, 168.2, 144.0, 141.2, 140.3, 139.3, 136.9, 135.6, 135.5, 134.2, 133.2, 132.1, 131.4, 130.7, 128.8, 127.82, 127.80, 127.7, 125.9, 125.3, 123.8, 123.1, 120.0, 116.2, 110.2, 66.7, 61.0, 50.1, 43.7, 36.0; HRMS (ESI) *m*/*z* calcd for C_33_H_20_O_3_N_3_S [M – H]^−^ 538.1220, found 538.1238.

### 3′-((λ^2^-Azaneylidene)-λ^3^-methyl)-1-(4-methoxybenzyl)-5′-phenyl-3′,4′-dihydro-1′*H*-spiro­[indoline-3,11′-[3,10*b*]­methanoindeno­[1,2-*b*]­azepine]-2,2′,6′-trione (**3r**)

Following the general procedure with **1k** (31.5 mg, 0.10 mmol), **2a** (37.2 mg, 0.15 mmol), and quinine (6.8 mg, 20 mol %), the title compound was obtained as a brick red powder after purification by column chromatography on silica gel using a hexane/ethyl acetate eluent (7:1 to 5:1 to 3:1): yield 84% (47.3 mg); dr >20:1; mp 194.5–195.5 °C; ^1^H NMR (400 MHz, CDCl_3_) δ 7.66–7.62 (m, 3H), 7.59–7.56 (m, 1H), 7.55–7.49 (m, 3H), 7.45–7.389 (m, 2H), 7.25–7.19 (m, 3H), 7.16 (td, *J* = 7.7, 1.2 Hz, 1H), 7.10 (dd, *J* = 7.8, 1.2 Hz, 1H), 6.88–6.80 (m, 3H), 6.73 (dd, *J* = 7.8, 1.1 Hz, 1H), 4.97 (d, *J* = 15.4 Hz, 1H), 4.89 (d, *J* = 15.3 Hz, 1H), 3.77 (s, 3H), 3.59 (d, *J* = 20.3 Hz, 1H), 3.38 (d, *J* = 20.1 Hz, 1H); ^13^C­{^1^H} NMR (101 MHz, CDCl_3_) δ 186.2, 173.2, 168.5, 159.4, 148.0, 143.9, 140.6, 140.4, 136.7, 135.8, 134.2, 131.6, 131.3, 130.9, 129.1, 128.8, 128.7, 126.8, 125.7, 125.4, 124.7, 123.5, 119.6, 115.6, 114.3, 110.3, 66.1, 61.1, 55.4, 50.2, 43.9, 36.3; HRMS (ESI) *m*/*z* calcd for C_36_H_24_O_4_N_3_ [M – H]^−^ 562.1761, found 562.1762.

### 3′-((λ^2^-Azaneylidene)-λ^3^-methyl)-1-(4-bromobenzyl)-5′-phenyl-3′,4′-dihydro-1′*H*-spiro­[indoline-3,11′-[3,10*b*]­methanoindeno­[1,2-*b*]­azepine]-2,2′,6′-trione (**3s**)

Following the general procedure with **1l** (36.4 mg, 0.10 mmol), **2a** (37.2 mg, 0.15 mmol), and quinine (6.8 mg, 20 mol %), the title compound was obtained as a brick red powder after purification by column chromatography on silica gel using a hexane/ethyl acetate eluent (7:1 to 5:1 to 3:1): yield 36% (22.2 mg); dr >20:1; mp 245.0–246.0 °C; ^1^H NMR (400 MHz, CDCl_3_) δ 7.68–7.62 (m, 3H), 7.56–7.41 (m, 8H), 7.20–7.11 (m, 4H), 7.03 (bs, 1H), 6.89 (t, *J* = 7.5 Hz, 1H), 6.68 (d, *J* = 7.9 Hz, 1H), 5.00 (d, *J* = 15.7 Hz, 1H), 4.91 (d, *J* = 15.8 Hz, 1H), 3.60 (d, *J* = 20.1 Hz, 1H), 3.39 (d, *J* = 20.2 Hz, 1H); ^13^C­{^1^H} NMR (101 MHz, CDCl_3_) δ 186.0, 173.3, 168.3, 148.1, 143.5, 140.5, 136.5, 135.8, 134.1, 133.8, 132.1, 131.7, 131.4, 131.1, 129.4, 128.7, 128.7, 125.9, 125.2, 124.9, 123.8, 122.1, 119.5, 115.5, 110.2, 66.1, 61.1, 50.2, 43.8, 36.3; HRMS (ESI) *m*/*z* calcd for C_35_H_21_O_3_N_3_Br [M – H]^−^ 610.0761, found 610.0766.

### 3′-((λ^2^-Azaneylidene)-λ^3^-methyl)-1-methyl-5′-phenyl-3′,4′-dihydro-1′*H*-spiro­[indoline-3,11′-[3,10*b*]­methanoindeno­[1,2-*b*]­azepine]-2,2′,6′-trione (**3t**)

Following the general procedure with **1m** (20.9 mg, 0.10 mmol), **2a** (37.2 mg, 0.15 mmol), and quinine (6.8 mg, 20 mol %), the title compound was obtained as a red powder after purification by column chromatography on silica gel using a hexane/ethyl acetate eluent (7:1 to 5:1 to 3:1): yield 98% (44.8 mg); dr >20:1; mp 177.7–178.7 °C; ^1^H NMR (400 MHz, CDCl_3_) δ 7.76–7.65 (m, 5H), 7.61–7.50 (m, 4H), 7.32–7.26 (m, 2H), 7.11 (d, *J* = 7.6 Hz, 1H), 6.91 (t, *J* = 7.7 Hz, 1H), 6.79 (d, *J* = 7.7 Hz, 1H), 3.59 (d, *J* = 20.3 Hz, 1H), 3.43–3.33 (m, 4H); ^13^C­{^1^H} NMR (101 MHz, CDCl_3_) δ 186.2, 173.1, 168.5, 148.0, 144.6, 140.7, 140.5, 136.6, 135.9, 134.2, 131.6, 131.3, 131.0, 128.8, 128.7, 125.6, 125.0, 124.8, 123.6, 119.5, 115.5, 109.4, 66.1, 61.3, 50.0, 36.3, 26.9; HRMS (ESI) calcd for C_29_H_19_N_3_O_3_Na [M + Na]^+^ 480.1319, found 480.1310.

### 3′-((λ^2^-Azaneylidene)-λ^3^-methyl)-1-ethyl-5′-phenyl-3′,4′-dihydro-1′*H*-spiro­[indoline-3,11′-[3,10*b*]­methanoindeno­[1,2-*b*]­azepine]-2,2′,6′-trione (**3u**)

Following the general procedure with **1n** (22.3 mg, 0.10 mmol), **2a** (37.2 mg, 0.15 mmol), and quinine (6.8 mg, 20 mol %), the title compound was obtained as a brick red powder after purification by column chromatography on silica gel using a hexane/ethyl acetate eluent (7:1 to 5:1 to 3:1): yield 88% (41.3 mg); dr >20:1; mp 218.7–219.7 °C; ^1^H NMR (400 MHz, CDCl_3_) δ 7.74 (dd, *J* = 7.7, 2.9 Hz, 1H), 7.67–7.63 (m, 3H), 7.61–7.56 (m, 1H), 7.54–7.49 (m, 3H), 7.47–7.37 (m, 2H), 7.24 (t, *J* = 7.9 Hz, 1H), 7.10 (d, *J* = 7.7 Hz, 1H), 6.88 (t, *J* = 7.7 Hz, 1H), 6.79 (d, *J* = 7.8 Hz, 1H), 3.83 (q, *J* = 7.0 Hz, 2H), 3.56 (d, *J* = 20.1 Hz, 1H), 3.34 (d, *J* = 20.2 Hz, 1H), 1.26 (t, *J* = 7.0 Hz, 3H); ^13^C­{^1^H} NMR (101 MHz, CDCl_3_) δ 186.3, 172.7, 168.6, 147.9, 143.6, 140.8, 140.5, 136.7, 135.8, 134.2, 131.6, 131.2, 131.0, 128.8, 128.6, 125.8, 125.3, 124.7, 123.3, 119.8, 115.4, 109.5, 66.0, 60.9, 50.1, 36.2, 35.4, 12.5; HRMS (ESI) *m*/*z* calcd for C_30_H_20_O_3_N_3_ [M – H]^−^ 470.1499, found 470.1502.

### 3′-((λ^2^-Azaneylidene)-λ^3^-methyl)-1-allyl-5′-phenyl-3′,4′-dihydro-1′*H*-spiro­[indoline-3,11′-[3,10*b*]­methanoindeno­[1,2-*b*]­azepine]-2,2′,6′-trione (**3v**)

Following the general procedure with **1o** (23.5 mg, 0.10 mmol), **2a** (37.2 mg, 0.15 mmol), and quinine (6.8 mg, 20 mol %), the title compound was obtained as a brick red powder after purification by column chromatography on silica gel using a hexane/ethyl acetate eluent (7:1 to 5:1 to 3:1): yield 77% (37.1 mg); dr >20:1; mp 187.3–188.3 °C; ^1^H NMR (400 MHz, CDCl_3_) δ 7.74–7.46 (m, 9H), 7.22 (dd, *J* = 7.8, 1.2 Hz, 1H), 7.11 (dd, *J* = 7.9, 1.2 Hz, 1H), 6.89 (td, *J* = 7.7, 1.1 Hz, 1H), 6.84 (d, *J* = 5.3 Hz, 1H), 6.77 (d, *J* = 7.9 Hz, 1H), 5.83–5.76 (m, 1H), 5.23–5.13 (m, 2H), 4.57–4.51 (m, 1H), 4.40–4.35 (m, 1H), 3.61 (d, *J* = 20.2 Hz, 1H), 3.38 (d, *J* = 20.2 Hz, 1H); ^13^C­{^1^H} NMR (101 MHz, CDCl_3_) δ 186.1, 172.8, 168.2, 148.1, 143.9, 140.6, 140.5, 136.6, 135.8, 134.1, 131.7, 131.3, 131.0, 130.4, 128.7, 128.7, 125.7, 125.4, 124.8, 123.5, 119.4, 118.5, 115.4, 110.3, 66.0, 61.1, 50.1, 42.8, 36.4; HRMS (ESI) *m*/*z* calcd for C_31_H_20_O_3_N_3_ [M – H]^−^ 482.1499, found 482.1502.

### *tert*-Butyl 3′-((λ^2^-Azaneylidene)-λ^3^-methyl)-1-benzyl-6-bromo-6′-((*tert*-butoxycarbonyl)­oxy)-2,2′-dioxo-5′-phenyl-2′,3′-dihydro-1′*H*-spiro­[indoline-3,11′-[3,10*b*]­methanoindeno­[1,2-*b*]­azepine]-1′-carboxylate (**4**)

In a 7 mL glass vial, compound **3h** (61.2 mg, 0.1 mmol) was dissolved in dichloromethane (1.0 mL) with triethylamine (14 μL, 0.1 mmol) and DMAP (1.2 mg, 0.01 mmol). Consequently, Boc_2_O (65.5 mg, 0.3 mmol) was added to the mixture, and it was stirred at room temperature overnight. After completion of the reaction (determined by TLC), the reaction mixture was extracted with saturated NH_4_Cl_(aq)_ (2 × 10 mL). Then, the combined organic layer was washed with brine (2 × 20 mL) and dried over anhydrous Na_2_SO_4_, and the crude product was purified by silica gel column chromatography using a 5:1 hexane/ethyl acetate eluent to obtain product **4** (61.3 mg, 75% yield) as a yellow powder: dr >20:1; mp 203.0–204.0 °C; ^1^H NMR (400 MHz, CDCl_3_) δ 7.46–7.41 (m, 5H), 7.33–7.28 (m, 5H), 7.21 (dd, *J* = 6.7, 2.8 Hz, 2H), 7.18 (d, *J* = 8.3 Hz, 1H), 7.15 (d, *J* = 7.6 Hz, 1H), 7.08 (td, *J* = 7.6, 1.1 Hz, 1H), 7.00 (dd, *J* = 8.3, 1.8 Hz, 1H), 6.72 (d, *J* = 1.8 Hz, 1H), 6.15 (s, 1H), 4.99 (d, *J* = 15.7 Hz, 1H), 4.91 (d, *J* = 15.7 Hz, 1H), 1.28 (s, 9H), 1.08 (s, 9H); ^13^C­{^1^H} NMR (101 MHz, CDCl_3_) δ 172.6, 163.6, 148.7, 148.1, 147.2, 144.6, 139.2, 139.0, 138.4, 135.8, 134.3, 130.0, 129.5, 129.0, 128.7, 128.5, 128.2, 127.6, 127.5, 126.3, 124.9, 123.1, 122.1, 120.9, 119.7, 117.6, 114.4, 112.7, 84.8, 84.1, 72.5, 60.8, 54.7, 44.4, 27.5, 27.4; HRMS (ESI) *m*/*z* calcd for C_45_H_37_ON_3_Br [M – H]^−^ 810.1809, found 810.1827.

## Supplementary Material



## Data Availability

The data underlying this study are available in the published article and its Supporting Information.

## Notes

General information, X-ray analysis data, and NMR spectra of all compounds (PDF)
