# Supplementary material for: Organocatalytic Synthesis of Spiro-Bridged Heterocyclic Compounds via a Chemoselective Vinylogous Michael/Cyclization/Rearrangement Sequence
Source: J Org Chem. 2025 May 16;90(21):7125–33. doi: 10.1021/acs.joc.5c00443 (PMC12131217; doi:10.1021/acs.joc.5c00443)
Supplement: Supplementary file 1 [file jo5c00443_si_001.pdf]

Supporting Information

Organocatalytic Synthesis of Spiro-Bridged Heterocyclic Compounds via a Chemoselective Vinylogous Michael/Cyclization/Rearrangement Sequence

I-Ting Chen, Hsuan Lin and Jeng-Liang Han*

Department of Chemistry, National Chung Hsing University, 145 Xingda Rd., South Dist., Taichung 40227, Taiwan

E-mail: jlhan@nchu.edu.tw

Table of Contents:

1.	General Experimental Details	S2
2.	Preparation of substrates	S3
3.	Computational Methods	S4
4.	References	S4
5.	Typical procedure of the synthesis of compounds **3**	S5
6.	Synthesis of 1.0 mmol scale of **3h**	S5
7.	X-Ray Analysis Data	S6
8.	Asymmetric catalysis	S25
9.	HPLC Spectra of **3a**	S26
10.	Further substrate scope	S27
11.	^{1}H NMR and ^{13}C NMR spectra of new compounds	S28
12.	HRMS spectra of new compounds	S77
13.	Cartesian Coordinates	S100

1. General Experimental Details

All commercially available reagents were used without further purification unless otherwise stated. All reaction solvents were purified before use. Proton nuclear magnetic resonance (^{1}H NMR) spectra were recorded on a commercial instrument at 400 MHz. Carbon-13 nuclear magnetic resonance (^{13}C{^{1}H} NMR) spectra were recorded at 100 MHz. The proton signal for residual non-deuterated solvent (δ 7.26 for $CHCl_3$) was used as an internal reference for ^{1}H NMR spectra. For ^{13}C{^{1}H} NMR spectra, chemical shifts are reported relative to the δ 77.0 resonance of $CHCl_3$. Coupling constants are reported in Hz. Melting points were determined on a BUCHI B-545 melting point apparatus and are uncorrected. High resolution mass spectra were recorded on a Thermo Fisher Scientific LTQ Orbitrap XL mass spectrometer. The single crystal was measured by Bruker D8 VENTURE X-ray Single Crystal Diffractometer. Analytical thin-layer chromatography (TLC) was performed on silica gel 60 F254 pre-coated plates with visualization under UV light. Column chromatography was generally performed using 40-63 μm (230-400 mesh) silica gel, typically using a 50-100:1 weight ratio of silica gel to crude product.

2. Preparation of substrates

2.1 Preparation of isatylidene-malononitriles 1

Isatylidene-malononitriles **1** were prepared according to known procedures. [1]

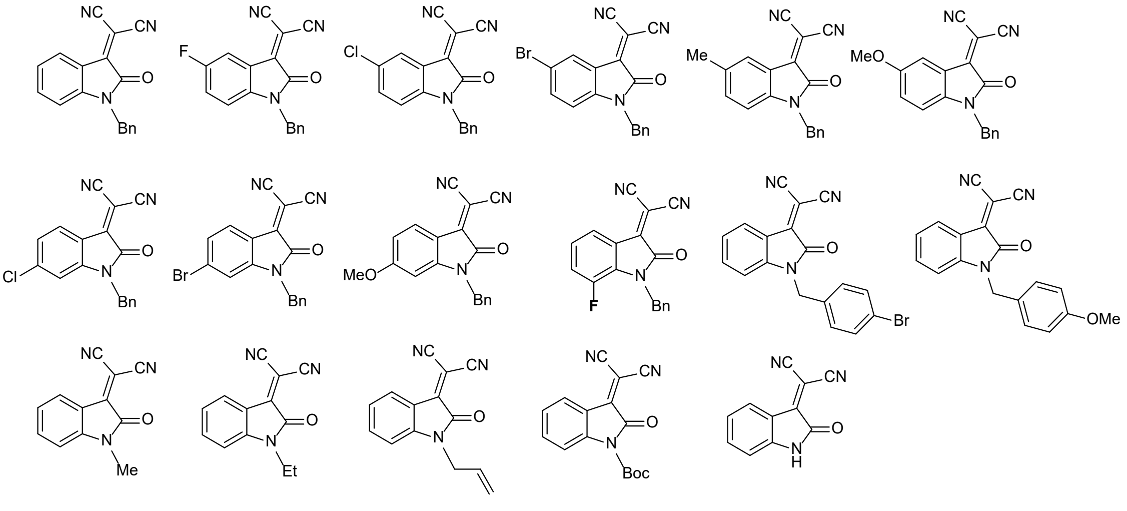

2.2 Preparation of spiroindane-1,3-diones 2

Spiroindane-1,3-diones **2** were prepared according to known procedures. [2]

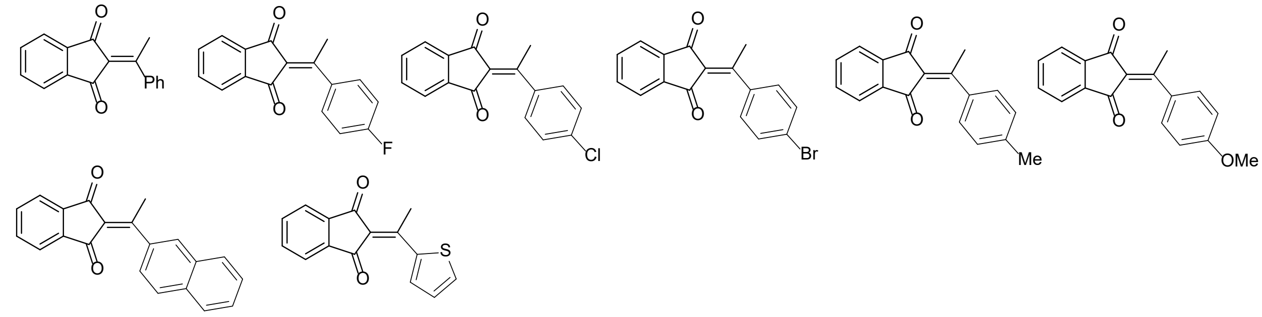

3. Computational Methods

Quantum mechanical calculations were performed using Gaussian 16 (Revision 1.1).[3] The transition state of geometries were optimized using the Becke's three parameter functional varied by Lee-Yang-Parr correlation functional (B3LYP).[4-6] The split-valence 6-311G(d,p)[7,8] basis set and an ultrafine integration grid within the IEFPCM model. All of transition state of geometries were checked with one imaginary frequency. Single point energies were calculated using M06-2X[9] add the D3[10] version of Grimme's dispersion (M06-2X(D3)), the def2-TZVPP[11] basis set and an ultrafine integration grid within the SMD model. Computed structures are illustrated with CYLView20.[12]

4. References

1. Auria-Luna, F.; Marqués-López, E.; Mohammadi, S.; Heiran, R.; Herrera, R.P. *Molecules* **2015**, *20*, 15807.
2. (a) Möhlmann, L.; Chang, G.; Madhusudhan, G.; Lee, C.; Lin, W. *Org. Lett.* **2016**, *18*, 688−691. (b) Gao, Y.; Liu, D.; Fu, Z.; Huang, W. *Org. Lett.* **2019**, *21*, 926. (c) Kuan, J.-Y.; Chen, I.-T.; Lin, H.; Han, J.-L. *Adv. Synth. Catal.* **2023**, *365*, 3493.
3. Gaussian 16, Revision 1.1, Frisch, M. J.; Trucks, G. W.; Schlegel, H. B.; Scuseria, G. E.; Robb, M. A.; Cheeseman, J. R.; Scalmani, G.; Barone, V.; Petersson, G. A.; Nakatsuji, H.; Li, X.; Caricato, M.; Marenich, A. V.; Bloino, J.; Janesko, B. G.; Gomperts, R.; Mennucci, B.; Hratchian, H. P.; Ortiz, J. V.; Izmaylov, A. F.; Sonnenberg, J. L.; Williams-Young, D.; Ding, F.; Lipparini, F.; Egidi, F.; Goings, J.; Peng, B.; Petrone, A.; Henderson, T.; Ranasinghe, D.; Zakrzewski, V. G.; Gao, J.; Rega, N.; Zheng, G.; Liang, W.; Hada, M.; Ehara, M.; Toyota, K.; Fukuda, R.; Hasegawa, J.; Ishida, M.; Nakajima, T.; Honda, Y.; Kitao, O.; Nakai, H.; Vreven, T.; Throssell, K.; Montgomery, J. A., Jr.; Peralta, J. E.; Ogliaro, F.; Bearpark, M. J.; Heyd, J. J.; Brothers, E. N.; Kudin, K. N.; Staroverov, V. N.; Keith, T. A.; Kobayashi, R.; Normand, J.; Raghavachari, K.; Rendell, A. P.; Burant, J. C.; Iyengar, S. S.; Tomasi, J.; Cossi, M.; Millam, J. M.; Klene, M.; Adamo, C.; Cammi, R.; Ochterski, J. W.; Martin, R. L.; Morokuma, K.; Farkas, O.; Foresman, J. B.; Fox, D. J. Gaussian, Inc., Wallingford CT, 2016.
4. Becke, A. D. *J. Chem. Phys.* **1992**, *96*, 2155.
5. Becke, A. D. *J. Chem. Phys.* **1993**, *98*, 5648.
6. Lee, C.; Yang, W.; Parr, R. G. *J. Chem. Phys.* **1980**, *72*, 5639.
7. Tomasi, J.; Mennucci, B.; Cammi, R. *Chem. Rev.* **2005**, *105*, 2999.
8. Raghavachari, K.; Binkley, J. S.; Seeger, R.; Pople, J. A. *J. Chem. Phys.* **1980**, *72*, 650.
9. Zhao, Y.; Truhlar, D. G. *Theor. Chem. Acc.* **2008**, *120*, 215.
10. Grimme, S.; Ehrlich, S.; Goerigk, L. *J. Comp. Chem.* **2011**, *32*, 1456.
11. Weigend, F.; Ahlrichs, R. *Phys. Chem. Chem. Phys.*, **2005**, *7,* 3297.
12. CYLview20; C. Y. Legault, Université de Sherbrooke, **2020** (http://www.cylview.org).

5.Typical procedure of the synthesis of compounds 3

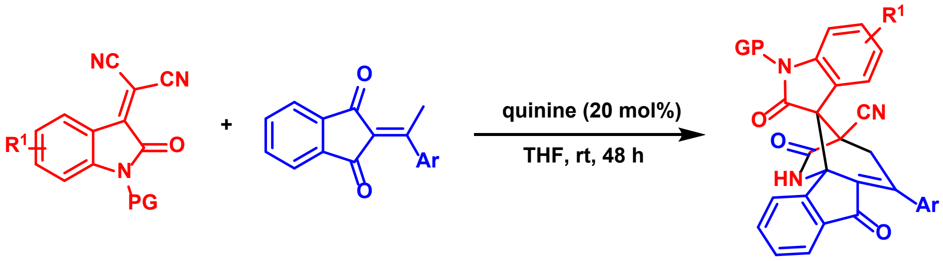

1 2 3

To a 7 mL vial equipped with stirring bar was added **1** (0.10 mmol), **2** (0.15 mmol) and quinie (20 mol%). Then 1.0 mL of dry THF was added to the mixture, the resulted reaction mixture was allowed to stir at room temperature for 48 h. After completion of the reaction as indicated by TLC, the solvent was removed under reduced pressure and the resulted residue was purified by column chromatography on silica gel using petroleum ether-ethyl acetate (7:1 v/v) as eluent to afford **3**.

6. Synthesis of 1.0 mmol scale of 3h

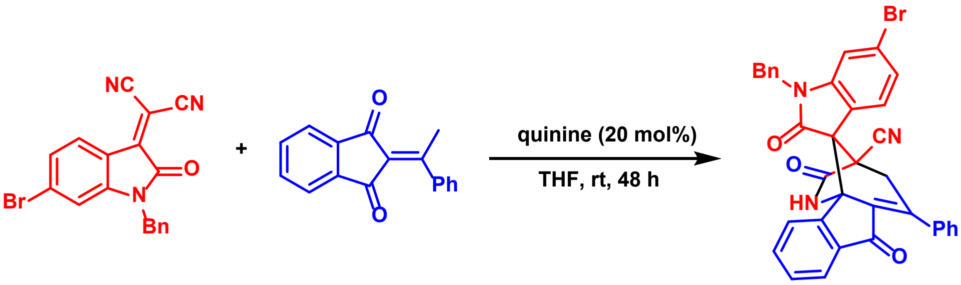

1h 2a 3h

To a 25 mL vial equipped with stirring bar was added **1h** (364 mg, 1.0 mmol), **2** (372 mg, 0.15 mmol) and quinie (65 mg, 0.2 mmol, 20 mol%). Then 10 mL of dry THF was added to the mixture, the resulted reaction mixture was allowed to stir at room temperature for 48 h. After completion of the reaction as indicated by TLC, the solvent was removed under reduced pressure and the resulted residue was purified by column chromatography on silica gel using petroleum ether-ethyl acetate (7:1 v/v) as eluent to afford **3h** (453 mg, 74% yield).

7. X-Ray Analysis Data

CCDC 2370395 (3f)

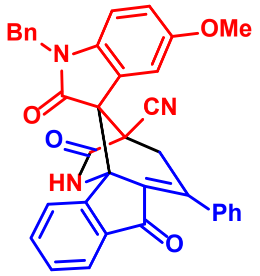

3f

ORTEP drawing of **3f** showing thermal ellipsoids at the 50% probability level

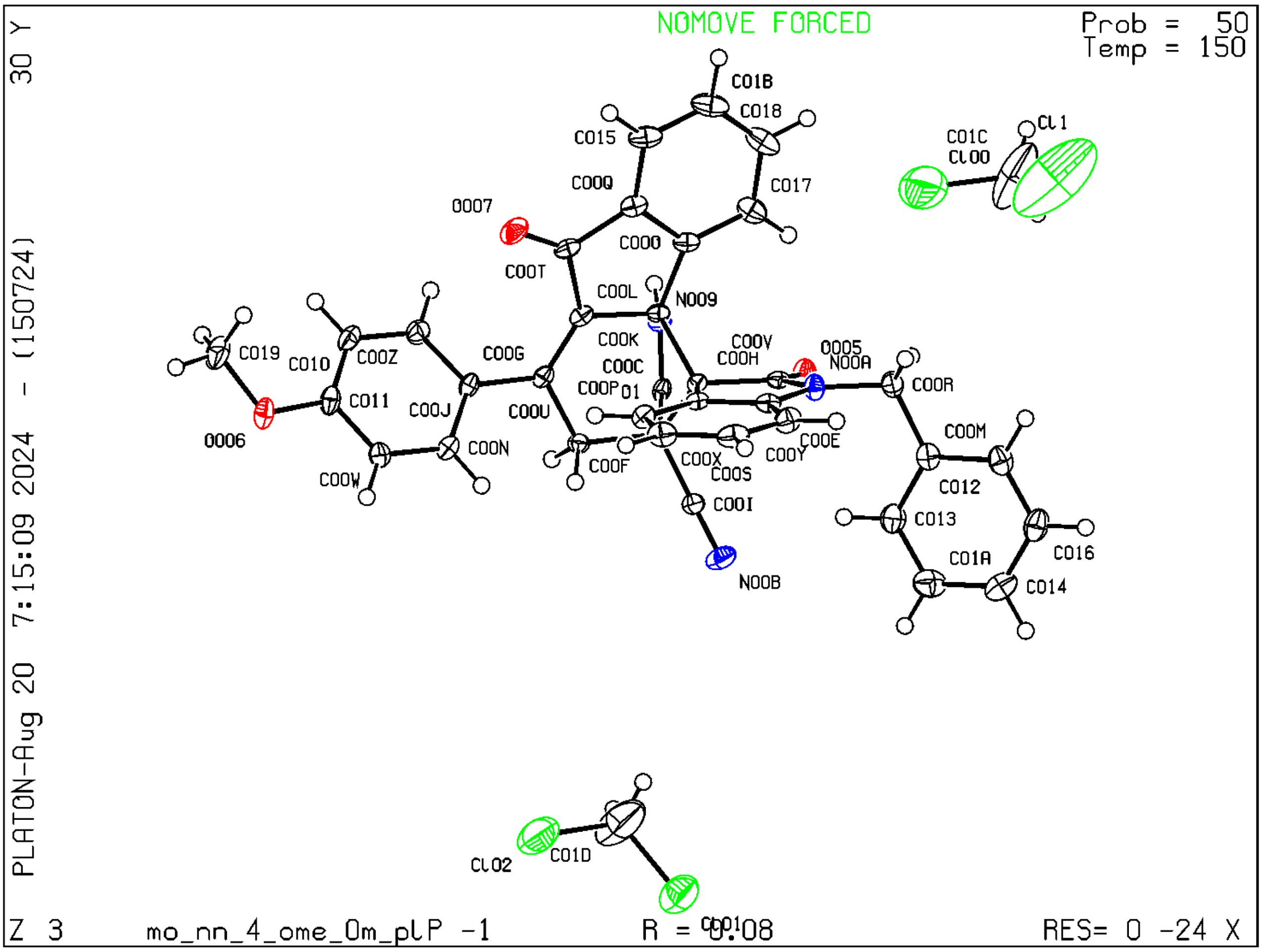

The crystal was obtained by slow evaporation of **3f** in Hexane : DCM = 3:1 at room temperature.

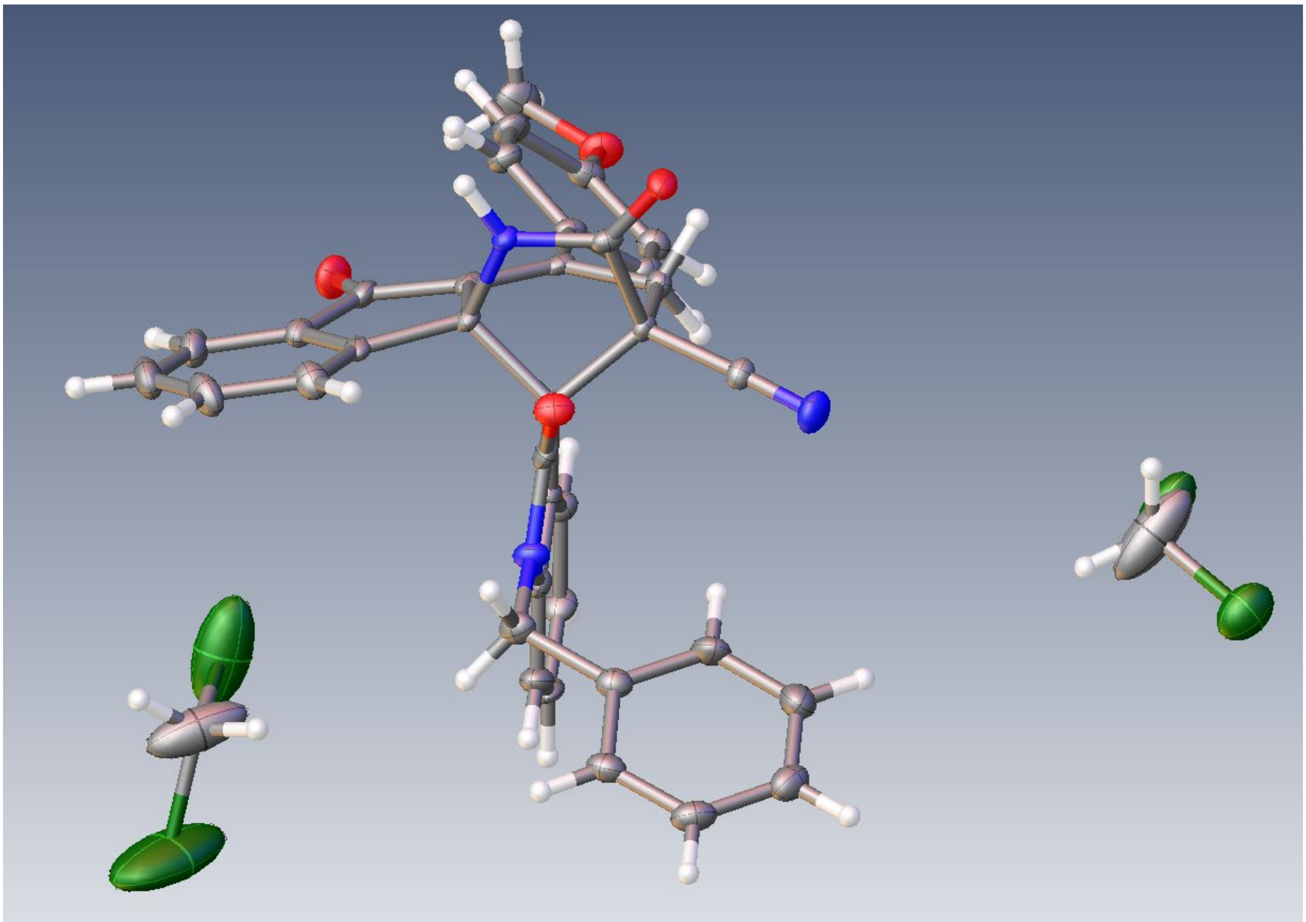

Different angle of view of **3f**

Table S1. Crystal data and structure refinement for **3f**.

Identification code	mo_nn_4_ome_0m_pl	
Empirical formula	C38 H29 Cl4 N3 O4	
Formula weight	733.44	
Temperature	150.15 K	
Wavelength	0.71076 Å	
Crystal system	Triclinic	
Space group	P-1	
Unit cell dimensions	a = 11.1790(6) Å	a= 96.455(2)°.
	b = 11.5699(7) Å	b= 106.583(3)°.
	c = 14.2713(7) Å	g = 97.291(3)°.
Volume	1733.19(17) $Å^3$	
Z	2	
Density (calculated)	1.405 Mg/m^3	
Absorption coefficient	0.387 mm^{-1}	
F(000)	756	
Crystal size		
Theta range for data collection	2.786 to 26.433°.	
Index ranges	-14<=h<=13, -14<=k<=14, -17<=l<=17	
Reflections collected	52247	
Independent reflections	7107 [R(int) = 0.0985]	
Completeness to theta = 25.243°	99.8 %	
Absorption correction	None	
Refinement method	Full-matrix least-squares on F^2	
Data / restraints / parameters	7107 / 0 / 447	
Goodness-of-fit on F^2	1.102	
Final R indices [I>2sigma(I)]	R1 = 0.0807, wR2 = 0.1829	
R indices (all data)	R1 = 0.1016, wR2 = 0.1936	
Extinction coefficient	n/a	
Largest diff. peak and hole	1.060 and -0.831 e.$Å^{-3}$	

Table S2. Atomic coordinates (x 10^4) and equivalent isotropic displacement parameters ($Å^2$x 10^3) for **3f**. U(eq) is defined as one third of the trace of the orthogonalized U^{ij} tensor.

	x	y	z	U(eq)
O(005)	3557(2)	4797(2)	686(2)	22(1)
O(006)	-305(2)	7706(2)	6423(2)	26(1)
O(007)	2069(3)	9091(2)	3492(2)	28(1)
N(009)	1473(3)	5857(2)	1067(2)	15(1)
N(00A)	5298(3)	5527(3)	2031(2)	19(1)
N(00B)	2924(3)	2635(3)	2376(2)	28(1)
C(00C)	1291(3)	4733(3)	1200(2)	15(1)
C(00D)	4395(3)	6043(3)	3242(2)	14(1)
C(00E)	5533(3)	5931(3)	3048(2)	18(1)
C(00F)	2220(3)	4687(3)	2226(2)	13(1)
C(00G)	1497(3)	6320(3)	3225(2)	15(1)
C(00H)	4043(3)	5275(3)	1545(2)	17(1)
C(00I)	2594(3)	3519(3)	2298(2)	16(1)
C(00J)	972(3)	6677(3)	4034(2)	16(1)
C(00K)	1964(3)	7071(3)	2710(2)	15(1)
C(00L)	2481(3)	6601(3)	1888(2)	14(1)
C(00M)	6766(3)	4177(3)	1726(3)	21(1)
C(00N)	1271(3)	6173(3)	4910(3)	19(1)
C(00O)	3090(3)	7690(3)	1602(2)	16(1)
C(00P)	4432(3)	6412(3)	4212(2)	18(1)
C(00Q)	2952(3)	8699(3)	2168(3)	19(1)
C(00R)	6280(3)	5320(3)	1569(3)	22(1)
C(00S)	6706(3)	6528(3)	4736(3)	23(1)
C(00T)	2278(3)	8389(3)	2886(3)	19(1)
C(00U)	1574(3)	5017(3)	3028(2)	16(1)
C(00V)	3332(3)	5672(3)	2283(2)	12(1)
C(00W)	841(3)	6544(3)	5691(3)	19(1)
C(00X)	5597(3)	6651(3)	4957(3)	23(1)
C(00Y)	6692(3)	6165(3)	3768(3)	23(1)
C(00Z)	199(3)	7535(3)	3974(3)	21(1)
C(010)	-240(3)	7915(3)	4752(3)	24(1)
C(011)	91(3)	7417(3)	5619(3)	20(1)
C(012)	7859(3)	3997(3)	1476(3)	24(1)

C(013)	6141(3)	3278(4)	2066(3)	28(1)
C(014)	7654(4)	2039(3)	1890(3)	30(1)
C(015)	3365(4)	9821(3)	2006(3)	27(1)
C(016)	8291(4)	2930(4)	1556(3)	28(1)
C(017)	3648(4)	7785(3)	855(3)	25(1)
C(018)	4045(4)	8909(4)	689(3)	32(1)
C(019)	-769(4)	8805(4)	6508(3)	32(1)
C(01A)	6593(4)	2217(4)	2152(3)	32(1)
C(01B)	3899(4)	9912(3)	1251(3)	32(1)
Cl(1)	9887(3)	8729(3)	960(2)	176(2)
Cl(00)	7210(3)	8402(2)	921(2)	126(1)
C(01C)	8294(7)	8234(8)	276(5)	97(3)
Cl(01)	4272(1)	-999(1)	5730(1)	56(1)
Cl(02)	3034(2)	620(1)	6712(1)	71(1)
C(01D)	3487(10)	199(7)	5698(5)	103(3)
O(1)	524(2)	3901(2)	671(2)	19(1)

Table S3. Bond lengths [Å] and angles [°] for **3f**.

O(005)-C(00H)	1.222(4)
O(006)-C(011)	1.366(4)
O(006)-C(019)	1.438(5)
O(007)-C(00T)	1.212(4)
N(009)-C(00C)	1.334(4)
N(009)-C(00L)	1.469(4)
N(009)-H(009)	0.95(4)
N(00A)-C(00E)	1.412(4)
N(00A)-C(00H)	1.353(4)
N(00A)-C(00R)	1.463(4)
N(00B)-C(00I)	1.137(4)
C(00C)-C(00F)	1.545(4)
C(00C)-O(1)	1.221(4)
C(00D)-C(00E)	1.396(5)
C(00D)-C(00P)	1.388(5)
C(00D)-C(00V)	1.510(4)
C(00E)-C(00Y)	1.377(5)
C(00F)-C(00I)	1.471(4)
C(00F)-C(00U)	1.555(4)
C(00F)-C(00V)	1.553(4)
C(00G)-C(00J)	1.479(4)
C(00G)-C(00K)	1.347(5)
C(00G)-C(00U)	1.520(4)
C(00H)-C(00V)	1.554(4)
C(00J)-C(00N)	1.410(5)
C(00J)-C(00Z)	1.391(5)
C(00K)-C(00L)	1.527(4)
C(00K)-C(00T)	1.496(5)
C(00L)-C(00O)	1.508(4)
C(00L)-C(00V)	1.571(4)
C(00M)-C(00R)	1.508(5)
C(00M)-C(012)	1.401(5)
C(00M)-C(013)	1.388(5)
C(00N)-H(00N)	0.9500
C(00N)-C(00W)	1.382(5)
C(00O)-C(00Q)	1.394(5)

C(00O)-C(017)	1.388(5)
C(00P)-H(00P)	0.9500
C(00P)-C(00X)	1.399(5)
C(00Q)-C(00T)	1.485(5)
C(00Q)-C(015)	1.389(5)
C(00R)-H(00A)	0.9900
C(00R)-H(00B)	0.9900
C(00S)-H(00S)	0.9500
C(00S)-C(00X)	1.384(5)
C(00S)-C(00Y)	1.393(5)
C(00U)-H(00C)	0.9900
C(00U)-H(00D)	0.9900
C(00W)-H(00W)	0.9500
C(00W)-C(011)	1.387(5)
C(00X)-H(00X)	0.9500
C(00Y)-H(00Y)	0.9500
C(00Z)-H(00Z)	0.9500
C(00Z)-C(010)	1.388(5)
C(010)-H(010)	0.9500
C(010)-C(011)	1.397(5)
C(012)-H(012)	0.9500
C(012)-C(016)	1.387(5)
C(013)-H(013)	0.9500
C(013)-C(01A)	1.389(6)
C(014)-H(014)	0.9500
C(014)-C(016)	1.383(6)
C(014)-C(01A)	1.374(6)
C(015)-H(015)	0.9500
C(015)-C(01B)	1.379(6)
C(016)-H(016)	0.9500
C(017)-H(017)	0.9500
C(017)-C(018)	1.389(5)
C(018)-H(018)	0.9500
C(018)-C(01B)	1.389(6)
C(019)-H(01A)	0.9800
C(019)-H(01B)	0.9800
C(019)-H(01C)	0.9800
C(01A)-H(01D)	0.9500

C(01B)-H(01E)	0.9500
Cl(1)-C(01C)	1.753(7)
Cl(00)-C(01C)	1.736(7)
C(01C)-H(01F)	0.9900
C(01C)-H(01G)	0.9900
Cl(01)-C(01D)	1.730(6)
Cl(02)-C(01D)	1.705(6)
C(01D)-H(01H)	0.9900
C(01D)-H(01I)	0.9900
C(011)-O(006)-C(019)	117.4(3)
C(00C)-N(009)-C(00L)	112.7(3)
C(00C)-N(009)-H(009)	123(3)
C(00L)-N(009)-H(009)	124(3)
C(00E)-N(00A)-C(00R)	124.5(3)
C(00H)-N(00A)-C(00E)	111.6(3)
C(00H)-N(00A)-C(00R)	123.8(3)
N(009)-C(00C)-C(00F)	105.5(3)
O(1)-C(00C)-N(009)	129.2(3)
O(1)-C(00C)-C(00F)	125.2(3)
C(00E)-C(00D)-C(00V)	108.2(3)
C(00P)-C(00D)-C(00E)	118.3(3)
C(00P)-C(00D)-C(00V)	133.5(3)
C(00D)-C(00E)-N(00A)	109.9(3)
C(00Y)-C(00E)-N(00A)	126.6(3)
C(00Y)-C(00E)-C(00D)	123.5(3)
C(00C)-C(00F)-C(00U)	108.3(2)
C(00C)-C(00F)-C(00V)	101.4(2)
C(00I)-C(00F)-C(00C)	112.1(3)
C(00I)-C(00F)-C(00U)	110.2(3)
C(00I)-C(00F)-C(00V)	113.4(3)
C(00V)-C(00F)-C(00U)	111.0(2)
C(00J)-C(00G)-C(00U)	115.9(3)
C(00K)-C(00G)-C(00J)	124.6(3)
C(00K)-C(00G)-C(00U)	119.4(3)
O(005)-C(00H)-N(00A)	126.1(3)
O(005)-C(00H)-C(00V)	126.3(3)
N(00A)-C(00H)-C(00V)	107.6(3)

N(00B)-C(00I)-C(00F)	177.6(4)
C(00N)-C(00J)-C(00G)	120.6(3)
C(00Z)-C(00J)-C(00G)	121.7(3)
C(00Z)-C(00J)-C(00N)	117.7(3)
C(00G)-C(00K)-C(00L)	120.0(3)
C(00G)-C(00K)-C(00T)	130.9(3)
C(00T)-C(00K)-C(00L)	108.3(3)
N(009)-C(00L)-C(00K)	111.1(3)
N(009)-C(00L)-C(00O)	113.9(3)
N(009)-C(00L)-C(00V)	101.1(2)
C(00K)-C(00L)-C(00V)	107.9(2)
C(00O)-C(00L)-C(00K)	104.5(3)
C(00O)-C(00L)-C(00V)	118.4(3)
C(012)-C(00M)-C(00R)	118.5(3)
C(013)-C(00M)-C(00R)	122.6(3)
C(013)-C(00M)-C(012)	118.8(3)
C(00J)-C(00N)-H(00N)	119.5
C(00W)-C(00N)-C(00J)	121.1(3)
C(00W)-C(00N)-H(00N)	119.5
C(00Q)-C(00O)-C(00L)	110.2(3)
C(017)-C(00O)-C(00L)	129.2(3)
C(017)-C(00O)-C(00Q)	120.4(3)
C(00D)-C(00P)-H(00P)	120.3
C(00D)-C(00P)-C(00X)	119.3(3)
C(00X)-C(00P)-H(00P)	120.3
C(00O)-C(00Q)-C(00T)	111.1(3)
C(015)-C(00Q)-C(00O)	121.5(3)
C(015)-C(00Q)-C(00T)	127.3(3)
N(00A)-C(00R)-C(00M)	114.3(3)
N(00A)-C(00R)-H(00A)	108.7
N(00A)-C(00R)-H(00B)	108.7
C(00M)-C(00R)-H(00A)	108.7
C(00M)-C(00R)-H(00B)	108.7
H(00A)-C(00R)-H(00B)	107.6
C(00X)-C(00S)-H(00S)	119.5
C(00X)-C(00S)-C(00Y)	121.0(3)
C(00Y)-C(00S)-H(00S)	119.5
O(007)-C(00T)-C(00K)	129.0(3)

O(007)-C(00T)-C(00Q)	125.1(3)
C(00Q)-C(00T)-C(00K)	105.8(3)
C(00F)-C(00U)-H(00C)	108.6
C(00F)-C(00U)-H(00D)	108.6
C(00G)-C(00U)-C(00F)	114.5(3)
C(00G)-C(00U)-H(00C)	108.6
C(00G)-C(00U)-H(00D)	108.6
H(00C)-C(00U)-H(00D)	107.6
C(00D)-C(00V)-C(00F)	119.2(3)
C(00D)-C(00V)-C(00H)	102.3(3)
C(00D)-C(00V)-C(00L)	118.3(3)
C(00F)-C(00V)-C(00H)	110.2(3)
C(00F)-C(00V)-C(00L)	95.9(2)
C(00H)-C(00V)-C(00L)	111.1(2)
C(00N)-C(00W)-H(00W)	119.9
C(00N)-C(00W)-C(011)	120.1(3)
C(011)-C(00W)-H(00W)	119.9
C(00P)-C(00X)-H(00X)	119.7
C(00S)-C(00X)-C(00P)	120.6(3)
C(00S)-C(00X)-H(00X)	119.7
C(00E)-C(00Y)-C(00S)	117.2(3)
C(00E)-C(00Y)-H(00Y)	121.4
C(00S)-C(00Y)-H(00Y)	121.4
C(00J)-C(00Z)-H(00Z)	119.1
C(010)-C(00Z)-C(00J)	121.8(3)
C(010)-C(00Z)-H(00Z)	119.1
C(00Z)-C(010)-H(010)	120.3
C(00Z)-C(010)-C(011)	119.3(3)
C(011)-C(010)-H(010)	120.3
O(006)-C(011)-C(00W)	115.6(3)
O(006)-C(011)-C(010)	124.4(3)
C(00W)-C(011)-C(010)	120.0(3)
C(00M)-C(012)-H(012)	120.0
C(016)-C(012)-C(00M)	120.0(3)
C(016)-C(012)-H(012)	120.0
C(00M)-C(013)-H(013)	119.8
C(00M)-C(013)-C(01A)	120.4(3)
C(01A)-C(013)-H(013)	119.8

C(016)-C(014)-H(014)	120.1
C(01A)-C(014)-H(014)	120.1
C(01A)-C(014)-C(016)	119.8(4)
C(00Q)-C(015)-H(015)	121.0
C(01B)-C(015)-C(00Q)	117.9(4)
C(01B)-C(015)-H(015)	121.0
C(012)-C(016)-H(016)	119.8
C(014)-C(016)-C(012)	120.5(4)
C(014)-C(016)-H(016)	119.8
C(00O)-C(017)-H(017)	121.1
C(00O)-C(017)-C(018)	117.8(4)
C(018)-C(017)-H(017)	121.1
C(017)-C(018)-H(018)	119.2
C(017)-C(018)-C(01B)	121.5(4)
C(01B)-C(018)-H(018)	119.2
O(006)-C(019)-H(01A)	109.5
O(006)-C(019)-H(01B)	109.5
O(006)-C(019)-H(01C)	109.5
H(01A)-C(019)-H(01B)	109.5
H(01A)-C(019)-H(01C)	109.5
H(01B)-C(019)-H(01C)	109.5
C(013)-C(01A)-H(01D)	119.8
C(014)-C(01A)-C(013)	120.5(4)
C(014)-C(01A)-H(01D)	119.8
C(015)-C(01B)-C(018)	120.8(4)
C(015)-C(01B)-H(01E)	119.6
C(018)-C(01B)-H(01E)	119.6
Cl(1)-C(01C)-H(01F)	108.4
Cl(1)-C(01C)-H(01G)	108.4
Cl(00)-C(01C)-Cl(1)	115.5(4)
Cl(00)-C(01C)-H(01F)	108.4
Cl(00)-C(01C)-H(01G)	108.4
H(01F)-C(01C)-H(01G)	107.5
Cl(01)-C(01D)-H(01H)	108.1
Cl(01)-C(01D)-H(01I)	108.1
Cl(02)-C(01D)-Cl(01)	116.8(4)
Cl(02)-C(01D)-H(01H)	108.1
Cl(02)-C(01D)-H(01I)	108.1

H(01H)-C(01D)-H(01I)	107.3

Symmetry transformations used to generate equivalent atoms:

Table S4. Anisotropic displacement parameters ($Å^2$x 10^3) for **3f**. The anisotropic displacement factor exponent takes the form: $-2p^2[h^2 a^{*2}U^{11} + ... + 2 h k a^* b^* U^{12}]$

	U^{11}	U^{22}	U^{33}	U^{23}	U^{13}	U^{12}
O(005)	22(1)	28(1)	13(1)	-2(1)	4(1)	5(1)
O(006)	25(1)	32(2)	25(1)	-1(1)	13(1)	10(1)
O(007)	37(2)	16(1)	31(2)	-2(1)	12(1)	9(1)
N(009)	14(1)	15(1)	13(1)	3(1)	0(1)	3(1)
N(00A)	15(1)	26(2)	16(1)	2(1)	5(1)	5(1)
N(00B)	36(2)	17(2)	31(2)	4(1)	9(2)	10(1)
C(00C)	12(2)	19(2)	12(2)	1(1)	2(1)	6(1)
C(00D)	14(2)	11(2)	16(2)	4(1)	1(1)	2(1)
C(00E)	19(2)	15(2)	18(2)	6(1)	3(1)	3(1)
C(00F)	15(2)	11(2)	14(2)	2(1)	4(1)	5(1)
C(00G)	12(2)	18(2)	13(2)	1(1)	-1(1)	6(1)
C(00H)	22(2)	16(2)	16(2)	8(1)	6(1)	7(1)
C(00I)	20(2)	15(2)	13(2)	2(1)	4(1)	4(1)
C(00J)	12(2)	18(2)	16(2)	-1(1)	2(1)	4(1)
C(00K)	14(2)	14(2)	12(2)	0(1)	-2(1)	4(1)
C(00L)	14(2)	15(2)	13(2)	5(1)	0(1)	3(1)
C(00M)	18(2)	26(2)	17(2)	2(1)	4(1)	3(1)
C(00N)	18(2)	18(2)	21(2)	0(1)	5(1)	6(1)
C(00O)	14(2)	16(2)	15(2)	4(1)	-3(1)	1(1)
C(00P)	20(2)	19(2)	15(2)	1(1)	3(1)	5(1)
C(00Q)	18(2)	16(2)	19(2)	2(1)	0(1)	2(1)
C(00R)	20(2)	28(2)	20(2)	6(1)	9(1)	6(2)
C(00S)	16(2)	21(2)	22(2)	6(1)	-8(1)	1(1)
C(00T)	18(2)	16(2)	21(2)	5(1)	1(1)	6(1)
C(00U)	17(2)	15(2)	15(2)	4(1)	5(1)	3(1)
C(00V)	14(2)	14(2)	8(1)	2(1)	1(1)	5(1)
C(00W)	20(2)	21(2)	17(2)	2(1)	7(1)	3(1)
C(00X)	26(2)	23(2)	13(2)	2(1)	-1(1)	3(2)
C(00Y)	14(2)	23(2)	29(2)	4(2)	2(1)	3(1)
C(00Z)	19(2)	25(2)	22(2)	6(1)	6(1)	8(1)
C(010)	20(2)	24(2)	30(2)	4(2)	9(2)	11(2)
C(011)	16(2)	22(2)	21(2)	-6(1)	6(1)	2(1)
C(012)	16(2)	32(2)	22(2)	3(2)	4(1)	1(2)

C(013)	15(2)	33(2)	36(2)	9(2)	8(2)	5(2)
C(014)	29(2)	24(2)	30(2)	0(2)	0(2)	8(2)
C(015)	30(2)	16(2)	31(2)	6(2)	1(2)	0(2)
C(016)	20(2)	37(2)	26(2)	0(2)	4(2)	10(2)
C(017)	28(2)	26(2)	19(2)	4(1)	4(2)	-2(2)
C(018)	33(2)	33(2)	27(2)	10(2)	8(2)	-8(2)
C(019)	34(2)	29(2)	37(2)	-5(2)	16(2)	10(2)
C(01A)	23(2)	29(2)	38(2)	9(2)	3(2)	-1(2)
C(01B)	33(2)	22(2)	34(2)	10(2)	1(2)	-7(2)
Cl(1)	132(2)	156(3)	150(2)	-57(2)	-90(2)	76(2)
Cl(00)	238(3)	83(1)	121(2)	42(1)	122(2)	78(2)
C(01C)	88(5)	145(7)	44(3)	-29(4)	-13(3)	80(5)
Cl(01)	68(1)	52(1)	48(1)	4(1)	14(1)	26(1)
Cl(02)	107(1)	67(1)	59(1)	22(1)	34(1)	54(1)
C(01D)	184(9)	109(6)	46(3)	26(4)	41(5)	107(6)
O(1)	19(1)	15(1)	17(1)	0(1)	-1(1)	0(1)

Table S5. Atomic coordinates (x 10^4) and equivalent isotropic displacement parameters (Å^{2}x 10^3) for **3f**.

	x	y	z	U(eq)
H(00N)	1777	5569	4963	23
H(00P)	3673	6500	4367	22
H(00A)	6999	5980	1837	26
H(00B)	5936	5323	849	26
H(00S)	7489	6694	5251	27
H(00C)	705	4559	2818	19
H(00D)	2047	4777	3654	19
H(00W)	1059	6200	6278	23
H(00X)	5628	6901	5622	27
H(00Y)	7450	6082	3611	28
H(00Z)	-34	7871	3383	26
H(010)	-761	8508	4697	28
H(012)	8305	4607	1251	29
H(013)	5400	3388	2241	33
H(014)	7949	1307	1938	35
H(015)	3283	10505	2403	33
H(016)	9029	2809	1381	34
H(017)	3755	7103	470	30
H(018)	4426	8995	180	38
H(01A)	-1586	8745	5999	48
H(01B)	-162	9439	6418	48
H(01C)	-875	8979	7165	48
H(01D)	6165	1611	2394	38
H(01E)	4169	10669	1114	39
H(01F)	8084	8664	-296	117
H(01G)	8193	7387	10	117
H(01H)	4041	880	5597	124
H(01I)	2721	16	5114	124
H(009)	940(40)	6150(40)	540(30)	29(11)

Table S6. Torsion angles [°] for **3f**.

O(005)-C(00H)-C(00V)-C(00D)	172.8(3)
O(005)-C(00H)-C(00V)-C(00F)	45.0(4)
O(005)-C(00H)-C(00V)-C(00L)	-60.0(4)
N(009)-C(00C)-C(00F)-C(00I)	-152.7(3)
N(009)-C(00C)-C(00F)-C(00U)	85.5(3)
N(009)-C(00C)-C(00F)-C(00V)	-31.5(3)
N(009)-C(00L)-C(00O)-C(00Q)	120.8(3)
N(009)-C(00L)-C(00O)-C(017)	-54.7(5)
N(009)-C(00L)-C(00V)-C(00D)	-171.0(3)
N(009)-C(00L)-C(00V)-C(00F)	-43.2(3)
N(009)-C(00L)-C(00V)-C(00H)	71.1(3)
N(00A)-C(00E)-C(00Y)-C(00S)	-178.4(3)
N(00A)-C(00H)-C(00V)-C(00D)	-5.7(3)
N(00A)-C(00H)-C(00V)-C(00F)	-133.5(3)
N(00A)-C(00H)-C(00V)-C(00L)	121.5(3)
C(00C)-N(009)-C(00L)-C(00K)	-86.9(3)
C(00C)-N(009)-C(00L)-C(00O)	155.4(3)
C(00C)-N(009)-C(00L)-C(00V)	27.4(3)
C(00C)-C(00F)-C(00U)-C(00G)	-74.0(3)
C(00C)-C(00F)-C(00V)-C(00D)	171.8(3)
C(00C)-C(00F)-C(00V)-C(00H)	-70.4(3)
C(00C)-C(00F)-C(00V)-C(00L)	44.7(3)
C(00D)-C(00E)-C(00Y)-C(00S)	0.2(5)
C(00D)-C(00P)-C(00X)-C(00S)	0.2(5)
C(00E)-N(00A)-C(00H)-O(005)	-171.9(3)
C(00E)-N(00A)-C(00H)-C(00V)	6.6(4)
C(00E)-N(00A)-C(00R)-C(00M)	78.4(4)
C(00E)-C(00D)-C(00P)-C(00X)	-0.3(5)
C(00E)-C(00D)-C(00V)-C(00F)	124.7(3)
C(00E)-C(00D)-C(00V)-C(00H)	2.9(3)
C(00E)-C(00D)-C(00V)-C(00L)	-119.5(3)
C(00G)-C(00J)-C(00N)-C(00W)	176.6(3)
C(00G)-C(00J)-C(00Z)-C(010)	-176.6(3)
C(00G)-C(00K)-C(00L)-N(009)	65.3(4)
C(00G)-C(00K)-C(00L)-C(00O)	-171.5(3)
C(00G)-C(00K)-C(00L)-C(00V)	-44.7(4)
C(00G)-C(00K)-C(00T)-O(007)	-7.8(6)

C(00G)-C(00K)-C(00T)-C(00Q)	170.9(3)
C(00H)-N(00A)-C(00E)-C(00D)	-4.8(4)
C(00H)-N(00A)-C(00E)-C(00Y)	173.9(3)
C(00H)-N(00A)-C(00R)-C(00M)	-96.7(4)
C(00I)-C(00F)-C(00U)-C(00G)	163.0(3)
C(00I)-C(00F)-C(00V)-C(00D)	-67.8(4)
C(00I)-C(00F)-C(00V)-C(00H)	50.0(3)
C(00I)-C(00F)-C(00V)-C(00L)	165.0(3)
C(00J)-C(00G)-C(00K)-C(00L)	-180.0(3)
C(00J)-C(00G)-C(00K)-C(00T)	11.1(6)
C(00J)-C(00G)-C(00U)-C(00F)	-175.7(3)
C(00J)-C(00N)-C(00W)-C(011)	0.7(5)
C(00J)-C(00Z)-C(010)-C(011)	-0.6(6)
C(00K)-C(00G)-C(00J)-C(00N)	-137.1(3)
C(00K)-C(00G)-C(00J)-C(00Z)	41.3(5)
C(00K)-C(00G)-C(00U)-C(00F)	0.5(4)
C(00K)-C(00L)-C(00O)-C(00Q)	-0.6(3)
C(00K)-C(00L)-C(00O)-C(017)	-176.1(3)
C(00K)-C(00L)-C(00V)-C(00D)	-54.3(4)
C(00K)-C(00L)-C(00V)-C(00F)	73.4(3)
C(00K)-C(00L)-C(00V)-C(00H)	-172.3(3)
C(00L)-N(009)-C(00C)-C(00F)	2.3(3)
C(00L)-N(009)-C(00C)-O(1)	178.8(3)
C(00L)-C(00K)-C(00T)-O(007)	-177.7(3)
C(00L)-C(00K)-C(00T)-C(00Q)	1.0(3)
C(00L)-C(00O)-C(00Q)-C(00T)	1.2(4)
C(00L)-C(00O)-C(00Q)-C(015)	-176.1(3)
C(00L)-C(00O)-C(017)-C(018)	174.5(3)
C(00M)-C(012)-C(016)-C(014)	-0.5(6)
C(00M)-C(013)-C(01A)-C(014)	-0.9(6)
C(00N)-C(00J)-C(00Z)-C(010)	1.8(5)
C(00N)-C(00W)-C(011)-O(006)	178.9(3)
C(00N)-C(00W)-C(011)-C(010)	0.6(5)
C(00O)-C(00L)-C(00V)-C(00D)	63.9(4)
C(00O)-C(00L)-C(00V)-C(00F)	-168.3(3)
C(00O)-C(00L)-C(00V)-C(00H)	-54.0(4)
C(00O)-C(00Q)-C(00T)-O(007)	177.4(3)
C(00O)-C(00Q)-C(00T)-C(00K)	-1.4(4)

C(00O)-C(00Q)-C(015)-C(01B)	1.2(5)
C(00O)-C(017)-C(018)-C(01B)	0.3(6)
C(00P)-C(00D)-C(00E)-N(00A)	178.9(3)
C(00P)-C(00D)-C(00E)-C(00Y)	0.1(5)
C(00P)-C(00D)-C(00V)-C(00F)	-53.0(5)
C(00P)-C(00D)-C(00V)-C(00H)	-174.8(3)
C(00P)-C(00D)-C(00V)-C(00L)	62.7(5)
C(00Q)-C(00O)-C(017)-C(018)	-0.7(5)
C(00Q)-C(015)-C(01B)-C(018)	-1.6(6)
C(00R)-N(00A)-C(00E)-C(00D)	179.5(3)
C(00R)-N(00A)-C(00E)-C(00Y)	-1.7(5)
C(00R)-N(00A)-C(00H)-O(005)	3.8(5)
C(00R)-N(00A)-C(00H)-C(00V)	-177.7(3)
C(00R)-C(00M)-C(012)-C(016)	-176.2(3)
C(00R)-C(00M)-C(013)-C(01A)	176.8(4)
C(00T)-C(00K)-C(00L)-N(009)	-123.5(3)
C(00T)-C(00K)-C(00L)-C(00O)	-0.3(3)
C(00T)-C(00K)-C(00L)-C(00V)	126.5(3)
C(00T)-C(00Q)-C(015)-C(01B)	-175.6(3)
C(00U)-C(00F)-C(00V)-C(00D)	56.9(4)
C(00U)-C(00F)-C(00V)-C(00H)	174.7(2)
C(00U)-C(00F)-C(00V)-C(00L)	-70.2(3)
C(00U)-C(00G)-C(00J)-C(00N)	38.9(4)
C(00U)-C(00G)-C(00J)-C(00Z)	-142.8(3)
C(00U)-C(00G)-C(00K)-C(00L)	4.2(4)
C(00U)-C(00G)-C(00K)-C(00T)	-164.7(3)
C(00V)-C(00D)-C(00E)-N(00A)	0.7(4)
C(00V)-C(00D)-C(00E)-C(00Y)	-178.1(3)
C(00V)-C(00D)-C(00P)-C(00X)	177.2(3)
C(00V)-C(00F)-C(00U)-C(00G)	36.5(4)
C(00V)-C(00L)-C(00O)-C(00Q)	-120.6(3)
C(00V)-C(00L)-C(00O)-C(017)	63.9(5)
C(00X)-C(00S)-C(00Y)-C(00E)	-0.3(5)
C(00Y)-C(00S)-C(00X)-C(00P)	0.0(5)
C(00Z)-C(00J)-C(00N)-C(00W)	-1.8(5)
C(00Z)-C(010)-C(011)-O(006)	-178.7(3)
C(00Z)-C(010)-C(011)-C(00W)	-0.6(5)
C(012)-C(00M)-C(00R)-N(00A)	-168.4(3)

C(012)-C(00M)-C(013)-C(01A)	-0.2(6)
C(013)-C(00M)-C(00R)-N(00A)	14.7(5)
C(013)-C(00M)-C(012)-C(016)	0.9(5)
C(015)-C(00Q)-C(00T)-O(007)	-5.5(6)
C(015)-C(00Q)-C(00T)-C(00K)	175.7(3)
C(016)-C(014)-C(01A)-C(013)	1.3(6)
C(017)-C(00O)-C(00Q)-C(00T)	177.3(3)
C(017)-C(00O)-C(00Q)-C(015)	0.0(5)
C(017)-C(018)-C(01B)-C(015)	0.9(6)
C(019)-O(006)-C(011)-C(00W)	162.9(3)
C(019)-O(006)-C(011)-C(010)	-18.9(5)
C(01A)-C(014)-C(016)-C(012)	-0.6(6)
O(1)-C(00C)-C(00F)-C(00I)	30.6(4)
O(1)-C(00C)-C(00F)-C(00U)	-91.3(4)
O(1)-C(00C)-C(00F)-C(00V)	151.8(3)

Symmetry transformations used to generate equivalent atoms:

8. Asymmetric catalysis

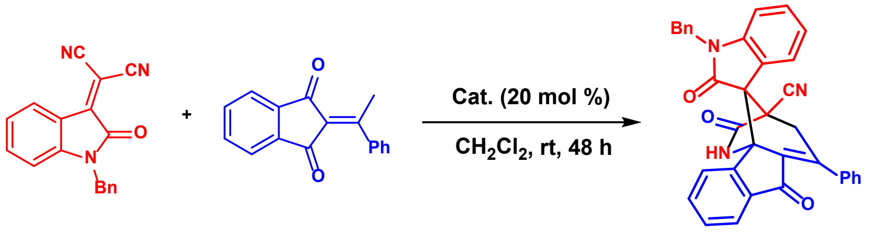

1a 2a 3a

Ar=3,5-$(CF_3)_2C_6H_3$

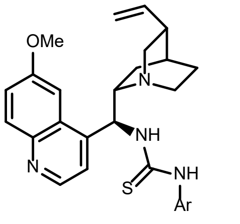

C1

64%, 21% ee, 7:1 dr

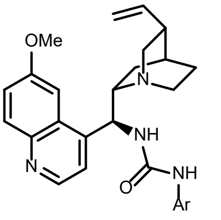

C2

66%, 30% ee, 7:1 dr

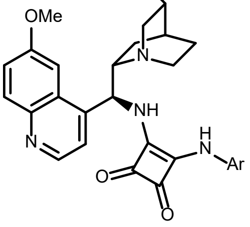

C3

70%, 54% ee, 12:1 dr

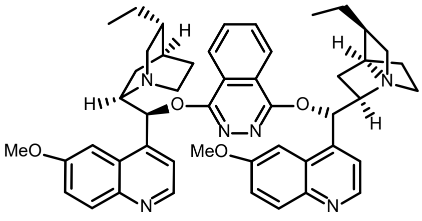

C4

61%, 26% ee, 16:1 dr

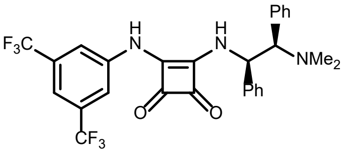

C5

15%, 75% ee, 15:1 dr

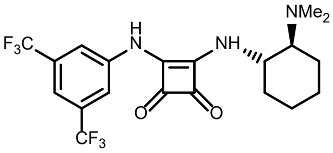

C6

84%, 37% ee, 9:1 dr

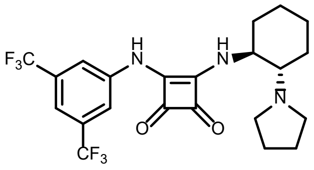

C7

75%, 51% ee, 19:1 dr

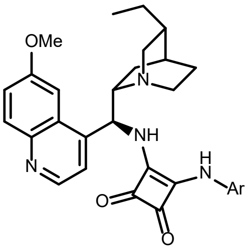

C8

60%, 61% ee, 19:1 dr

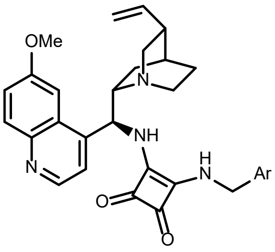

C9

64%, 70% ee, 4:1 dr

9. HPLC Spectra of 3a

With catalyst C9:

rac-**3a**

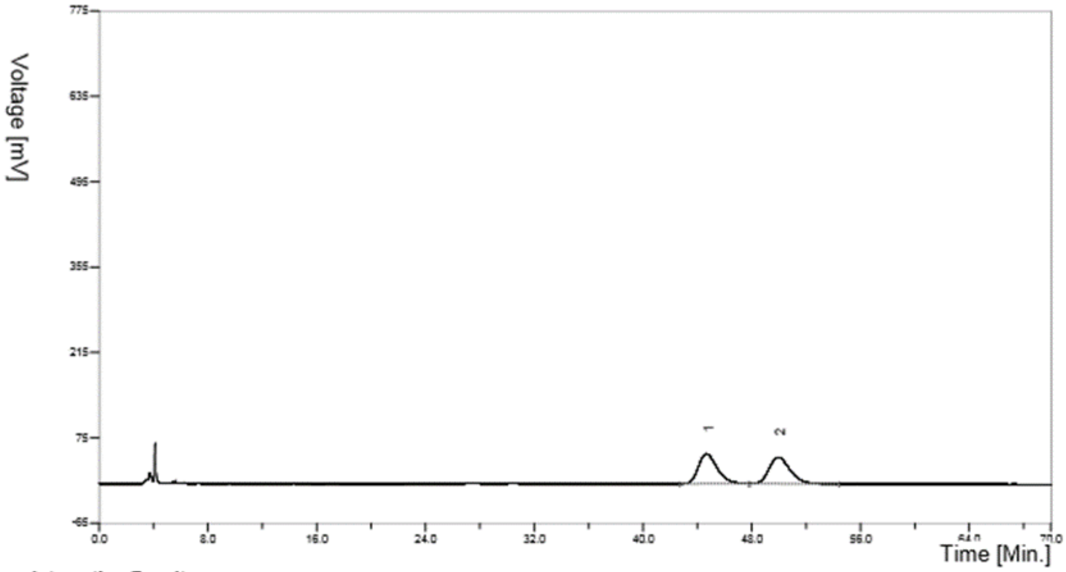

Integration Result

#	Ret. Time(min)	Area Percentage(%)
1	44.66	50.4126
2	49.94	49.5874
Total		100

Chiral **3a**

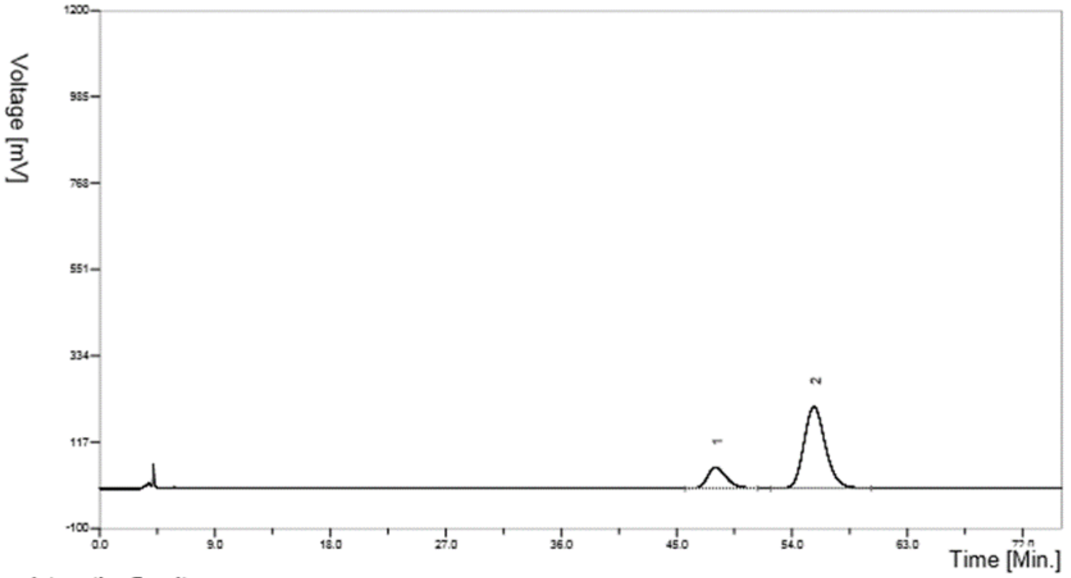

Integration Result

#	Ret. Time(min)	Area Percentage(%)
1	48.05	18.3523
2	55.71	81.6477
Total		100

10. Further substrate scope

(a)

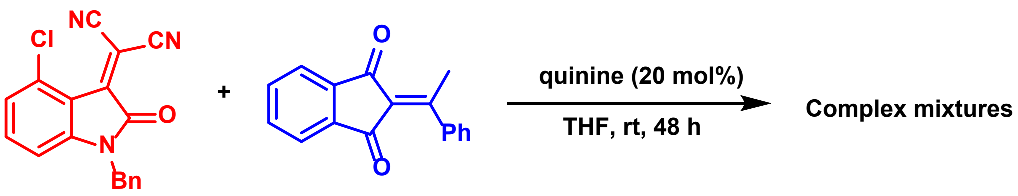

(b)

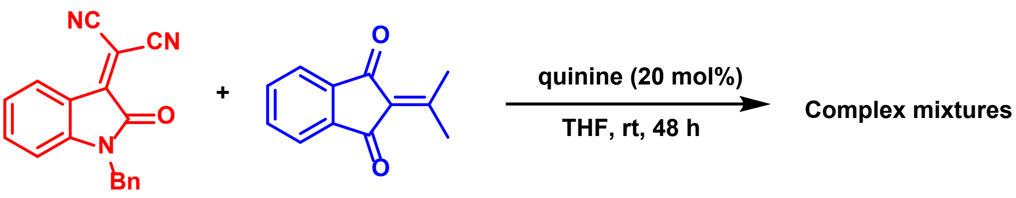

(c)

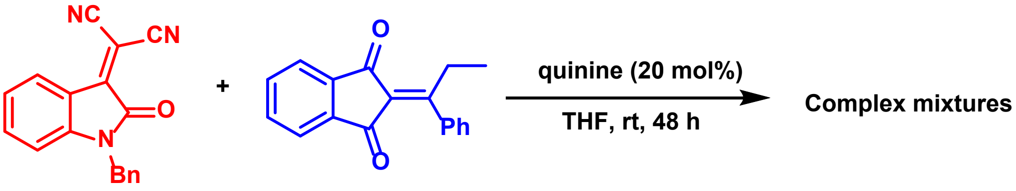

(d)

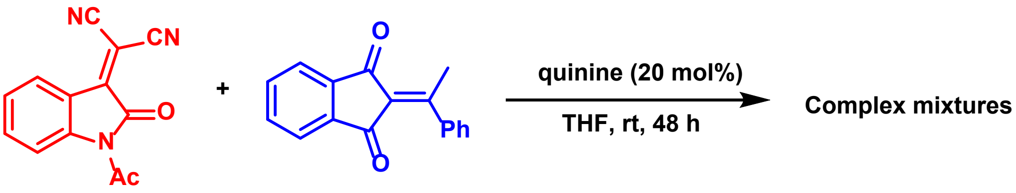

11. ^{1}H NMR and ^{13}C NMR spectra of new compounds

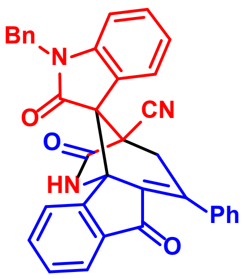

3a

(^{1}H NMR, 400MHz, $CDCl_3$)

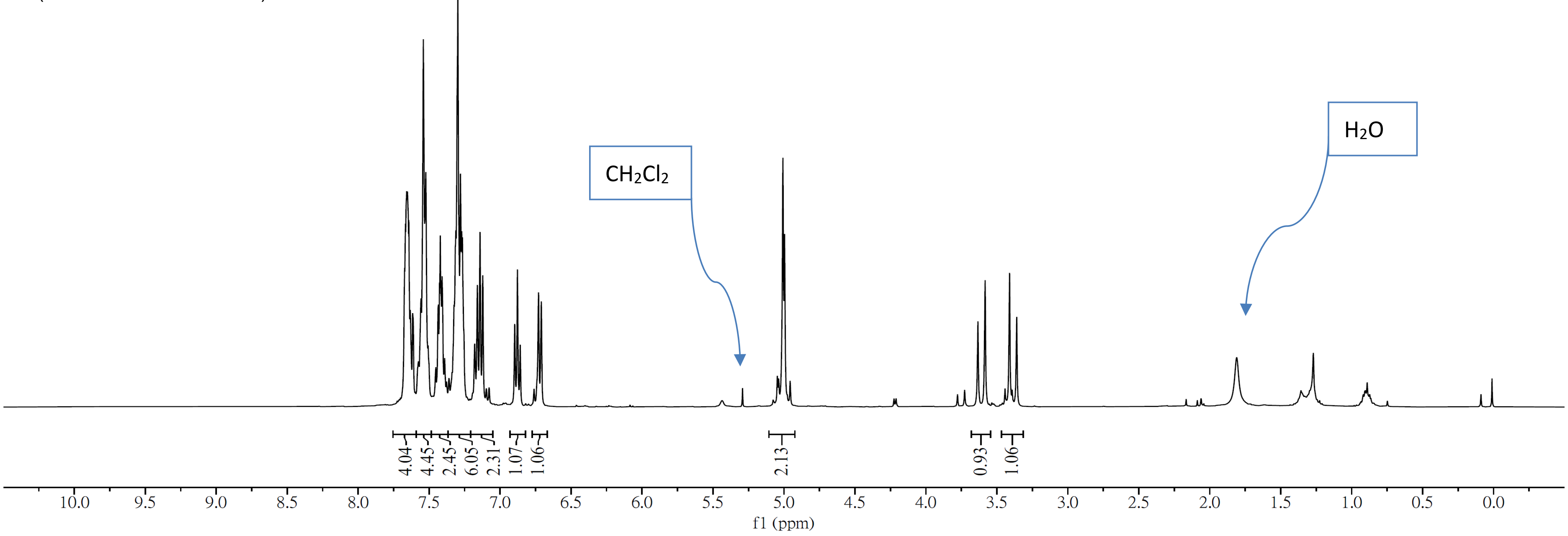

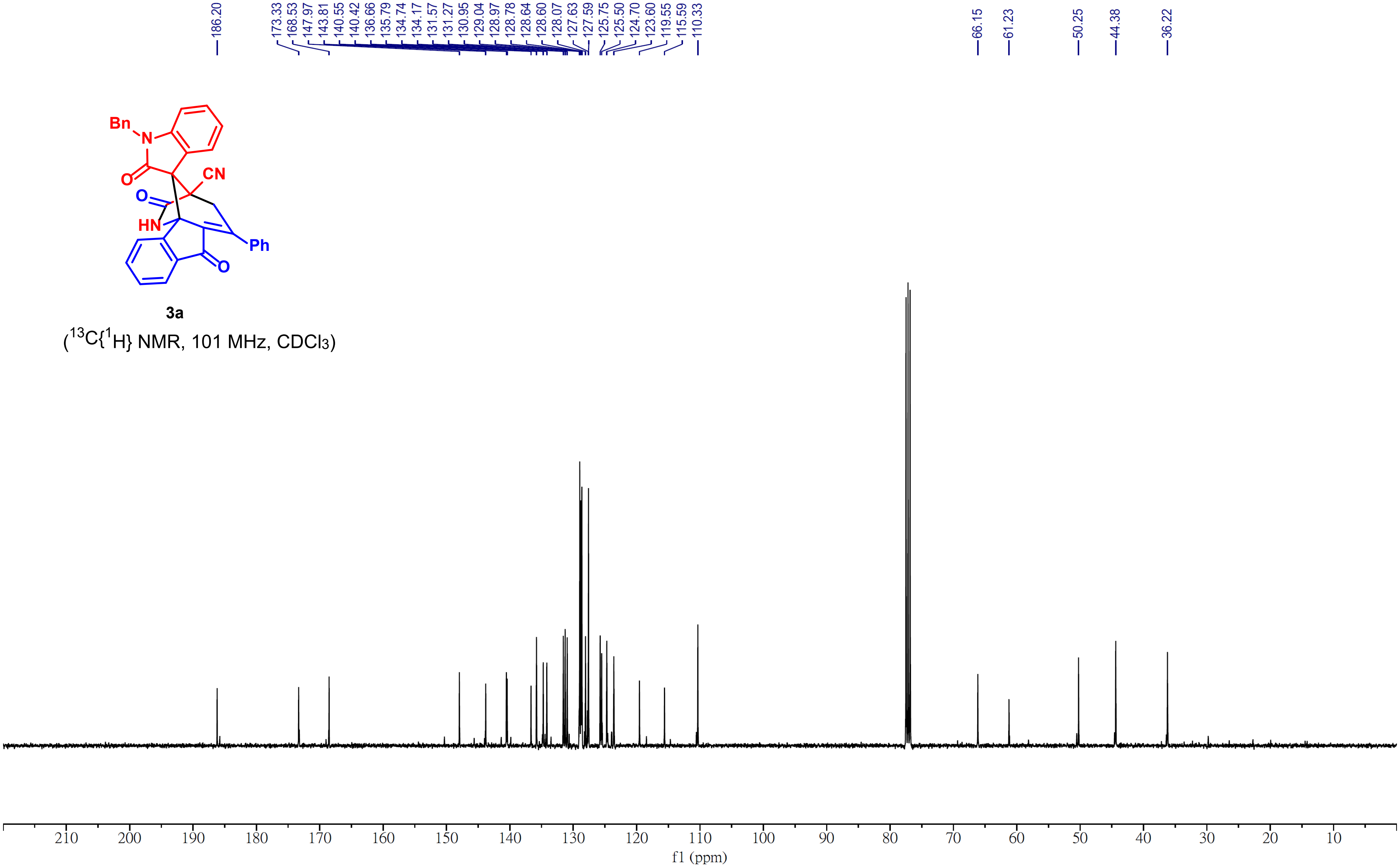

3a

($^{13}C\{^{1}H\}$ NMR, 101 MHz, $CDCl_3$)

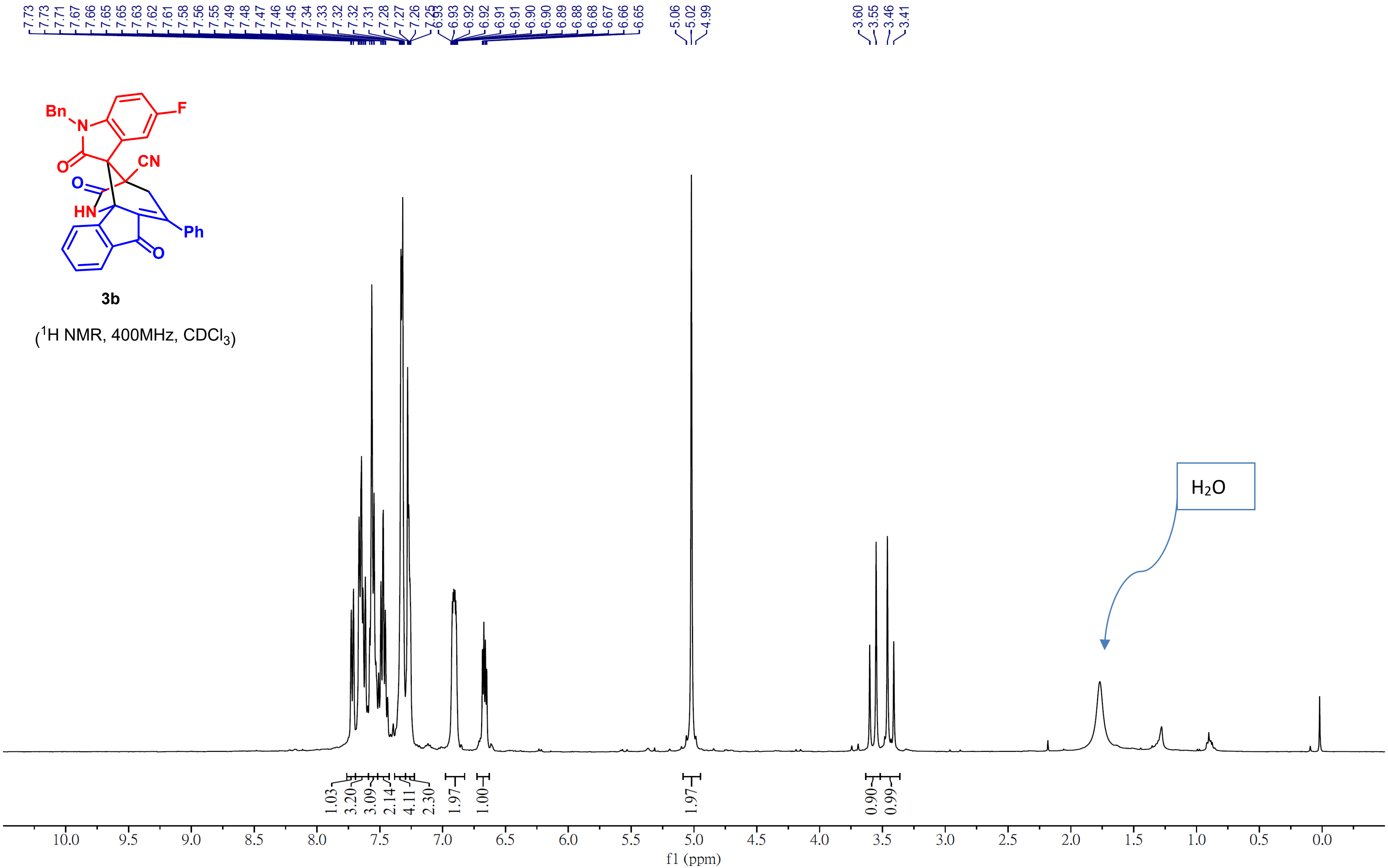
7.73
7.73
7.71
7.67
7.66
7.65
7.65
7.63
7.62
7.61
7.58
7.56
7.55
7.49
7.48
7.47
7.46
7.45
7.34
7.33
7.32
7.32
7.31
7.28
7.27
7.26
7.25
6.93
6.93
6.92
6.92
6.91
6.91
6.90
6.90
6.89
6.88
6.68
6.67
6.66
6.65
5.06
5.02
4.99
3.60
3.55
3.46
3.41
Bn
N
F
O
CN
O
HN
Ph
O
3b
(^{1}H NMR, 400MHz, $CDCl_3$)
H_2O
1.03
3.20
3.09
2.14
4.11
2.30
1.97
1.00
1.97
0.90
0.99
10.0 9.5 9.0 8.5 8.0 7.5 7.0 6.5 6.0 5.5 5.0 4.5 4.0 3.5 3.0 2.5 2.0 1.5 1.0 0.5 0.0
f1 (ppm)

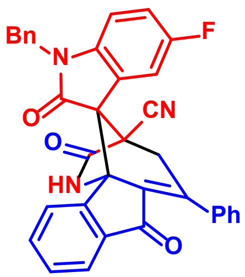

3b

($^{13}C\{^{1}H\}$ NMR, 101 MHz, $CDCl_3$)

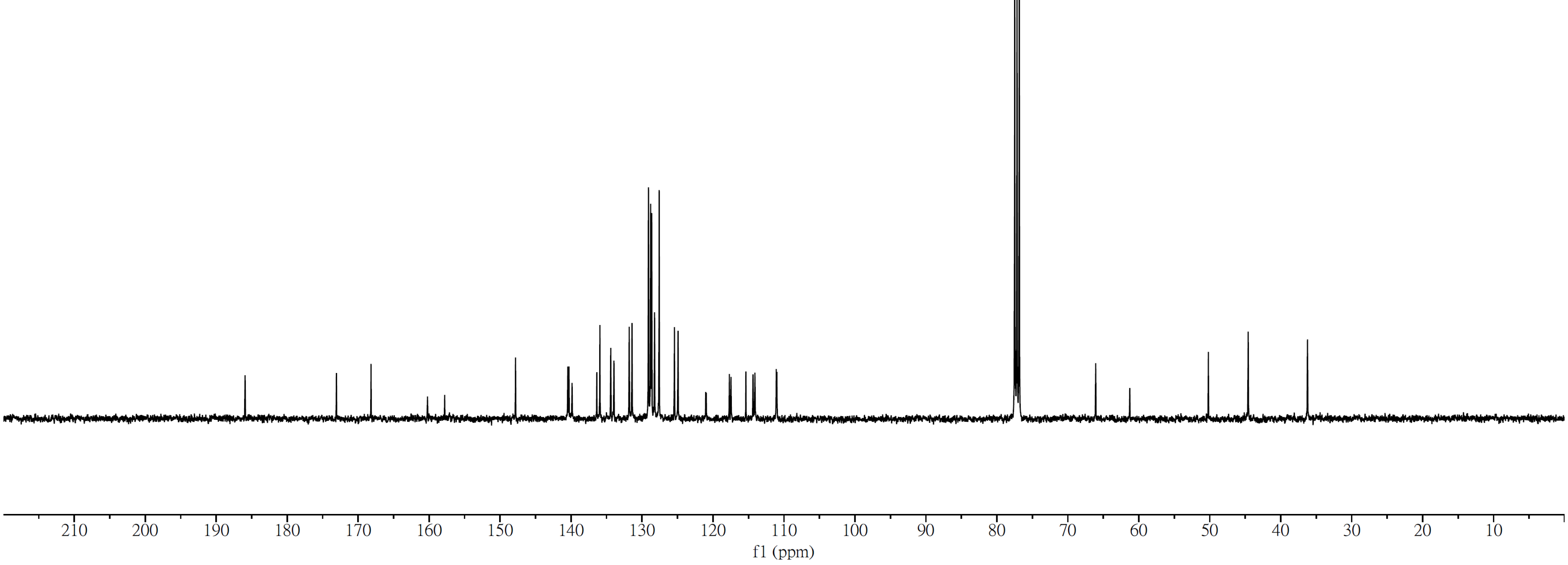

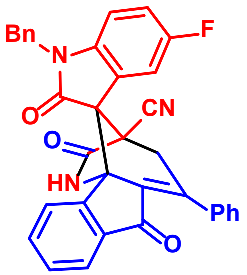

3b

(^{19}F NMR, 376MHz, $CDCl_3$)

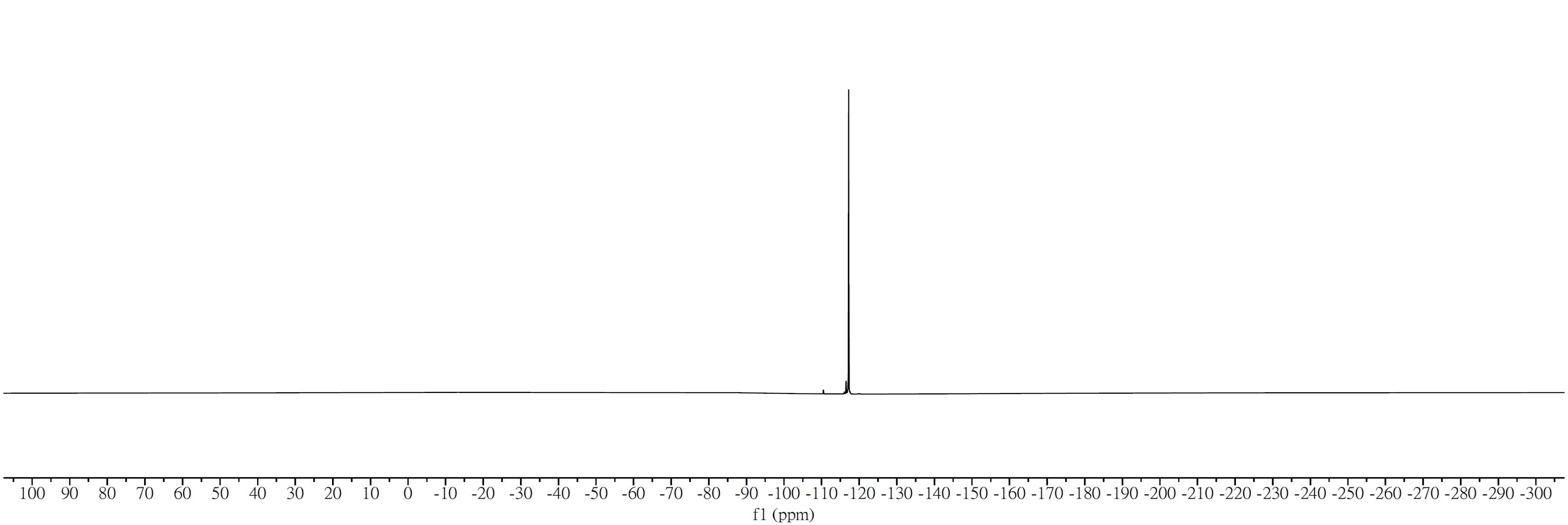

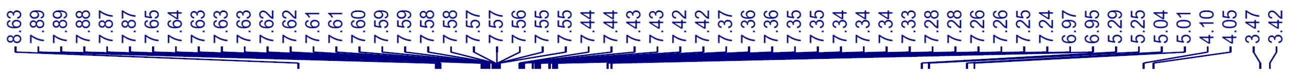

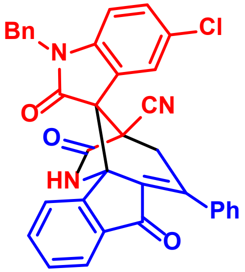

3c

(^{1}H NMR, 400MHz, Acetone-D6)

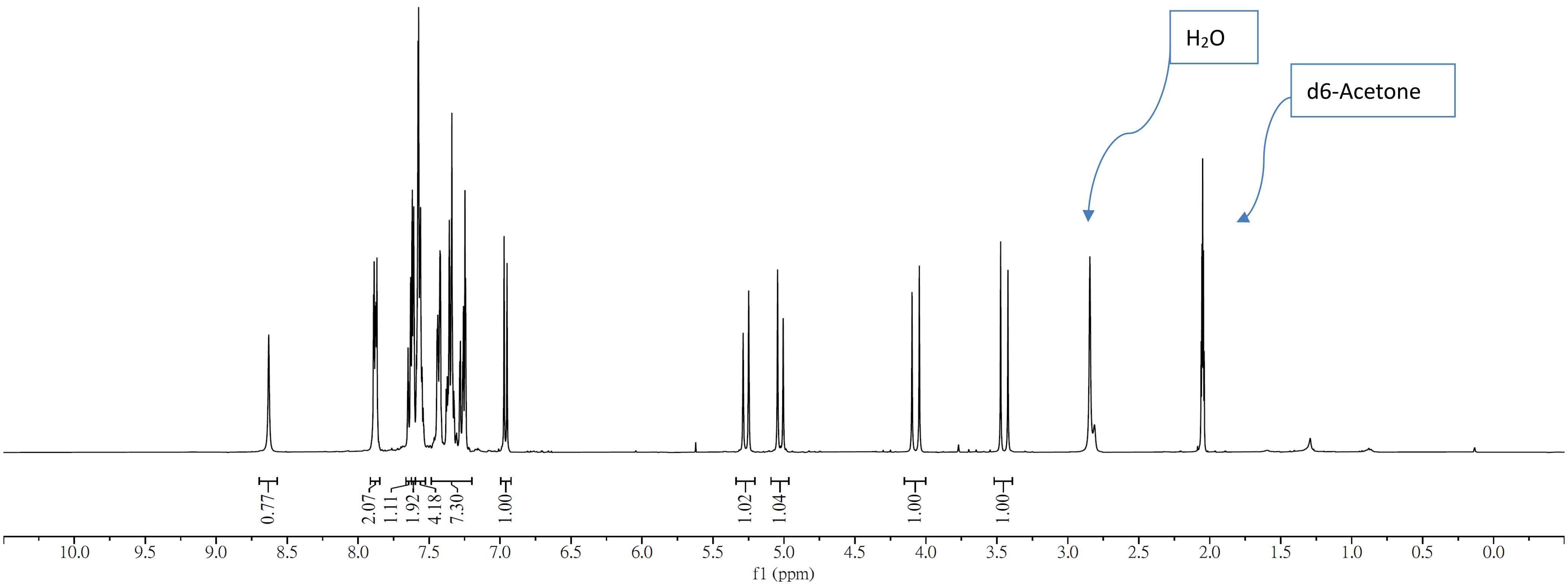

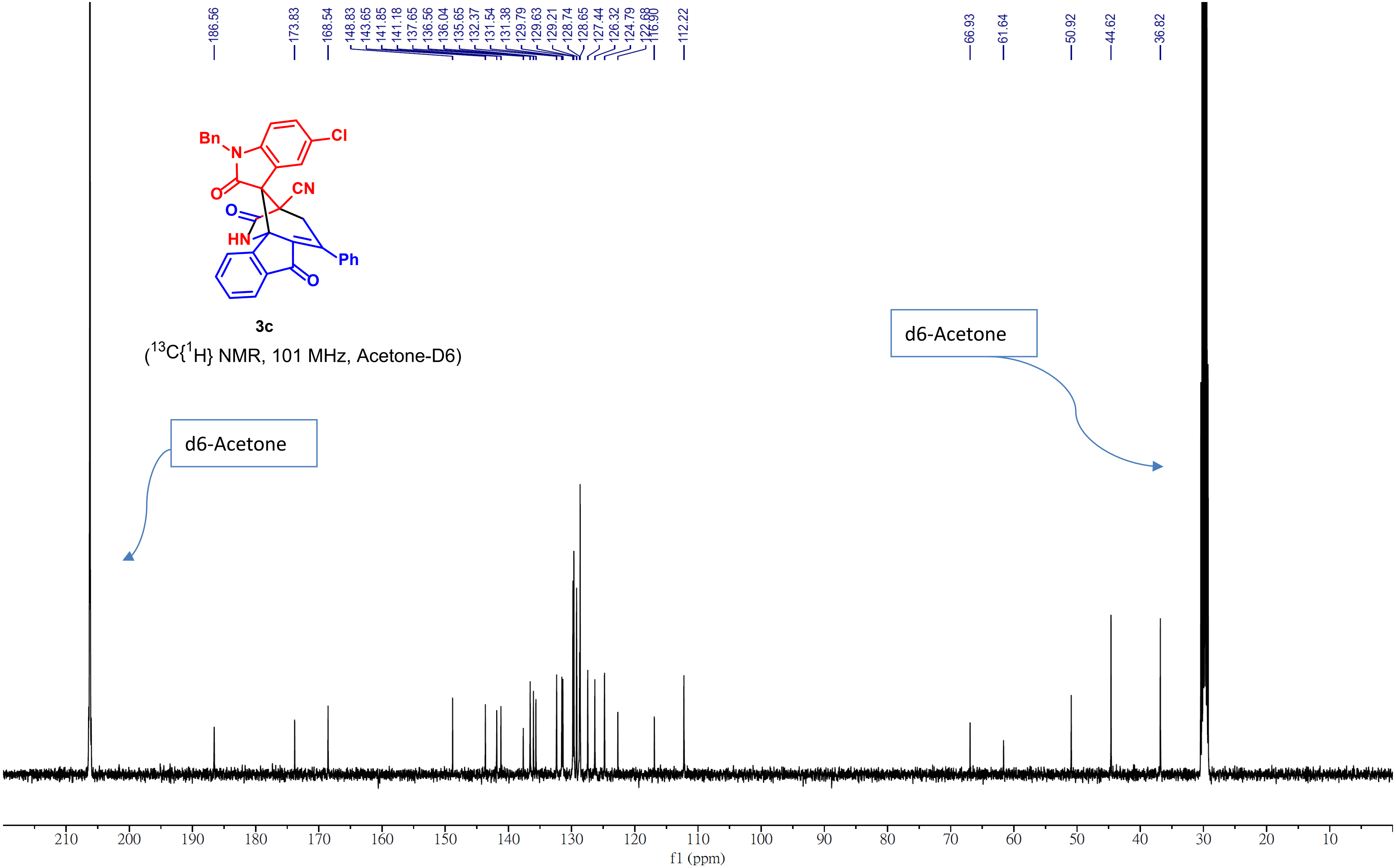

3c

($^{13}C\{^1H\}$ NMR, 101 MHz, Acetone-D6)

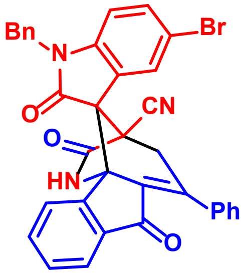

3d

(^{1}H NMR, 400MHz, Acetone-D6)

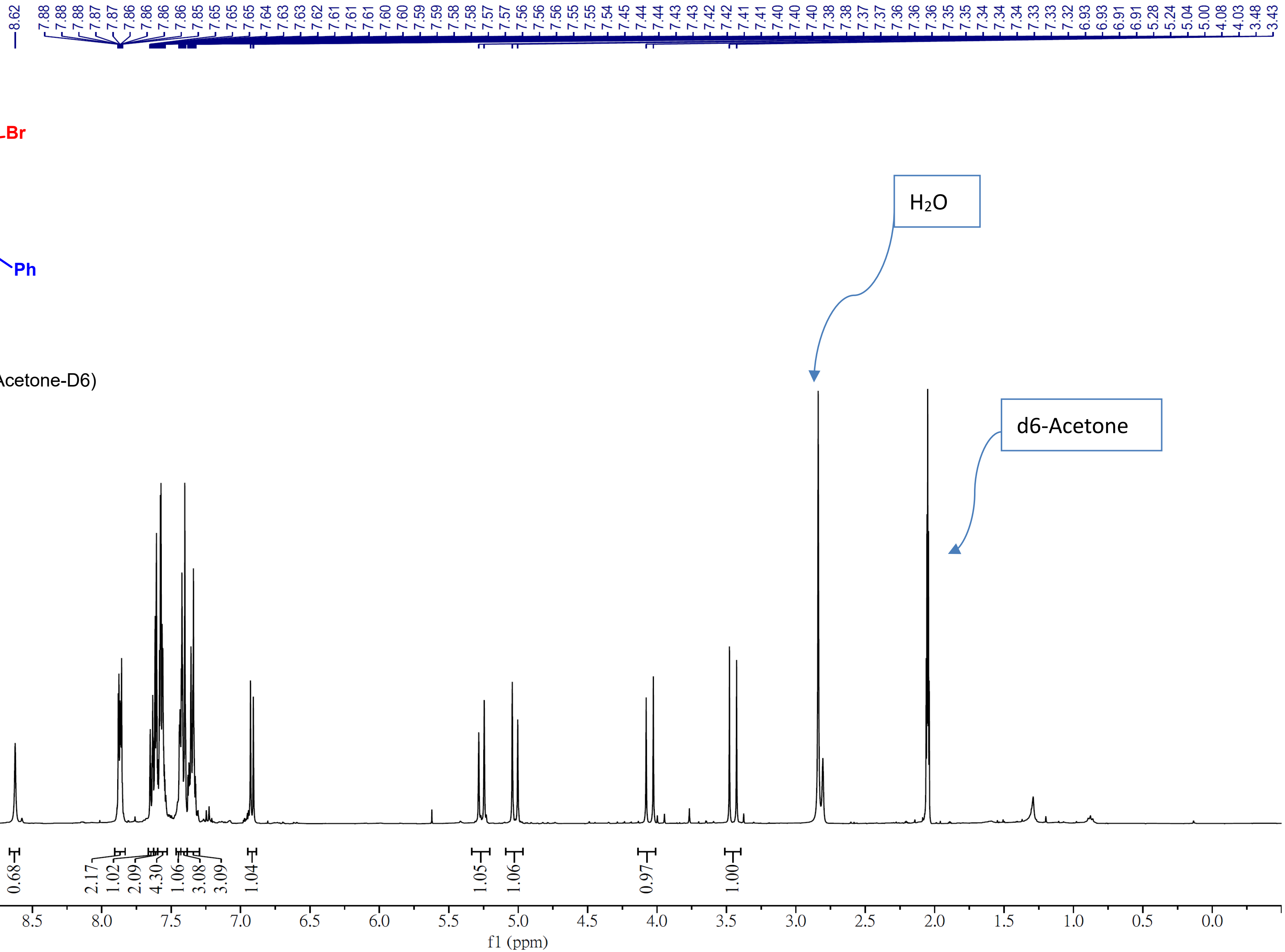

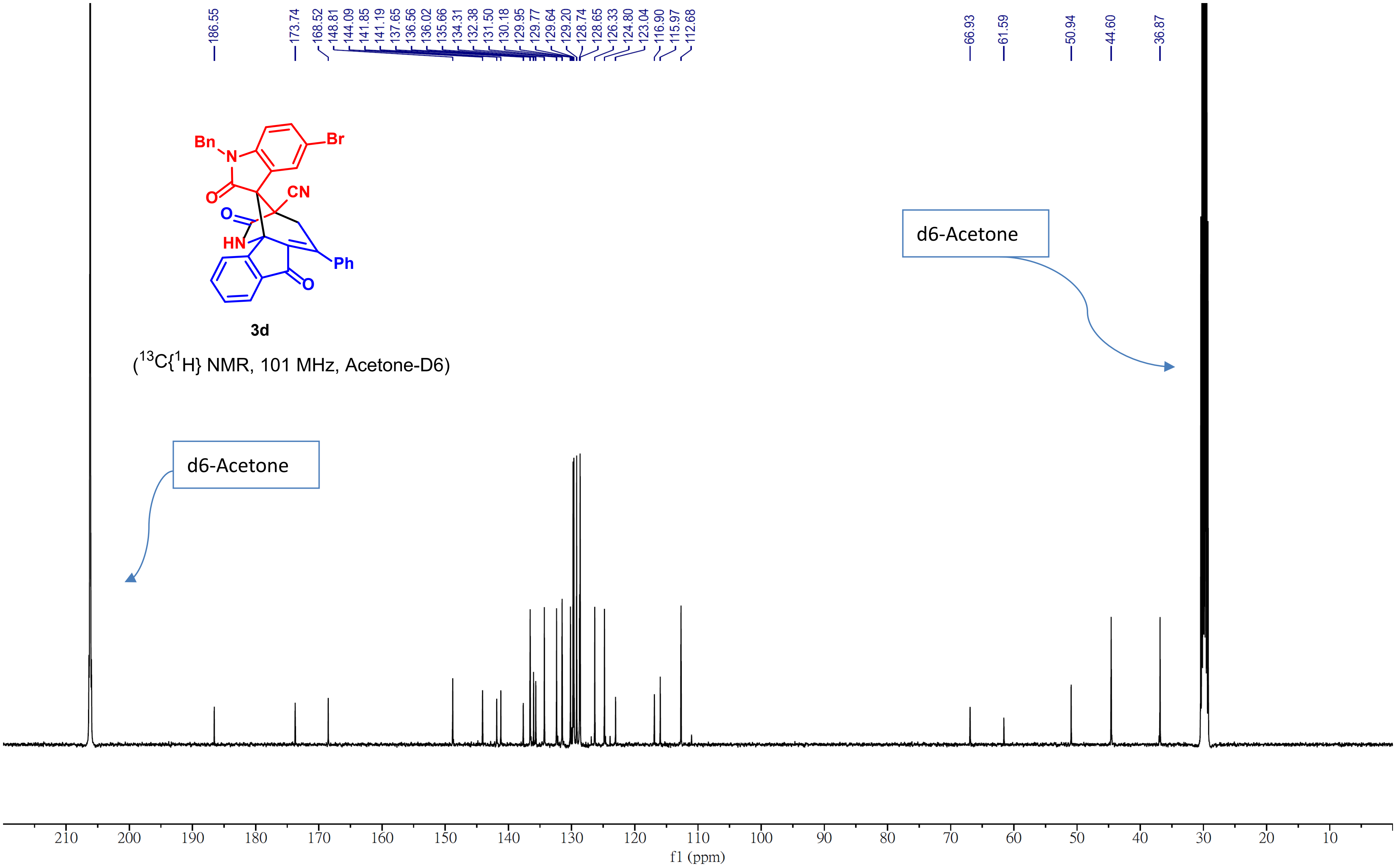

3d

($^{13}C\{^{1}H\}$ NMR, 101 MHz, Acetone-D6)

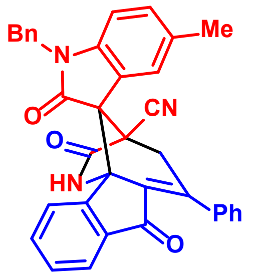

3e

(^{1}H NMR, 400MHz, $CDCl_3$)

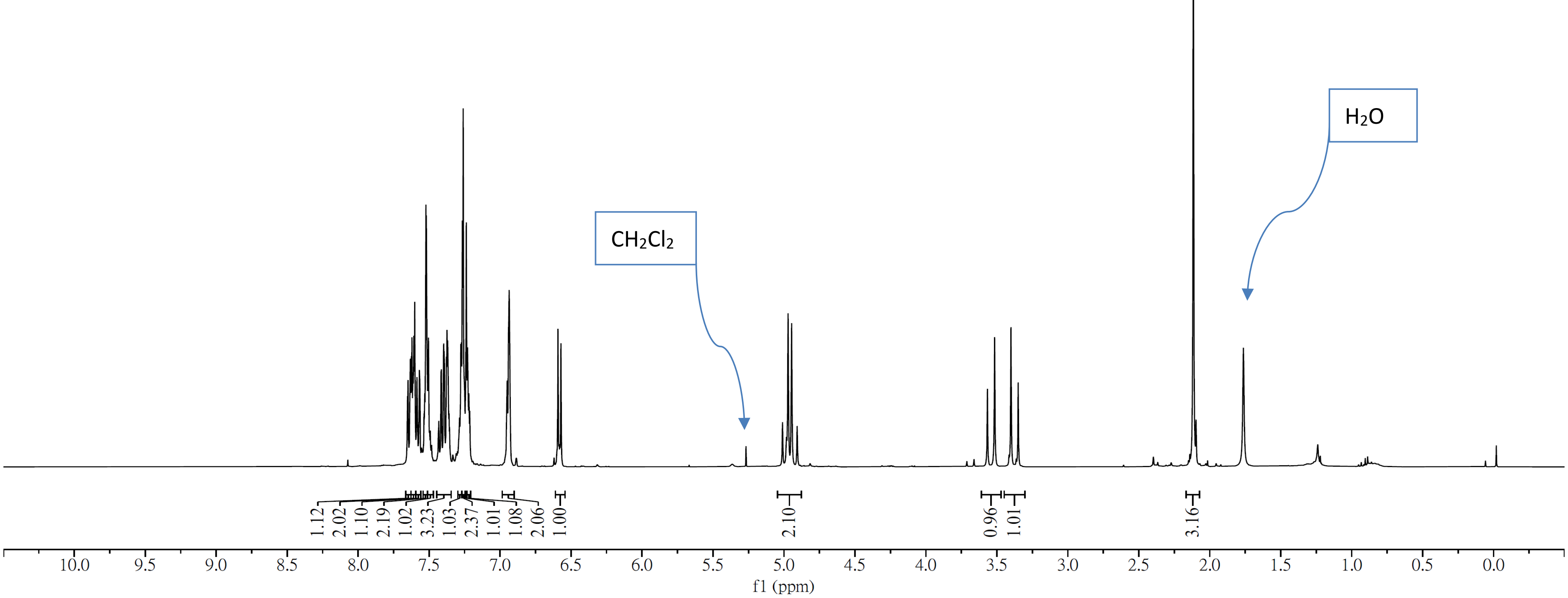

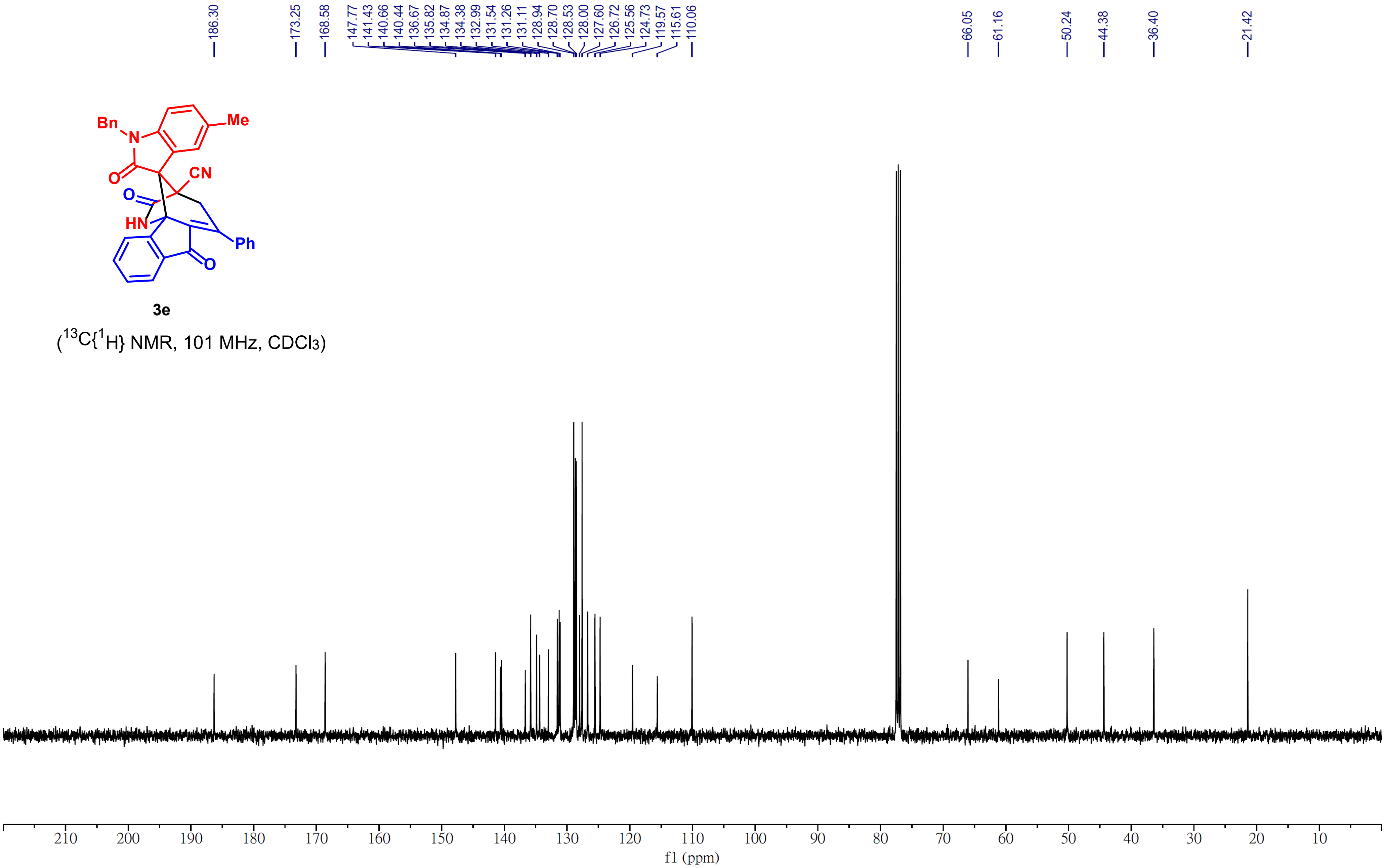
186.30
173.25
168.58
147.77
141.43
140.66
140.44
136.67
135.82
134.87
134.38
132.99
131.54
131.26
131.11
128.94
128.70
128.53
128.00
127.60
126.72
125.56
124.73
119.57
115.61
110.06
66.05
61.16
50.24
44.38
36.40
21.42
Bn
N
Me
O
CN
O
HN
Ph
O
3e
(^{13}C{^{1}H} NMR, 101 MHz, CDCl$_3$)
210
200
190
180
170
160
150
140
130
120
110
100
90
80
70
60
50
40
30
20
10
f1 (ppm)

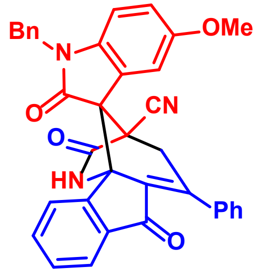

3f

(^{1}H NMR, 400MHz, Acetone-D6)

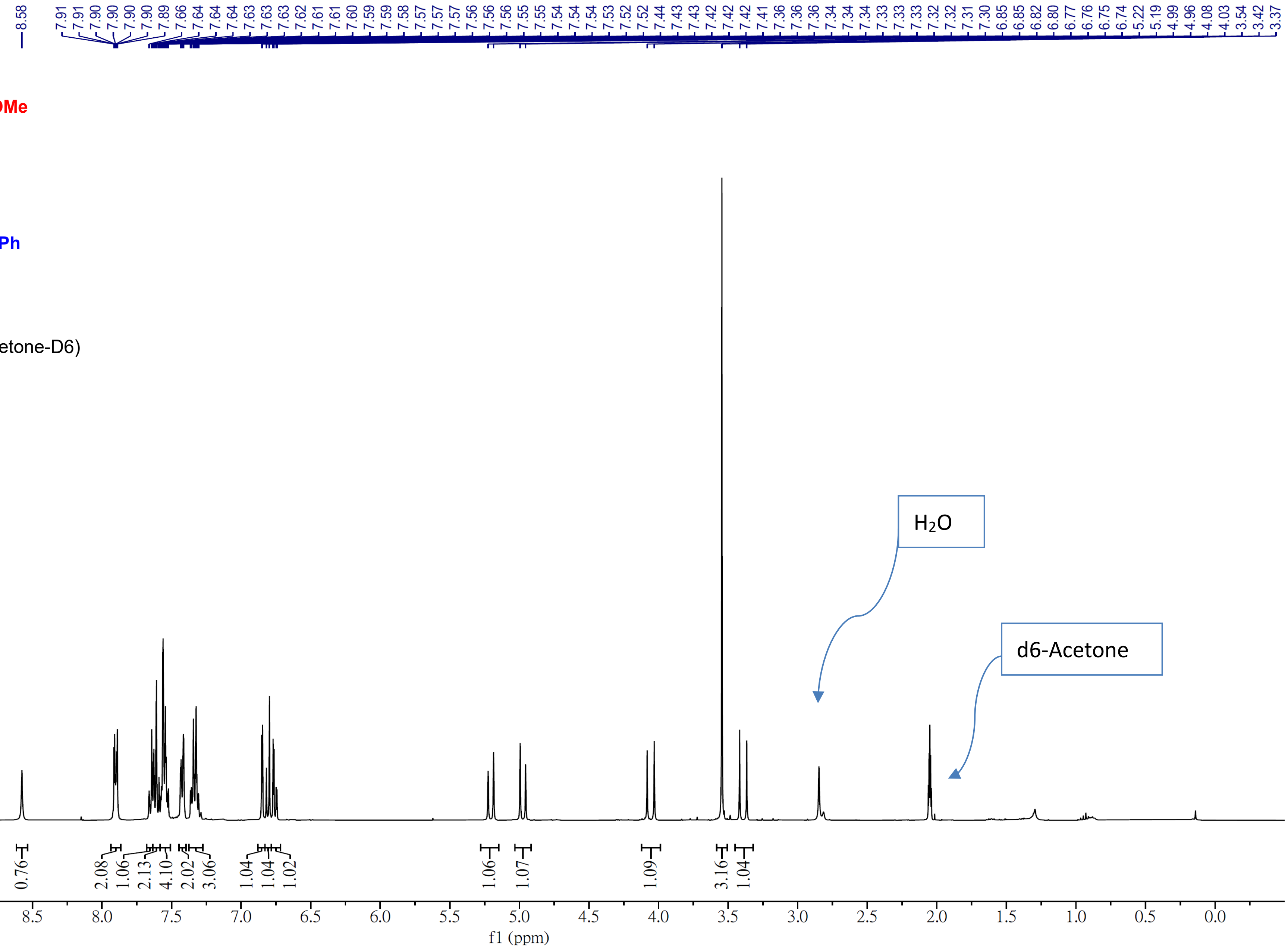

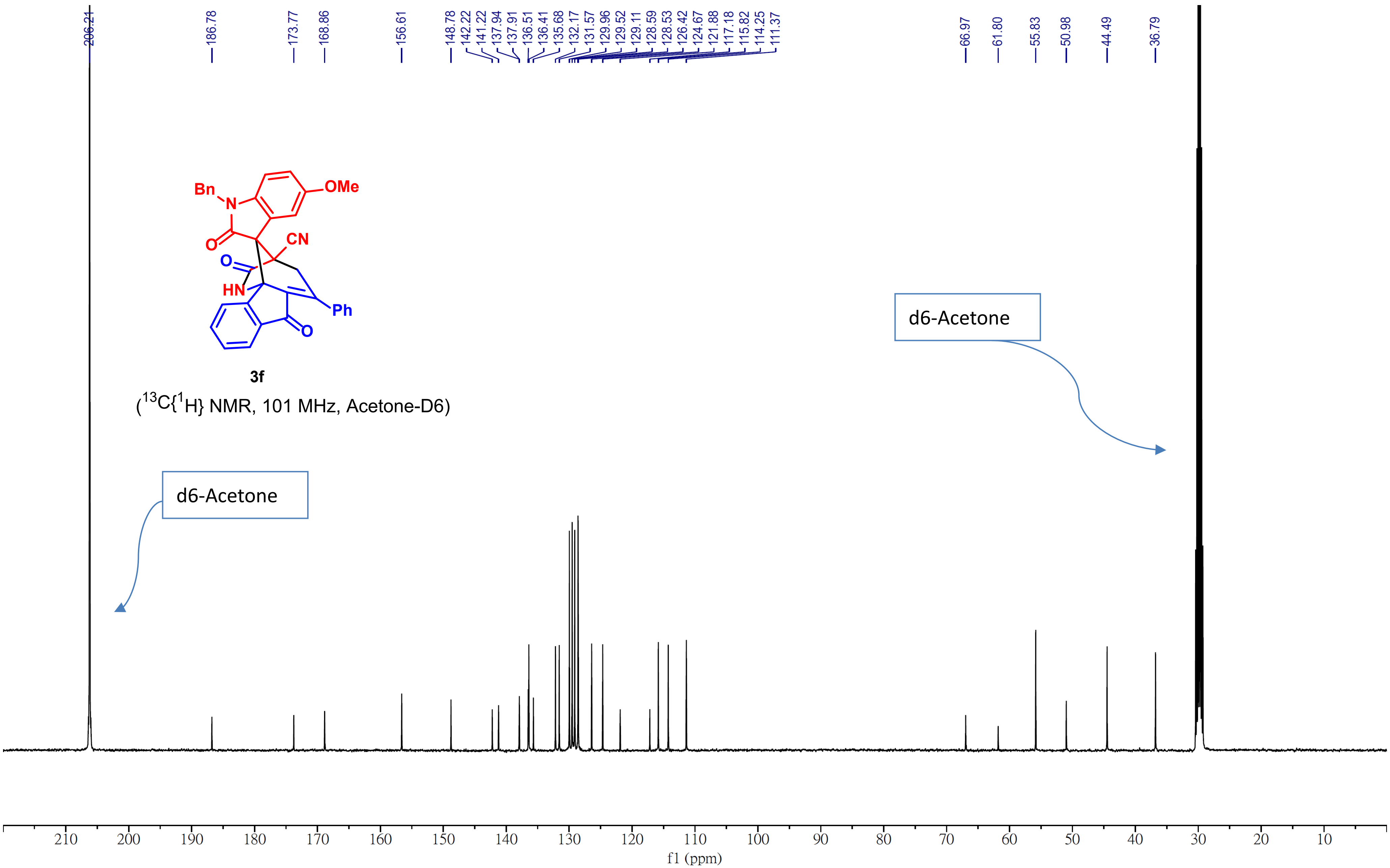
206.21
186.78
173.77
168.86
156.61
148.78
142.22
141.22
137.94
137.91
136.51
136.41
135.68
132.17
131.57
129.96
129.52
129.11
128.59
128.53
126.42
124.67
121.88
117.18
115.82
114.25
111.37
66.97
61.80
55.83
50.98
44.49
36.79
Bn
N
OMe
O
CN
O
HN
Ph
O
3f
($^{13}C\{^{1}H\}$ NMR, 101 MHz, Acetone-D6)
d6-Acetone
d6-Acetone
210 200 190 180 170 160 150 140 130 120 110 100 90 80 70 60 50 40 30 20 10
f1 (ppm)

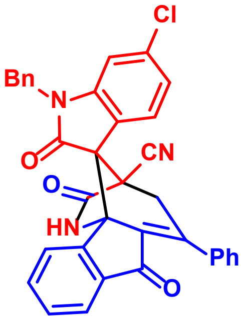

3g

(^{1}H NMR, 400MHz, $CDCl_3$)

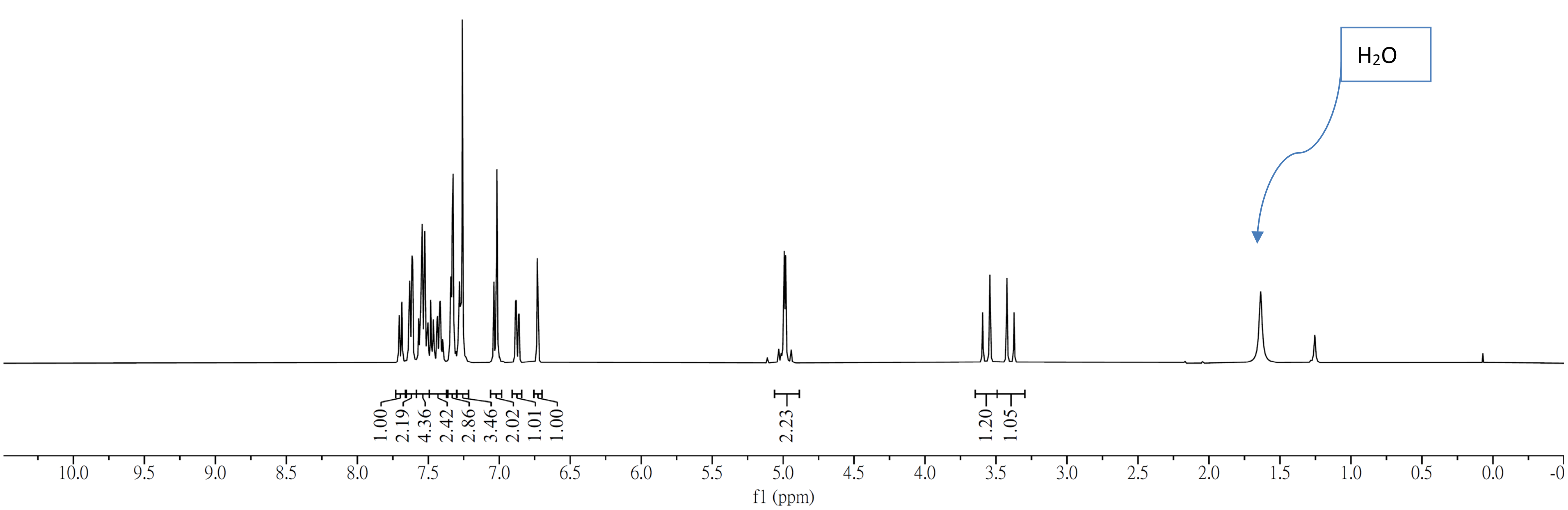

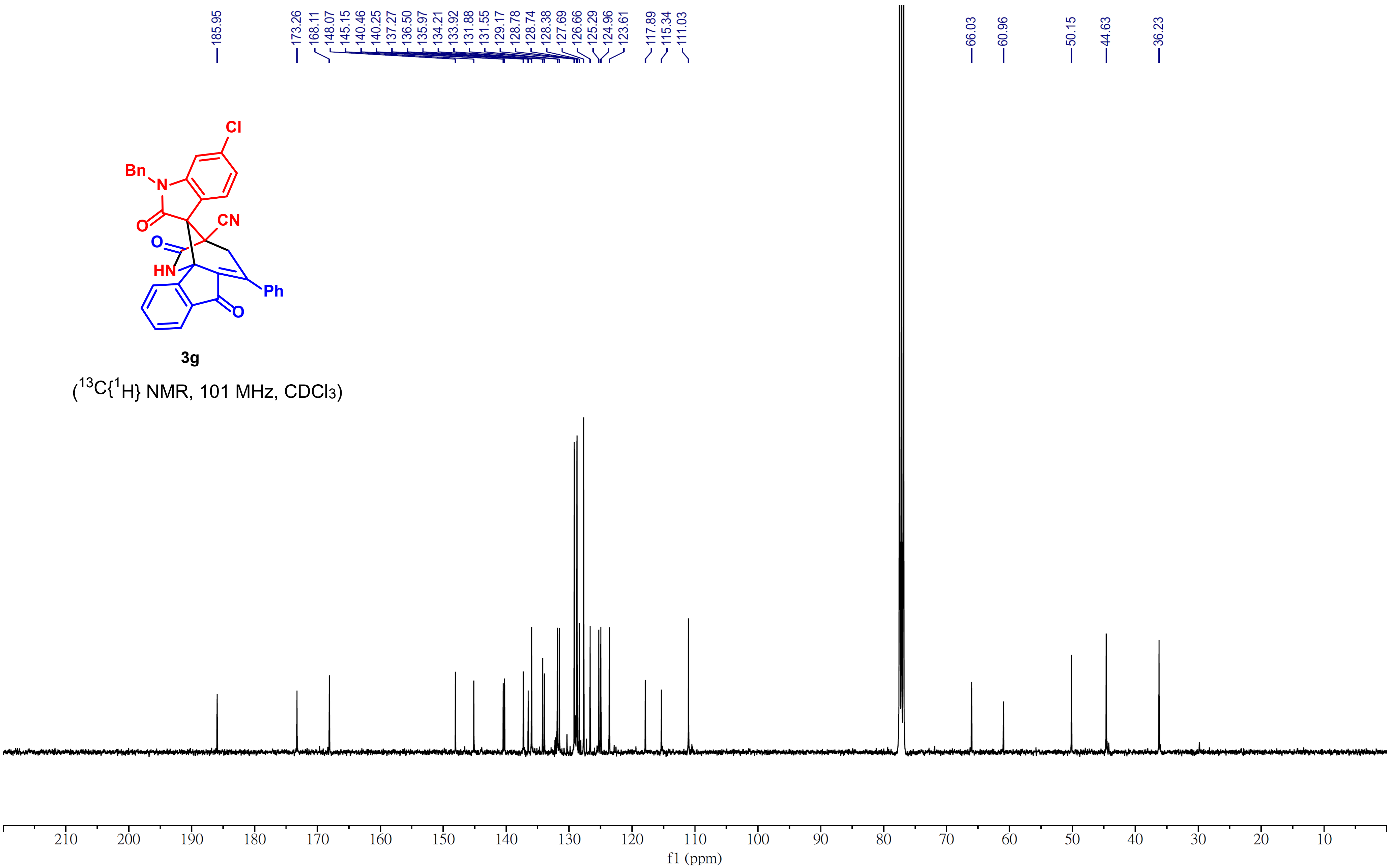
185.95
173.26
168.11
148.07
145.15
140.46
140.25
137.27
136.50
135.97
134.21
133.92
131.88
131.55
129.17
128.78
128.74
128.38
127.69
126.66
125.29
124.96
123.61
117.89
115.34
111.03
66.03
60.96
50.15
44.63
36.23
Cl
Bn
N
O
CN
O
HN
Ph
O
3g
($^{13}C\{^{1}H\}$ NMR, 101 MHz, $CDCl_3$)
210 200 190 180 170 160 150 140 130 120 110 100 90 80 70 60 50 40 30 20 10
f1 (ppm)

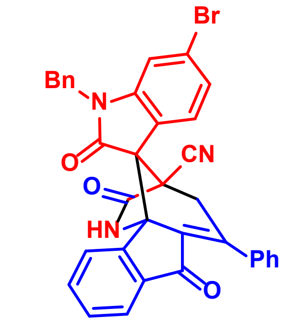

3h

(^{1}H NMR, 400MHz, $CDCl_3$)

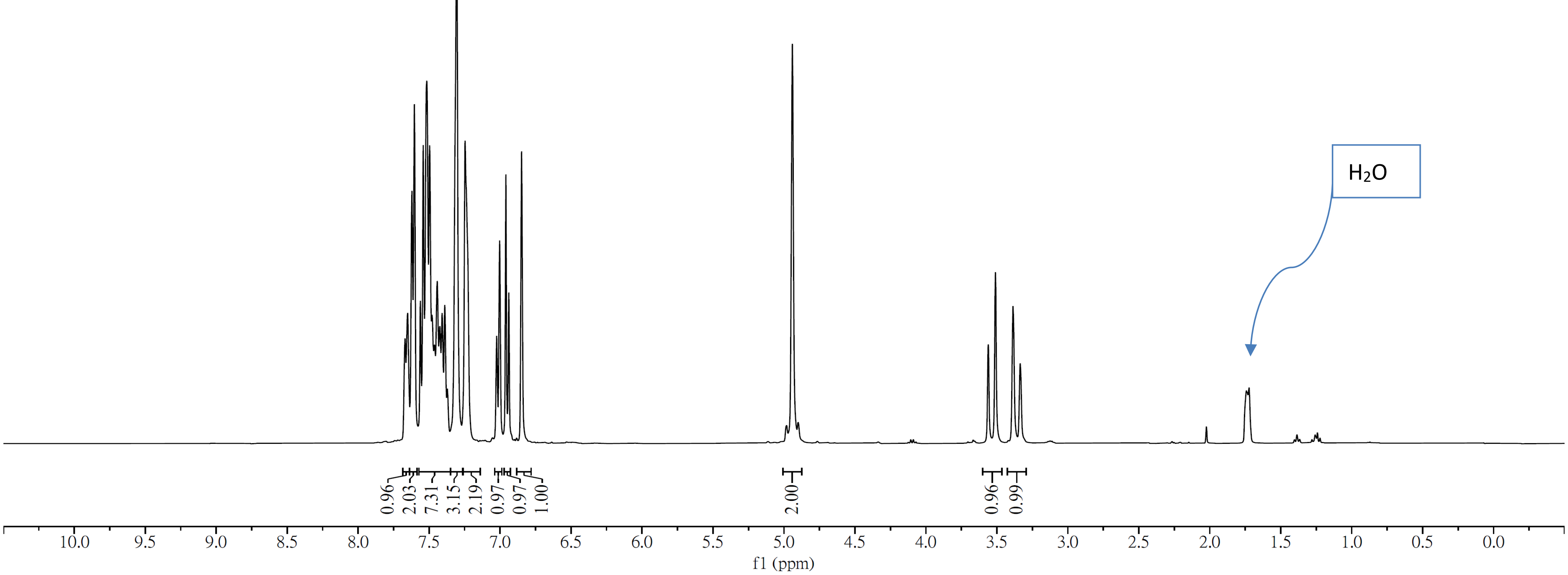

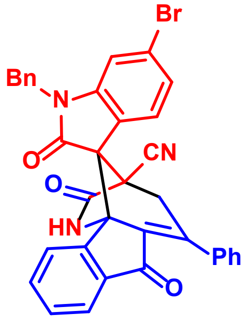

3h

($^{13}C\{^{1}H\}$ NMR, 101 MHz, $CDCl_3$)

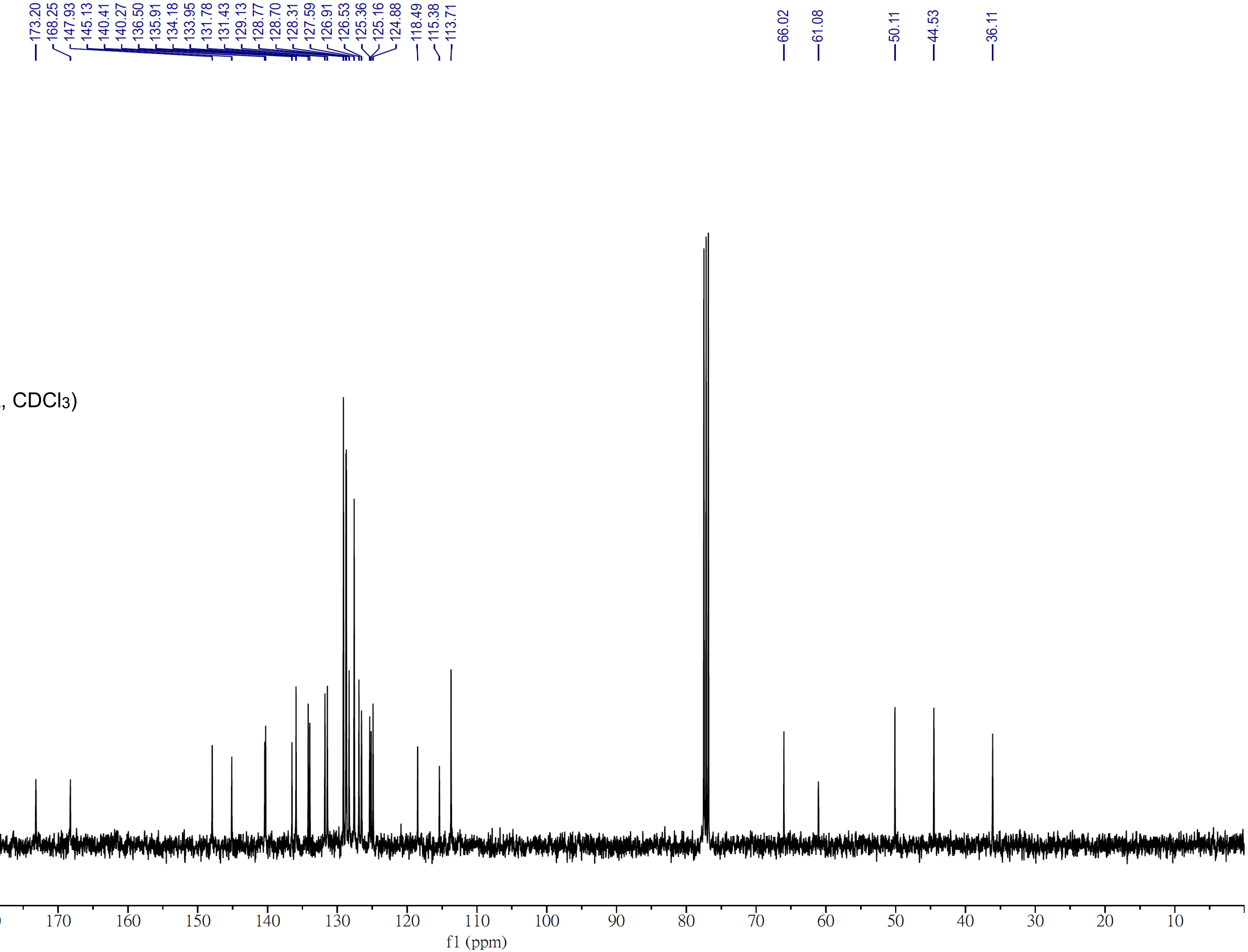

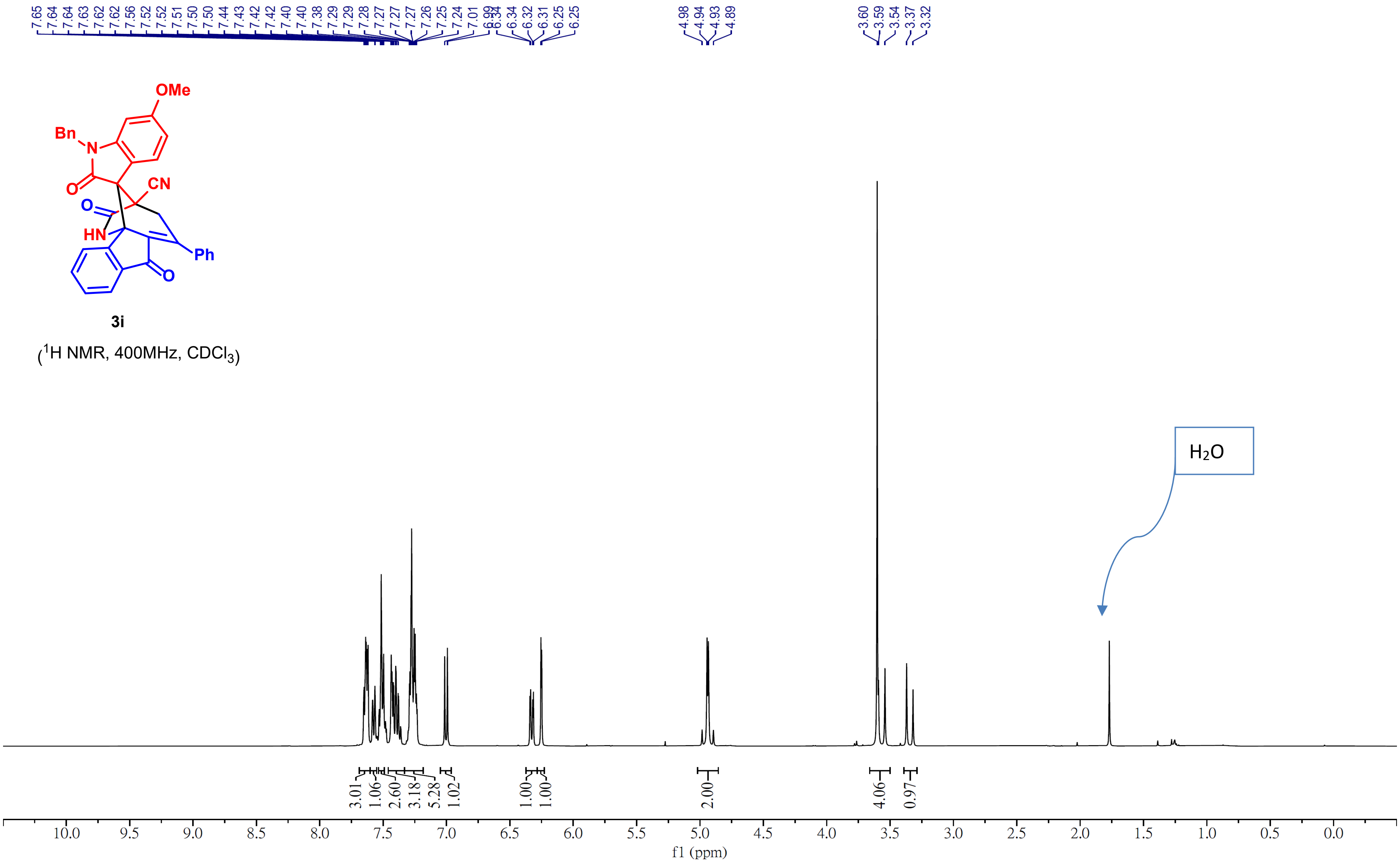

7.65 7.64 7.64 7.63 7.62 7.62 7.56 7.52 7.52 7.51 7.50 7.50 7.44 7.43 7.42 7.42 7.40 7.40 7.38 7.29 7.29 7.28 7.27 7.27 7.26 7.25 7.24 7.01
6.34 6.32 6.31 6.25 6.25
4.98 4.94 4.93 4.89
3.60 3.59 3.54 3.37 3.32
OMe
Bn
N
O
CN
O
HN
Ph
O
3i
(^{1}H NMR, 400MHz, $CDCl_3$)
H_2O
3.01 1.06 2.60 3.18 5.28 1.02 1.00 1.00 2.00 4.06 0.97
10.0 9.5 9.0 8.5 8.0 7.5 7.0 6.5 6.0 5.5 5.0 4.5 4.0 3.5 3.0 2.5 2.0 1.5 1.0 0.5 0.0
f1 (ppm)

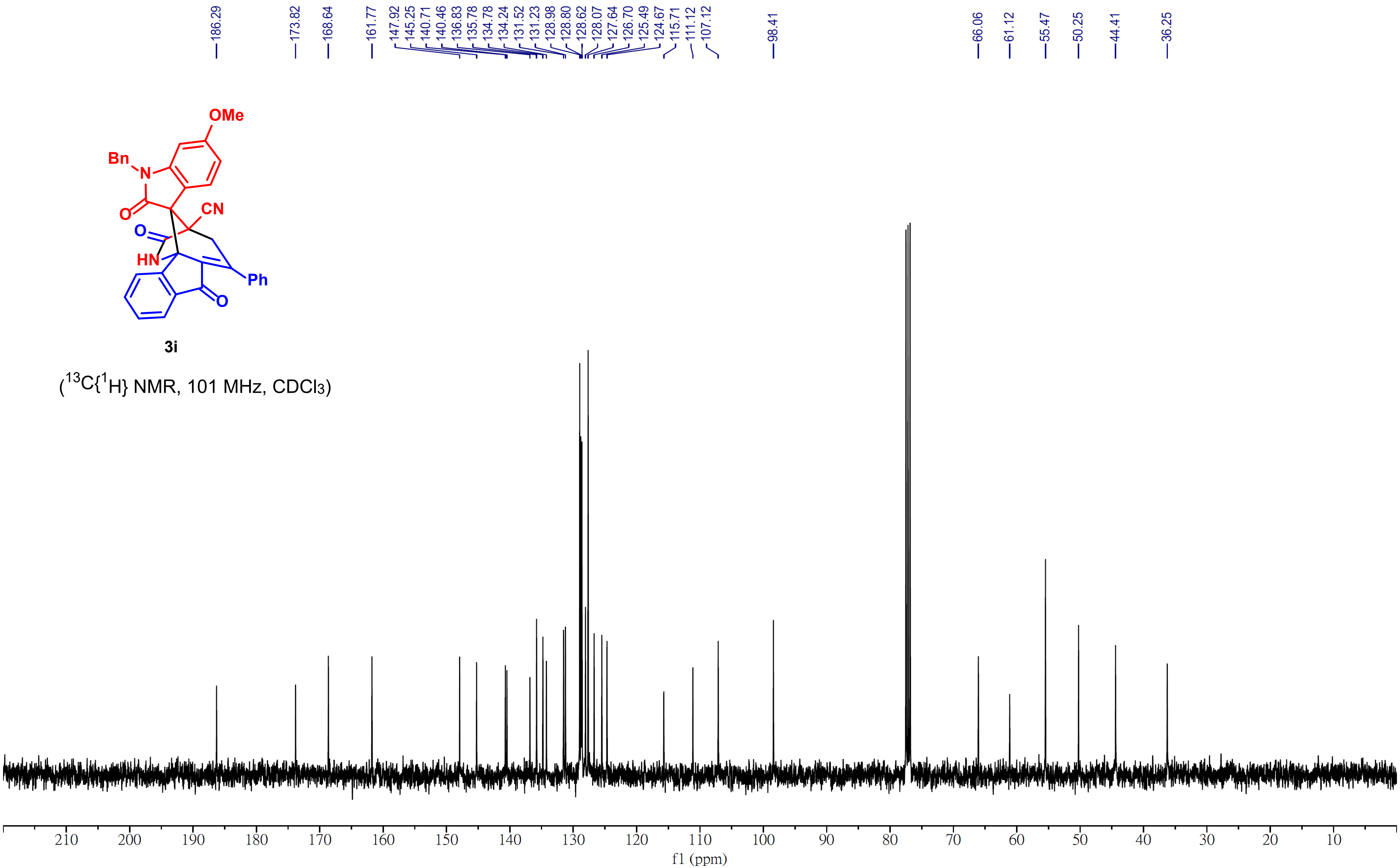
OMe
Bn
N
O
CN
O
HN
Ph
O
3i
(13C{1H} NMR, 101 MHz, CDCl3)
186.29
173.82
168.64
161.77
147.92
145.25
140.71
140.46
136.83
135.78
134.78
134.24
131.52
131.23
128.98
128.80
128.62
128.07
127.64
126.70
125.49
124.67
115.71
111.12
107.12
98.41
66.06
61.12
55.47
50.25
44.41
36.25
210
200
190
180
170
160
150
140
130
120
110
100
90
80
70
60
50
40
30
20
10
f1 (ppm)

7.64 7.64 7.63 7.63 7.62 7.62 7.61 7.61 7.52 7.52 7.52 7.50 7.50 7.49 7.47 7.43 7.42 7.42 7.41 7.40 7.39 7.38 7.38 7.34 7.33 7.31 7.31 7.30 7.29 7.28 7.27 7.25 6.99 6.97 6.96 6.94 6.93 6.93 6.91 6.86 6.84 6.84 6.82 6.82 6.80

5.21 5.21 5.17 5.17 5.09 5.09 5.06 5.05

3.59 3.54 3.54 3.40 3.39 3.35 3.34

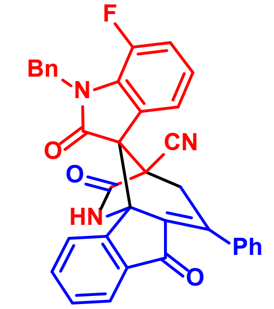

3j

(^{1}H NMR, 400MHz, $CDCl_3$)

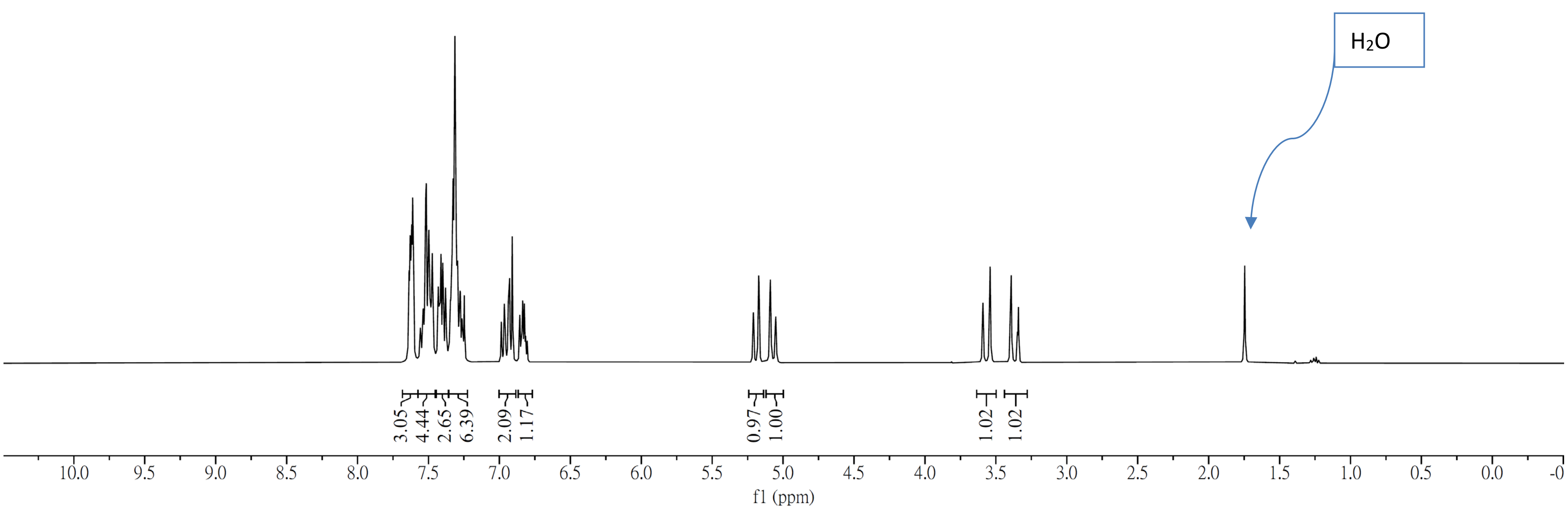

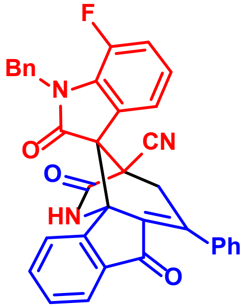

3j

($^{13}C\{^{1}H\}$ NMR, 101 MHz, $CDCl_3$)

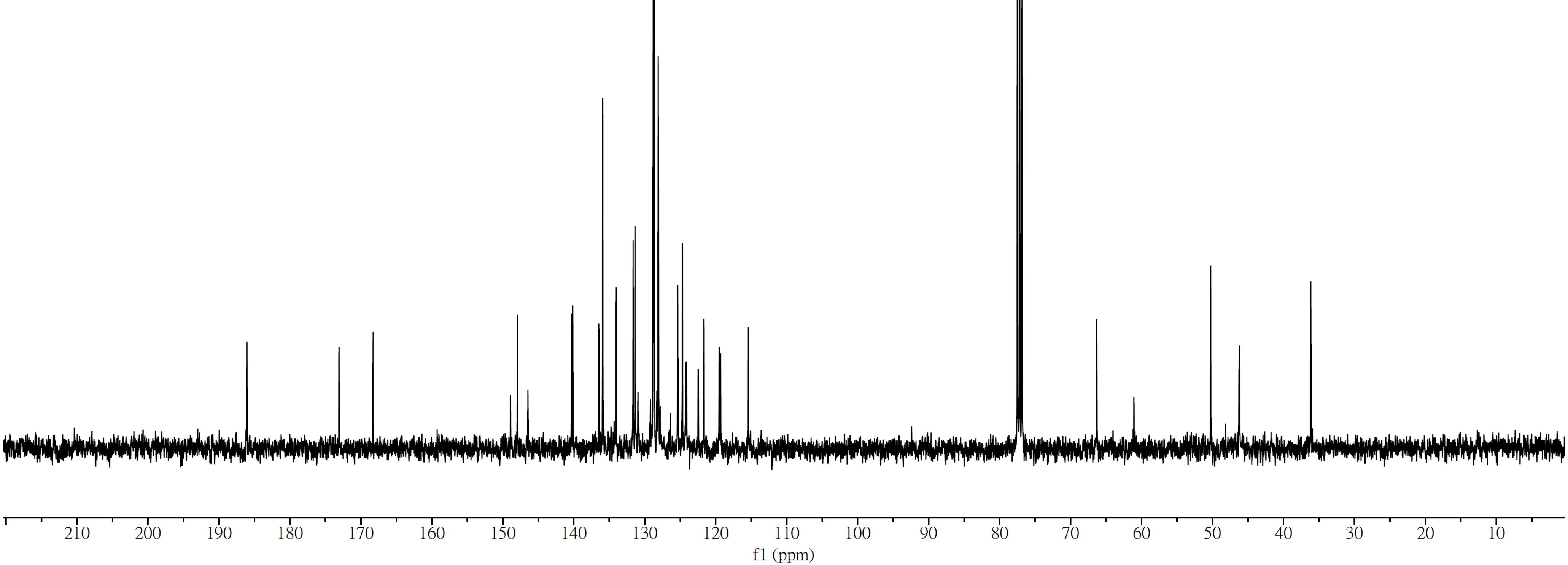

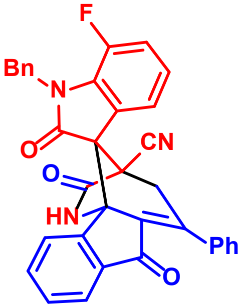

3j

(19F NMR, 376MHz, $CDCl_3$)

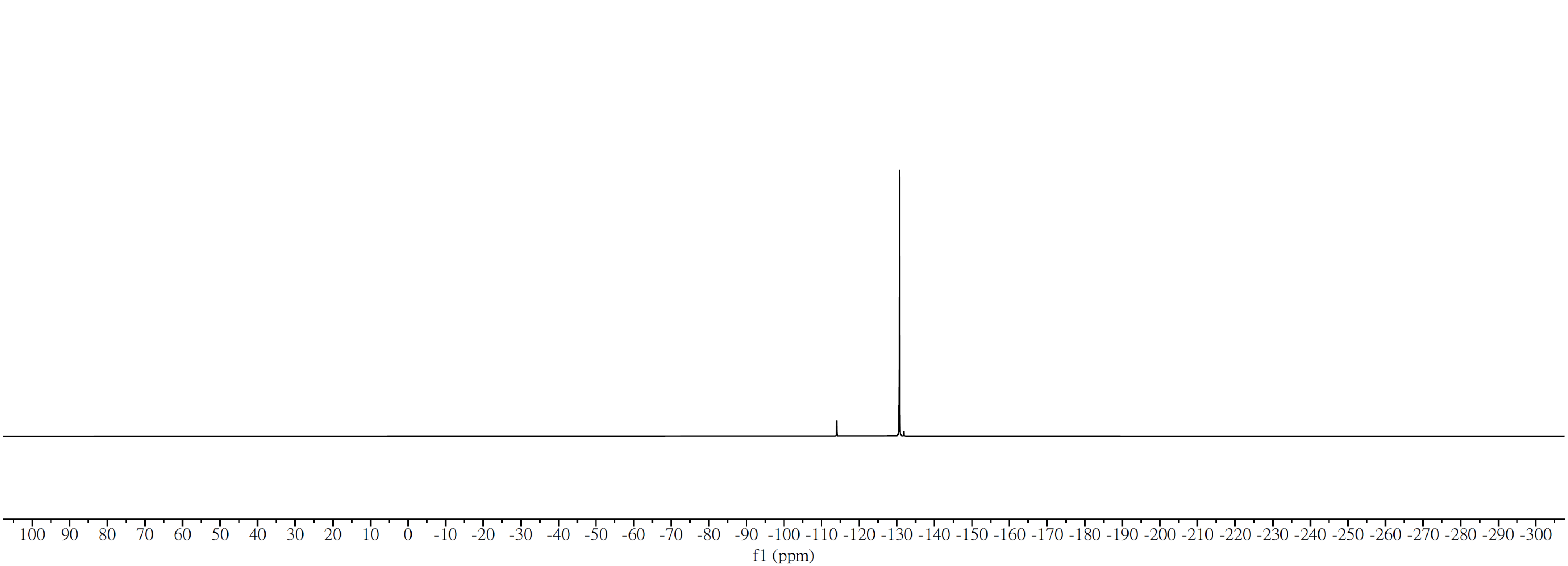

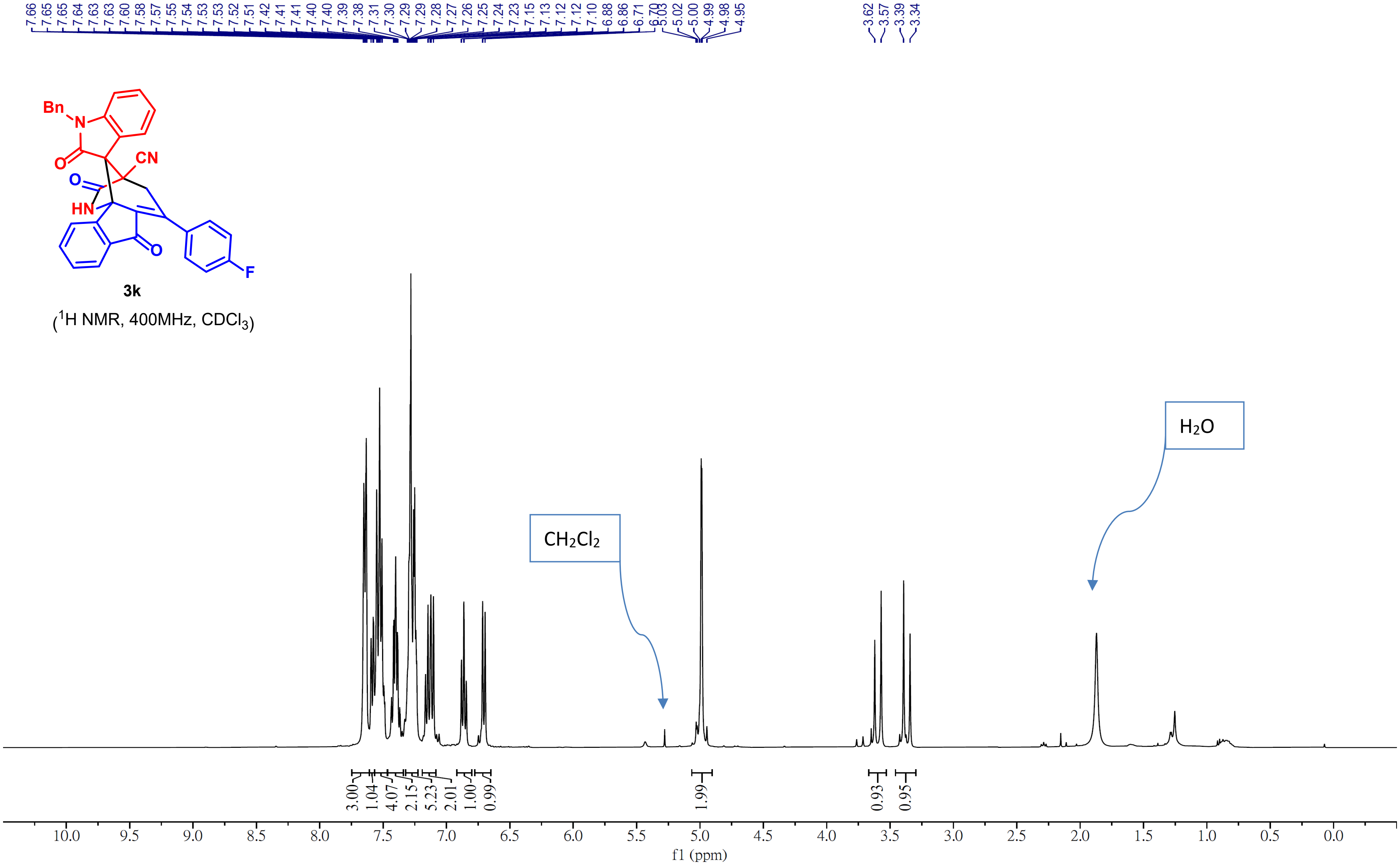
Bn
N
O
CN
O
HN
O
F
3k
(1H NMR, 400MHz, CDCl3)
CH2Cl2
H2O
f1 (ppm)

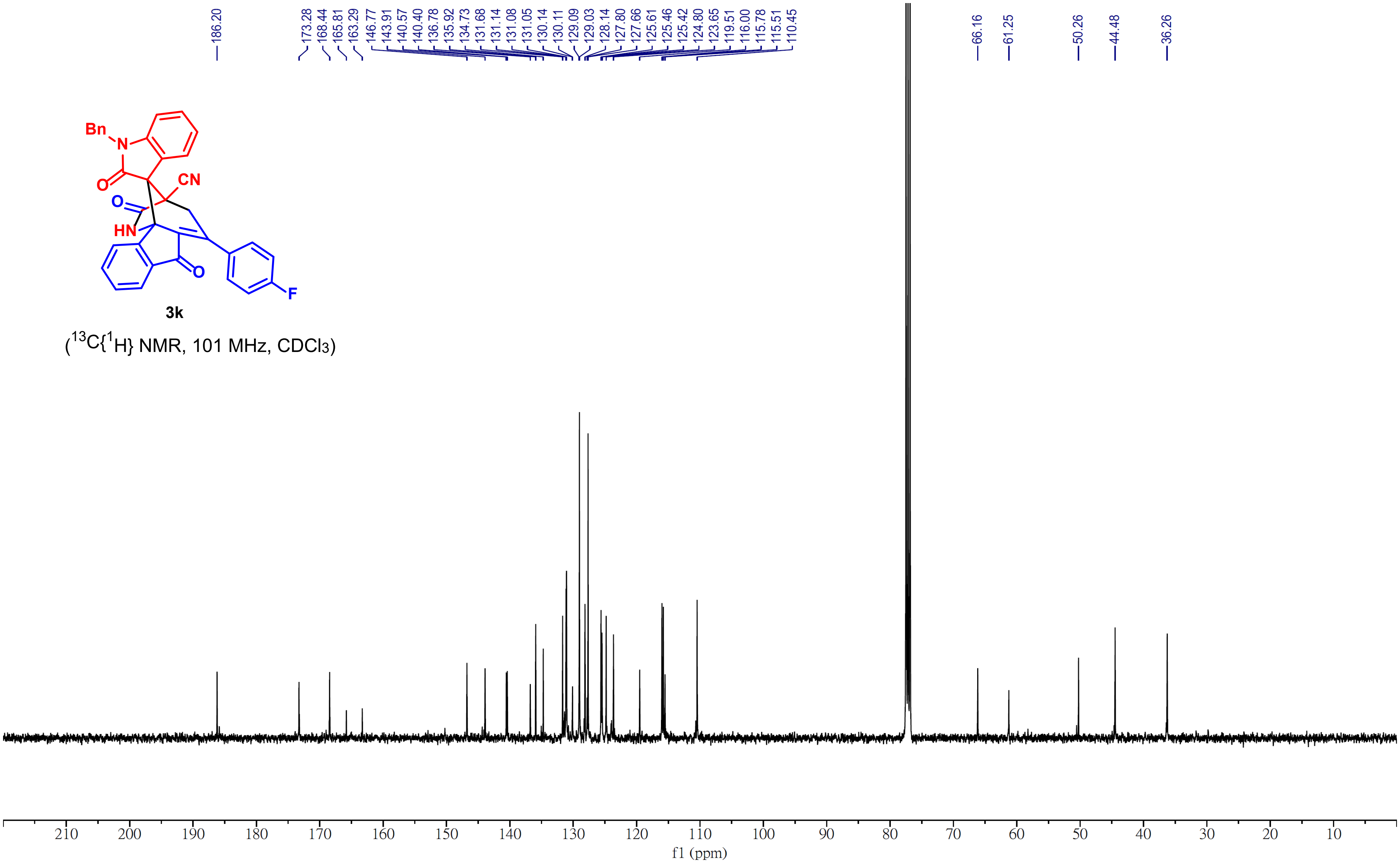

3k

($^{13}C\{^1H\}$ NMR, 101 MHz, $CDCl_3$)

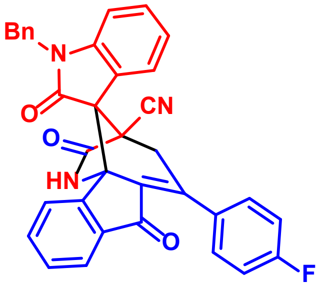

3k

(^{19}F NMR, 376MHz, $CDCl_3$)

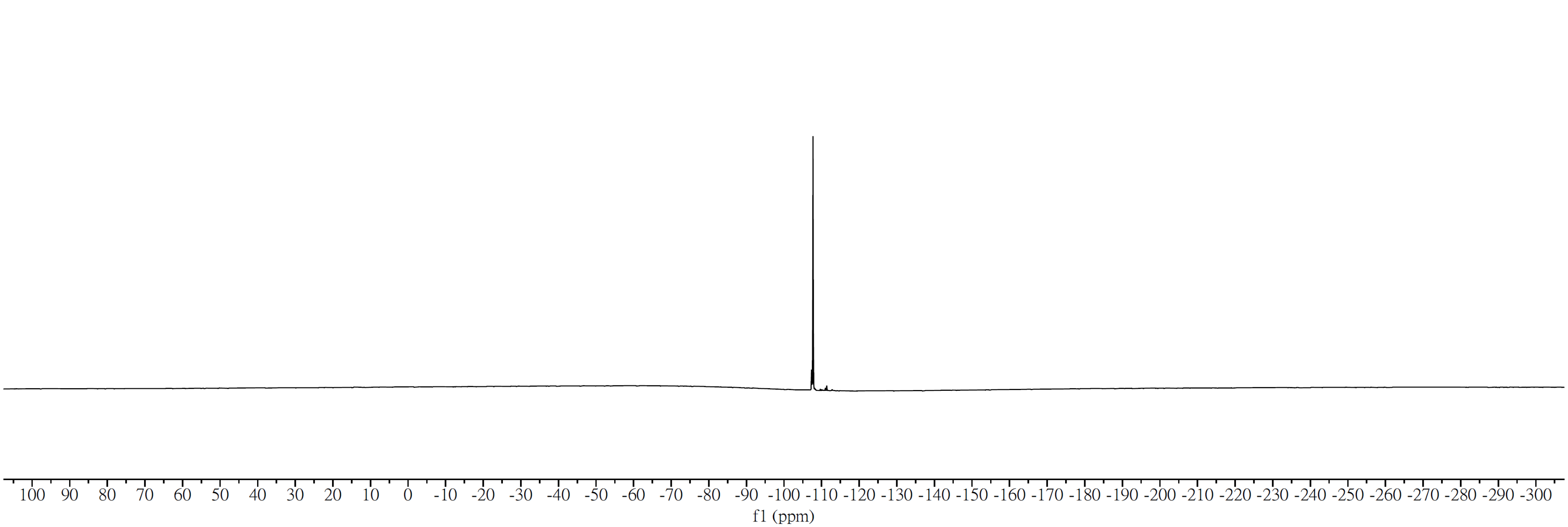

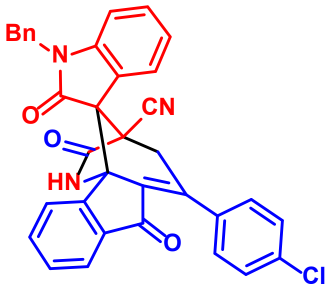

3l

(^{1}H NMR, 400MHz, $CDCl_3$)

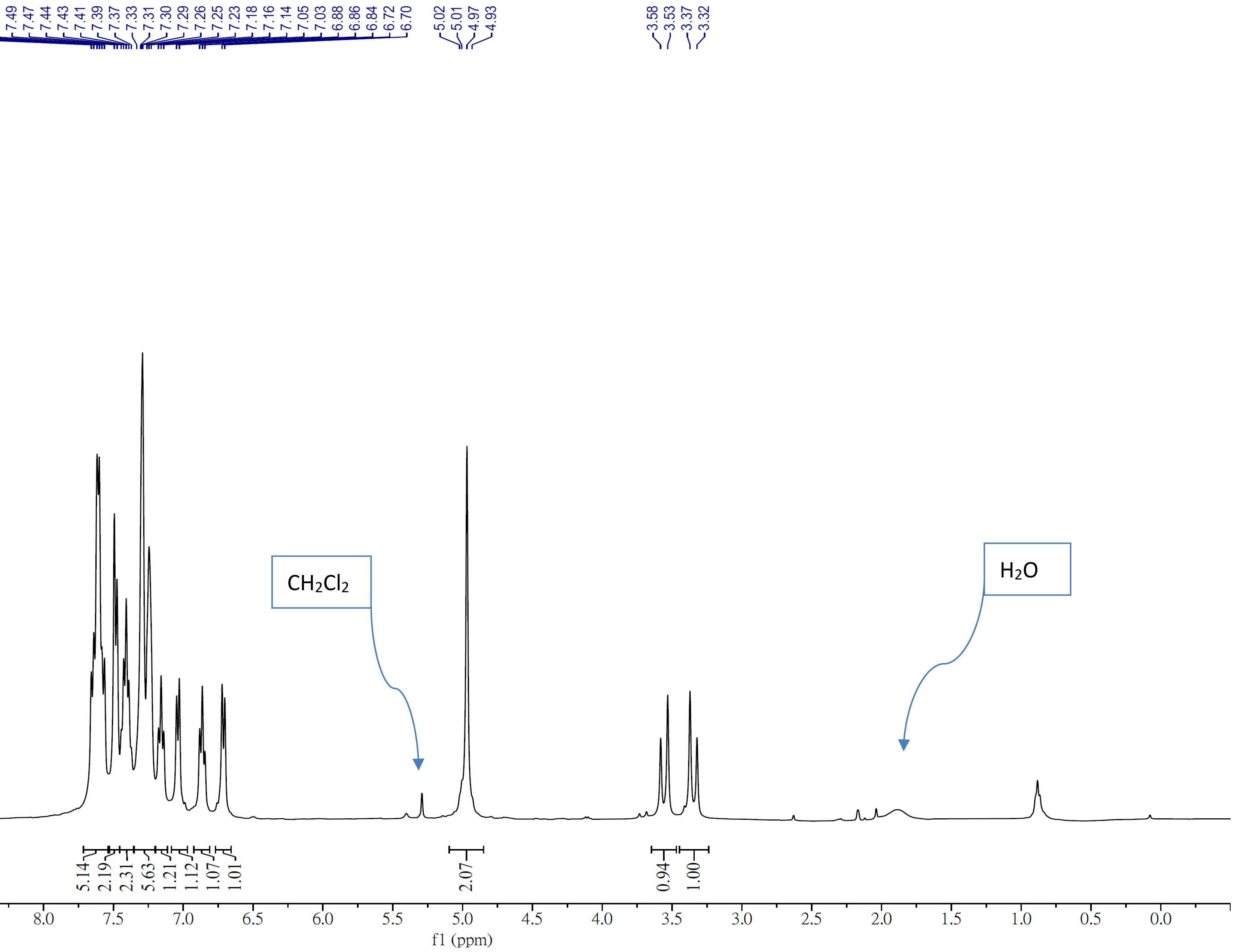

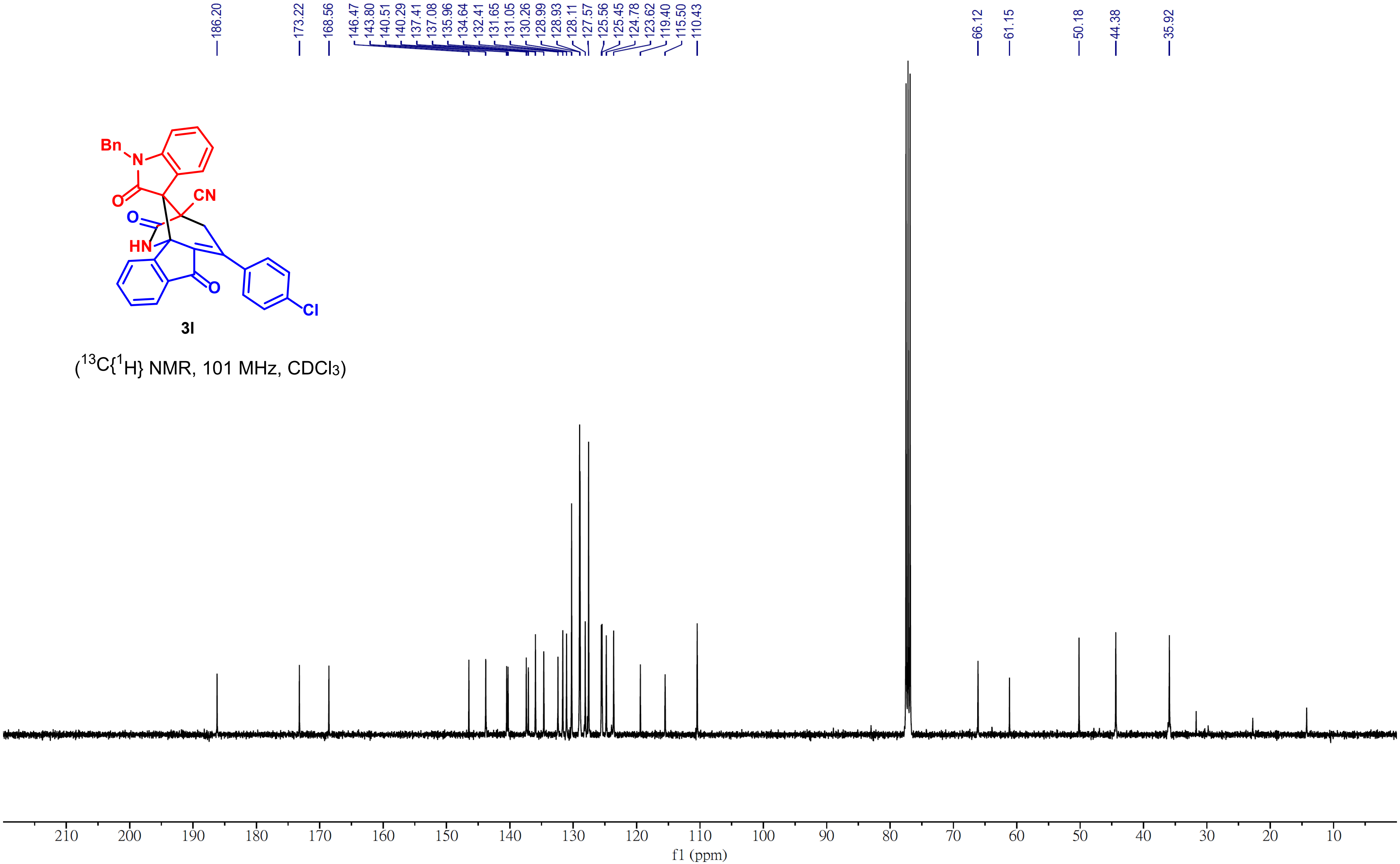

3l

($^{13}C\{^{1}H\}$ NMR, 101 MHz, $CDCl_3$)

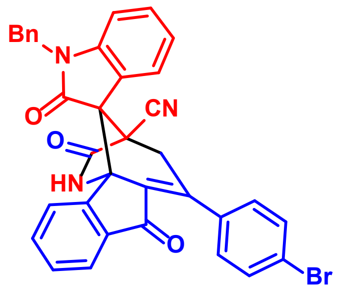

3m

(^{1}H NMR, 400MHz, $CDCl_3$)

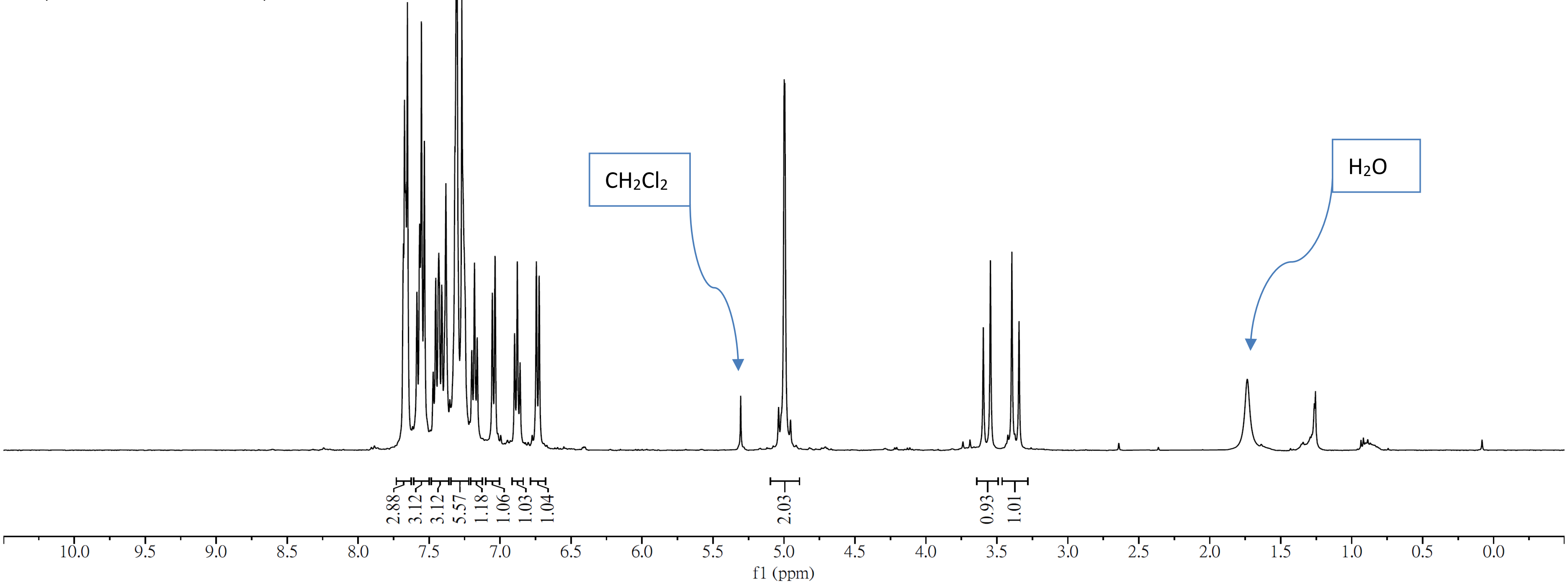

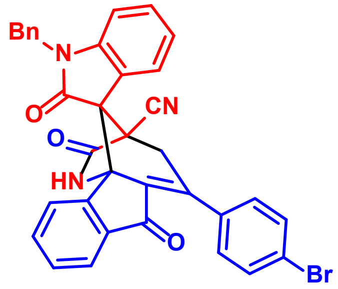

3m

($^{13}C\{^{1}H\}$ NMR, 101 MHz, $CDCl_3$)

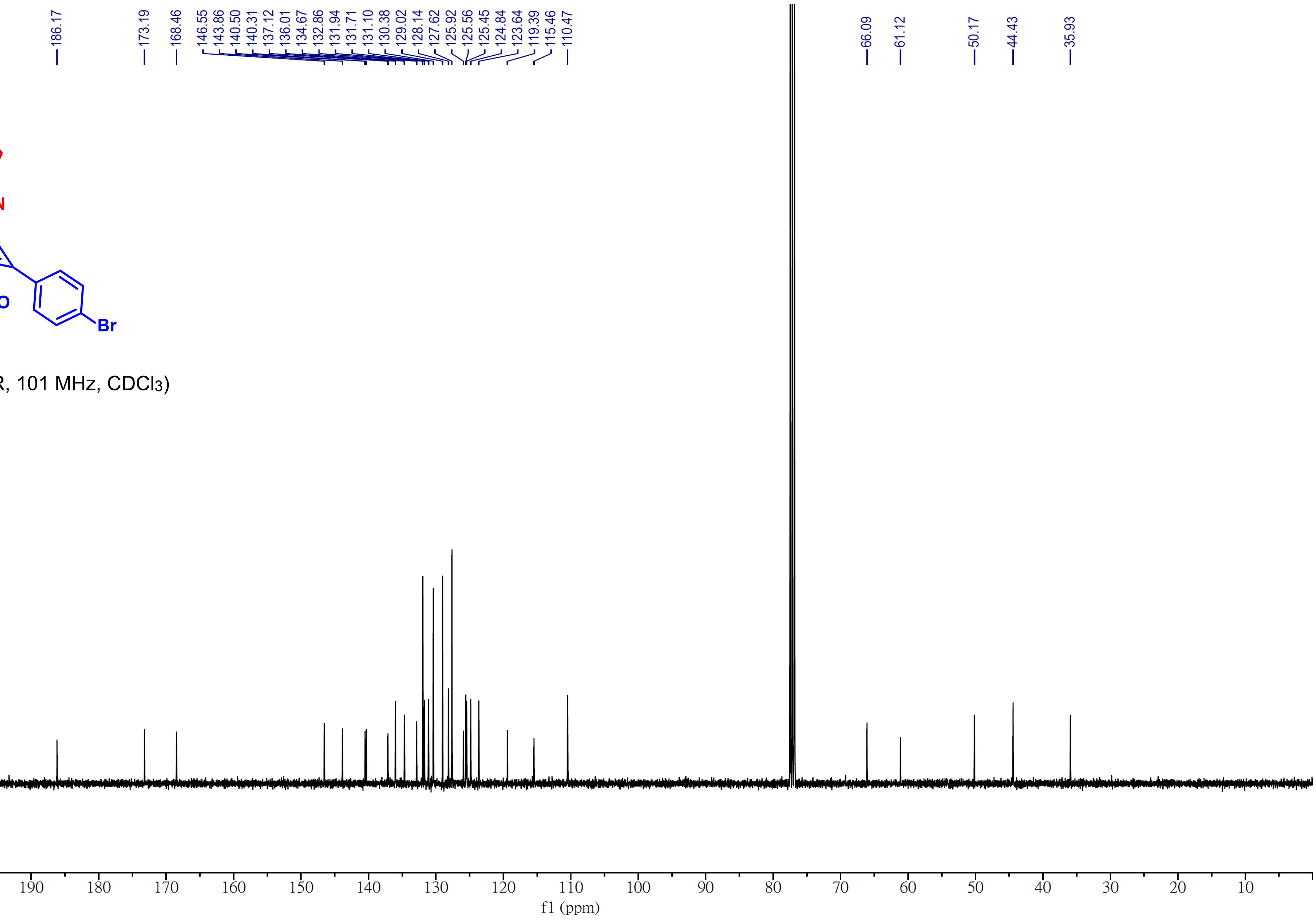

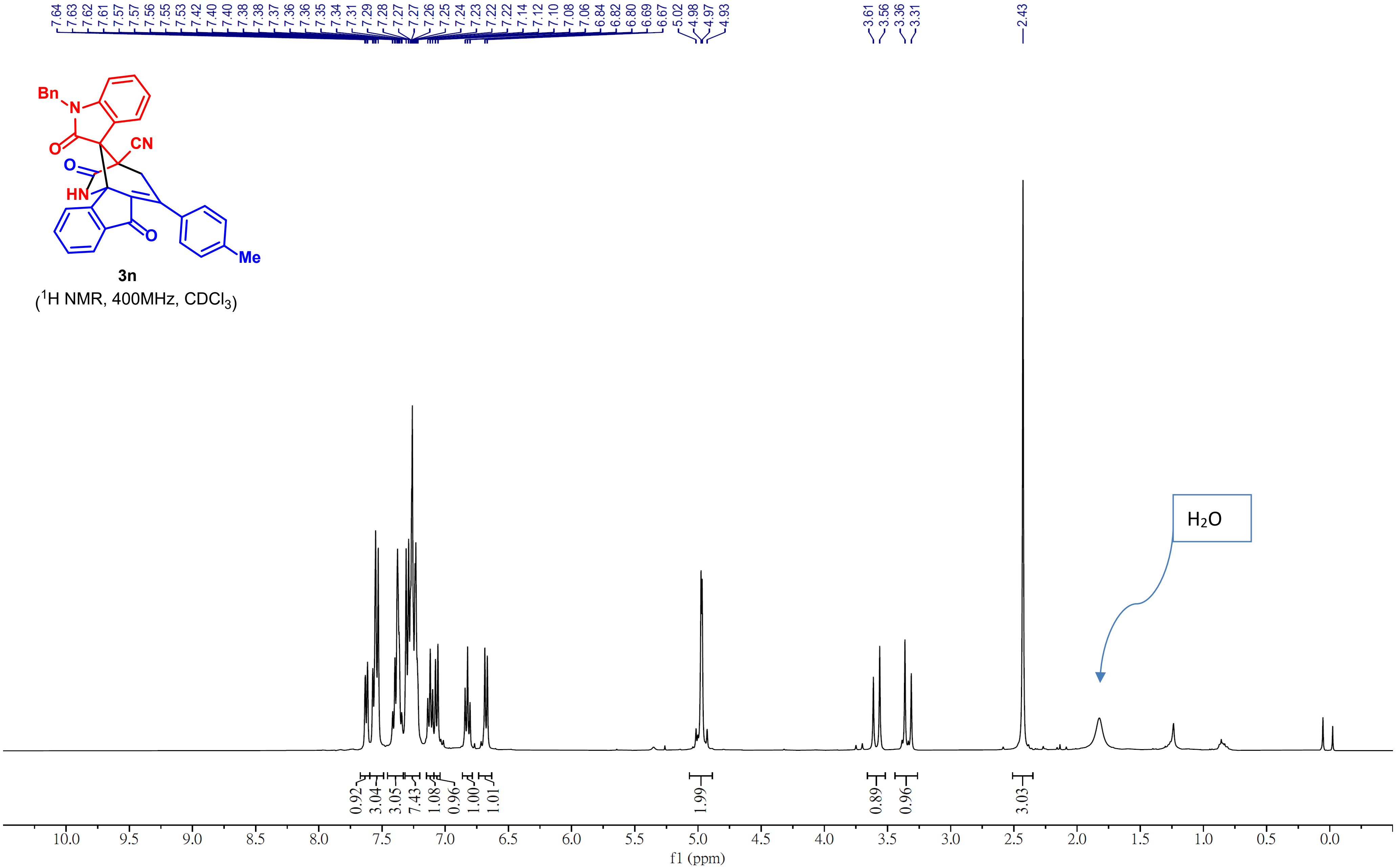
7.64 7.63 7.62 7.61 7.57 7.57 7.56 7.55 7.53 7.42 7.40 7.40 7.38 7.38 7.37 7.36 7.36 7.35 7.34 7.31 7.29 7.28 7.27 7.27 7.26 7.25 7.24 7.23 7.22 7.22 7.14 7.12 7.10 7.08 7.06 6.84 6.82 6.80 6.69 6.67 5.02 4.98 4.97 4.93
3.61 3.56 3.36 3.31
2.43
Bn
N
O
CN
O
HN
O
Me
3n
(1H NMR, 400MHz, CDCl3)
H2O
0.92 3.04 3.05 7.43 1.08 0.96 1.00 1.01
1.99
0.89 0.96
3.03
10.0 9.5 9.0 8.5 8.0 7.5 7.0 6.5 6.0 5.5 5.0 4.5 4.0 3.5 3.0 2.5 2.0 1.5 1.0 0.5 0.0
f1 (ppm)

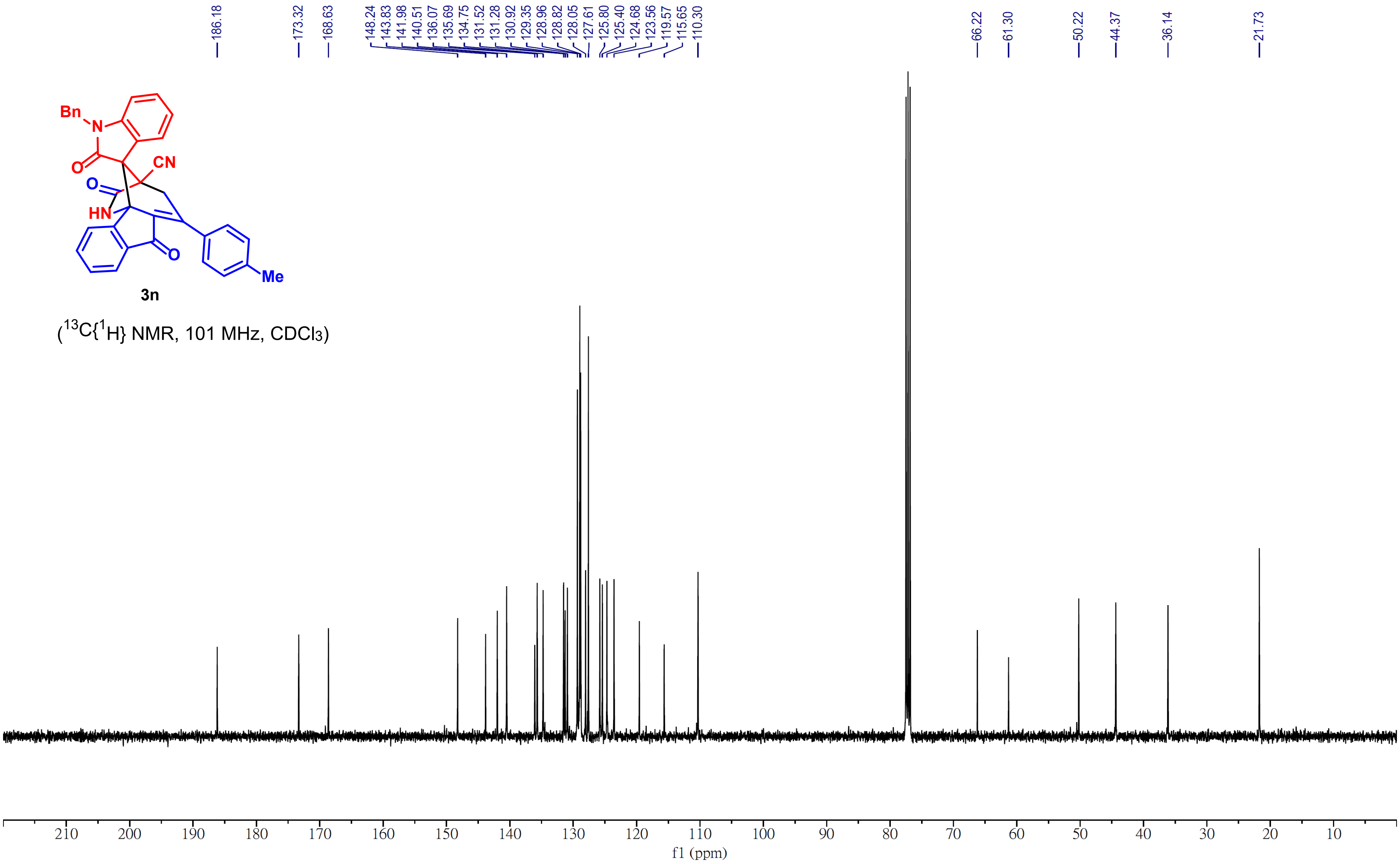
186.18
173.32
168.63
148.24
143.83
141.98
140.51
136.07
135.69
134.75
131.52
131.28
130.92
129.35
128.96
128.82
128.05
127.61
125.80
125.40
124.68
123.56
119.57
115.65
110.30
66.22
61.30
50.22
44.37
36.14
21.73
Bn
N
O
CN
O
HN
O
Me
3n
(^{13}C{^{1}H} NMR, 101 MHz, $CDCl_3$)
210
200
190
180
170
160
150
140
130
120
110
100
90
80
70
60
50
40
30
20
10
f1 (ppm)

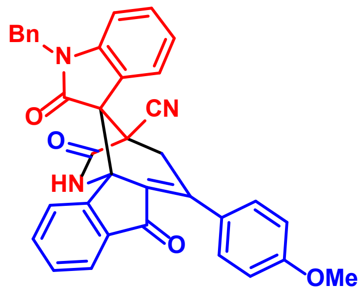

3o

(^{1}H NMR, 400MHz, $CDCl_3$)

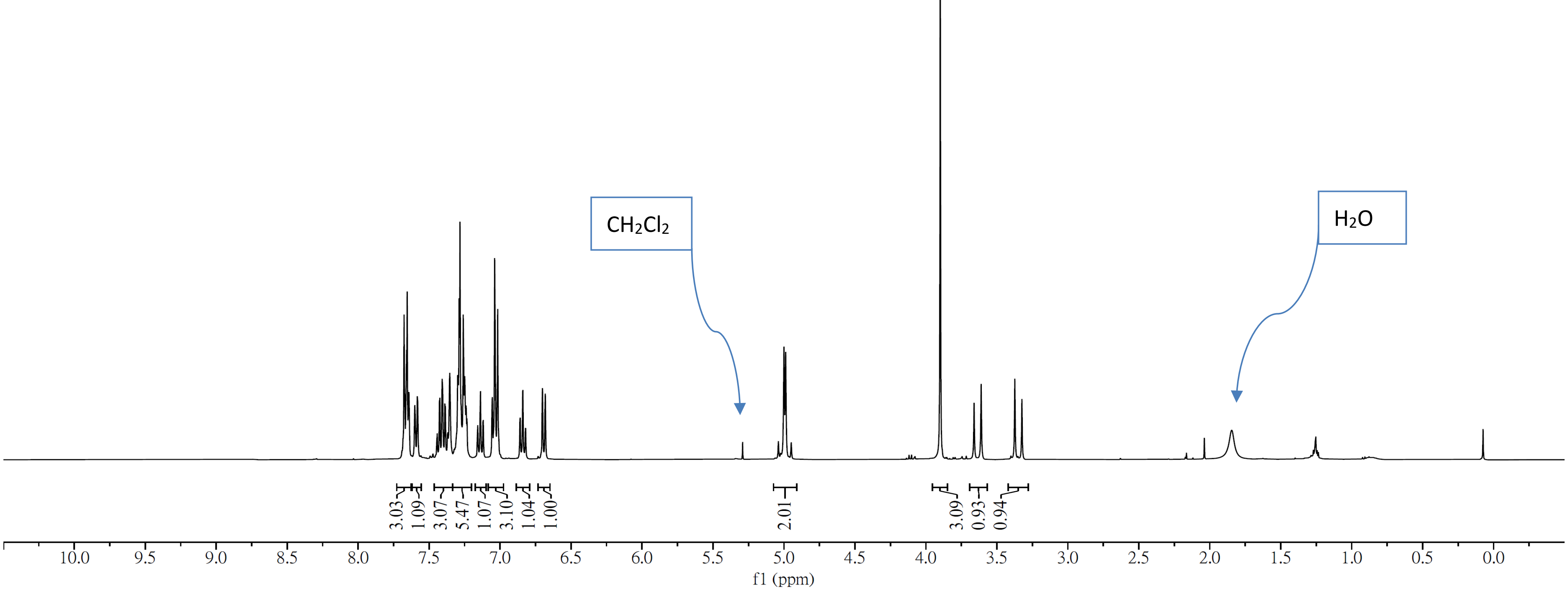

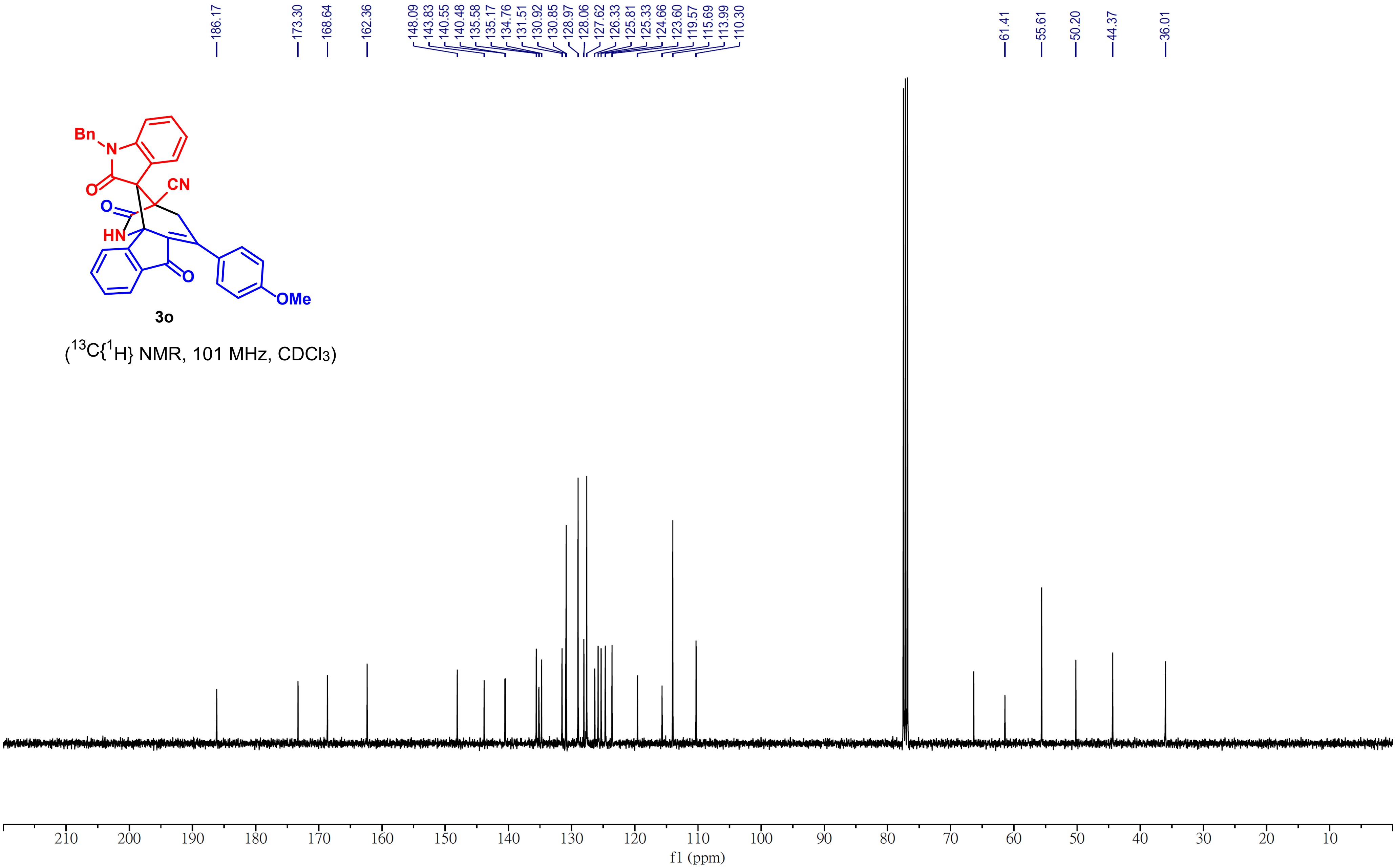

($^{13}C\{^1H\}$ NMR, 101 MHz, $CDCl_3$)

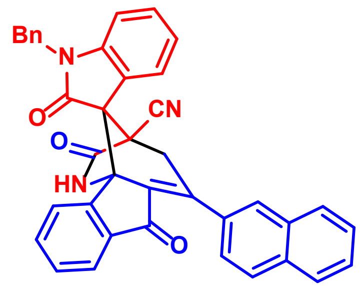

3p

(^{1}H NMR, 400MHz, $CDCl_3$)

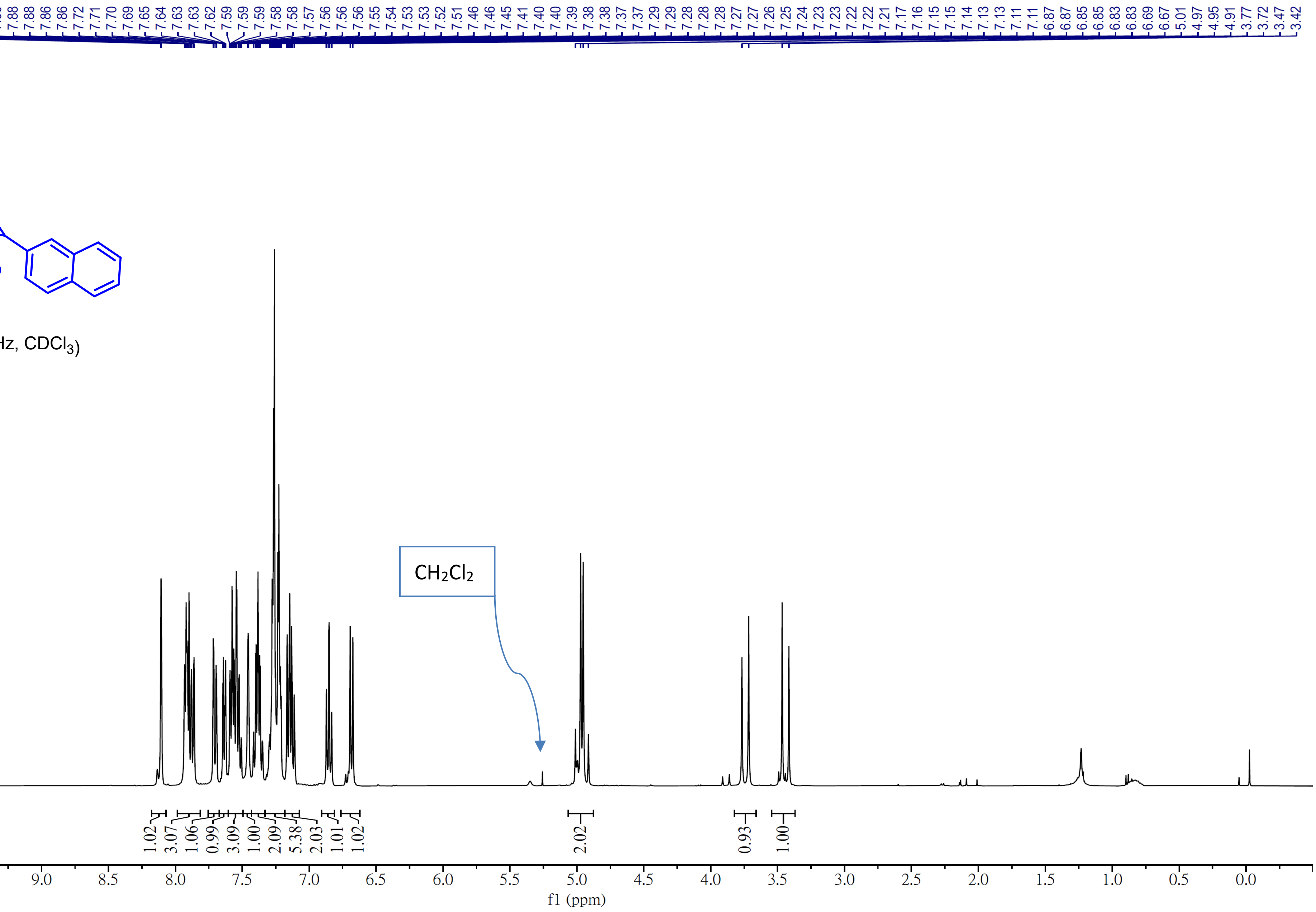

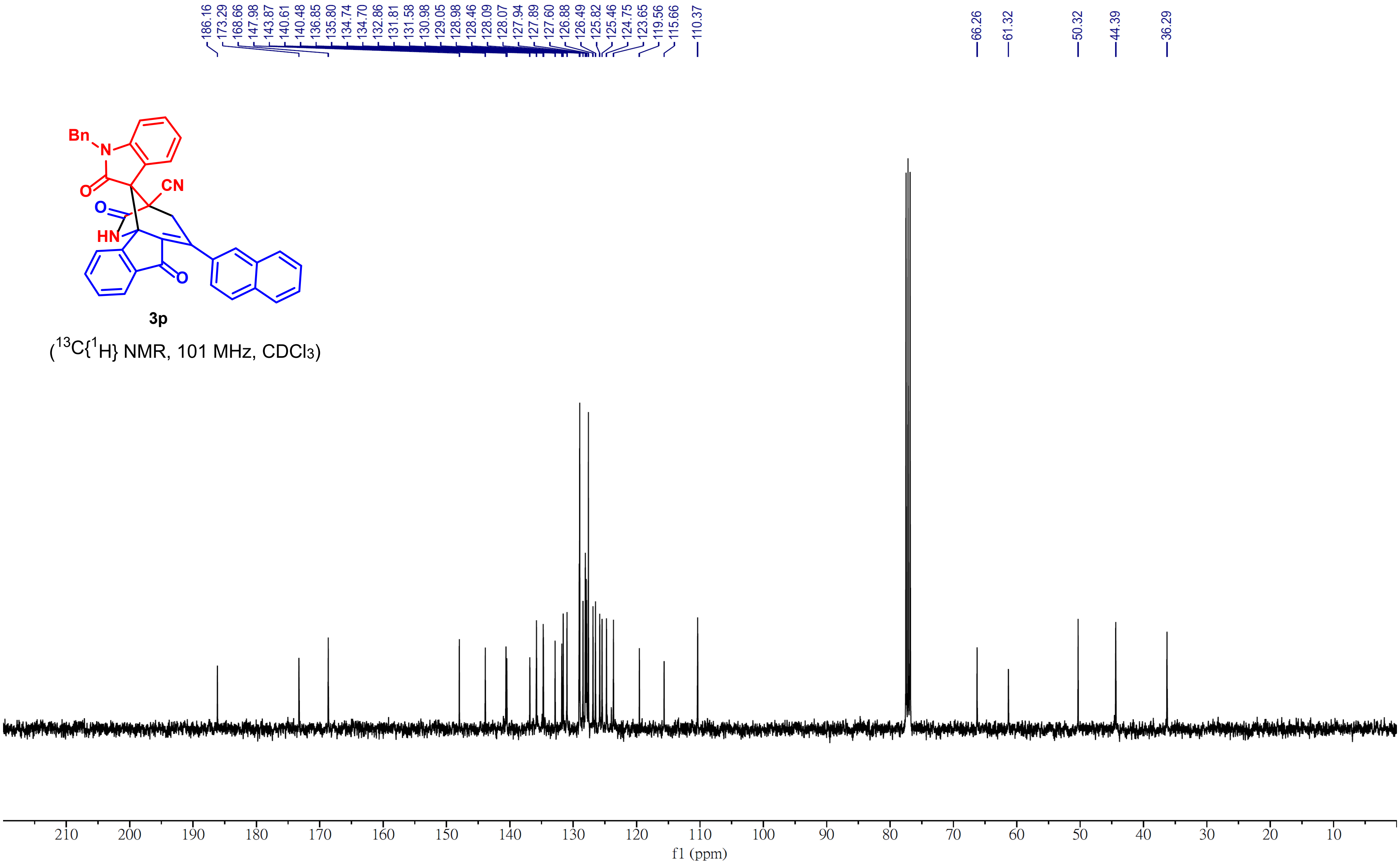
Bn
N
O
CN
O
HN
O
3p
(13C{1H} NMR, 101 MHz, CDCl3)
186.16
173.29
168.66
147.98
143.87
140.61
140.48
136.85
135.80
134.74
134.70
132.86
131.81
131.58
130.98
129.05
128.98
128.46
128.09
128.07
127.94
127.89
127.60
126.88
126.49
125.82
125.46
124.75
123.65
119.56
115.66
110.37
66.26
61.32
50.32
44.39
36.29
210
200
190
180
170
160
150
140
130
120
110
100
90
80
70
60
50
40
30
20
10
f1 (ppm)

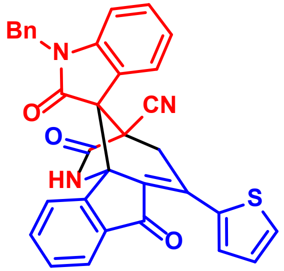

3q

(^{1}H NMR, 400MHz, Acetone-D6)

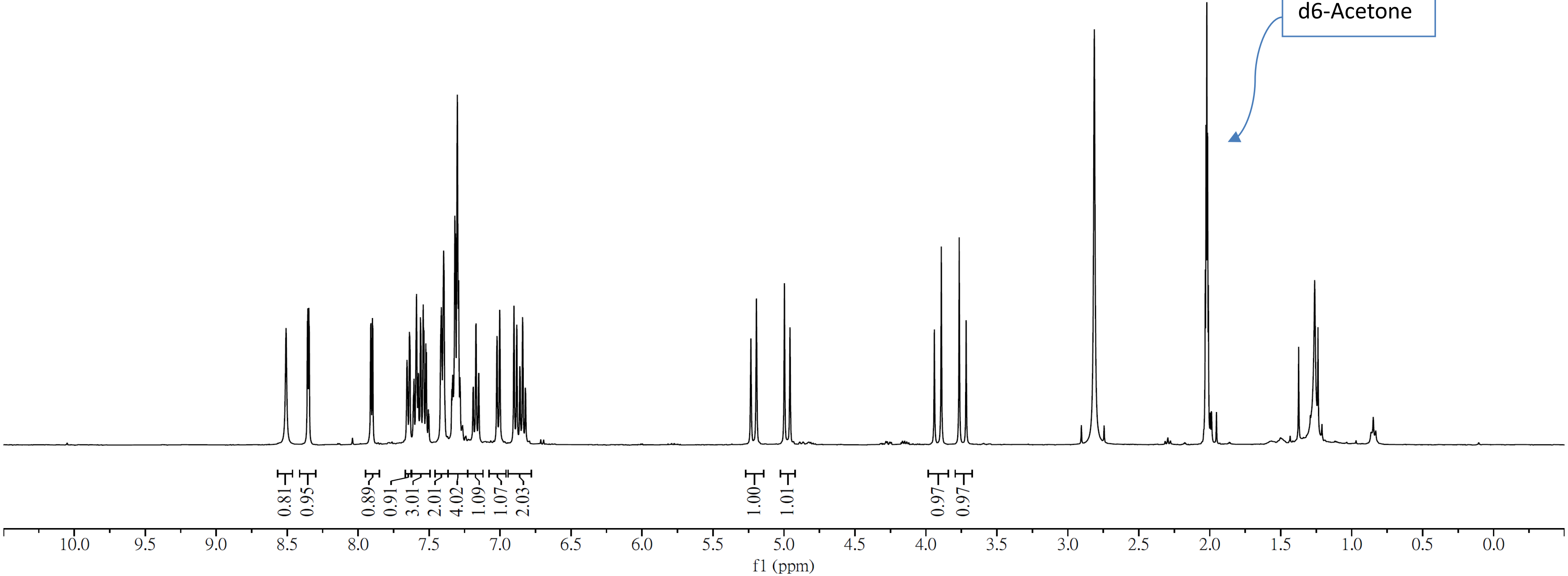

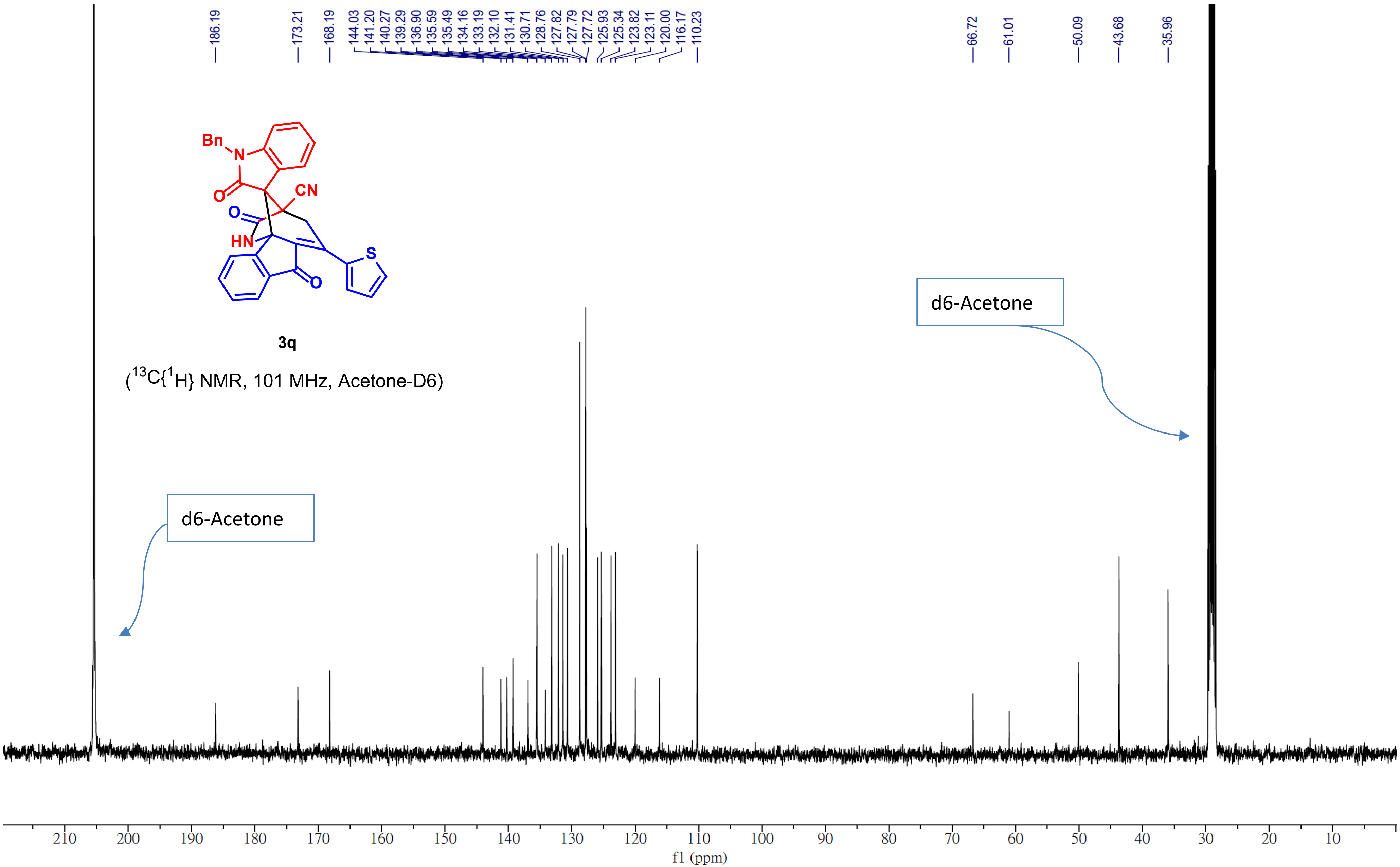

3q

($^{13}C\{^1H\}$ NMR, 101 MHz, Acetone-D6)

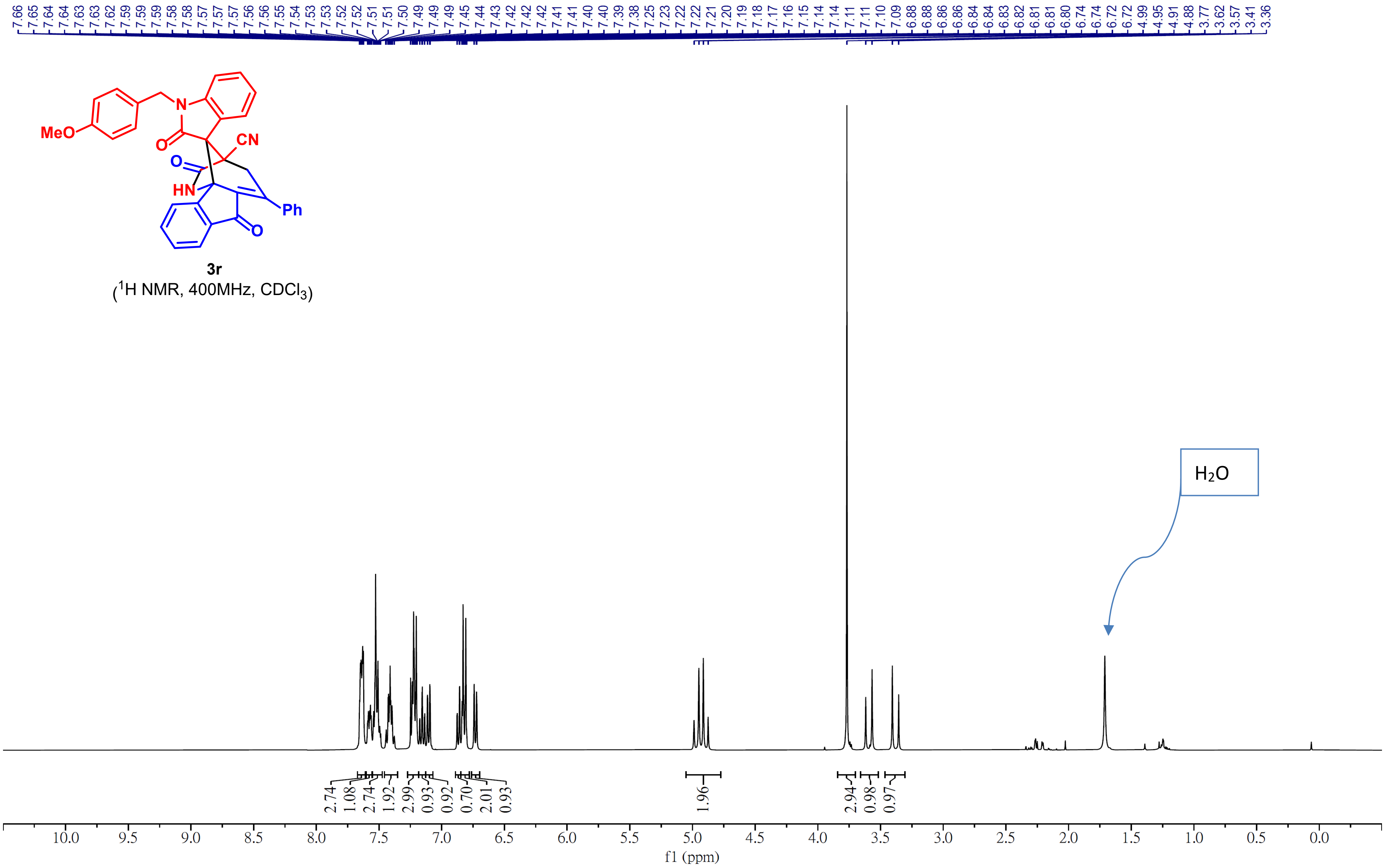
7.66 7.65 7.64 7.63 7.63 7.62 7.59 7.59 7.59 7.58 7.58 7.57 7.57 7.57 7.56 7.56 7.55 7.54 7.53 7.53 7.52 7.52 7.51 7.51 7.50 7.49 7.49 7.49 7.45 7.44 7.43 7.42 7.42 7.42 7.41 7.41 7.40 7.40 7.39 7.38 7.25 7.23 7.22 7.22 7.21 7.20 7.19 7.18 7.17 7.16 7.15 7.14 7.14 7.11 7.11 7.10 7.09 6.88 6.88 6.86 6.86 6.84 6.84 6.83 6.82 6.81 6.81 6.80 6.74 6.74 6.72 6.72 4.99 4.95 4.91 4.88 3.77 3.62 3.57 3.41 3.36
MeO
N
O
CN
O
HN
Ph
O
3r
(^{1}H NMR, 400MHz, $CDCl_3$)
H_2O
2.74 1.08 2.74 1.92 2.99 0.93 0.92 0.70 2.01 0.93 1.96 2.94 0.98 0.97
10.0 9.5 9.0 8.5 8.0 7.5 7.0 6.5 6.0 5.5 5.0 4.5 4.0 3.5 3.0 2.5 2.0 1.5 1.0 0.5 0.0
f1 (ppm)

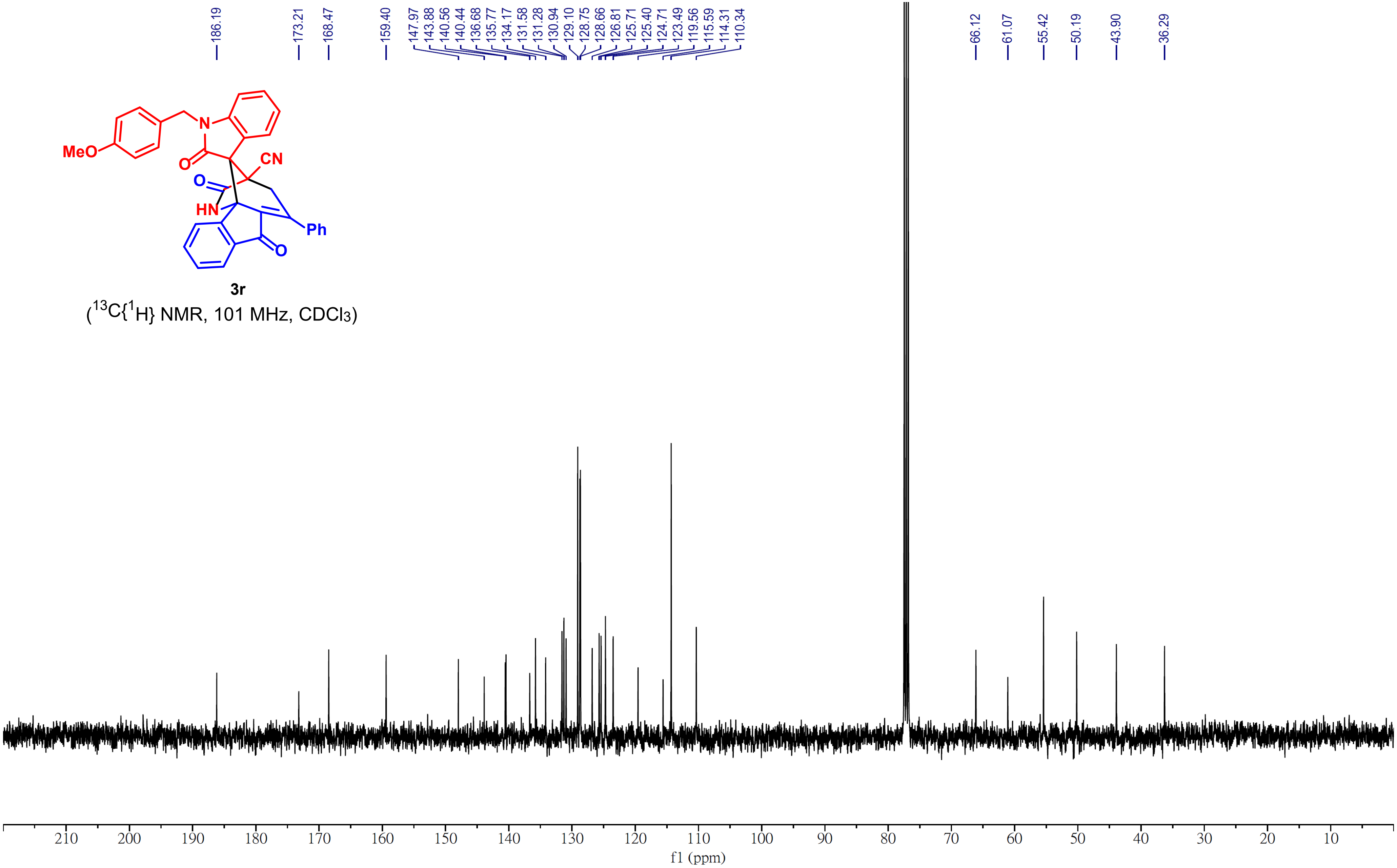

3r

($^{13}C\{^{1}H\}$ NMR, 101 MHz, $CDCl_3$)

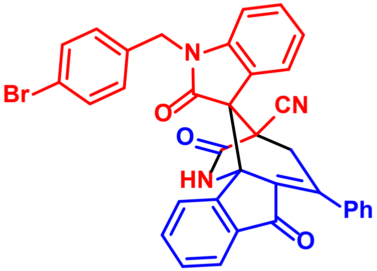

3s

(^{1}H NMR, 400MHz, $CDCl_3$)

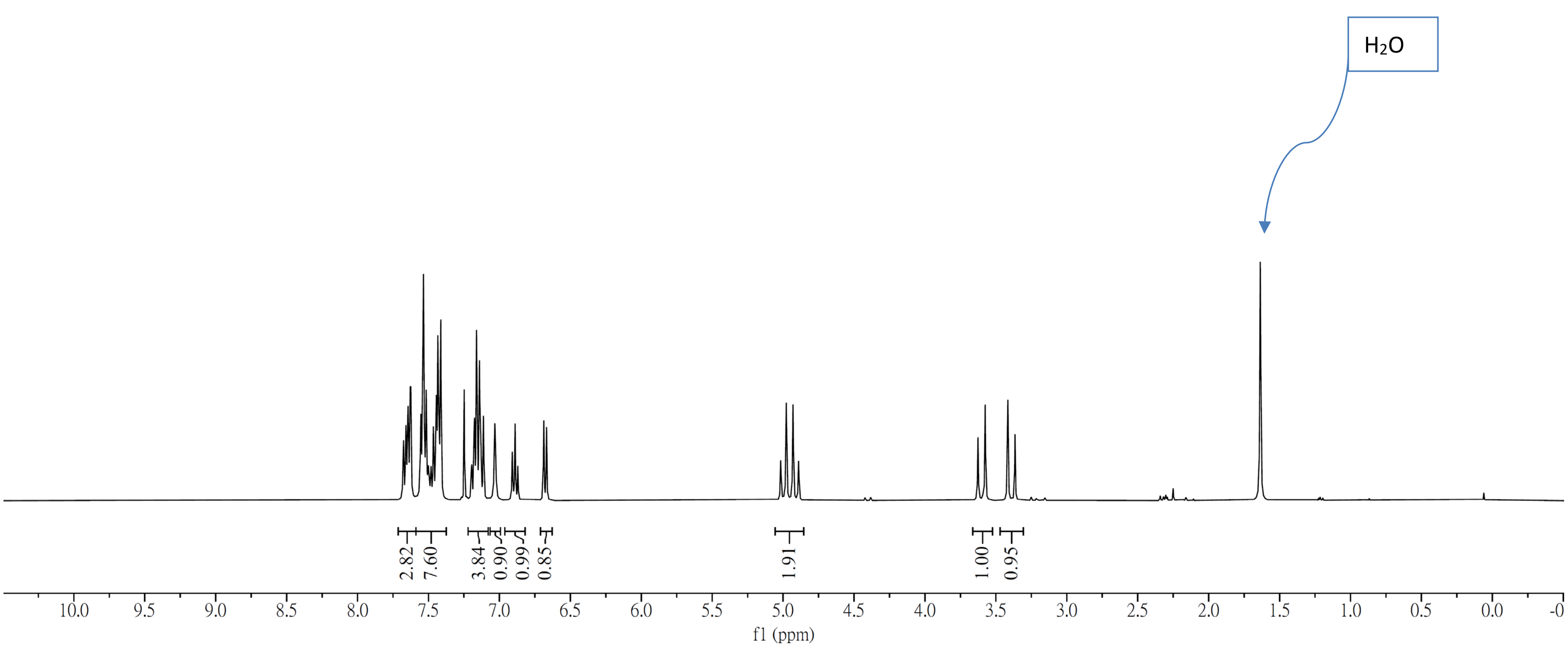

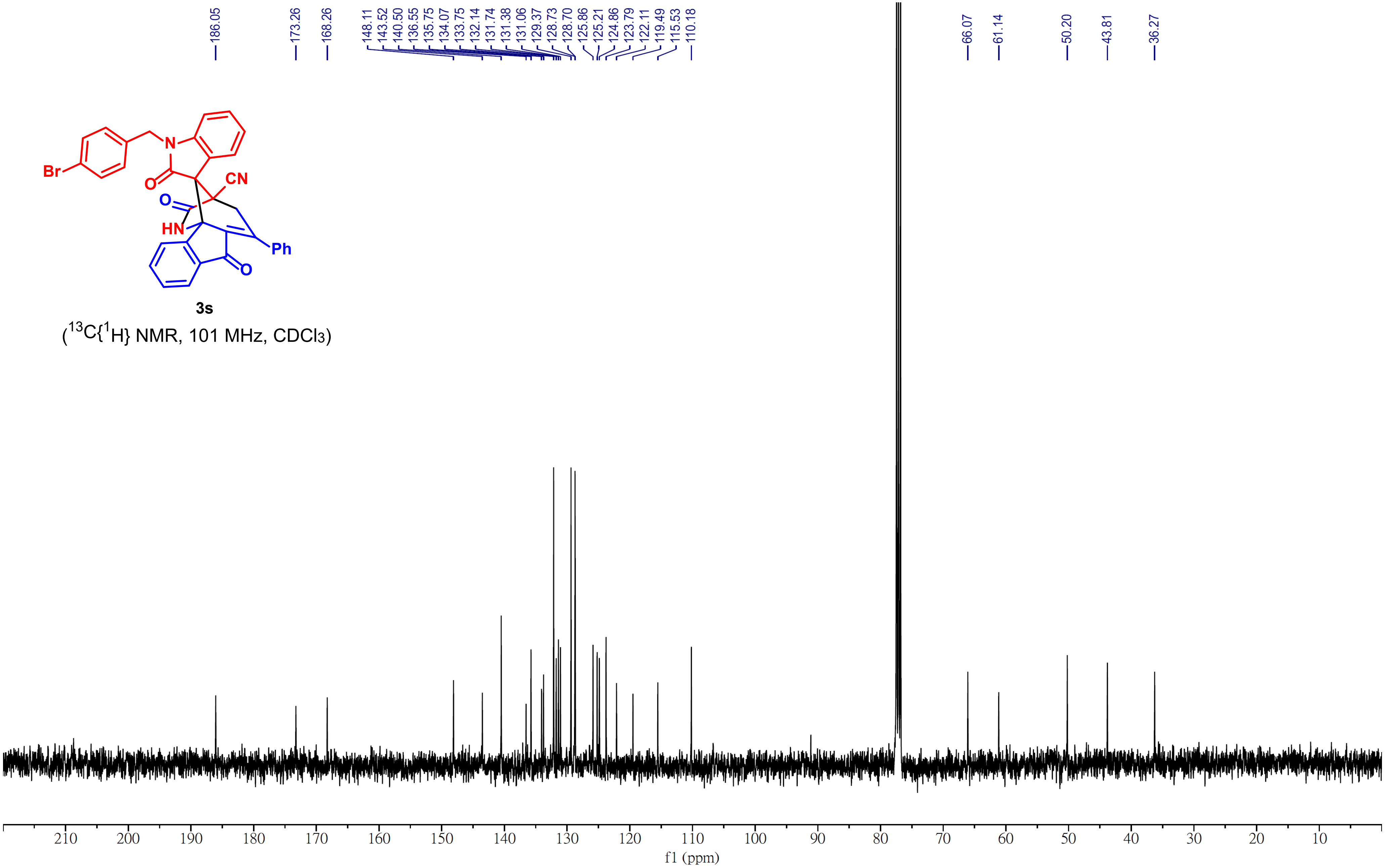

3s

($^{13}C\{^{1}H\}$ NMR, 101 MHz, $CDCl_3$)

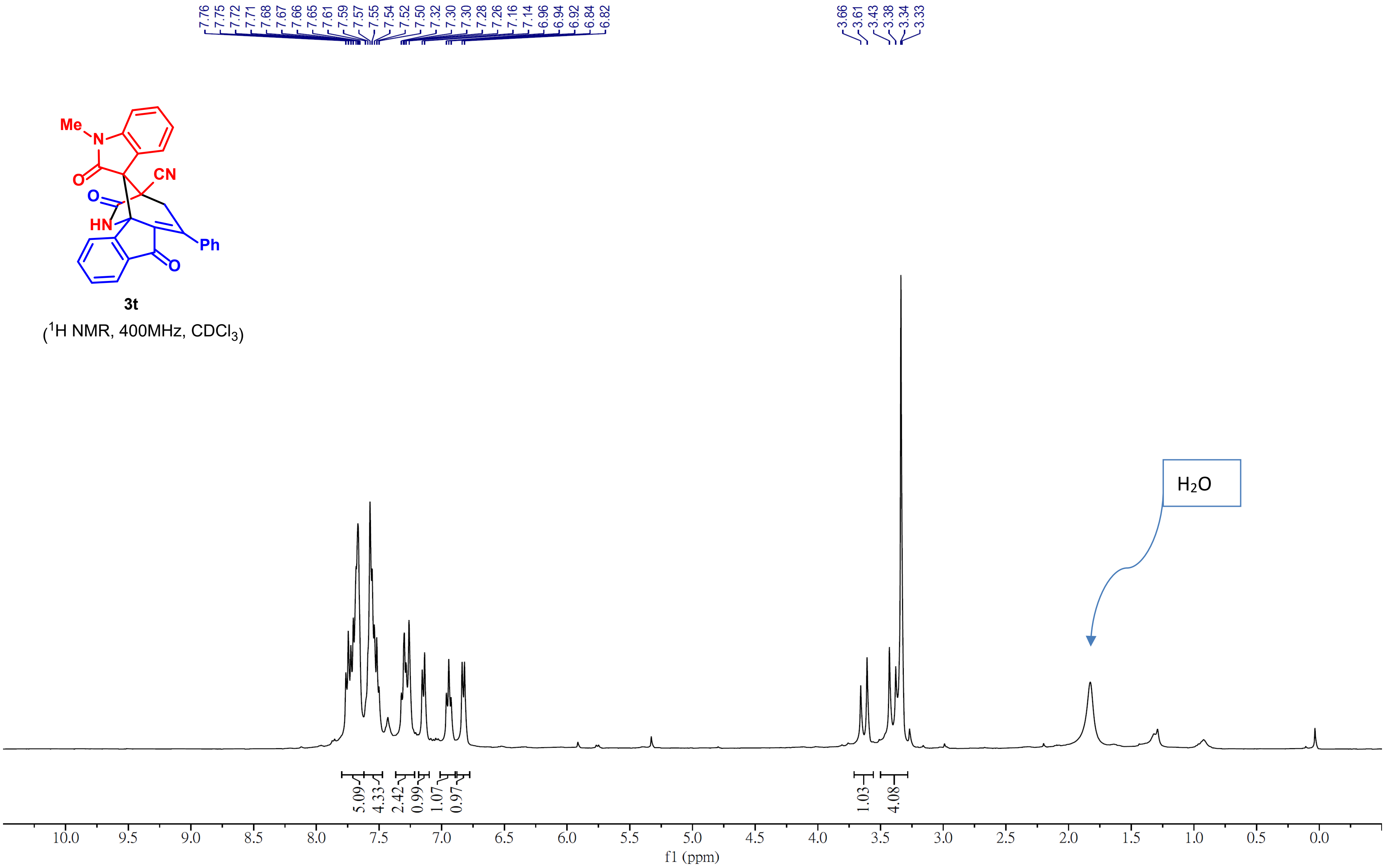
7.76 7.75 7.72 7.71 7.68 7.67 7.66 7.65 7.61 7.59 7.57 7.55 7.54 7.52 7.50 7.32 7.30 7.30 7.28 7.26 7.16 7.14 6.96 6.94 6.92 6.84 6.82
3.66 3.61 3.43 3.38 3.34 3.33
Me
N
O
CN
O
HN
Ph
O
3t
(^{1}H NMR, 400MHz, $CDCl_3$)
H_2O
5.09 4.33 2.42 0.99 1.07 0.97
1.03 4.08
10.0 9.5 9.0 8.5 8.0 7.5 7.0 6.5 6.0 5.5 5.0 4.5 4.0 3.5 3.0 2.5 2.0 1.5 1.0 0.5 0.0
f1 (ppm)

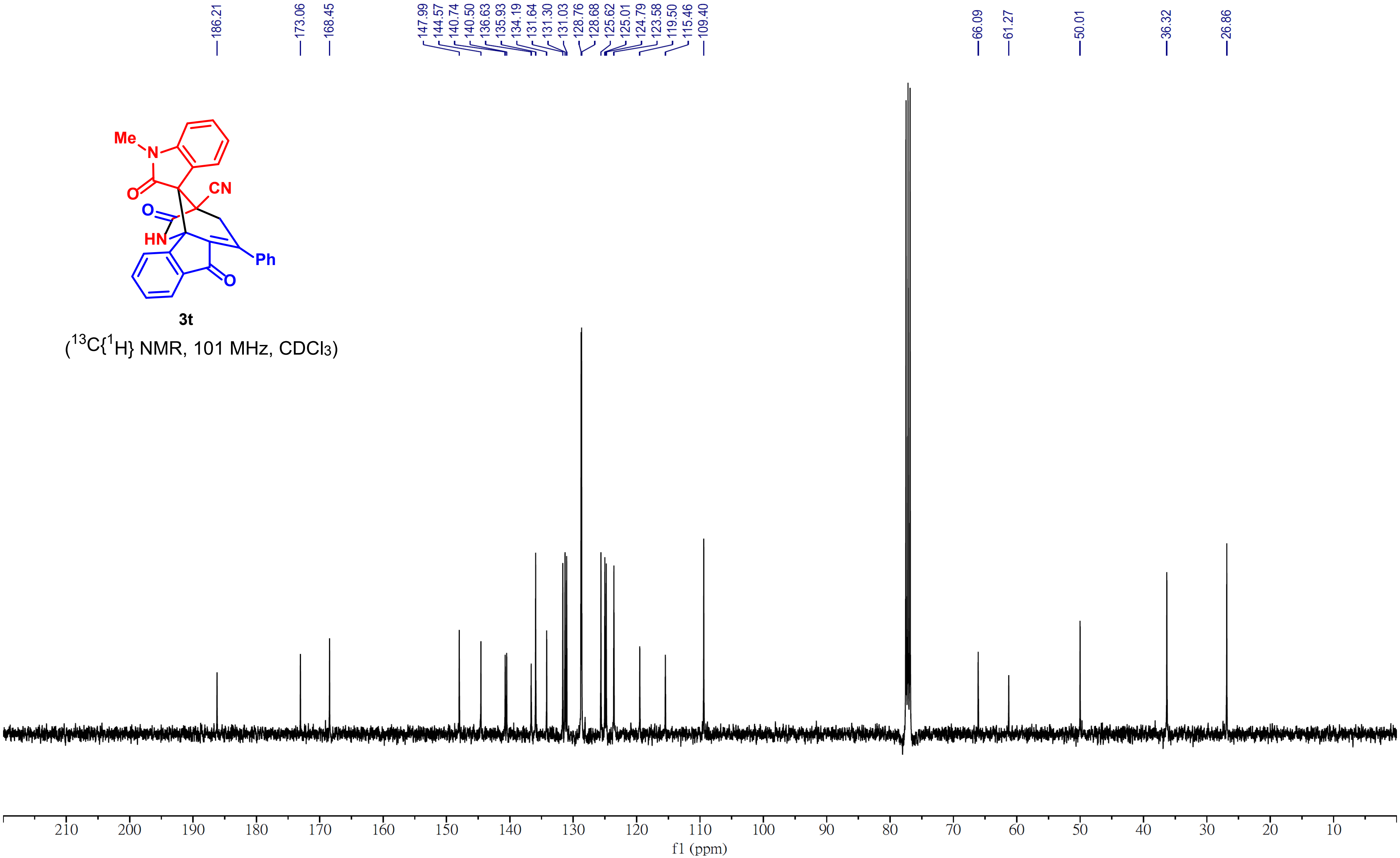

3t

($^{13}C\{^{1}H\}$ NMR, 101 MHz, $CDCl_3$)

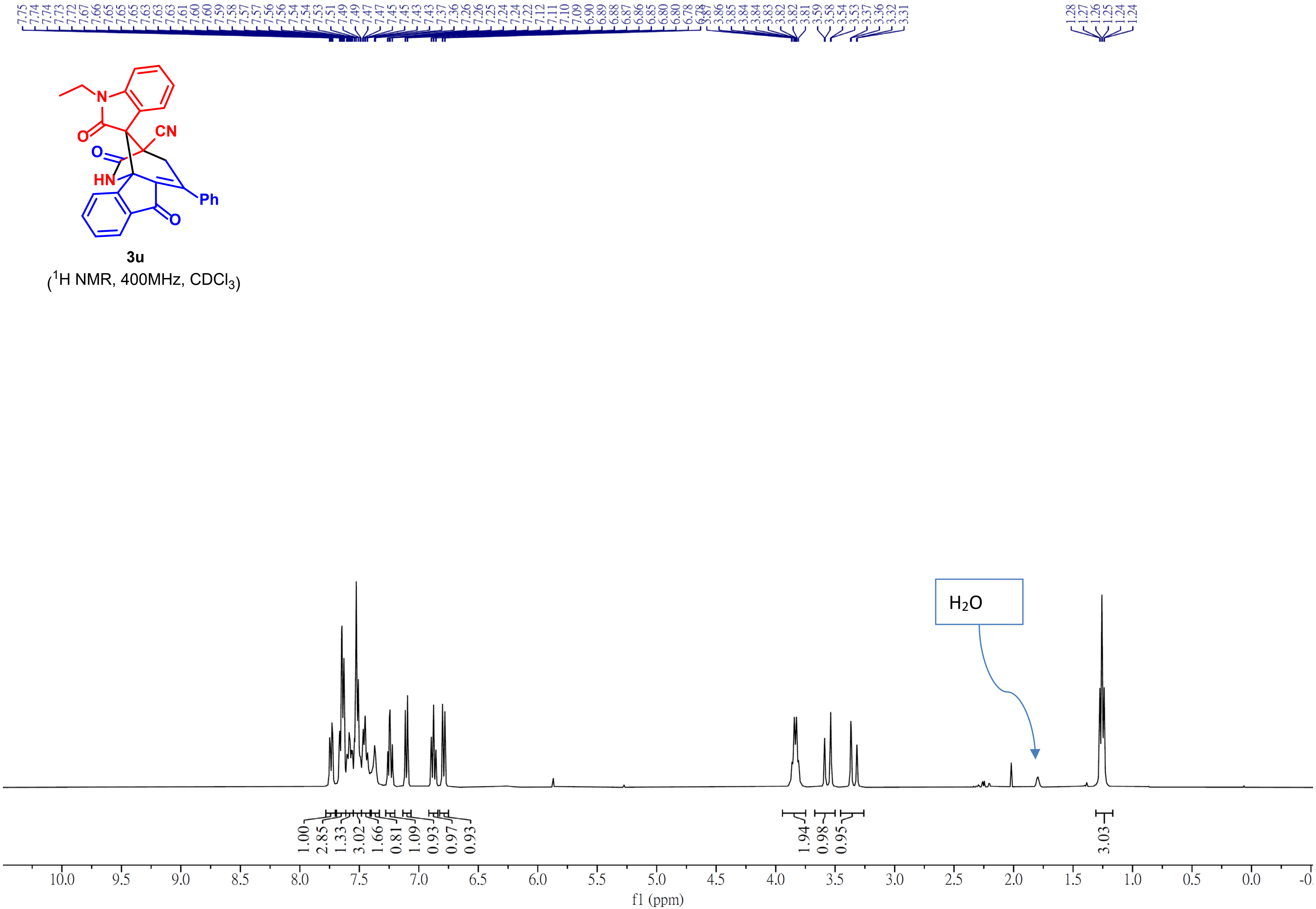

3u

(^{1}H NMR, 400MHz, $CDCl_3$)

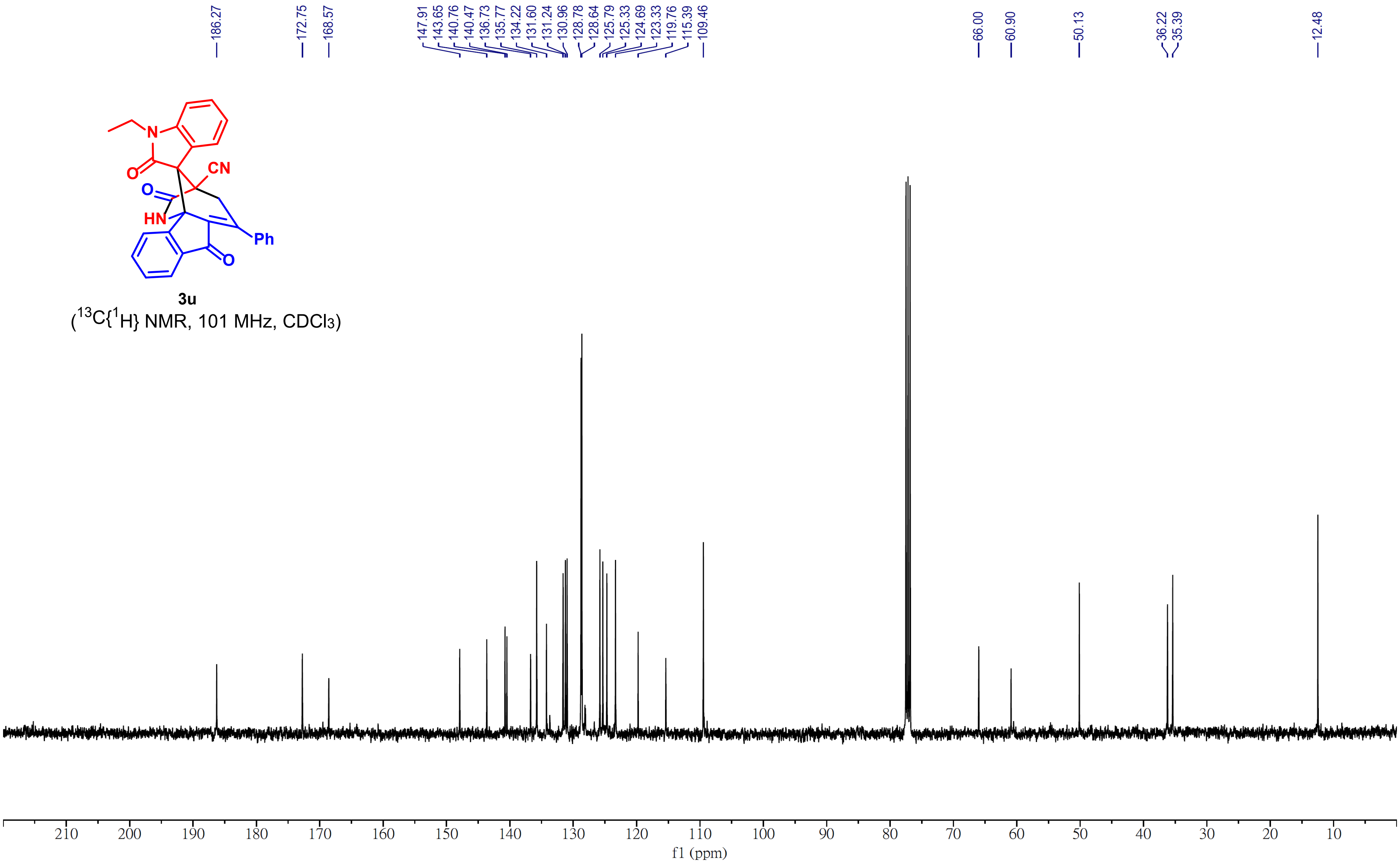

3u

($^{13}C\{^{1}H\}$ NMR, 101 MHz, $CDCl_3$)

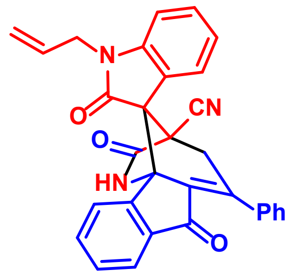

3u

(^{1}H NMR, 400MHz, $CDCl_3$)

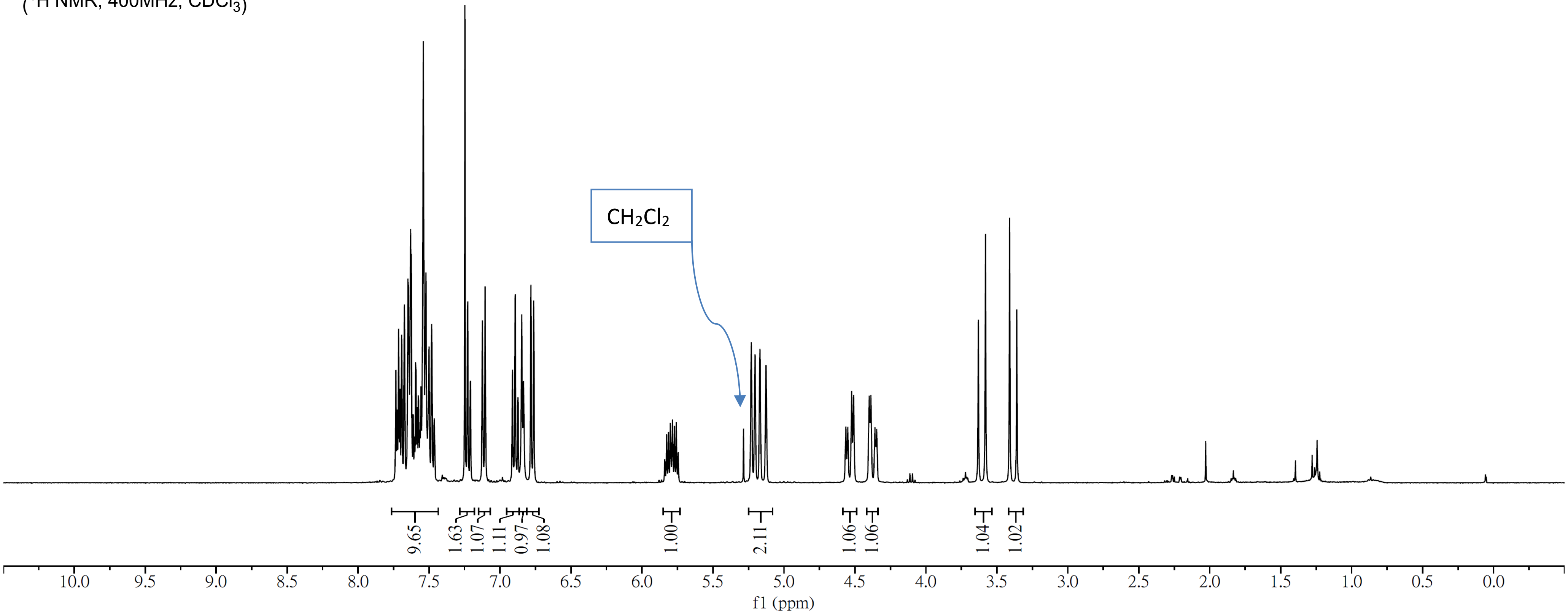

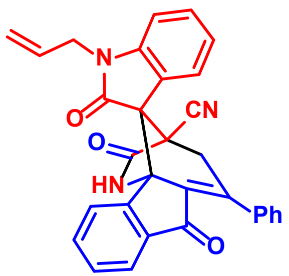

3u

($^{13}C\{^{1}H\}$ NMR, 101 MHz, $CDCl_3$)

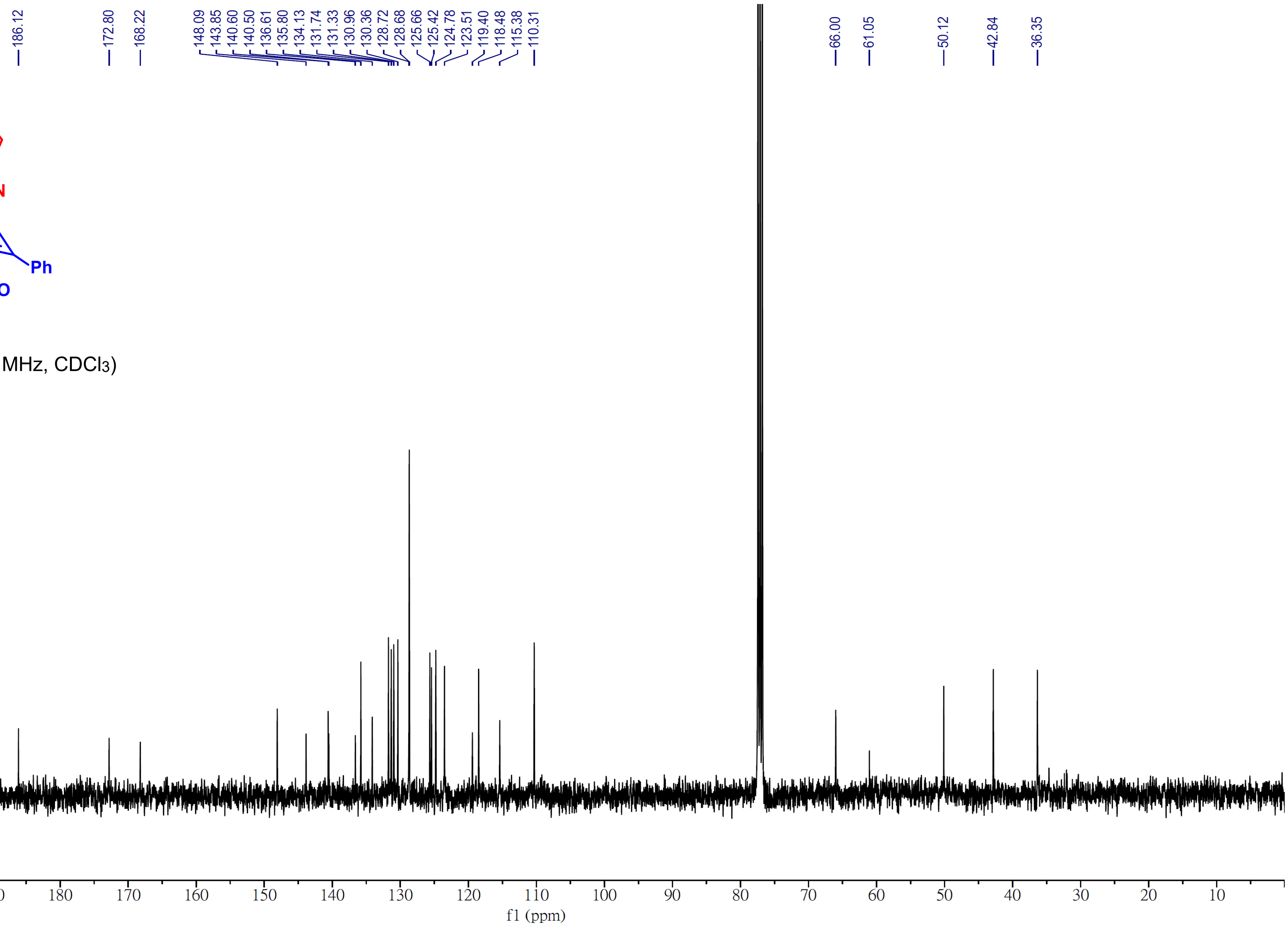

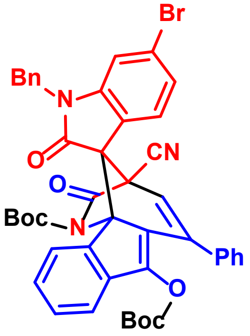

4

(^{1}H NMR, 400MHz, $CDCl_3$)

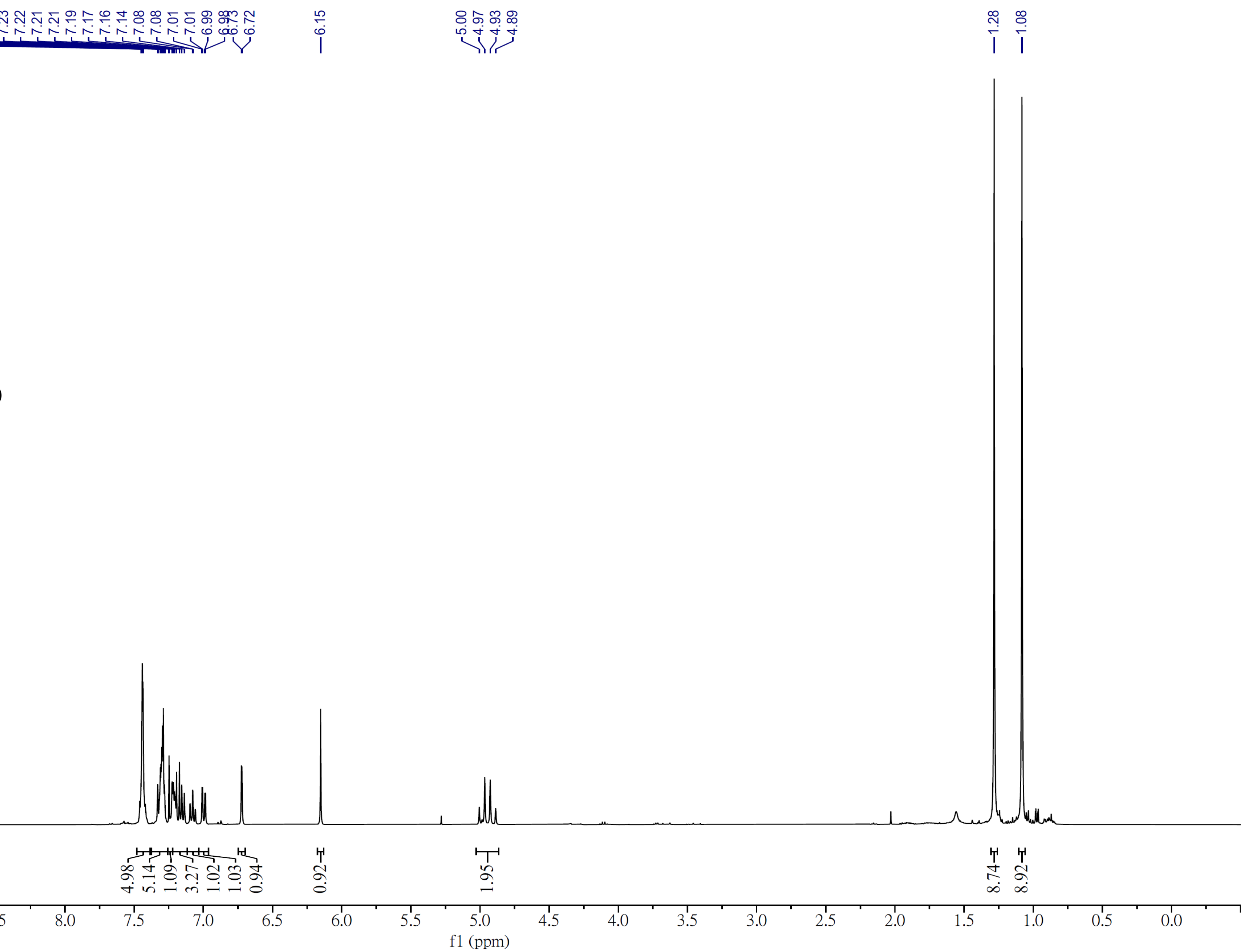

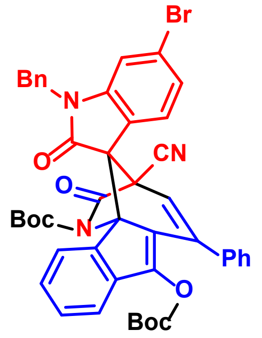

4

($^{13}C\{^{1}H\}$ NMR, 101 MHz, $CDCl_3$)

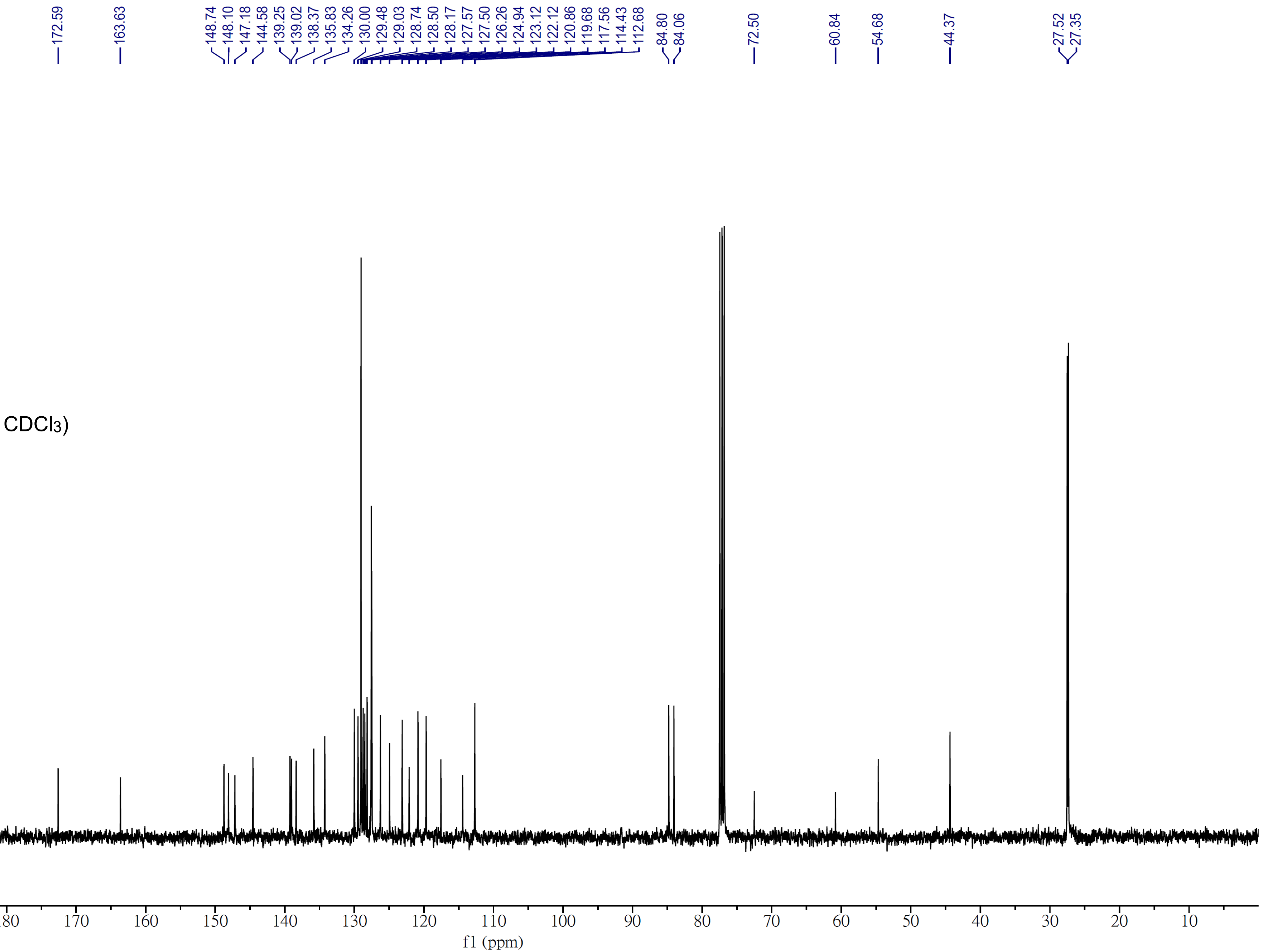

12. HRMS spectra of new compounds

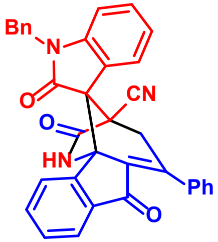

3a

Spectrum

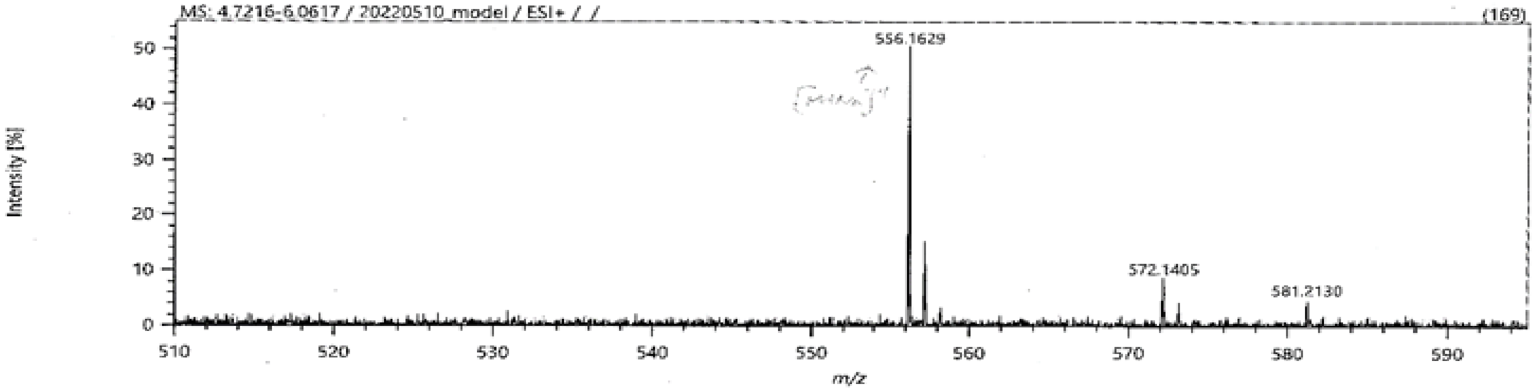

Elemental Composition

Parameters

Tolerance:	±10.00 ppm
Electron:	Odd/Even
Charge:	+1
DBE:	-99.0 - 999.0

Elements Set 1:

Symbol	C	H	O	N	Na
Min	0	0	3	3	1
Max	400	1000	3	3	1

Results

Mass	Formula	Calculated Mass	Mass Difference [mDa]	Mass Difference [ppm]	DBE
556.16288	C35 H23 N3 O3 Na	556.16316	-0.28	-0.50	25.5

1 / 1

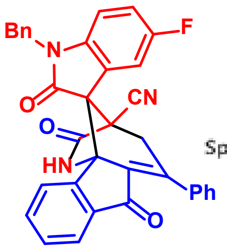

3b

ele-5-F (HR-ESI)

Spectrum

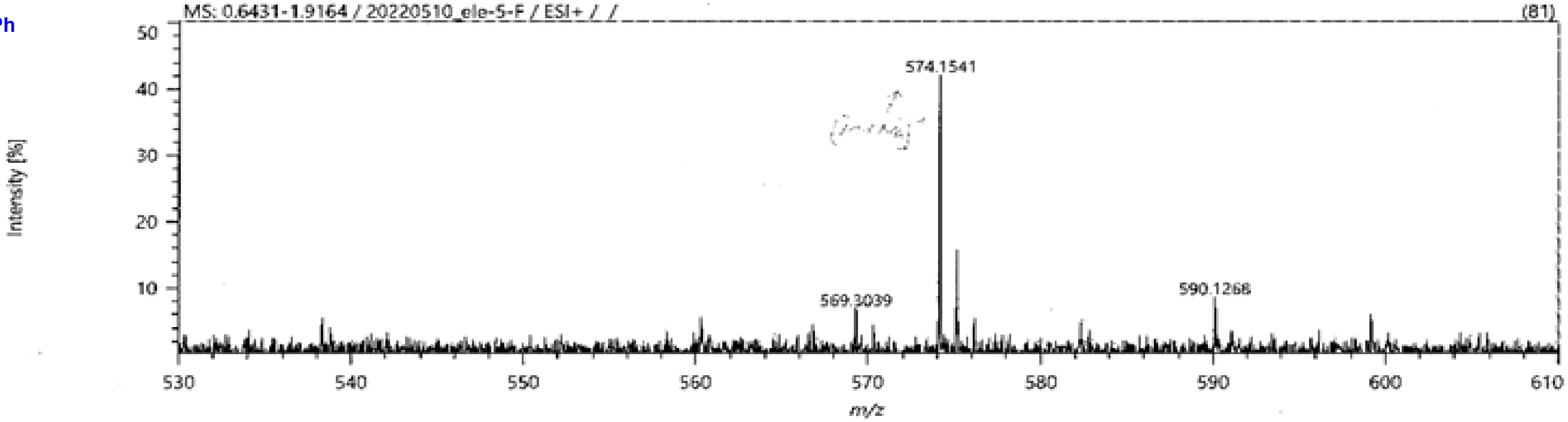

Elemental Composition

Parameters

Tolerance:	±10.00 ppm
Electron:	Odd/Even
Charge:	+1
DBE:	-99.0 - 999.0

Elements Set 1:

Symbol	C	H	O	N	Na	F
Min	0	0	3	3	1	1
Max	400	1000	4	3	1	1

Results

Mass	Formula	Calculated Mass	Mass Difference [mDa]	Mass Difference [ppm]	DBE
574.15415	C35 H22 N3 O3 F Na	574.15374	0.41	0.71	25.5

1 / 1

ele-5-Cl (2017-853)

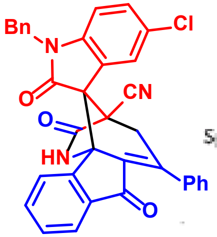

3c

Spectrum

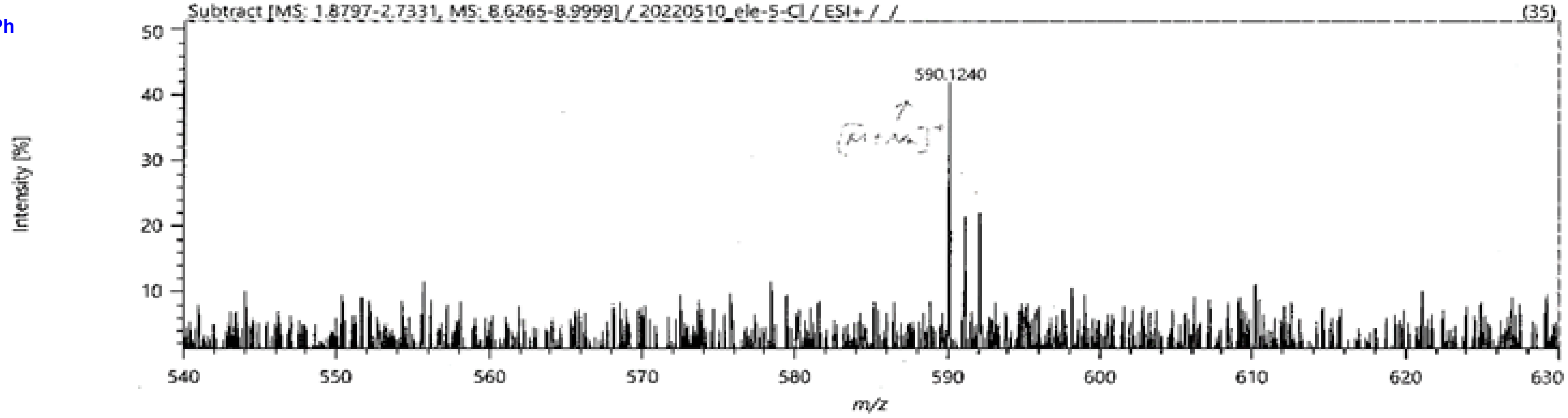

Elemental Composition

Parameters

Tolerance:	±10.00 ppm
Electron:	Odd/Even
Charge:	+1
DBE:	-99.0 - 999.0

Elements Set 1:

Symbol	C	H	O	N	Na	Cl
Min	0	0	3	3	1	1
Max	400	1000	4	3	1	1

Results

Mass	Formula	Calculated Mass	Mass Difference [mDa]	Mass Difference [ppm]	DBE
590.12398	C35 H22 N3 O3 Na Cl	590.12419	-0.21	-0.36	25.5

1 / 1

ele-5-Br (HR-ESI)

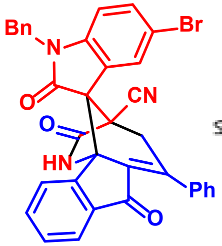

3d

Spectrum

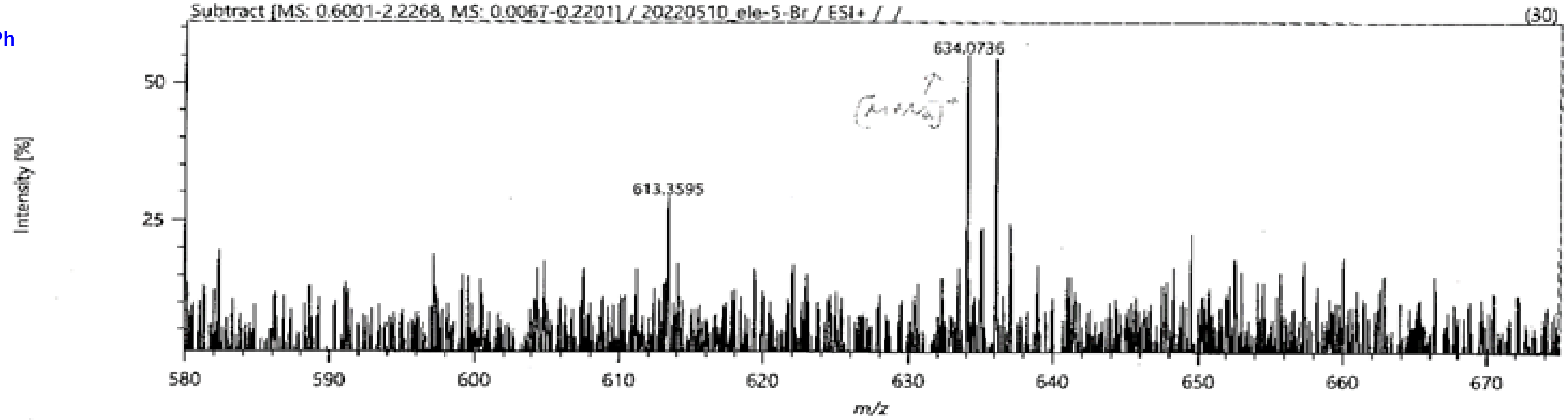

Elemental Composition

Parameters

Tolerance:	±10.00 ppm
Electron:	Odd/Even
Charge:	+1
DBE:	-99.0 - 999.0

Elements Set 1:

Symbol	C	H	O	N	Na	Br
Min	0	0	3	3	1	1
Max	400	1000	4	3	1	1

Results

Mass	Formula	Calculated Mass	Mass Difference [mDa]	Mass Difference [ppm]	DBE
634.07361	C35 H22 N3 O3 Na Br	634.07368	-0.06	-0.10	25.5

1 / 1

ele-5-Me (HR-ESI)

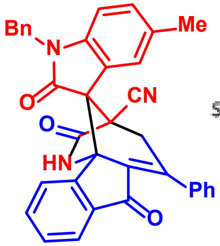

3e

Spectrum

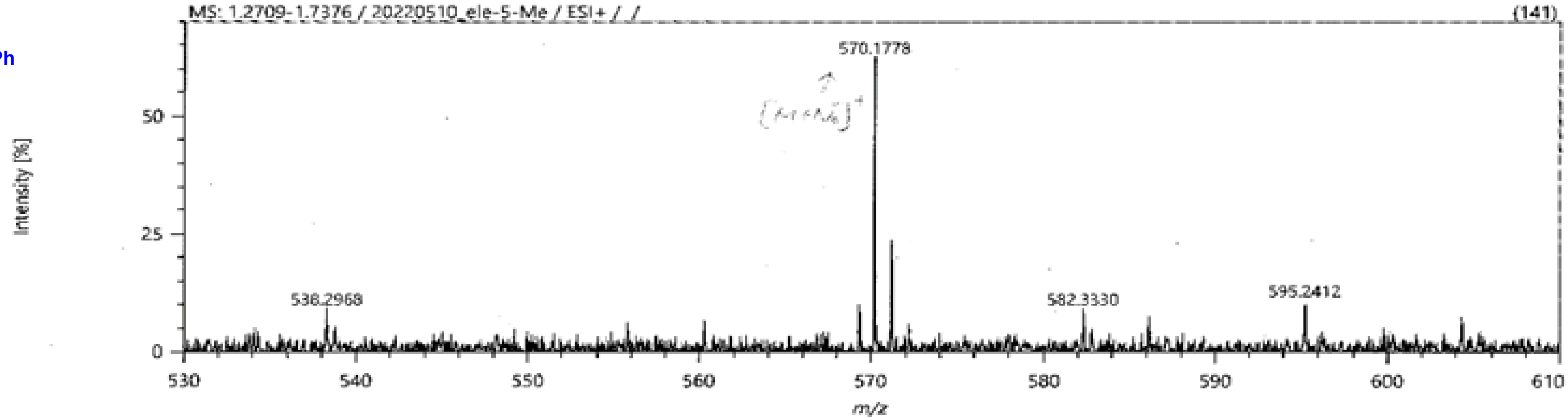

Elemental Composition

Parameters

Tolerance:	±10.00 ppm
Electron:	Odd/Even
Charge:	+1
DBE:	-99.0 - 999.0

Elements Set 1:

Symbol	C	H	O	N	Na
Min	0	0	3	3	1
Max	400	1000	4	3	1

Results

Mass	Formula	Calculated Mass	Mass Difference [mDa]	Mass Difference [ppm]	DBE
570.17784	C36 H25 N3 O3 Na	570.17881	-0.98	-1.71	25.5

1 / 1

ele-5-OMe (4.12-853)

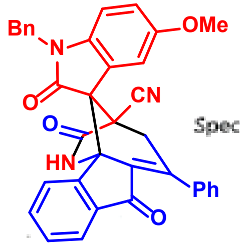

3f

Spectrum

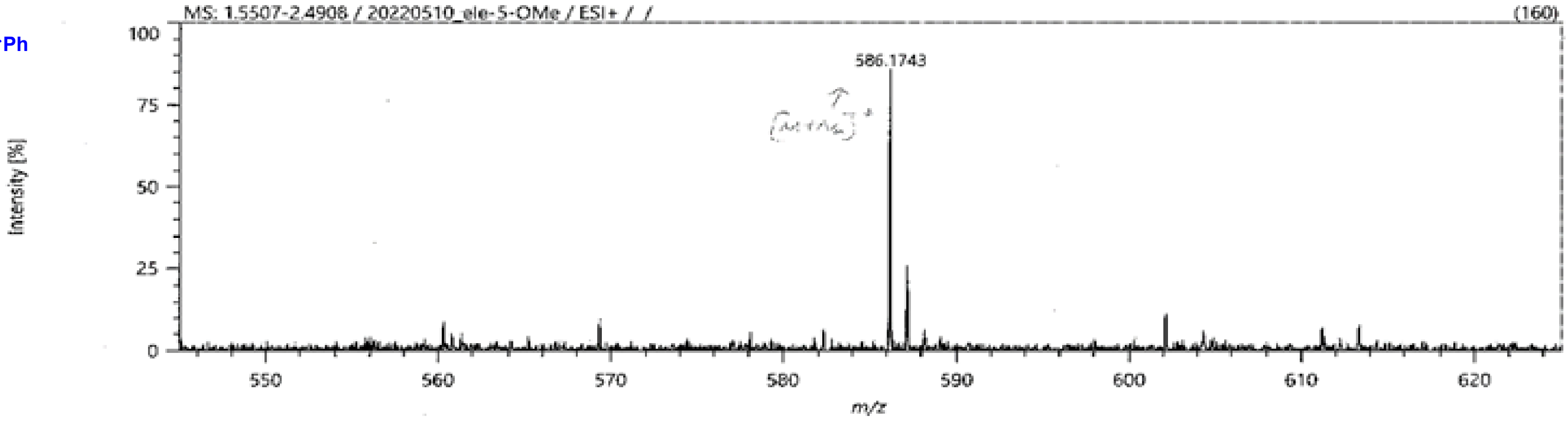

Elemental Composition

Parameters

Tolerance:	±10.00 ppm
Electron:	Odd/Even
Charge:	+1
DBE:	-99.0 - 999.0

Elements Set 1:

Symbol	C	H	O	N	Na
Min	0	0	4	3	1
Max	400	1000	4	3	1

Results

Mass	Formula	Calculated Mass	Mass Difference [mDa]	Mass Difference [ppm]	DBE
586.17429	C36 H25 N3 O4 Na	586.17373	0.56	0.96	25.5

1 / 1

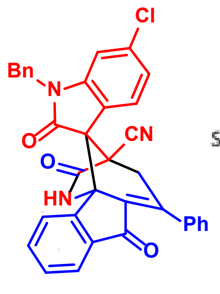

3g

ele-6-Cl (HR-ESI)

Spectrum

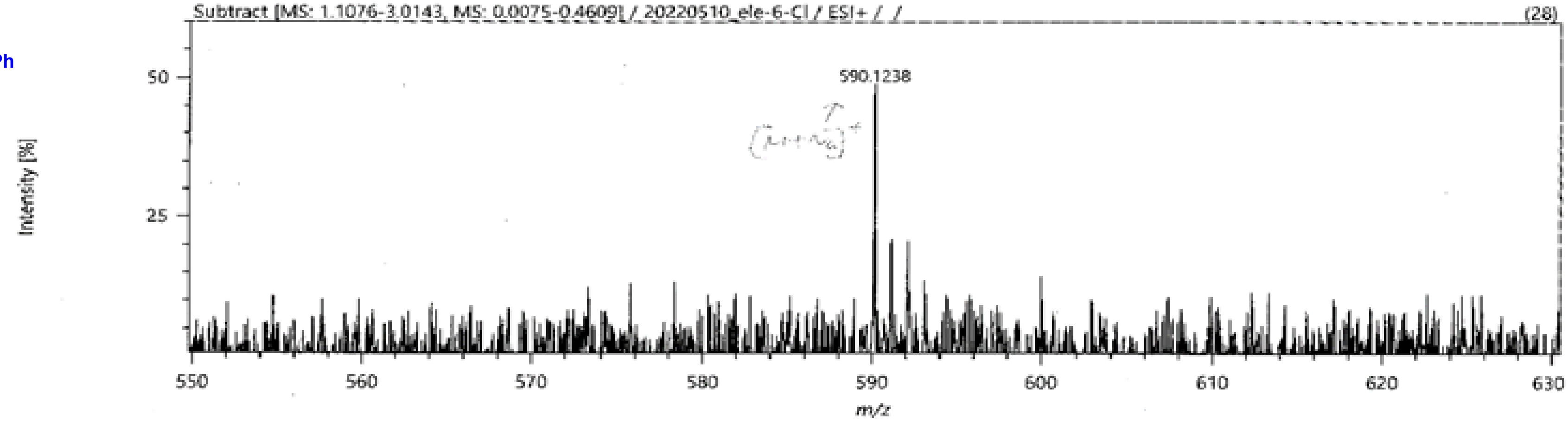

Elemental Composition

Parameters

Tolerance:	±10.00 ppm
Electron:	Odd/Even
Charge:	+1
DBE:	-99.0 - 999.0

Elements Set 1:

Symbol	C	H	O	N	Na	Cl
Min	0	0	3	3	1	1
Max	400	1000	4	3	1	1

Results

Mass	Formula	Calculated Mass	Mass Difference [mDa]	Mass Difference [ppm]	DBE
590.12378	C35 H22 N3 O3 Na Cl	590.12419	-0.41	-0.70	25.5

1 / 1

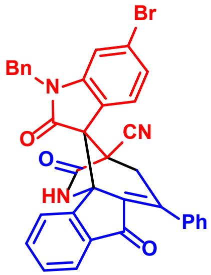

3h

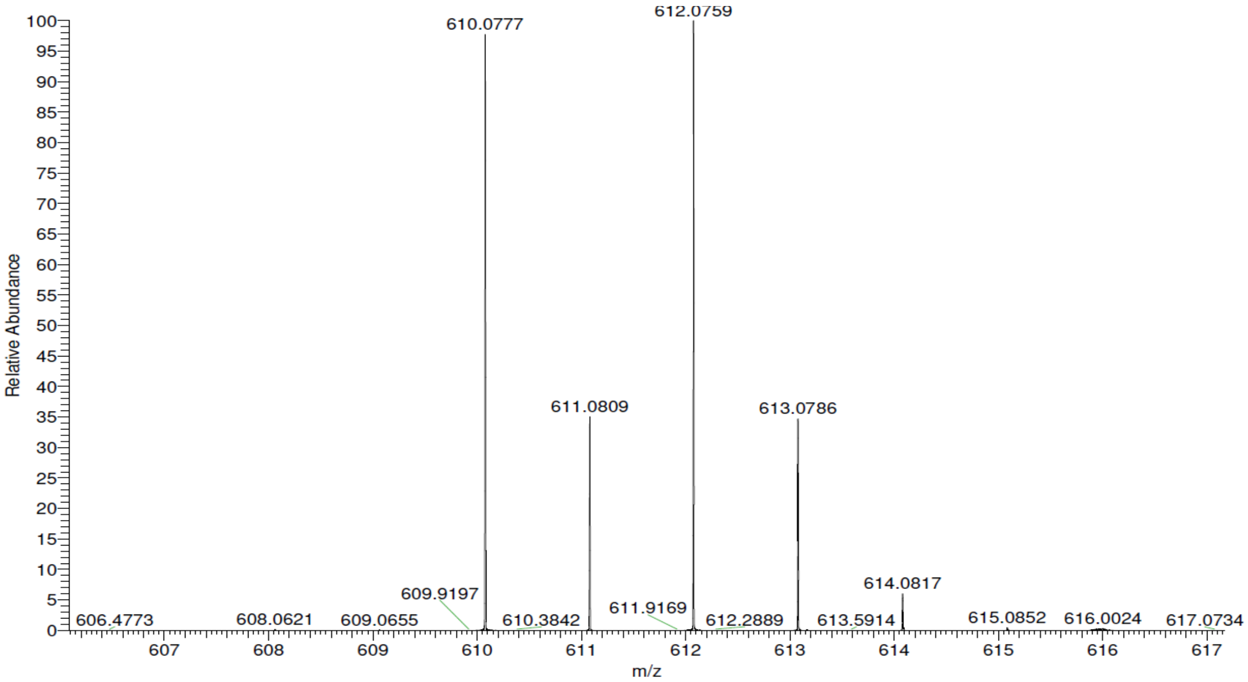

75-148p2-ele-6Br-N-H#1-20 RT: 0.02-0.57 AV: 20
T: FTMS - p ESI Full ms [250.00-1000.00]
m/z= 609.4199-610.9189

Isotope	Min	Max
N-14	3	3
O-16	3	3
C-12	35	35
H-1	0	60
Br-79	1	1

Charge 1
Mass tolerance 140.00 ppm
Nitrogen rule not used
RDB equiv -1.00-100.00
max results 1

m/z	Intensity	Relative	Theo. Mass	Delta (ppm)	Composition
610.0777	1662582.8	100.00	610.0761	2.73	$C_{35}H_{21}O_3N_3Br$

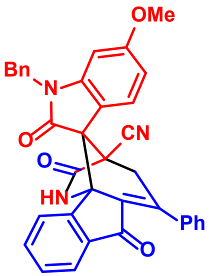

3i

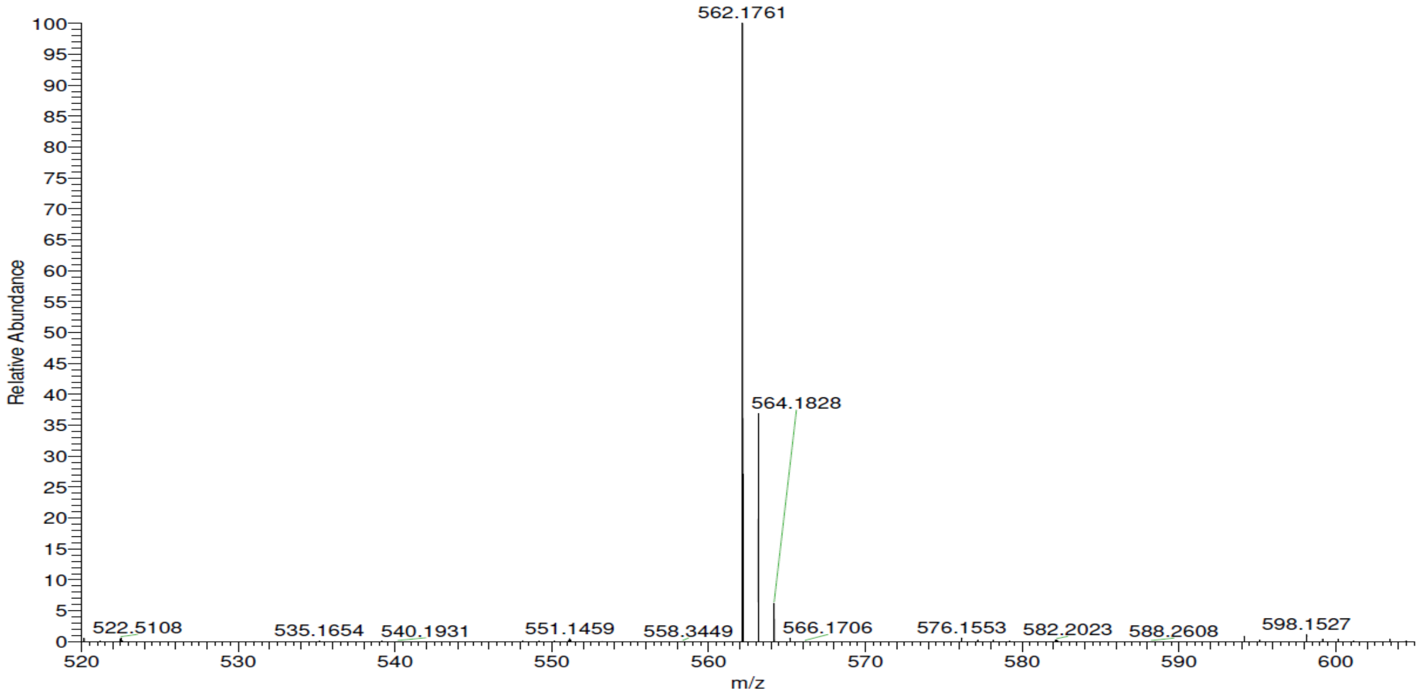

82-148p2-ele-6OMe-N-H#1-20 RT: 0.02-0.57 AV: 20
T: FTMS - p ESI Full ms [250.00-1000.00]
m/z= 520.0000-605.0000

Isotope	Min	Max
N-14	2	3
O-16	2	4
C-12	28	36
H-1	0	60
Br-79	0	1

Charge 1
Mass tolerance 140.00 ppm
Nitrogen rule not used
RDB equiv -1.00-100.00
max results 1

m/z	Intensity	Relative	Theo. Mass	Delta (ppm)	Composition
562.1761	1145723.3	100.00	562.1761	-0.08	$C_{36}H_{24}O_4N_3$

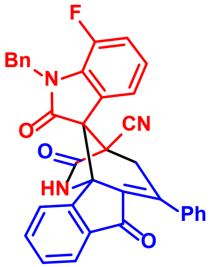

3j

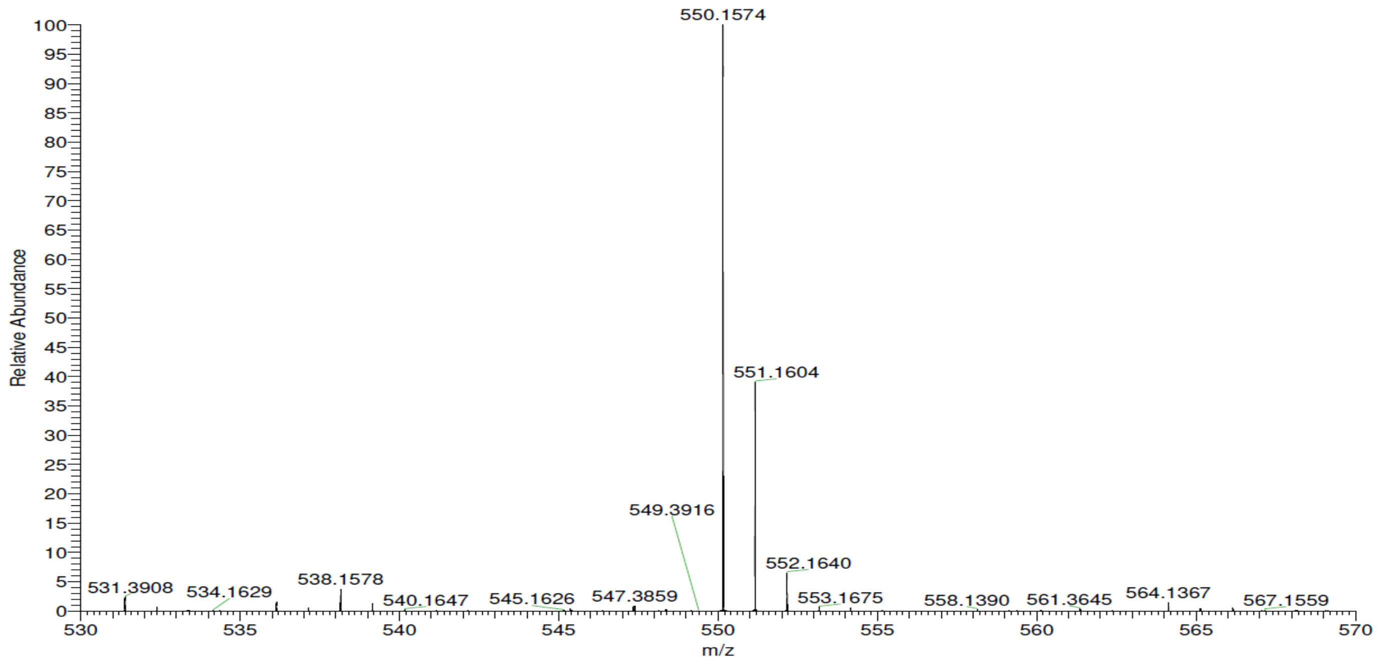

84-148p2-ele-7F-N-H#1-20 RT: 0.00-0.30 AV: 20
T: FTMS - p ESI Full ms [250.00-1000.00]
m/z= 530.0000-570.0000

Isotope	Min	Max
N-14	2	3
O-16	2	4
C-12	28	35
H-1	0	60
Br-79	0	1
F-19	0	1

Charge 1
Mass tolerance 140.00 ppm
Nitrogen rule not used
RDB equiv -1.00-100.00
max results 1

m/z	Intensity	Relative	Theo. Mass	Delta (ppm)	Composition
550.1574	3931640.3	100.00	550.1561	2.29	$C_{35}H_{21}O_{3}N_{3}F$

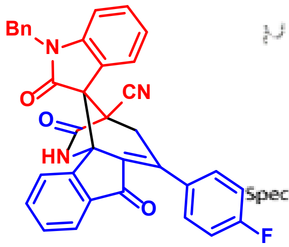

3k

Spectrum

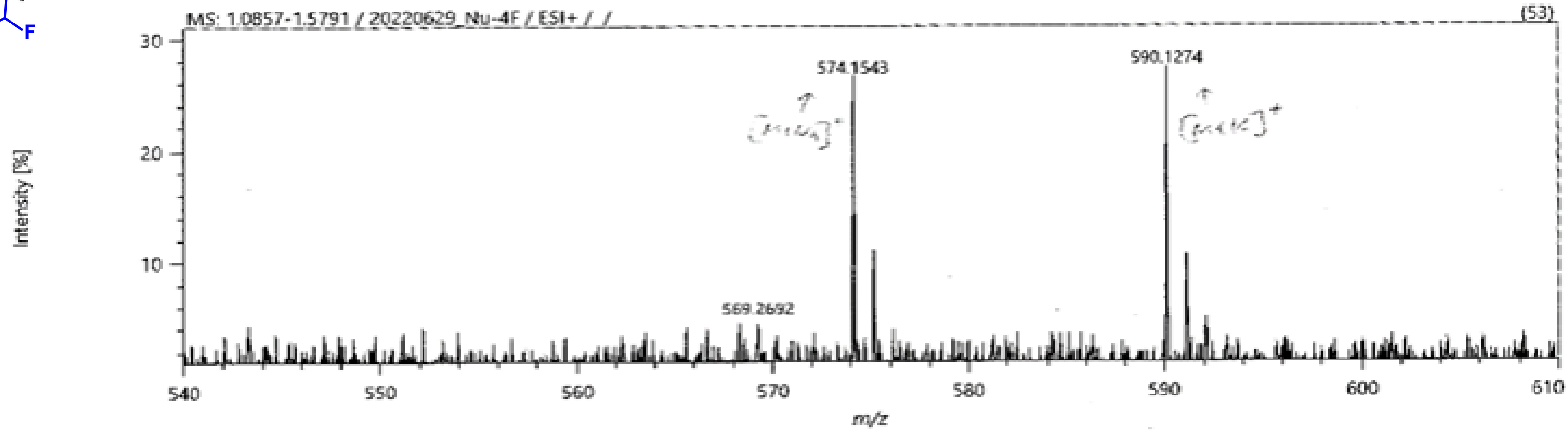

Elemental Composition

Parameters		Elements Set 1:								
Tolerance:	±2.00 ppm	Symbol	C	H	O	N	F	Na	K
Electron:	Odd/Even	Min	0	0	3	3	1	0	0
Charge:	+1	Max	400	1000	3	3	1	1	1
DBE:	-99.0 - 999.0								

Results

Mass	Formula	Calculated Mass	Mass Difference [mDa]	Mass Difference [ppm]	DBE
574.15434	C35 H22 N3 O3 F Na	574.15374	0.60	1.05	25.5
590.12743	C35 H22 N3 O3 F K	590.12768	-0.25	-0.43	25.5

1 / 1

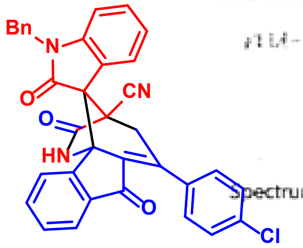

3l

Spectrum

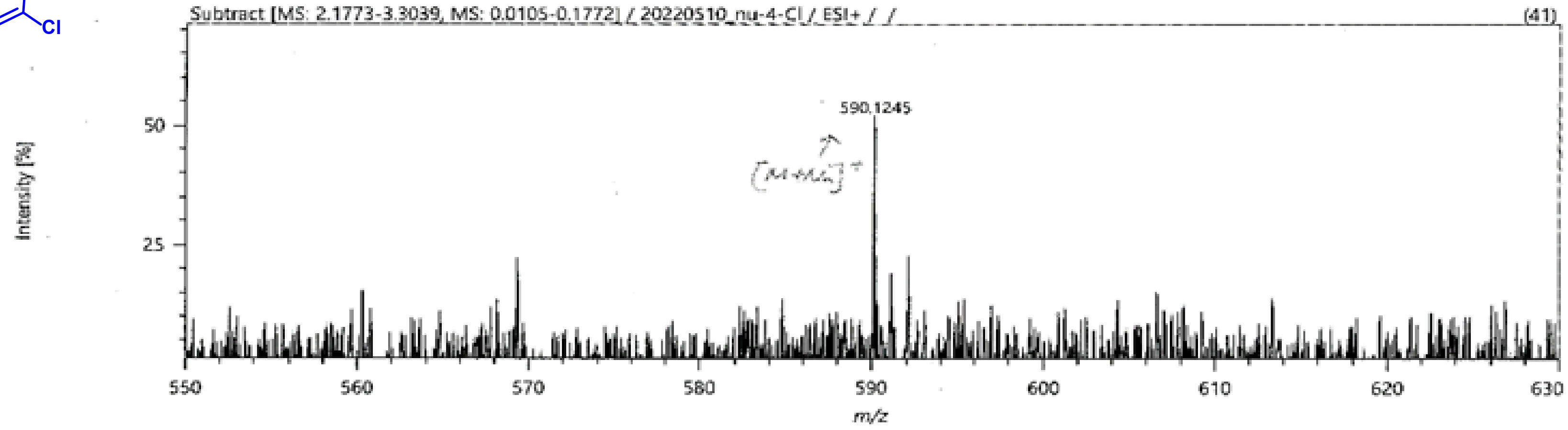

Elemental Composition

Parameters

Tolerance:	±10.00 ppm
Electron:	Odd/Even
Charge:	+1
DBE:	-99.0 - 999.0

Elements Set 1:

Symbol	C	H	O	N	Na	Cl
Min	0	0	3	3	1	1
Max	400	1000	4	3	1	1

Results

Mass	Formula	Calculated Mass	Mass Difference [mDa]	Mass Difference [ppm]	DBE
590.12445	C35 H22 N3 O3 Na Cl	590.12419	0.26	0.44	25.5

1 / 1

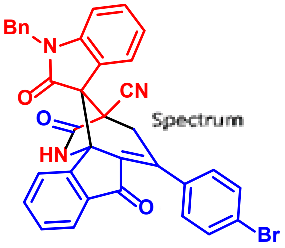

3m

Spectrum

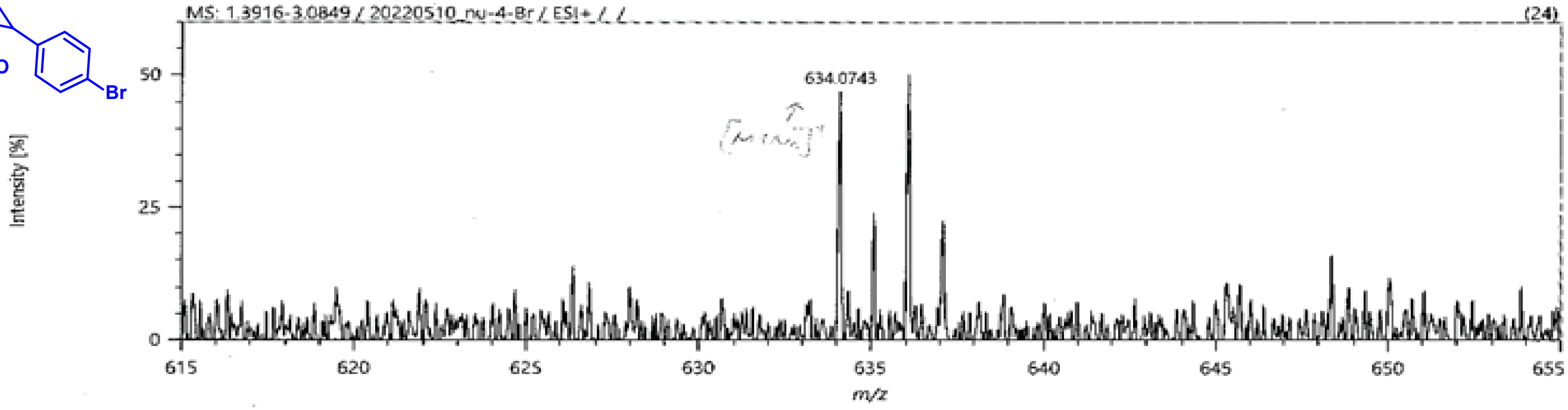

Elemental Composition

Parameters		Elements Set 1:						
Tolerance:	±10.00 ppm	Symbol	C	H	O	N	Na	Br
Electron:	Odd/Even	Min	0	0	3	3	1	1
Charge:	+1	Max	400	1000	4	3	1	1
DBE:	-99.0 - 999.0							

Results

Mass	Formula	Calculated Mass	Mass Difference [mDa]	Mass Difference [ppm]	DBE
634.07434	C35 H22 N3 O3 Na Br	634.07368	0.66	1.05	25.5

1 / 1

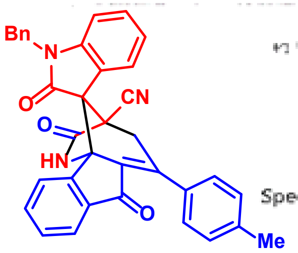

3n

nu-4-Me (HR-ESI)

Spectrum

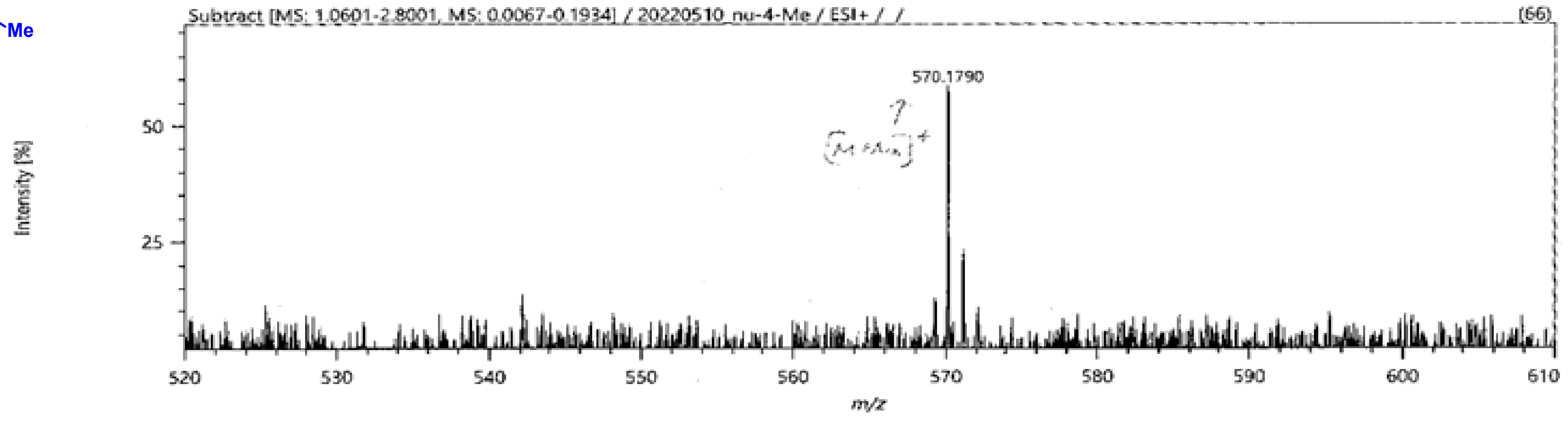

Elemental Composition

Parameters	
Tolerance:	±10.00 ppm
Electron:	Odd/Even
Charge:	+1
DBE:	-99.0 - 999.0

Elements Set 1:

Symbol	C	H	O	N	Na
Min	0	0	3	3	1
Max	400	1000	4	3	1

Results

Mass	Formula	Calculated Mass	Mass Difference [mDa]	Mass Difference [ppm]	DBE
570.17903	C36 H25 N3 O3 Na	570.17881	0.22	0.38	25.5

1 / 1

nu-4-OMe (HR-ESI)

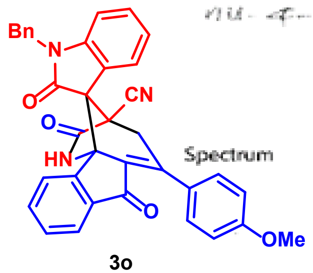

3o

Spectrum

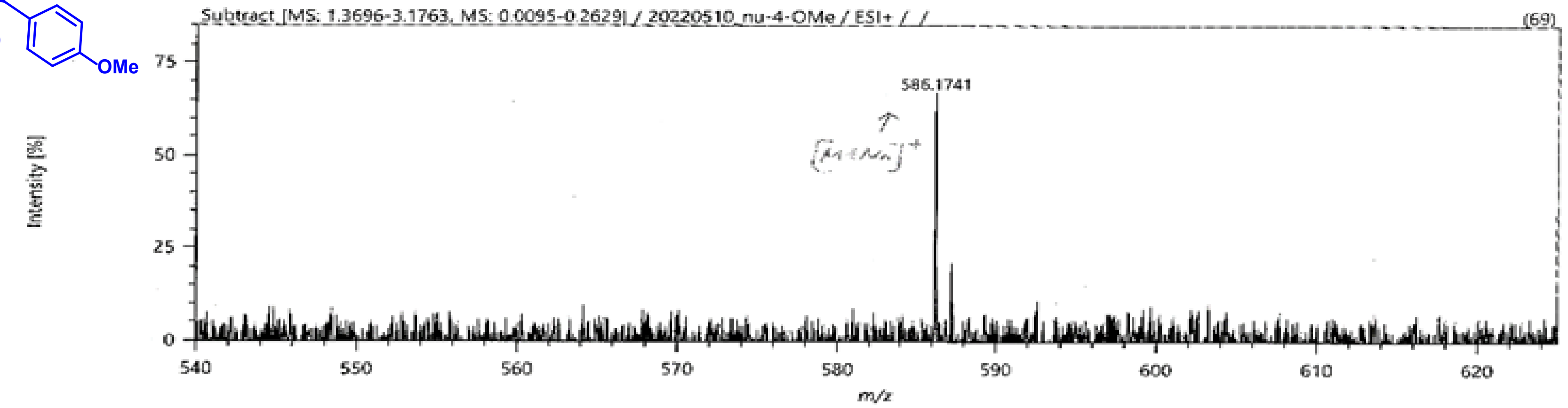

Elemental Composition

Parameters		Elements Set 1:					
Tolerance:	±10.00 ppm	Symbol	C	H	O	N	Na
Electron:	Odd/Even	Min	0	0	3	3	1
Charge:	+1	Max	400	1000	4	3	1
DBE:	-99.0 - 999.0						

Results

Mass	Formula	Calculated Mass	Mass Difference [mDa]	Mass Difference [ppm]	DBE
586.17406	C36 H25 N3 O4 Na	586.17373	0.33	0.57	25.5

1 / 1

nu-2-naph (+HR-ESI)

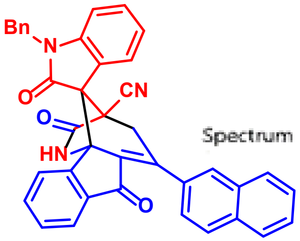

3p

Spectrum

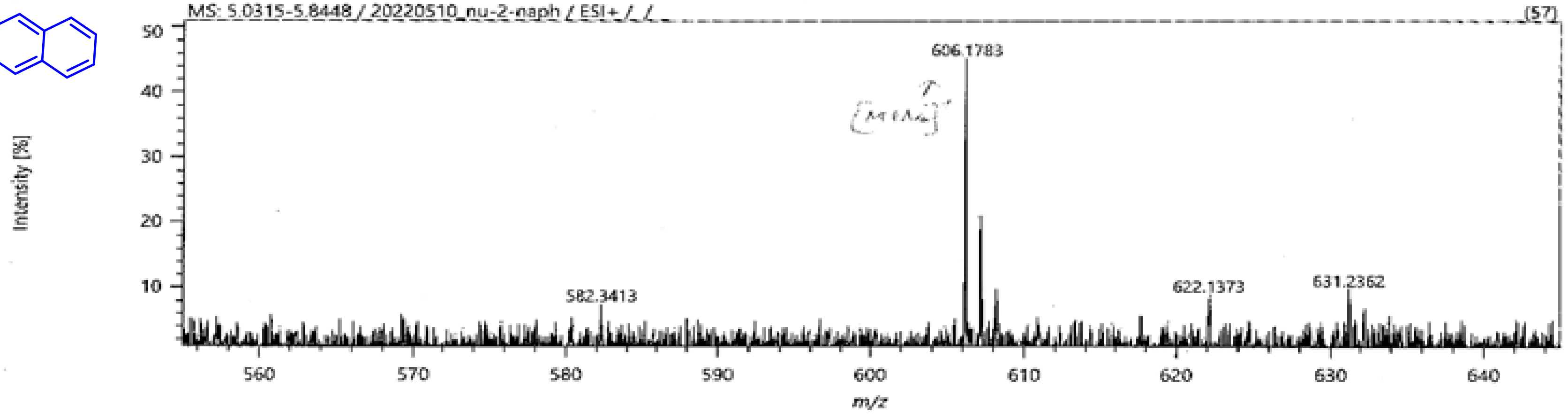

Elemental Composition

Parameters

Tolerance:	±10.00 ppm
Electron:	Odd/Even
Charge:	+1
DBE:	-99.0 - 999.0

Elements Set 1:

Symbol	C	H	O	N	Na
Min	0	0	3	3	1
Max	400	1000	3	3	1

Results

Mass	Formula	Calculated Mass	Mass Difference [mDa]	Mass Difference [ppm]	DBE
606.17833	C39 H25 N3 O3 Na	606.17881	-0.49	-0.80	28.5

1 / 1

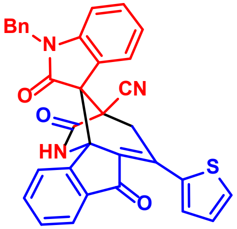

3q

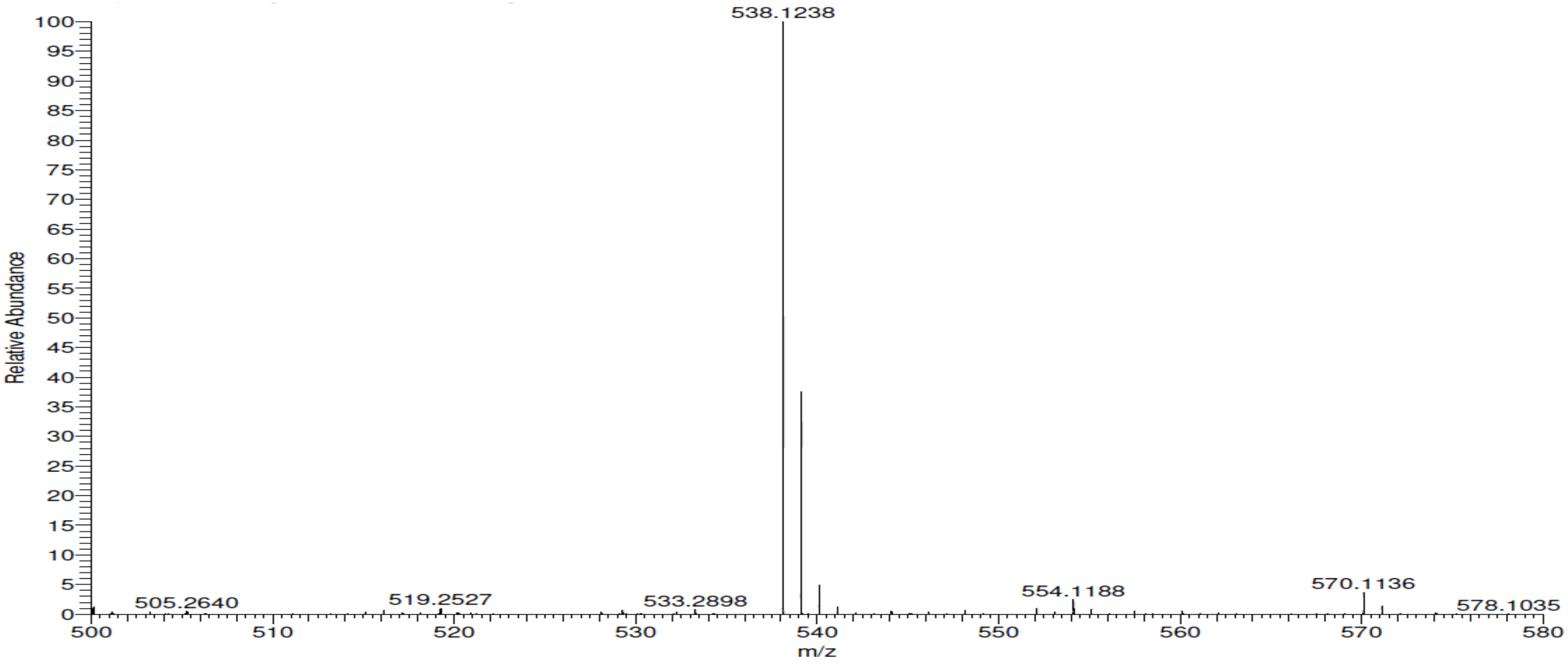

08-148p2-nuc-thienyl-H#1-20 RT: 0.00-0.09 AV: 20
T: FTMS - p ESI Full ms [200.0000-1000.0000]
m/z= 500.0000-580.0000

Isotope	Min	Max
N-14	3	3
O-16	3	3
C-12	33	33
H-1	0	60
S-32	1	1

Charge 1
Mass tolerance 140.00 ppm
Nitrogen rule not used
RDB equiv -1.00-100.00
max results 1

m/z	Intensity	Relative	Theo. Mass	Delta (ppm)	Composition
538.1238	19188414.0	100.00	538.1220	3.40	$C_{33}H_{20}O_3N_3S$

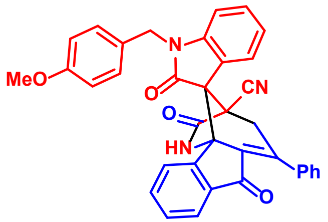

3r

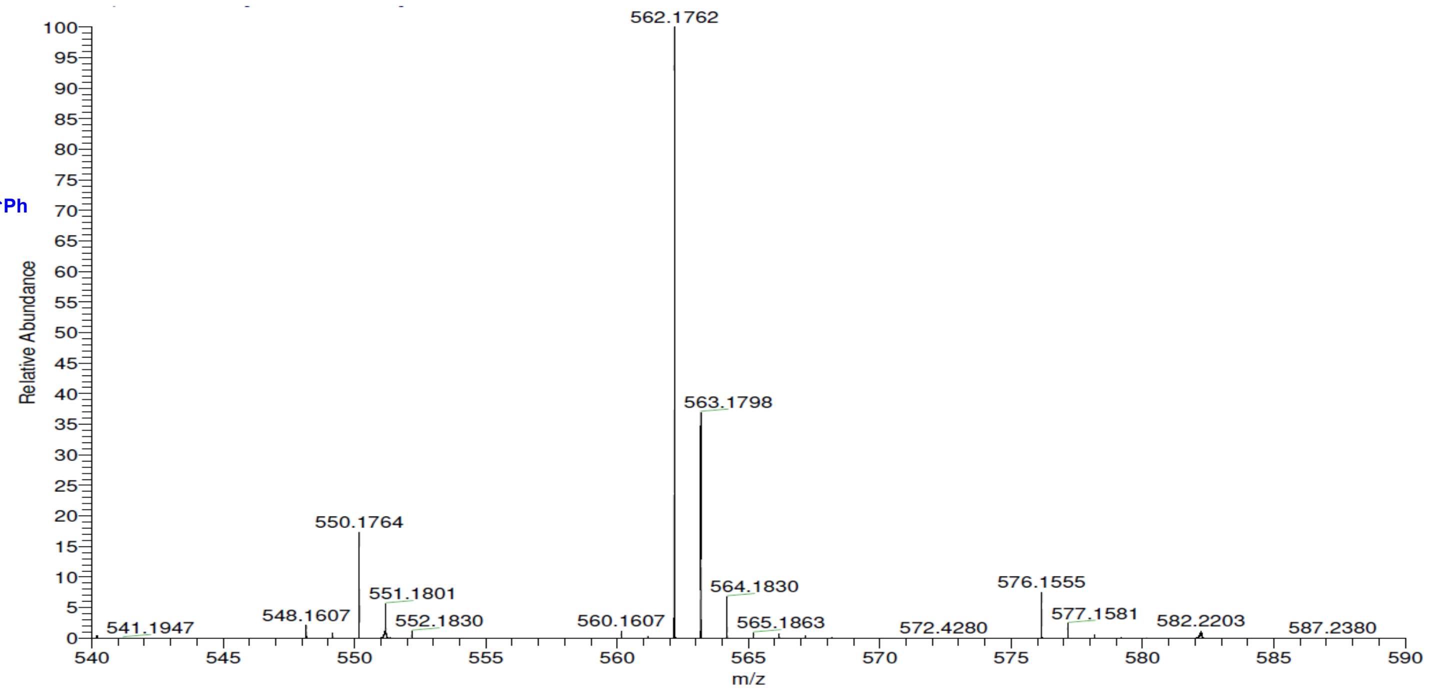

91-148p2-ele-NPMB-N-H#1-20 RT: 0.01-0.31 AV: 20
T: FTMS - p ESI Full ms [250.00-1000.00]
m/z= 540.0000-590.0000

Isotope	Min	Max
N-14	2	3
O-16	2	4
C-12	28	36
H-1	0	60
Br-79	0	1
F-19	0	1

Charge 1
Mass tolerance 140.00 ppm
Nitrogen rule not used
RDB equiv -1.00-100.00
max results 1

m/z	Intensity	Relative	Theo. Mass	Delta (ppm)	Composition
562.1762	440532.0	100.00	562.1761	0.16	$C_{36}H_{24}O_4N_3$

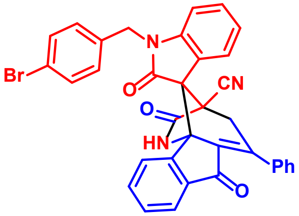

3s

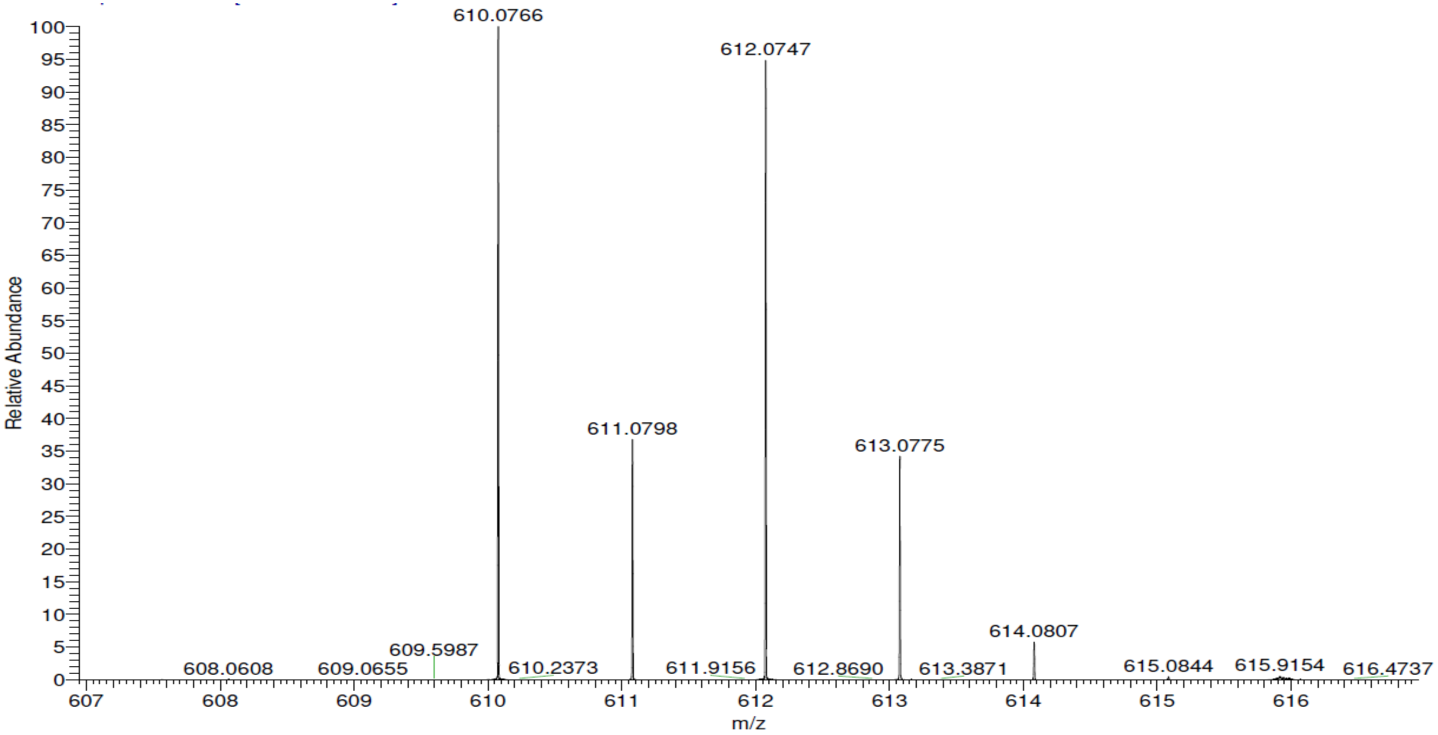

78-148p2-ele-NBn-4Br-N-H#1-20 RT: 0.00-0.55 AV: 20
T: FTMS - p ESI Full ms [250.00-1000.00]
m/z= 606.9480-616.9470

Isotope	Min	Max
N-14	2	3
O-16	2	3
C-12	28	35
H-1	0	60
Br-79	1	1

Charge 1
Mass tolerance 140.00 ppm
Nitrogen rule not used
RDB equiv -1.00-100.00
max results 1

m/z	Intensity	Relative	Theo. Mass	Delta (ppm)	Composition
610.0766	1137431.0	100.00	610.0761	0.87	$C_{35}H_{21}O_3N_3Br$

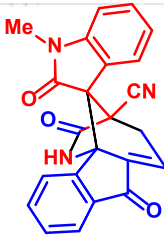

3t

Spectrum

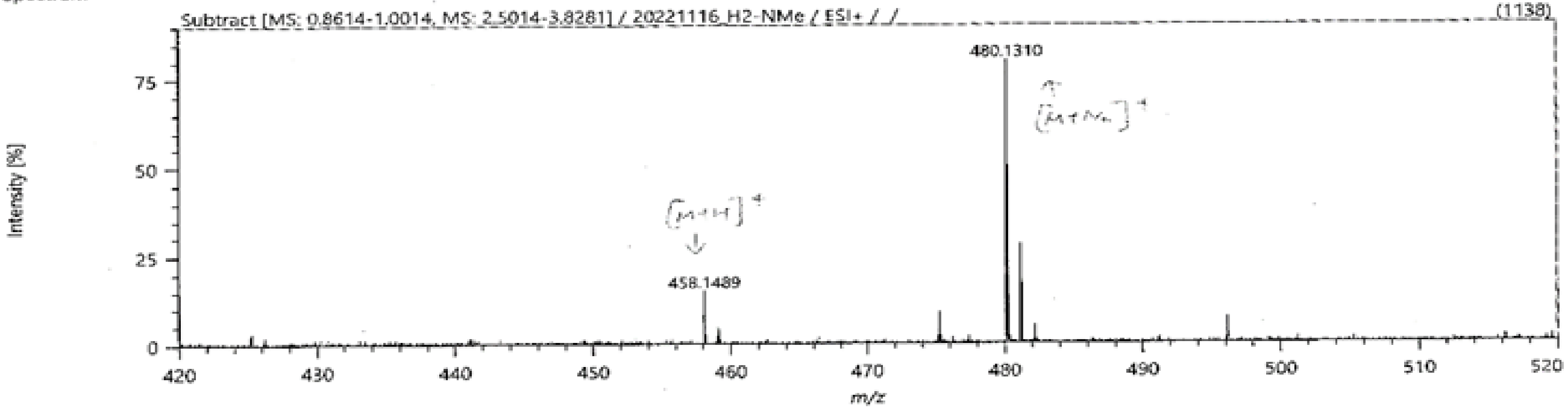

Elemental Composition

Parameters	
Tolerance:	±2.50 ppm
Electron:	Odd/Even
Charge:	+1
DBE:	-99.0 - 999.0

Elements Set 1:

Symbol	C	H	O	N	Na
Min	0	0	3	3	0
Max	400	1000	3	3	1

Results

Mass	Formula	Calculated Mass	Mass Difference [mDa]	Mass Difference [ppm]	DBE
458.14886	C29 H20 N3 O3	458.14992	-1.06	-2.32	21.5
480.13097	C29 H19 N3 O3 Na	480.13186	-0.89	-1.86	21.5

1 / 1

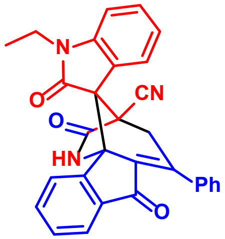

3u

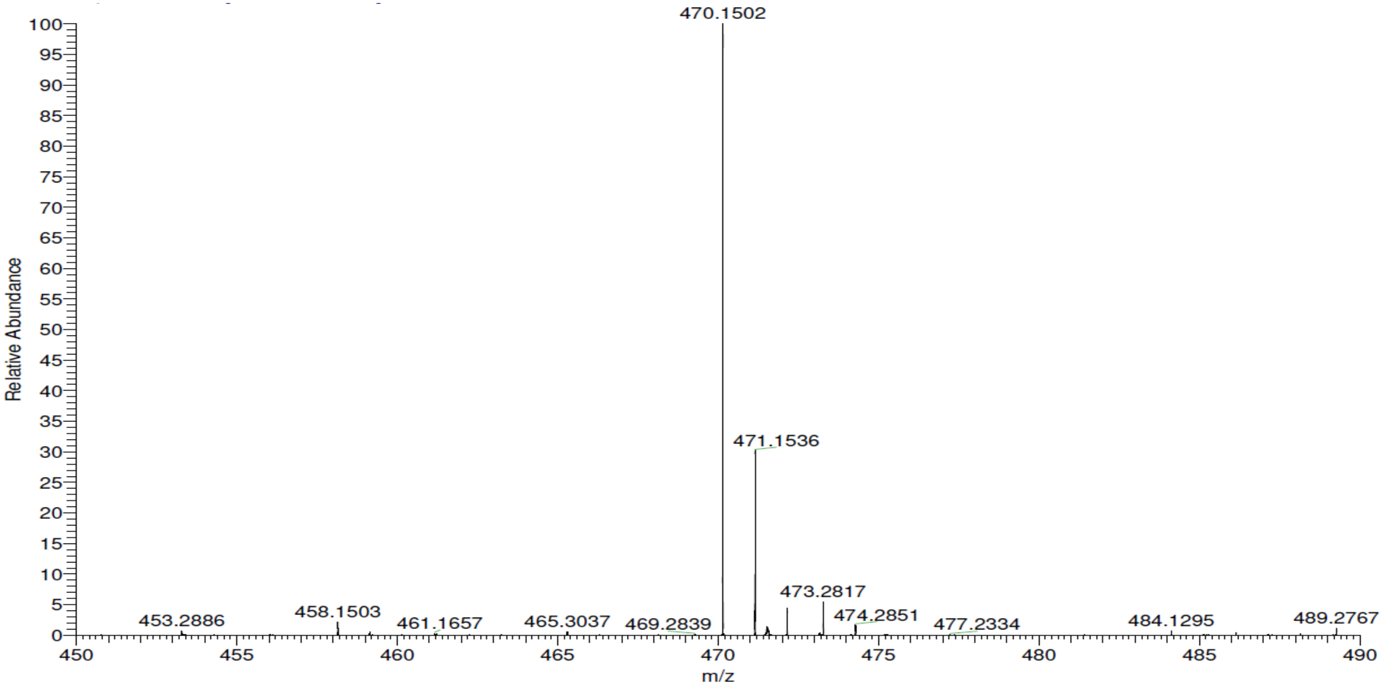

86-148p2-ele-NEt-N-H#1-20 RT: 0.01-0.31 AV: 20
T: FTMS - p ESI Full ms [250.00-1000.00]
m/z= 450.0000-490.0000

Isotope	Min	Max
N-14	2	3
O-16	2	4
C-12	28	30
H-1	0	60
Br-79	0	1
F-19	0	1

Charge 1
Mass tolerance 140.00 ppm
Nitrogen rule not used
RDB equiv -1.00-100.00
max results 1

m/z	Intensity	Relative	Theo. Mass	Delta (ppm)	Composition
470.1502	799334.6	100.00	470.1499	0.53	$C_{30}H_{20}O_3N_3$

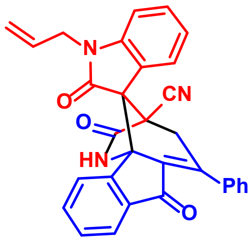

3v

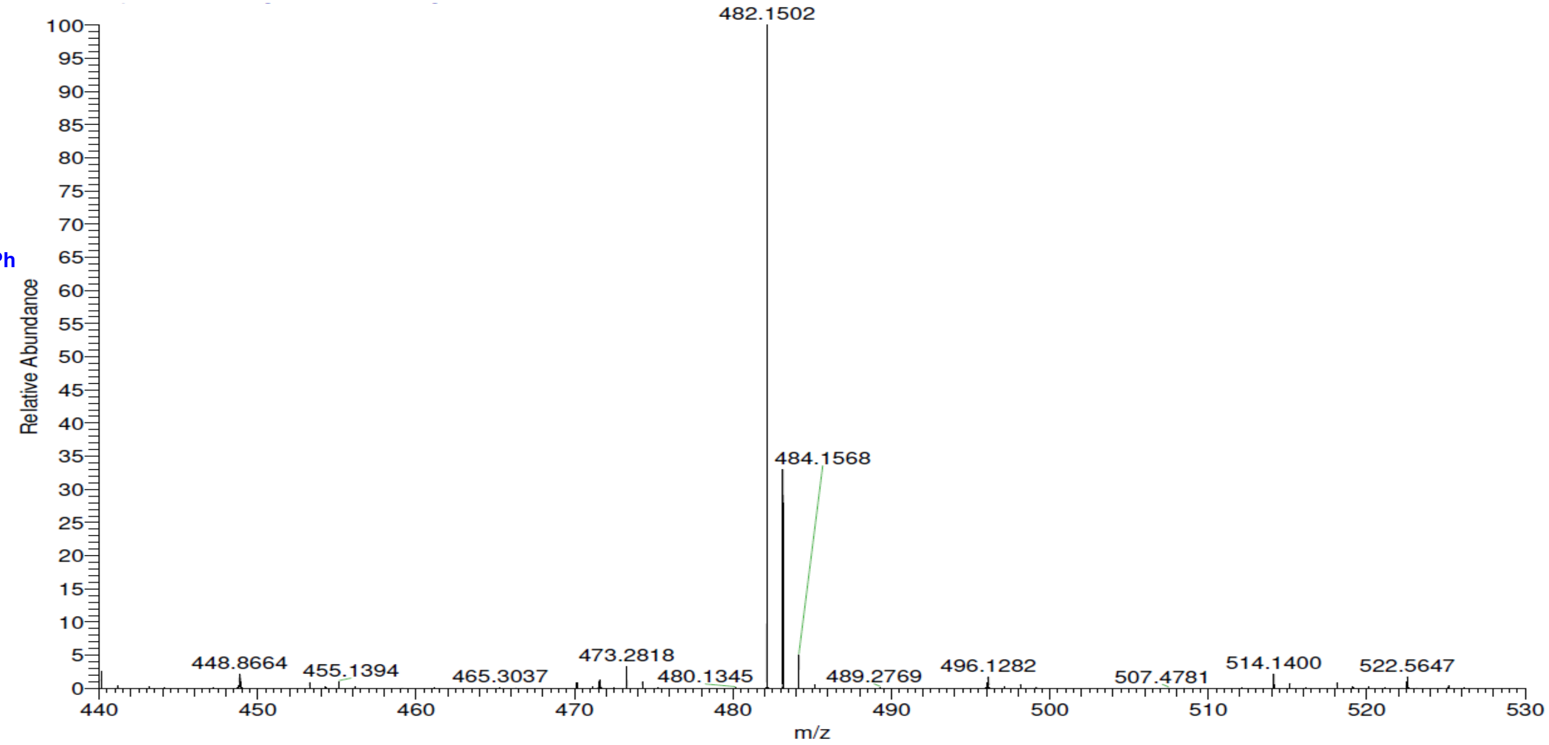

88-148p2-ele-NAllyl-N-H#1-20 RT: 0.01-0.31 AV: 20
T: FTMS - p ESI Full ms [250.00-1000.00]
m/z= 440.0000-530.0000

Isotope	Min	Max
N-14	2	3
O-16	2	4
C-12	28	31
H-1	0	60
Br-79	0	1
F-19	0	1

Charge 1
Mass tolerance 140.00 ppm
Nitrogen rule not used
RDB equiv -1.00-100.00
max results 1

m/z	Intensity	Relative	Theo. Mass	Delta (ppm)	Composition
482.1502	620263.9	100.00	482.1499	0.53	$C_{31}H_{20}O_3N_3$

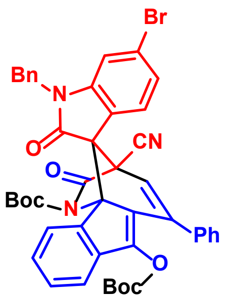

4

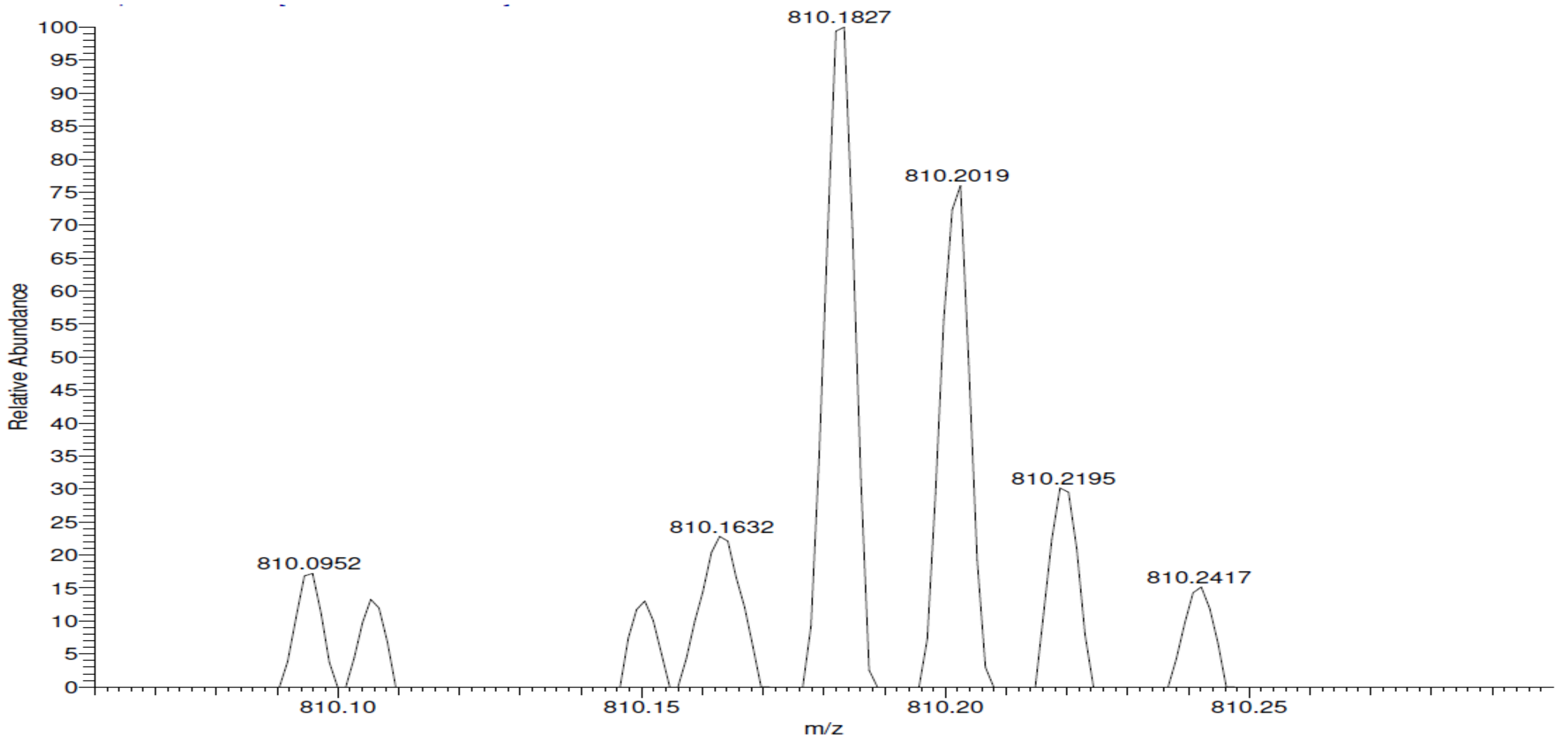

68-148p2-6Br-Boc-N-H#1-10 RT: 0.04-0.39 AV: 10
T: FTMS - p ESI SIM ms [800.0000-825.0000]
m/z= 810.0600-810.3000

Isotope	Min	Max
N-14	3	3
O-16	7	7
C-12	45	45
H-1	0	60
S-32	0	3
Br-79	1	1
F-19	0	3
Cl-35	0	0

Charge 1
Mass tolerance 140.00 ppm
Nitrogen rule not used
RDB equiv -1.00-100.00
max results 1

m/z	Intensity	Relative	Theo. Mass	Delta (ppm)	Composition
810.1827	141.0	100.00	810.1809	2.14	$C_{45}H_{37}O_7N_3Br$

13. Cartesian Coordinates

Int-5

Optimization energy :

E(B3LYP/6-311G(d,p)/IEFPCM(THF)) = -2083.572147 Hartree

Single point energy :

E(M06-2X-D3/def2-TZVPP/SMD(THF))= -2082.804670 Hartree

Thermal correction to Gibbs Free Energy= 0.0 kcal/mol

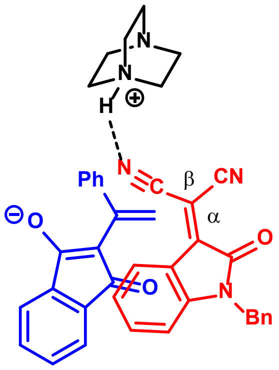

Symbol	X	Y	Z
C	5.0980580	-0.8792990	-1.1182450
C	4.0482030	-1.2331640	-0.2901230
C	3.7942320	-2.5673340	0.0277970
C	4.5833290	-3.5880920	-0.4683030
C	5.6556940	-3.2405580	-1.3019230
C	5.9091800	-1.9057070	-1.6219450
H	5.2814910	0.1588140	-1.3681530
H	4.3684930	-4.6215550	-0.2247920
H	6.2930960	-4.0167410	-1.7093180
H	6.7418560	-1.6626740	-2.2719320
C	2.5741260	-2.6253070	0.9051550
C	3.0088460	-0.3741820	0.3659820
C	2.1216830	-1.2449300	1.0924790
O	2.9630110	0.8583040	0.2102910
C	0.9851120	-0.8606450	1.8922160
C	-0.0293220	-1.7370580	2.1234450
H	-0.8609400	-1.4722190	2.7638910
C	0.9167600	0.4884620	2.5210930
C	2.0654100	1.0904800	3.0522610
C	-0.3059780	1.1545370	2.6742220
C	1.9937960	2.3155880	3.7066000
H	3.0179510	0.5863940	2.9564450
C	-0.3815610	2.3824850	3.3251970
H	-1.2041350	0.7120180	2.2675130

C	0.7699290	2.9723140	3.8419470
H	2.8952040	2.7591160	4.1139500
H	-1.3393350	2.8813790	3.4208150
H	0.7158450	3.9296660	4.3469240
H	0.0278460	-2.7519290	1.7692340
O	2.0978910	-3.6699990	1.3424110
C	-3.0585520	-4.6532280	0.3293770
C	-1.9275770	-4.0159350	-0.1815090
C	-2.9704420	-1.8895090	0.3543950
C	-4.0961550	-2.5125750	0.8607290
C	-4.1246710	-3.9121390	0.8415310
H	-3.1070160	-5.7347800	0.3299920
H	-1.0997980	-4.5964450	-0.5641140
H	-4.9297290	-1.9433750	1.2490440
H	-4.9948040	-4.4258290	1.2319940
C	-1.8798030	-2.6228340	-0.1711120
C	-0.8941880	-1.6546660	-0.6108760
C	0.2670990	-1.8134630	-1.3263310
C	0.9836890	-0.7288270	-1.9042240
C	0.7650910	-3.1003660	-1.6671320
N	1.5969310	0.0859090	-2.4500570
N	1.1689830	-4.1442720	-1.9557500
C	-1.4607350	-0.2992470	-0.3032710
N	-2.6937570	-0.5181950	0.2763220
O	-0.9680230	0.7941570	-0.5384440
C	-3.5868990	0.5454740	0.7031660
H	-3.8520520	0.3918360	1.7522500
H	-3.0062290	1.4677930	0.6389220
C	-4.8370870	0.6429380	-0.1458020
C	-6.0817420	0.8323280	0.4546080
C	-4.7565400	0.5637140	-1.5380960
C	-7.2321710	0.9487520	-0.3239690
H	-6.1531970	0.8842510	1.5358650
C	-5.9041450	0.6754070	-2.3169160
H	-3.7943550	0.4086310	-2.0126500
C	-7.1456570	0.8696000	-1.7115830
H	-8.1940650	1.0925330	0.1540500
H	-5.8307910	0.6097640	-3.3961610
H	-8.0393710	0.9535860	-2.3184980
C	0.6871880	3.0606050	-2.1438260

C	0.3033260	4.5666480	-2.1525840
H	1.2205150	2.7498950	-3.0392500
H	-0.1596690	2.3979600	-1.9908880
H	0.5148700	5.0049250	-3.1286320
H	-0.7623350	4.6886800	-1.9541950
C	2.8837330	3.6268910	-1.1871220
H	3.5089980	3.4307020	-0.3192140
H	3.3740270	3.2192790	-2.0696580
C	2.4911990	5.1220680	-1.3436990
H	3.0368880	5.7325110	-0.6233060
H	2.7399070	5.4800840	-2.3435250
H	1.8764590	1.8580380	-0.8746880
C	0.6889900	4.7985370	0.2024520
H	1.2758150	5.3355530	0.9491010
H	-0.3641480	5.0146070	0.3869600
C	0.9502370	3.2746160	0.2950610
H	1.6192730	2.9935340	1.1025520
H	0.0444850	2.6828040	0.3759900
N	1.6156040	2.8515740	-0.9846140
N	1.0502110	5.3117300	-1.1283730

TS1

Optimization energy :

E(B3LYP/6-311G(d,p)/IEFPCM(THF)) = -2083.551383 Hartree

Number of imaginary frequency=1(-140.17)

Single point energy :

E(M06-2X-D3/def2-TZVPP/SMD(THF))= -2082.803005 Hartree

Thermal correction to Gibbs Free Energy= 12.2 kcal/mol

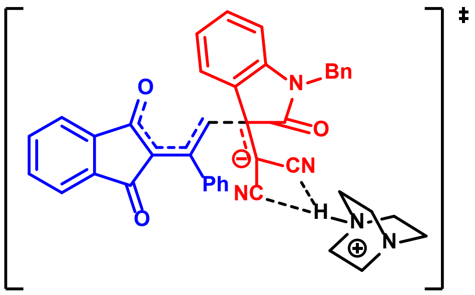

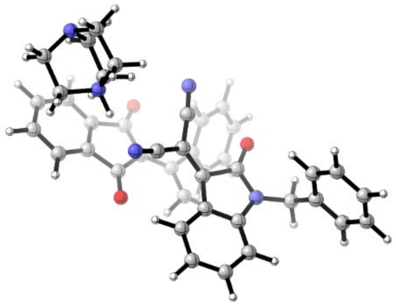

Symbol	X	Y	Z
C	4.7735470	2.1099160	0.2712110
C	3.4511410	1.9560330	0.6598520
C	3.0962420	1.0968610	1.6971410
C	4.0519780	0.3646690	2.3857870
C	5.3889900	0.5134050	2.0017250
C	5.7450650	1.3728880	0.9555210
H	5.0370760	2.7812280	-0.5369440
H	3.7635270	-0.2976150	3.1928910
H	6.1633040	-0.0393220	2.5209420
H	6.7881030	1.4692190	0.6785960
C	1.6118990	1.1365960	1.8860530
C	2.2161340	2.6117870	0.1148340
C	1.0705700	2.0550720	0.8667370
O	2.2412330	3.4468220	-0.7758910
C	-0.2787620	2.4112780	0.7547310
C	-1.2474010	1.7304310	1.5189770
H	-2.2713110	2.0794430	1.4712350
C	-0.7845210	3.4154360	-0.2203810
C	-1.6355670	4.4276390	0.2441450
C	-0.5040090	3.3492970	-1.5898570
C	-2.1772190	5.3645770	-0.6320360
H	-1.8581490	4.4898800	1.3030120
C	-1.0605700	4.2726560	-2.4664320
H	0.1282440	2.5616970	-1.9681570
C	-1.8935810	5.2863420	-1.9931920

H	-2.8207960	6.1494200	-0.2515730
H	-0.8466280	4.1976530	-3.5264140
H	-2.3206920	6.0068670	-2.6813880
H	-0.9365150	1.2363400	2.4238530
O	1.0353820	0.5094310	2.7705570
C	-2.4813350	-2.1273740	3.5936400
C	-1.5750030	-1.5557440	2.6952830
C	-3.4590790	-0.7848720	1.3843430
C	-4.3678110	-1.3416820	2.2706810
C	-3.8557180	-2.0218470	3.3811300
H	-2.1108730	-2.6489050	4.4676520
H	-0.5107870	-1.5984830	2.8810550
H	-5.4342920	-1.2646300	2.1067830
H	-4.5440230	-2.4694450	4.0882780
C	-2.0675290	-0.8968530	1.5769220
C	-1.4073380	-0.1736440	0.4743210
C	-0.1797500	-0.5732480	-0.1188700
C	0.2706590	-0.1408060	-1.3878370
C	0.6890230	-1.4983200	0.4747130
N	0.7400060	0.1382350	-2.4129020
N	1.4538980	-2.2704710	0.8900150
C	-2.5331240	0.2959660	-0.4209820
N	-3.7126210	-0.0539610	0.2179380
O	-2.4577540	0.8437440	-1.5039420
C	-5.0288400	0.2345510	-0.3229000
H	-5.6282160	0.7413570	0.4377310
H	-4.8657700	0.9366200	-1.1429720
C	-5.7464700	-1.0043660	-0.8175150
C	-7.0908230	-1.2134180	-0.5100430
C	-5.0734160	-1.9454700	-1.6008350
C	-7.7584500	-2.3428980	-0.9817030
H	-7.6176930	-0.4915830	0.1051580
C	-5.7363650	-3.0753250	-2.0696190
H	-4.0279620	-1.7917410	-1.8422070
C	-7.0819070	-3.2772320	-1.7616710
H	-8.8024400	-2.4949910	-0.7334110
H	-5.2039160	-3.7989180	-2.6759570
H	-7.5970360	-4.1586240	-2.1252220
C	3.7604760	-3.5785380	-1.5905880
C	5.0553580	-3.4728560	-2.4470420

H	3.6461410	-4.5477500	-1.1081620
H	2.8565660	-3.3556180	-2.1552760
H	5.6273400	-4.3990190	-2.3856840
H	4.8011710	-3.3020370	-3.4930420
C	5.0879710	-2.7892460	0.3314310
H	5.0658300	-2.0683330	1.1453620
H	5.0000070	-3.7935310	0.7428440
C	6.3224400	-2.6191680	-0.5963740
H	6.9324170	-1.7789480	-0.2636420
H	6.9404290	-3.5168690	-0.5709950
H	3.0218980	-2.5941940	0.1029810
C	5.1486660	-1.1101020	-2.0420010
H	5.8033350	-0.2915030	-1.7429070
H	4.8426870	-0.9379550	-3.0740070
C	3.9091640	-1.1674670	-1.1111850
H	3.9646190	-0.4628610	-0.2877670
H	2.9721110	-1.0120410	-1.6415280
N	3.8637330	-2.5455900	-0.5047890
N	5.9039870	-2.3688310	-1.9810630

Int-13

Optimization energy :

E(B3LYP/6-311G(d,p)/IEFPCM(THF)) = -2083.566858 Hartree

Single point energy :

E(M06-2X-D3/def2-TZVPP/SMD(THF))= -2082.827461 Hartree

Thermal correction to Gibbs Free Energy= -3.1 kcal/mol

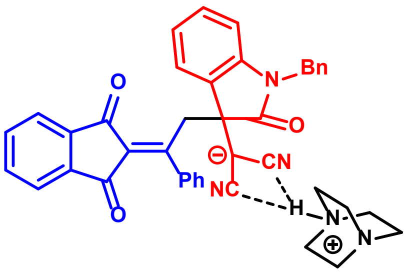

Symbol	X	Y	Z
C	5.1926540	1.7825320	-0.0696630
C	3.8745860	1.7464200	0.3681260
C	3.4830340	0.9106230	1.4117780
C	4.3993170	0.1006140	2.0716490
C	5.7237160	0.1282310	1.6352190
C	6.1142340	0.9554410	0.5720250
H	5.4861160	2.4347390	-0.8827610
H	4.0850820	-0.5275940	2.8961500
H	6.4645430	-0.4936790	2.1232140
H	7.1491830	0.9536470	0.2521450
C	2.0153080	1.0378670	1.6385530
C	2.6982430	2.5260790	-0.1223210
C	1.5103400	2.0430950	0.6530350
O	2.7638880	3.3860010	-0.9784650
C	0.2278520	2.5037040	0.5809380
C	-0.8312950	1.8779870	1.4355360
H	-1.6380920	2.5883940	1.6177320
C	-0.2104370	3.5909770	-0.3221030
C	-0.9187190	4.6736120	0.2182410
C	0.0093380	3.5460710	-1.7034050
C	-1.3708090	5.7042170	-0.5996730
H	-1.0957970	4.7233680	1.2860770
C	-0.4705410	4.5604640	-2.5210670
H	0.5129160	2.6932910	-2.1321370
C	-1.1534230	5.6466840	-1.9742280
H	-1.8987870	6.5444400	-0.1642200
H	-0.3142740	4.5014970	-3.5918730

H	-1.5198530	6.4391030	-2.6165720
H	-0.4069760	1.5684740	2.3846410
O	1.4146400	0.4249430	2.5063260
C	-2.5553480	-1.5226430	3.8601810
C	-1.6826050	-0.7982640	3.0371980
C	-3.5371390	-0.3493620	1.5612680
C	-4.4153230	-1.0695790	2.3581060
C	-3.8995900	-1.6570320	3.5190820
H	-2.1802300	-1.9851670	4.7650940
H	-0.6340410	-0.6958930	3.2860650
H	-5.4580340	-1.1813310	2.0916300
H	-4.5622630	-2.2261990	4.1605300
C	-2.1814960	-0.2211030	1.8835230
C	-1.4874950	0.5794670	0.8003660
C	-0.5789230	-0.3192330	-0.0279210
C	-0.1600180	0.0095830	-1.3213590
C	-0.0712260	-1.5158910	0.4398250
N	0.2423370	0.2270620	-2.3948240
N	0.4065320	-2.5396460	0.7548300
C	-2.6755100	0.9704310	-0.1146220
N	-3.8071780	0.3491660	0.3766800
O	-2.6398040	1.6733290	-1.1045340
C	-5.0899470	0.3738450	-0.2994730
H	-5.8591010	0.7380950	0.3871530
H	-4.9885170	1.1059170	-1.1036010
C	-5.4826490	-0.9803300	-0.8558140
C	-6.8194700	-1.3804950	-0.8477580
C	-4.5200920	-1.8344840	-1.3992680
C	-7.1938610	-2.6115890	-1.3829820
H	-7.5710860	-0.7275890	-0.4163810
C	-4.8917150	-3.0662980	-1.9303740
H	-3.4771410	-1.5395330	-1.3983290
C	-6.2298470	-3.4582530	-1.9256040
H	-8.2353700	-2.9114150	-1.3684370
H	-4.1352910	-3.7216840	-2.3463630
H	-6.5176710	-4.4187240	-2.3368570
C	2.7479480	-4.0868560	-1.3917980
C	4.1076350	-4.1730450	-2.1427890
H	2.5075860	-4.9947190	-0.8409380
H	1.9144890	-3.8433820	-2.0485390

H	4.5933060	-5.1284710	-1.9421670
H	3.9492920	-4.0947730	-3.2184710
C	3.9965430	-3.2360830	0.5503610
H	3.9762250	-2.4517260	1.3036690
H	3.7988390	-4.1925700	1.0317490
C	5.3054880	-3.2376140	-0.2868580
H	5.9492010	-2.4110720	0.0155950
H	5.8528900	-4.1675540	-0.1307510
H	1.9631150	-2.8994720	0.1525520
C	4.3630860	-1.7958060	-1.9541880
H	5.0629630	-1.0007870	-1.6971710
H	4.1323690	-1.7119020	-3.0163890
C	3.0699680	-1.6646920	-1.1057760
H	3.1494970	-0.8989760	-0.3400970
H	2.1823450	-1.4645420	-1.7020460
N	2.8604650	-2.9725740	-0.3932960
N	5.0095440	-3.0943120	-1.7180030

TS2

Optimization energy :

E(B3LYP/6-311G(d,p)/IEFPCM(THF)) = -2083.555719 Hartree

Number of imaginary frequency=1(-358.58)

Single point energy :

E(M06-2X-D3/def2-TZVPP/SMD(THF))= -2082.80467 Hartree

Thermal correction to Gibbs Free Energy= 11.2 kcal/mol

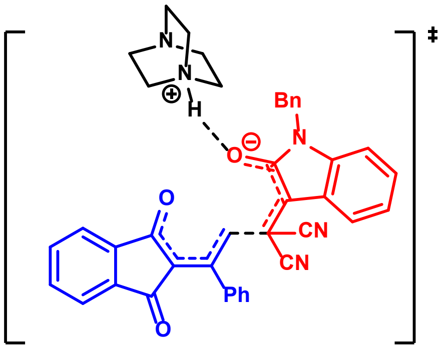

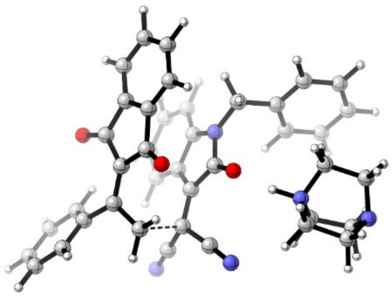

Symbol	X	Y	Z
C	2.3756010	3.9023330	-1.1133010
C	2.3543360	2.5345140	-1.3483030
C	1.3914110	1.9572460	-2.1720110
C	0.4312870	2.7300220	-2.8105310
C	0.4419040	4.1050780	-2.5754700
C	1.4003000	4.6843840	-1.7321660
H	3.1263680	4.3389850	-0.4664420
H	-0.3047670	2.2699310	-3.4582440
H	-0.3009630	4.7374890	-3.0472820
H	1.3832820	5.7549610	-1.5659230
C	1.5910020	0.4798990	-2.2123840
C	3.2809120	1.4770560	-0.8440670
C	2.7251690	0.1720180	-1.2977560
O	4.2787130	1.7103460	-0.1832410
C	3.1592940	-1.1101930	-0.9950550
C	2.2413400	-2.1888850	-1.2046960
C	4.4442510	-1.4705150	-0.3338370
C	5.1898880	-2.5165280	-0.9018100
C	4.9217860	-0.8771180	0.8402840
C	6.3803690	-2.9458580	-0.3242200
H	4.8409650	-2.9839580	-1.8149720
C	6.1004820	-1.3215340	1.4294040
H	4.3691360	-0.0715050	1.2920140
C	6.8375520	-2.3526810	0.8501770

H	6.9456470	-3.7451200	-0.7891490
H	6.4434240	-0.8586260	2.3475480
H	7.7585730	-2.6913090	1.3102850
O	0.9558990	-0.2700610	-2.9421850
C	2.3884560	1.5311030	3.1816690
C	2.1150860	0.3614850	2.4720900
C	0.3856130	1.5732080	1.2873450
C	0.6470980	2.7354890	1.9958260
C	1.6693170	2.7060070	2.9467920
H	3.1715130	1.5236350	3.9306890
H	2.6723860	-0.5382540	2.6859030
H	0.0767510	3.6388720	1.8198800
H	1.8992440	3.6022310	3.5102300
C	1.1132390	0.3690930	1.4967910
C	0.5578910	-0.6173090	0.5924740
C	0.9558310	-1.9821370	0.3693760
C	-0.0614880	-2.8940600	-0.1066990
C	1.7402880	-2.6126290	1.4075630
N	-0.8292630	-3.6769580	-0.4668250
N	2.3781870	-3.1255500	2.2205390
C	-0.5172380	0.0156820	-0.1233240
N	-0.5718250	1.3389200	0.3052960
O	-1.2991420	-0.4431570	-0.9889970
C	-1.5906110	2.2605730	-0.1378710
H	-1.1820150	3.2724130	-0.0997860
H	-1.7928350	2.0398620	-1.1863070
C	-2.8855030	2.1701960	0.6482520
C	-4.0210620	2.8264320	0.1637480
C	-2.9876080	1.4204070	1.8200360
C	-5.2396650	2.7207810	0.8276610
H	-3.9539230	3.4074250	-0.7502070
C	-4.2087560	1.3116210	2.4866620
H	-2.1154060	0.9100510	2.2089240
C	-5.3392530	1.9558010	1.9904680
H	-6.1134730	3.2268270	0.4341950
H	-4.2740530	0.7203280	3.3926260
H	-6.2886300	1.8662380	2.5046450
C	-4.6431810	-0.5792390	-0.8861000
C	-6.0468450	-1.2275150	-0.7559680
H	-4.4647230	0.1866130	-0.1376380

H	-4.4556590	-0.1553590	-1.8712260
H	-6.6551860	-0.6603080	-0.0506140
H	-6.5615630	-1.2326580	-1.7175380
C	-3.7164500	-2.1677810	0.7465320
H	-2.9912160	-2.9735890	0.8361880
H	-3.4251960	-1.3514680	1.4044120
C	-5.1782660	-2.6353860	0.9765390
H	-5.1909990	-3.6477410	1.3812780
H	-5.6816820	-1.9803910	1.6888960
H	-2.6655400	-1.2399060	-0.8052340
C	-5.2254030	-3.4160190	-1.2906570
H	-5.0959210	-4.4244520	-0.8957150
H	-5.8420100	-3.4832900	-2.1875210
C	-3.8469090	-2.7837700	-1.6304210
H	-3.0148530	-3.4743280	-1.5161170
H	-3.8160720	-2.3521180	-2.6299540
N	-3.6236860	-1.6582100	-0.6629240
N	-5.9357150	-2.6144530	-0.2831660
H	1.4976040	-2.0641320	-1.9776080
H	2.6053740	-3.1982320	-1.0535300

Int-6

Optimization energy :

E(B3LYP/6-311G(d,p)/IEFPCM(THF)) = -2083.566086 Hartree

Single point energy :

E(M06-2X-D3/def2-TZVPP/SMD(THF))= -2082.826435 Hartree

Thermal correction to Gibbs Free Energy= -2.5 kcal/mol

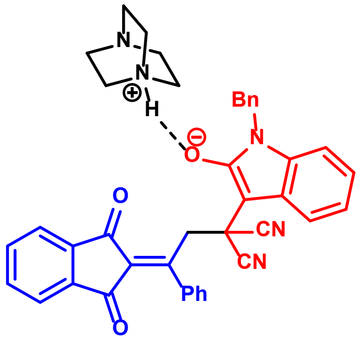

Symbol	X	Y	Z
C	1.298107	-2.74611	3.331636
C	1.413816	-1.54323	2.647521
C	0.323962	-0.68353	2.501985
C	-0.91206	-0.9898	3.056421
C	-1.03934	-2.20278	3.729433
C	0.052594	-3.07361	3.861743
H	2.150546	-3.40524	3.436749
H	-1.7497	-0.31296	2.946591
H	-1.9944	-2.48251	4.15803
H	-0.07848	-4.01106	4.388568
C	0.729056	0.507195	1.716955
C	2.616395	-0.94575	2.005162
C	2.121737	0.240178	1.227148
O	3.750488	-1.36448	2.126855
C	2.729691	0.892803	0.201335
C	1.870148	1.766765	-0.65769
C	4.131709	0.723142	-0.25208
C	4.86909	1.878809	-0.55865
C	4.742094	-0.52386	-0.43823
C	6.178121	1.791566	-1.01969
H	4.424373	2.855723	-0.41262
C	6.040256	-0.61014	-0.92548
H	4.197217	-1.42598	-0.2158
C	6.766285	0.544362	-1.21352

H	6.733047	2.697074	-1.23421
H	6.486057	-1.58527	-1.0819
H	7.7803	0.471824	-1.58853
O	0.071647	1.538658	1.629781
C	1.900314	-3.72808	-0.8195
C	1.840216	-2.38412	-1.19161
C	-0.42427	-2.32704	-0.3352
C	-0.37097	-3.66379	0.029087
C	0.813425	-4.36394	-0.21179
H	2.808734	-4.28778	-1.01183
H	2.687339	-1.9269	-1.6805
H	-1.22321	-4.1491	0.488106
H	0.884538	-5.40817	0.068582
C	0.674072	-1.65008	-0.9434
C	0.260238	-0.28294	-1.13667
C	1.042389	0.860496	-1.71863
C	0.160271	1.823742	-2.40987
C	1.994421	0.387521	-2.74219
N	-0.46079	2.637606	-2.9384
N	2.753705	0.033467	-3.53223
C	-1.07034	-0.16021	-0.66831
N	-1.45674	-1.41637	-0.18169
O	-1.85631	0.82863	-0.63018
C	-2.77993	-1.71587	0.315729
H	-2.69981	-2.22114	1.281307
H	-3.26229	-0.75043	0.484401
C	-3.60626	-2.55555	-0.63764
C	-4.44888	-3.55322	-0.14603
C	-3.55332	-2.33027	-2.01524
C	-5.23437	-4.31068	-1.01349
H	-4.48566	-3.74352	0.921765
C	-4.33398	-3.08714	-2.88408
H	-2.89202	-1.56545	-2.40536
C	-5.17855	-4.0792	-2.38587
H	-5.8809	-5.08589	-0.61834
H	-4.28143	-2.90536	-3.9514
H	-5.78292	-4.67095	-3.06344
C	-3.0831	3.723085	-0.84087
C	-3.65467	5.086863	-0.36474
H	-2.55767	3.783207	-1.79215

H	-3.83788	2.941954	-0.91217
H	-3.51403	5.846896	-1.13417
H	-4.72385	5.006133	-0.16465
C	-0.94479	4.244207	0.248163
H	-0.24982	3.862592	0.991823
H	-0.46222	4.244489	-0.72724
C	-1.5392	5.631086	0.622762
H	-1.05969	6.01755	1.522807
H	-1.37234	6.348717	-0.18137
H	-1.75766	2.301321	-0.112
C	-3.23133	4.558158	1.937179
H	-2.71421	4.897267	2.835804
H	-4.30034	4.538754	2.152498
C	-2.73418	3.145956	1.523315
H	-1.98726	2.729661	2.19511
H	-3.54472	2.427052	1.41253
N	-2.08108	3.270441	0.179282
N	-2.9849	5.533462	0.865406
H	1.119528	2.281089	-0.0707
H	2.449583	2.4837	-1.23506

TS3

Optimization energy :

E(B3LYP/6-311G(d,p)/IEFPCM(THF)) = -2083.548161 Hartree

Number of imaginary frequency=1(-325.11)

Single point energy :

E(M06-2X-D3/def2-TZVPP/SMD(THF))= -2082.811279 Hartree

Thermal correction to Gibbs Free Energy= 7.0 kcal/mol

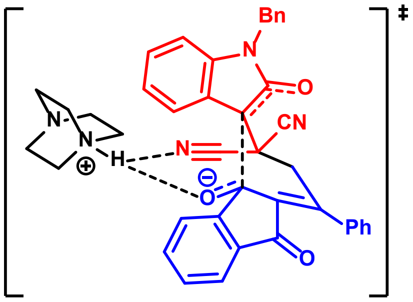

Symbol	X	Y	Z
C	0.855369	-1.40272	3.928853
C	1.161733	-0.70385	2.762837
C	0.201745	0.054876	2.09275
C	-1.08448	0.170291	2.606121
C	-1.40219	-0.53598	3.764649
C	-0.44347	-1.32403	4.418549
H	1.617952	-1.98429	4.432735
H	-1.82635	0.777256	2.103761
H	-2.40564	-0.47573	4.170352
H	-0.71829	-1.86276	5.317452
C	0.800279	0.703798	0.869807
C	2.476929	-0.59567	2.08324
C	2.237173	0.198139	0.841586
O	3.519722	-1.07574	2.496607
C	3.007376	0.387713	-0.25093
C	2.307674	1.072034	-1.3961
C	4.414038	-0.01968	-0.46404
C	5.276703	0.863859	-1.1341
C	4.917735	-1.26636	-0.06724
C	6.601684	0.522058	-1.38172
H	4.914448	1.836329	-1.44579
C	6.236441	-1.61573	-0.33621
H	4.280299	-1.96084	0.45591
C	7.085653	-0.72376	-0.98857
H	7.252895	1.226435	-1.88566

H	6.601845	-2.58977	-0.03258
H	8.114579	-0.99771	-1.18984
O	0.504416	1.919904	0.58019
C	1.463369	-3.95476	0.00569
C	1.550055	-2.63833	-0.45672
C	-0.77976	-2.35527	0.096925
C	-0.88038	-3.65521	0.567409
C	0.267671	-4.45208	0.523234
H	2.337451	-4.59301	-0.04122
H	2.48183	-2.27464	-0.8645
H	-1.81754	-4.04575	0.941494
H	0.219405	-5.47275	0.883524
C	0.428706	-1.81887	-0.39323
C	0.196669	-0.38873	-0.66936
C	0.940484	0.334773	-1.81043
C	0.136859	1.389065	-2.45713
C	1.258478	-0.6372	-2.87164
N	-0.36381	2.253244	-3.03119
N	1.534728	-1.39083	-3.69796
C	-1.26606	-0.20117	-0.51467
N	-1.77272	-1.37801	0.014991
O	-1.93383	0.816025	-0.69367
C	-3.14373	-1.54109	0.451015
H	-3.15471	-1.83776	1.503762
H	-3.59938	-0.55106	0.380636
C	-3.92006	-2.54011	-0.3821
C	-4.85243	-3.38271	0.224476
C	-3.73214	-2.6173	-1.76372
C	-5.59448	-4.28366	-0.53712
H	-4.99425	-3.33858	1.29932
C	-4.46904	-3.51909	-2.526
H	-2.99988	-1.97634	-2.24066
C	-5.40397	-4.35422	-1.91523
H	-6.31303	-4.935	-0.05313
H	-4.31122	-3.57257	-3.59694
H	-5.97455	-5.05884	-2.50878
C	-2.59521	3.669531	-0.83441
C	-3.23166	5.080775	-0.69846
H	-2.25533	3.443989	-1.84215
H	-3.24772	2.865083	-0.50776

H	-3.33486	5.546104	-1.6795
H	-4.22604	5.011633	-0.255
C	-0.37536	4.642931	-0.43152
H	0.478716	4.565477	0.237504
H	-0.06906	4.331073	-1.4276
C	-1.05312	6.039637	-0.41252
H	-0.46815	6.73836	0.186812
H	-1.126	6.443476	-1.4231
H	-0.90802	2.703202	0.056202
C	-2.33315	5.387503	1.503839
H	-1.69527	6.030104	2.112092
H	-3.33166	5.39377	1.942413
C	-1.76932	3.9408	1.46365
H	-0.8757	3.801358	2.068304
H	-2.50702	3.194692	1.755876
N	-1.38451	3.640083	0.046743
N	-2.40797	5.954931	0.150712
H	2.011146	2.070905	-1.08567
H	2.929611	1.129575	-2.28529

Int-7

Optimization energy :

E(B3LYP/6-311G(d,p)/IEFPCM(THF)) = -2083.567457 Hartree

Single point energy :

E(M06-2X-D3/def2-TZVPP/SMD(THF))= -2082.834771 Hartree

Thermal correction to Gibbs Free Energy= -7.7 kcal/mol

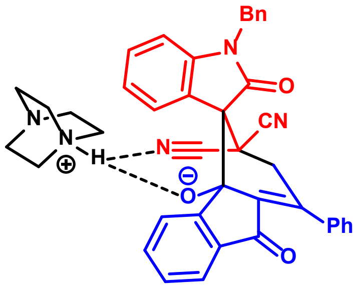

Symbol	X	Y	Z
C	2.069282	1.812751	3.386683
C	1.981305	1.249334	2.116766
C	0.748399	0.996329	1.50938
C	-0.43034	1.297603	2.178161
C	-0.34902	1.850372	3.457108
C	0.888002	2.112348	4.057404
H	3.038926	1.998942	3.83257
H	-1.39785	1.116204	1.732426
H	-1.26128	2.083942	3.99328
H	0.920721	2.544774	5.049936
C	0.969178	0.524312	0.073823
C	3.09383	0.810343	1.237131
C	2.434766	0.107394	0.091253
O	4.281067	0.975218	1.438965
C	2.961863	-0.80035	-0.73765
C	2.104811	-1.58374	-1.69977
C	4.378681	-1.24913	-0.70873
C	5.171576	-1.12475	-1.85449
C	4.916722	-1.8572	0.430062
C	6.487215	-1.58028	-1.8532
H	4.766144	-0.65078	-2.74096
C	6.225975	-2.32557	0.424761
H	4.311577	-1.95696	1.322327
C	7.015988	-2.18535	-0.71536
H	7.096522	-1.46396	-2.74156
H	6.630693	-2.79737	1.312293
H	8.03756	-2.54647	-0.71652

O	0.882451	1.608874	-0.81773
C	0.548655	-3.49069	2.208277
C	0.936649	-2.57612	1.223597
C	-1.36023	-1.88425	1.038184
C	-1.76438	-2.77919	2.016081
C	-0.78436	-3.58776	2.59766
H	1.295048	-4.12471	2.669857
H	1.977621	-2.50283	0.94856
H	-2.8012	-2.8589	2.311753
H	-1.07191	-4.30008	3.361381
C	-0.02404	-1.77195	0.62876
C	0.068073	-0.65899	-0.39699
C	0.616919	-1.12752	-1.7985
C	0.504981	-0.09682	-2.85294
C	-0.19071	-2.27174	-2.25245
N	0.480239	0.566039	-3.79405
N	-0.82739	-3.17119	-2.58586
C	-1.41696	-0.23592	-0.55966
N	-2.15789	-0.96871	0.333637
O	-1.85719	0.579101	-1.34487
C	-3.59737	-0.81905	0.493333
H	-3.81212	-0.54716	1.530427
H	-3.87933	0.025085	-0.13843
C	-4.37216	-2.06106	0.107016
C	-5.45524	-2.47625	0.882661
C	-4.02817	-2.79189	-1.03189
C	-6.19107	-3.60391	0.52309
H	-5.72169	-1.91887	1.774509
C	-4.7578	-3.92207	-1.38818
H	-3.18308	-2.48897	-1.63698
C	-5.84238	-4.33064	-0.61292
H	-7.02913	-3.91789	1.134304
H	-4.47596	-4.4848	-2.27033
H	-6.40831	-5.21221	-0.89007
C	-1.02827	3.875984	-1.94824
C	-1.6875	5.27752	-2.10996
H	-0.02706	3.845262	-2.3829
H	-1.61544	3.083681	-2.41418
H	-1.00268	5.982093	-2.58773
H	-2.58803	5.21918	-2.72591

C	-0.11961	4.59752	0.169857
H	-0.00236	4.305575	1.21588
H	0.873053	4.613876	-0.28455
C	-0.84566	5.969329	0.025626
H	-1.13879	6.363438	1.001738
H	-0.19712	6.70845	-0.45046
H	0.097691	2.206411	-0.60512
C	-2.97018	4.873112	-0.13005
H	-3.28516	5.305729	0.822784
H	-3.85929	4.761223	-0.7552
C	-2.26105	3.500808	0.085308
H	-2.15154	3.276386	1.147583
H	-2.80248	2.679291	-0.38405
N	-0.90901	3.547022	-0.50863
N	-2.06141	5.823067	-0.79311
H	2.543248	-1.5747	-2.69902
H	2.122301	-2.62931	-1.37843

Int-8

Optimization energy :

E(B3LYP/6-311G(d,p)/IEFPCM(THF)) = -2083.577765 Hartree

Single point energy :

E(M06-2X-D3/def2-TZVPP/SMD(THF))= -2082.846078 Hartree

Thermal correction to Gibbs Free Energy= -14.8 kcal/mol

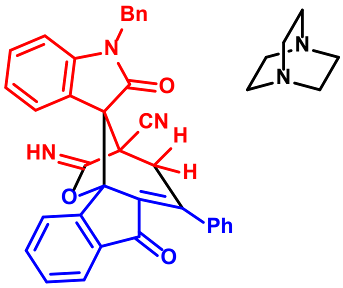

Symbol	X	Y	Z
C	-0.74617	4.84847	-1.26619
C	-0.30138	3.5587	-0.98807
C	-0.90758	2.442517	-1.56718
C	-1.95449	2.588419	-2.46889
C	-2.40017	3.879719	-2.75083
C	-1.80809	4.999177	-2.15145
H	-0.26461	5.70169	-0.80446
H	-2.40444	1.730111	-2.94955
H	-3.21656	4.019991	-3.44903
H	-2.17801	5.989109	-2.38929
C	-0.2561	1.185473	-1.06902
C	0.834686	3.153625	-0.11771
C	0.852202	1.664821	-0.14651
O	1.550105	3.912036	0.511066
C	1.523387	0.781538	0.620598
C	1.093624	-0.66753	0.63912
C	2.668715	1.116307	1.487029
C	3.701755	1.933897	1.008151
C	2.786332	0.562749	2.770168
C	4.823155	2.185141	1.788149
H	3.627487	2.353824	0.014987
C	3.899588	0.8339	3.558781
H	2.004191	-0.07301	3.16495
C	4.924806	1.63878	3.067421
H	5.619798	2.806648	1.397376
H	3.969466	0.40853	4.552577

H	5.799079	1.837505	3.675704
O	0.317546	0.417273	-2.16633
C	-2.1588	1.426787	3.184311
C	-1.2778	1.081492	2.153641
C	-3.17639	0.447769	0.820438
C	-4.06471	0.784109	1.829117
C	-3.53285	1.278684	3.022655
H	-1.76123	1.814373	4.113757
H	-0.21901	1.225131	2.301012
H	-5.1312	0.659926	1.70288
H	-4.20347	1.548008	3.829582
C	-1.78571	0.581186	0.96185
C	-1.14289	0.141662	-0.33226
C	-0.06893	-0.99226	-0.33412
C	0.464639	-0.87452	-1.77939
C	-0.61386	-2.30853	-0.04784
N	1.010658	-1.72174	-2.5299
N	-1.05083	-3.34759	0.191128
C	-2.36198	-0.29726	-1.18807
N	-3.4885	-0.05469	-0.45274
O	-2.32693	-0.78847	-2.29999
C	-4.83073	-0.37743	-0.91617
H	-5.4557	0.515395	-0.8407
H	-4.7277	-0.62682	-1.9735
C	-5.45192	-1.52657	-0.14986
C	-6.7742	-1.44572	0.287026
C	-4.71455	-2.68306	0.115914
C	-7.35742	-2.50737	0.976916
H	-7.34951	-0.54707	0.09113
C	-5.29369	-3.74133	0.809196
H	-3.68556	-2.75984	-0.21398
C	-6.61715	-3.65697	1.241251
H	-8.38491	-2.43197	1.313153
H	-4.71012	-4.63154	1.012552
H	-7.06623	-4.48098	1.783117
C	3.99261	-3.30671	-0.81048
C	5.442213	-3.60287	-1.31027
H	3.697161	-3.9913	-0.01121
H	3.268282	-3.40509	-1.62191
H	5.885045	-4.44072	-0.76554

H	5.448483	-3.85615	-2.37346
C	4.871175	-1.78008	0.821286
H	4.787577	-0.76348	1.20572
H	4.573994	-2.4637	1.620843
C	6.317415	-2.08194	0.319985
H	6.969456	-1.21535	0.457673
H	6.759735	-2.92093	0.863603
H	1.020425	-2.64169	-2.09412
C	5.726746	-1.28812	-1.86238
H	6.378647	-0.42226	-1.71961
H	5.73792	-1.54131	-2.92563
C	4.276698	-0.99116	-1.3692
H	4.19112	0.023336	-0.97534
H	3.544926	-1.1014	-2.17131
N	3.913078	-1.93055	-0.29027
N	6.29999	-2.4214	-1.11373
H	1.963517	-1.29487	0.392338
H	0.782156	-0.93206	1.653421

TS4

Optimization energy :

E(B3LYP/6-311G(d,p)/IEFPCM(THF)) = -2083.556239 Hartree

Number of imaginary frequency=1(-1575.59)

Single point energy :

E(M06-2X-D3/def2-TZVPP/SMD(THF))= - -2082.822445 Hartree

Thermal correction to Gibbs Free Energy= 0.0 kcal/mol

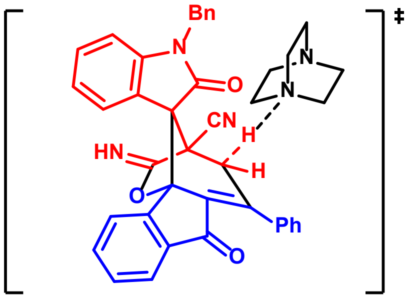

Symbol	X	Y	Z
C	-0.5285	4.837404	-1.33761
C	-0.11269	3.540704	-1.05675
C	-0.73932	2.439373	-1.64148
C	-1.77801	2.606362	-2.54829
C	-2.19509	3.907682	-2.83511
C	-1.58224	5.012073	-2.23191
H	-0.03327	5.680284	-0.87066
H	-2.245	1.756089	-3.02743
H	-3.00447	4.06519	-3.5381
H	-1.92876	6.010569	-2.47093
C	-0.11311	1.176127	-1.12507
C	1.007768	3.10103	-0.1676
C	1.000029	1.639147	-0.21578
O	1.728507	3.865161	0.46547
C	1.611764	0.708246	0.586375
C	1.2465	-0.68723	0.498348
C	2.683364	1.055957	1.552083
C	3.77613	1.832706	1.147605
C	2.665467	0.5438	2.855879
C	4.837555	2.065179	2.014546
H	3.792587	2.241551	0.146997
C	3.718558	0.794767	3.730827
H	1.823059	-0.04781	3.192571
C	4.813427	1.545526	3.308804
H	5.683323	2.654972	1.681564

H	3.685483	0.398855	4.738845
H	5.640032	1.729947	3.984678
O	0.419438	0.355548	-2.21786
C	-1.95218	1.503429	3.164011
C	-1.10133	1.140498	2.11328
C	-3.04059	0.526351	0.833051
C	-3.89954	0.878744	1.86153
C	-3.33166	1.372803	3.039079
H	-1.52585	1.891942	4.080533
H	-0.03814	1.267595	2.230734
H	-4.97046	0.767368	1.761753
H	-3.97821	1.655536	3.861161
C	-1.64444	0.641306	0.937089
C	-1.03885	0.175204	-0.36192
C	0.004076	-1.00245	-0.36404
C	0.439786	-0.94385	-1.84571
C	-0.57263	-2.28335	-0.00085
N	0.835063	-1.84437	-2.63432
N	-1.02713	-3.30175	0.291107
C	-2.28127	-0.2338	-1.19072
N	-3.38953	0.022576	-0.42859
O	-2.28926	-0.71959	-2.30728
C	-4.74547	-0.2709	-0.86455
H	-5.34988	0.63591	-0.78384
H	-4.66774	-0.52847	-1.92231
C	-5.38223	-1.40119	-0.08243
C	-6.71214	-1.30671	0.328434
C	-4.65456	-2.55391	0.222583
C	-7.3123	-2.35132	1.029549
H	-7.28007	-0.41009	0.103501
C	-5.25075	-3.59535	0.927149
H	-3.61908	-2.63986	-0.08336
C	-6.58175	-3.49791	1.332051
H	-8.34551	-2.26491	1.345191
H	-4.67384	-4.48246	1.161176
H	-7.04405	-4.30853	1.882987
C	3.432955	-3.21019	-0.91926
C	4.853091	-3.77241	-1.21914
H	2.897177	-3.79539	-0.17056
H	2.827512	-3.15762	-1.82072

H	5.112061	-4.57145	-0.52223
H	4.891842	-4.18283	-2.22944
C	4.487937	-1.81907	0.77292
H	4.489897	-0.81763	1.195762
H	4.065628	-2.50152	1.511427
C	5.902813	-2.25836	0.301464
H	6.605102	-1.42575	0.371978
H	6.281479	-3.07032	0.924582
H	0.734815	-2.76037	-2.20193
C	5.480667	-1.57856	-1.95587
H	6.279317	-0.83535	-1.93165
H	5.392889	-1.94294	-2.98076
C	4.137197	-0.95352	-1.47815
H	4.273188	0.040378	-1.05313
H	3.397229	-0.89199	-2.2752
N	3.574447	-1.82074	-0.40587
N	5.863096	-2.71086	-1.09659
H	2.436799	-1.31994	-0.03565
H	1.243115	-1.20996	1.453066

Int-9

Optimization energy :

E(B3LYP/6-311G(d,p)/IEFPCM(THF)) = - -2083.567598 Hartree

Single point energy :

E(M06-2X-D3/def2-TZVPP/SMD(THF))= -2082.838869 Hartree

Thermal correction to Gibbs Free Energy= -10.3 kcal/mol

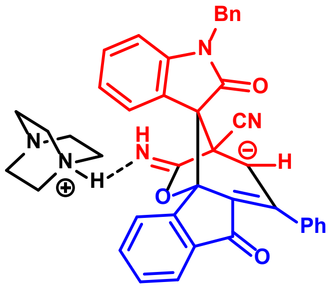

Symbol	X	Y	Z
C	-0.16857	4.411714	-1.74629
C	0.135974	3.149131	-1.25563
C	-0.47388	2.010119	-1.78245
C	-1.3769	2.097183	-2.83208
C	-1.67901	3.367116	-3.33461
C	-1.08688	4.511724	-2.79333
H	0.304841	5.288666	-1.32056
H	-1.82979	1.210668	-3.25708
H	-2.38129	3.464286	-4.15424
H	-1.34216	5.485203	-3.19625
C	0.006144	0.815027	-1.01338
C	1.097428	2.774098	-0.15975
C	1.039387	1.343656	-0.0638
O	1.75149	3.613515	0.476301
C	1.532113	0.431562	0.902368
C	0.995099	-0.84054	0.96918
C	2.616875	0.790173	1.854506
C	3.671087	1.62698	1.462429
C	2.647064	0.241818	3.144436
C	4.743005	1.868499	2.315158
H	3.640904	2.0943	0.489552
C	3.716969	0.48772	4.000619
H	1.821431	-0.37186	3.482479
C	4.775418	1.292867	3.584999
H	5.55291	2.510352	1.988788
H	3.719884	0.055156	4.99426
H	5.610352	1.482695	4.249175

O	0.57756	-0.21846	-1.90061
C	-2.21533	1.953421	2.911299
C	-1.3033	1.339425	2.043822
C	-3.15437	0.729194	0.631305
C	-4.07159	1.331824	1.476699
C	-3.57823	1.949291	2.630298
H	-1.85041	2.438066	3.80849
H	-0.25009	1.360351	2.271534
H	-5.13098	1.318487	1.25985
H	-4.27141	2.428163	3.311676
C	-1.77548	0.717513	0.896414
C	-1.08842	0.002379	-0.23233
C	-0.16397	-1.22677	0.064152
C	0.448822	-1.43686	-1.33762
C	-0.88761	-2.38497	0.557418
N	0.904818	-2.47431	-1.90093
N	-1.46444	-3.30331	0.948128
C	-2.26524	-0.43725	-1.12849
N	-3.41604	0.058867	-0.57392
O	-2.20366	-1.12049	-2.13604
C	-4.73832	-0.18071	-1.12696
H	-5.24827	0.776224	-1.26205
H	-4.57311	-0.61406	-2.11518
C	-5.57483	-1.1086	-0.26973
C	-6.92579	-0.83608	-0.05271
C	-5.01077	-2.25336	0.298619
C	-7.70745	-1.6975	0.715427
H	-7.36797	0.056965	-0.48179
C	-5.78856	-3.11168	1.069805
H	-3.96165	-2.47529	0.145207
C	-7.13973	-2.83722	1.279831
H	-8.7552	-1.47326	0.87865
H	-5.3376	-3.99418	1.508456
H	-7.74373	-3.50494	1.882936
C	3.915441	-3.09314	-1.12156
C	5.384217	-3.20263	-1.61527
H	3.650246	-3.86215	-0.3976
H	3.184002	-3.09387	-1.92597
H	5.919473	-3.97556	-1.06268
H	5.400697	-3.47127	-2.67159

C	4.804295	-1.65927	0.674453
H	4.589387	-0.75919	1.242042
H	4.660544	-2.52801	1.31485
C	6.20052	-1.63031	-0.0015
H	6.654188	-0.64501	0.111807
H	6.859806	-2.36117	0.467251
H	0.696265	-3.30128	-1.34424
C	5.342779	-0.85604	-2.10267
H	5.925323	0.064361	-2.05851
H	5.21742	-1.12592	-3.15154
C	3.962555	-0.64495	-1.42746
H	3.898342	0.285918	-0.87086
H	3.123985	-0.69589	-2.11746
N	3.780828	-1.76139	-0.42837
N	6.093506	-1.92908	-1.43533
H	2.837754	-1.66043	-0.01969
H	1.316369	-1.566	1.702377

TS5

Optimization energy :

E(B3LYP/6-311G(d,p)/IEFPCM(THF)) = -2083.56246 Hartree

Number of imaginary frequency=1(-254.28)

Single point energy :

E(M06-2X-D3/def2-TZVPP/SMD(THF))= -2082.821552 Hartree

Thermal correction to Gibbs Free Energy= 0.6 kcal/mol

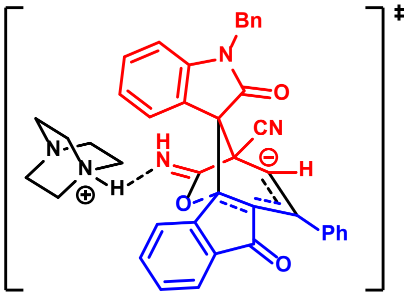

Symbol	X	Y	Z
C	-0.5285	4.837404	-1.33761
C	-0.11269	3.540704	-1.05675
C	-0.73932	2.439373	-1.64148
C	-1.77801	2.606362	-2.54829
C	-2.19509	3.907682	-2.83511
C	-1.58224	5.012073	-2.23191
H	-0.03327	5.680284	-0.87066
H	-2.245	1.756089	-3.02743
H	-3.00447	4.06519	-3.5381
H	-1.92876	6.010569	-2.47093
C	-0.11311	1.176127	-1.12507
C	1.007768	3.10103	-0.1676
C	1.000029	1.639147	-0.21578
O	1.728507	3.865161	0.46547
C	1.611764	0.708246	0.586375
C	1.2465	-0.68723	0.498348
C	2.683364	1.055957	1.552083
C	3.77613	1.832706	1.147605
C	2.665467	0.5438	2.855879
C	4.837555	2.065179	2.014546
H	3.792587	2.241551	0.146997
C	3.718558	0.794767	3.730827
H	1.823059	-0.04781	3.192571
C	4.813427	1.545526	3.308804
H	5.683323	2.654972	1.681564

H	3.685483	0.398855	4.738845
H	5.640032	1.729947	3.984678
O	0.419438	0.355548	-2.21786
C	-1.95218	1.503429	3.164011
C	-1.10133	1.140498	2.11328
C	-3.04059	0.526351	0.833051
C	-3.89954	0.878744	1.86153
C	-3.33166	1.372803	3.039079
H	-1.52585	1.891942	4.080533
H	-0.03814	1.267595	2.230734
H	-4.97046	0.767368	1.761753
H	-3.97821	1.655536	3.861161
C	-1.64444	0.641306	0.937089
C	-1.03885	0.175204	-0.36192
C	0.004076	-1.00245	-0.36404
C	0.439786	-0.94385	-1.84571
C	-0.57263	-2.28335	-0.00085
N	0.835063	-1.84437	-2.63432
N	-1.02713	-3.30175	0.291107
C	-2.28127	-0.2338	-1.19072
N	-3.38953	0.022576	-0.42859
O	-2.28926	-0.71959	-2.30728
C	-4.74547	-0.2709	-0.86455
H	-5.34988	0.63591	-0.78384
H	-4.66774	-0.52847	-1.92231
C	-5.38223	-1.40119	-0.08243
C	-6.71214	-1.30671	0.328434
C	-4.65456	-2.55391	0.222583
C	-7.3123	-2.35132	1.029549
H	-7.28007	-0.41009	0.103501
C	-5.25075	-3.59535	0.927149
H	-3.61908	-2.63986	-0.08336
C	-6.58175	-3.49791	1.332051
H	-8.34551	-2.26491	1.345191
H	-4.67384	-4.48246	1.161176
H	-7.04405	-4.30853	1.882987
C	3.432955	-3.21019	-0.91926
C	4.853091	-3.77241	-1.21914
H	2.897177	-3.79539	-0.17056
H	2.827512	-3.15762	-1.82072

H	5.112061	-4.57145	-0.52223
H	4.891842	-4.18283	-2.22944
C	4.487937	-1.81907	0.77292
H	4.489897	-0.81763	1.195762
H	4.065628	-2.50152	1.511427
C	5.902813	-2.25836	0.301464
H	6.605102	-1.42575	0.371978
H	6.281479	-3.07032	0.924582
H	0.734815	-2.76037	-2.20193
C	5.480667	-1.57856	-1.95587
H	6.279317	-0.83535	-1.93165
H	5.392889	-1.94294	-2.98076
C	4.137197	-0.95352	-1.47815
H	4.273188	0.040378	-1.05313
H	3.397229	-0.89199	-2.2752
N	3.574447	-1.82074	-0.40587
N	5.863096	-2.71086	-1.09659
H	2.436799	-1.31994	-0.03565
H	1.243115	-1.20996	1.453066

Int-10

Optimization energy :

E(B3LYP/6-311G(d,p)/IEFPCM(THF)) = -2083.591858 Hartree

Single point energy :

E(M06-2X-D3/def2-TZVPP/SMD(THF))= -2082.851226 Hartree

Thermal correction to Gibbs Free Energy= -18.0 kcal/mol

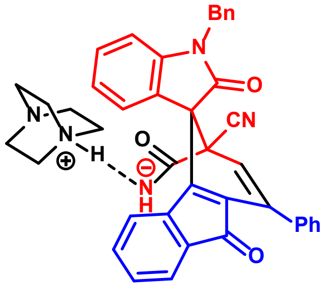

Symbol	X	Y	Z
C	3.137837	0.994853	3.343864
C	2.533976	0.292684	2.32444
C	1.154412	0.382493	2.069188
C	0.353052	1.176759	2.872553
C	0.961429	1.892531	3.918034
C	2.329503	1.813058	4.15046
H	4.204459	0.911769	3.514979
H	-0.7113	1.269031	2.712921
H	0.347587	2.522276	4.55047
H	2.772725	2.380707	4.9591
C	0.866908	-0.46104	0.886551
C	3.138559	-0.64858	1.328078
C	1.994722	-1.07945	0.44509
O	4.304901	-0.97042	1.264375
C	1.968393	-2.02163	-0.67445
C	0.866532	-2.0617	-1.44789
C	3.092753	-2.95131	-0.96646
C	4.372791	-2.48099	-1.27899
C	2.850107	-4.32924	-0.99082
C	5.385721	-3.37289	-1.61387
H	4.574434	-1.4189	-1.26106
C	3.868231	-5.22208	-1.31746
H	1.861215	-4.69878	-0.74609
C	5.138328	-4.74544	-1.63086
H	6.370864	-2.99587	-1.86226
H	3.667448	-6.2869	-1.32594
H	5.931931	-5.43758	-1.88661

O	-1.1858	0.713157	-2.58043
C	-1.7559	-3.94367	2.006633
C	-0.82481	-3.11097	1.376567
C	-2.50047	-1.3698	1.332981
C	-3.43907	-2.18391	1.946695
C	-3.04404	-3.48426	2.277427
H	-1.47397	-4.95193	2.282446
H	0.176694	-3.46194	1.16369
H	-4.4401	-1.8338	2.158715
H	-3.75714	-4.14377	2.757007
C	-1.21322	-1.82908	1.032714
C	-0.47681	-0.7318	0.28094
C	-0.30969	-1.12541	-1.26171
C	-0.17775	0.187761	-2.14068
C	-1.52602	-1.80792	-1.71349
N	1.073626	0.640425	-2.30562
N	-2.45468	-2.37679	-2.08717
C	-1.46216	0.464671	0.421217
N	-2.62973	-0.02539	0.945214
O	-1.23312	1.623621	0.145041
C	-3.83006	0.783669	1.110436
H	-4.19004	0.679118	2.136075
H	-3.51475	1.8186	0.968858
C	-4.91124	0.406742	0.120289
C	-6.21563	0.171073	0.553781
C	-4.61013	0.302351	-1.24071
C	-7.21444	-0.162	-0.36028
H	-6.45192	0.244617	1.610154
C	-5.60556	-0.03568	-2.15174
H	-3.5986	0.473641	-1.59077
C	-6.91004	-0.26741	-1.71496
H	-8.2243	-0.34607	-0.01255
H	-5.3609	-0.12252	-3.20377
H	-7.68265	-0.53347	-2.42685
C	2.613118	4.328861	-2.12349
C	2.627257	5.571693	-1.17739
H	3.57494	3.809888	-2.11497
H	2.395094	4.613804	-3.15537
H	3.621136	5.730786	-0.75144
H	2.347715	6.479689	-1.71754

C	1.901972	2.923898	-0.30471
H	1.10688	2.252951	0.012964
H	2.835779	2.356353	-0.34293
C	2.016371	4.147635	0.649064
H	1.340483	4.039948	1.500974
H	3.030764	4.251877	1.042498
H	1.229861	1.654103	-2.43696
C	0.315949	5.245491	-0.6315
H	-0.37468	5.07113	0.197347
H	0.038429	6.19138	-1.10374
C	0.26793	4.067844	-1.65204
H	-0.49352	3.335626	-1.38638
H	0.06259	4.423833	-2.66442
N	1.57787	3.378274	-1.67653
N	1.672482	5.386565	-0.07094
H	0.817997	-2.73167	-2.29613
H	1.827036	0.132672	-1.87011

TS6

Optimization energy :

E(B3LYP/6-311G(d,p)/IEFPCM(THF)) = -2083.555192 Hartree

Number of imaginary frequency=1(-251.16)

Single point energy :

E(M06-2X-D3/def2-TZVPP/SMD(THF))= -2082.811743 Hartree

Thermal correction to Gibbs Free Energy= 6.8 kcal/mol

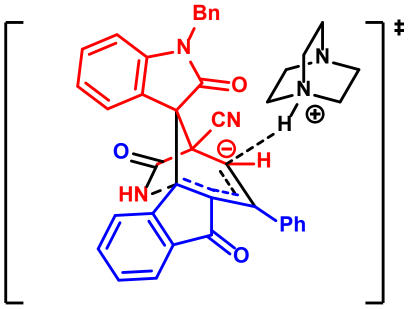

Symbol	X	Y	Z
C	2.236173	1.533706	3.791611
C	2.116573	0.975245	2.535732
C	0.868761	0.84768	1.909419
C	-0.28761	1.283373	2.535206
C	-0.17181	1.855451	3.811106
C	1.069121	1.980438	4.430547
H	3.206448	1.613745	4.267324
H	-1.2564	1.207596	2.062436
H	-1.06236	2.20589	4.318807
H	1.131893	2.423042	5.417565
C	1.099982	0.20545	0.594962
C	3.181827	0.395475	1.633752
C	2.47815	-0.05787	0.44253
O	4.369861	0.333787	1.931759
C	2.930913	-0.7094	-0.76439
C	2.028375	-1.0259	-1.7288
C	4.359845	-1.04187	-1.00052
C	5.363397	-0.07811	-0.84932
C	4.714906	-2.3262	-1.43024
C	6.687735	-0.38968	-1.13558
H	5.103488	0.913555	-0.50617
C	6.043617	-2.6411	-1.70528
H	3.945233	-3.08162	-1.53605
C	7.033438	-1.67218	-1.56194
H	7.453639	0.36876	-1.02205

H	6.303327	-3.64248	-2.02838
H	8.067943	-1.91395	-1.77624
O	0.272052	1.161193	-3.13649
C	0.810143	-4.17394	1.565412
C	1.121975	-2.99694	0.876423
C	-1.19539	-2.3555	1.048816
C	-1.52302	-3.51294	1.736552
C	-0.49386	-4.42549	1.98632
H	1.592774	-4.89211	1.775453
H	2.137451	-2.79647	0.568993
H	-2.53518	-3.70983	2.061525
H	-0.71937	-5.34129	2.519247
C	0.106553	-2.09373	0.605663
C	0.113993	-0.74282	-0.08163
C	0.544373	-0.70232	-1.57533
C	0.44586	0.78094	-2.01188
C	-0.28352	-1.56767	-2.40399
N	0.729808	1.630134	-0.88401
N	-0.95155	-2.25304	-3.04489
C	-1.37519	-0.32565	0.003842
N	-2.04772	-1.29257	0.692318
O	-1.86553	0.668741	-0.50432
C	-3.48177	-1.25148	0.945576
H	-3.65143	-1.32817	2.022213
H	-3.81284	-0.26256	0.624024
C	-4.24138	-2.33723	0.212862
C	-5.2463	-3.05052	0.866949
C	-3.95763	-2.62626	-1.12369
C	-5.96509	-4.03713	0.194133
H	-5.46436	-2.83837	1.908325
C	-4.66994	-3.61565	-1.79445
H	-3.17358	-2.08907	-1.64244
C	-5.67679	-4.32298	-1.13818
H	-6.74191	-4.58635	0.713168
H	-4.43511	-3.83544	-2.82923
H	-6.2289	-5.09509	-1.66095
C	-0.19928	4.899046	-0.72417
C	-1.08478	6.181984	-0.77282
H	0.460482	4.896637	0.146824
H	0.421688	4.804626	-1.6182

H	-0.8785	6.832727	0.080083
H	-0.89455	6.754778	-1.68331
C	-1.90383	3.791825	0.569338
H	-2.50478	2.883695	0.61952
H	-1.24044	3.808092	1.436207
C	-2.78735	5.075031	0.496629
H	-3.84967	4.820358	0.511865
H	-2.59094	5.735672	1.344498
H	1.594567	2.147673	-1.04134
C	-2.80622	4.950986	-1.89605
H	-3.87199	4.711383	-1.87943
H	-2.60653	5.516584	-2.80913
C	-1.94144	3.65612	-1.83735
H	-2.55	2.755679	-1.75924
H	-1.30213	3.550913	-2.7158
N	-1.06533	3.703171	-0.64402
N	-2.51093	5.819817	-0.74314
H	-0.05461	2.350253	-0.72154
H	2.334318	-1.44449	-2.67667

3a

Optimization energy :

E(B3LYP/6-311G(d,p)/IEFPCM(THF)) = -2083.609976 Hartree

Single point energy :

E(M06-2X-D3/def2-TZVPP/SMD(THF))= -2082.87411 Hartree

Thermal correction to Gibbs Free Energy= -32.4 kcal/mol

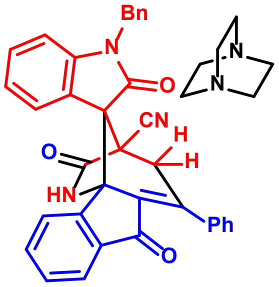

Symbol	X	Y	Z
C	-0.80347	4.778753	-1.32305
C	-0.32757	3.502603	-1.032
C	-0.89179	2.367968	-1.61799
C	-1.92814	2.483593	-2.53699
C	-2.40563	3.761054	-2.83068
C	-1.85438	4.898276	-2.22601
H	-0.35371	5.646141	-0.8555
H	-2.35033	1.611046	-3.01822
H	-3.2161	3.875991	-3.54039
H	-2.24786	5.876935	-2.47257
C	-0.21516	1.126089	-1.10206
C	0.805105	3.135057	-0.13912
C	0.862528	1.649812	-0.15472
O	1.488786	3.922299	0.492029
C	1.536784	0.784751	0.632847
C	1.119633	-0.66934	0.681009
C	2.677861	1.14456	1.494334
C	3.683045	1.996015	1.013636
C	2.825399	0.586376	2.772695
C	4.801396	2.279329	1.786671
H	3.58932	2.418345	0.023226
C	3.936681	0.886048	3.553578
H	2.067031	-0.07517	3.171112
C	4.931026	1.728107	3.061302
H	5.574585	2.928679	1.393826
H	4.028406	0.455269	4.543308
H	5.803071	1.950974	3.66447

O	1.052943	-1.93723	-2.31078
C	-2.16776	1.519178	3.119911
C	-1.27431	1.119388	2.119877
C	-3.1585	0.479239	0.771986
C	-4.05949	0.869789	1.749511
C	-3.54126	1.396176	2.935342
H	-1.77982	1.928725	4.044063
H	-0.216	1.242508	2.286651
H	-5.12562	0.761349	1.605934
H	-4.22165	1.708004	3.71849
C	-1.76831	0.590731	0.934144
C	-1.10959	0.095229	-0.33243
C	-0.04457	-1.04451	-0.27181
C	0.525663	-1.01641	-1.72628
C	-0.59935	-2.3395	0.077355
N	0.372619	0.263936	-2.13054
N	-1.06347	-3.35102	0.37561
C	-2.32288	-0.37044	-1.18627
N	-3.45755	-0.06993	-0.48452
O	-2.28508	-0.92185	-2.26981
C	-4.79599	-0.39863	-0.9517
H	-5.41055	0.504347	-0.93026
H	-4.68076	-0.70159	-1.99374
C	-5.44657	-1.50059	-0.14102
C	-6.79168	-1.40438	0.216643
C	-4.71754	-2.62903	0.241778
C	-7.40551	-2.42396	0.94219
H	-7.36098	-0.52619	-0.06934
C	-5.32792	-3.64519	0.970767
H	-3.67079	-2.71729	-0.02279
C	-6.67383	-3.54647	1.322328
H	-8.45052	-2.33673	1.215906
H	-4.75037	-4.51374	1.264817
H	-7.14707	-4.33753	1.892052
C	5.015504	-1.66515	0.807918
C	6.3809	-2.11938	0.203758
H	5.041186	-0.61861	1.115249
H	4.747384	-2.2614	1.683823
H	7.122832	-1.31824	0.259894
H	6.783835	-2.98376	0.738177

C	4.256076	-0.96299	-1.359
H	3.4631	-1.10649	-2.09482
H	4.231776	0.078363	-1.03032
C	5.652309	-1.34473	-1.9424
H	5.578693	-1.61244	-2.99972
H	6.358064	-0.51401	-1.85751
H	0.795615	0.621332	-2.97445
C	5.26103	-3.61889	-1.29503
H	5.133458	-3.87622	-2.34973
H	5.705972	-4.48241	-0.79356
C	3.899044	-3.22351	-0.64297
H	3.067476	-3.32222	-1.34297
H	3.680671	-3.84496	0.229621
N	3.950424	-1.81915	-0.19819
N	6.212686	-2.49544	-1.21151
H	2.003277	-1.2852	0.455214
H	0.817457	-0.90859	1.704171
